# Identification of mitochondria-related key genes in type 2 diabetes mellitus and elucidation of the Zhimu-Huangbai herb Pair’s mechanism: an integrated approach of bioinformatics, machine learning, and experimental validation

**DOI:** 10.3389/fcell.2026.1763178

**Published:** 2026-03-13

**Authors:** Sufang Zhang, Minghe Gu, Baoting Chen, Yiming Liu, Aihua Lin

**Affiliations:** 1 The Second Clinical College of Guangzhou University of Chinese Medicine, Guangzhou, China; 2 The Zhuhai Hospital of Guangdong Provincial Hospital of Chinese Medicine, Zhuhai, China; 3 Guangdong Provincial Key Laboratory of Clinical Research on Traditional Chinese Medicine Syndrome, Guangzhou, China

**Keywords:** bioinformatics, energy metabolism, machine learning, mitochondria, type 2 diabetes mellitus, zhimu-huangbai

## Abstract

**Background:**

Mitochondrial injury plays a critical role in type 2 diabetes mellitus (T2DM) pathogenesis by impairing cellular energy metabolism and insulin sensitivity. The Zhimu-Huangbai herb pair (ZB), a classic Traditional Chinese Medicine formulation composed of Anemarrhena asphodeloides and Phellodendron chinense, has shown efficacy in T2DM, but its molecular mechanisms remain unclear. In this study, we aimed to identify crucial mitochondrial related genes of type 2 diabetes and the potential mechanism of ZB.

**Methods:**

Gene expression datasets for T2DM (GSE76894, GSE25724, and GSE38642) were retrieved from the GEO database. Intersection targets of ZB herb pair and T2DM were identified by screening multiple databases, including the TCMSP and HERB. Mitochondrial function-related genes were obtained from human mitochondria-associated databases. WGCNA was employed to identify differentially expressed genes, which were then intersected with bioactive compound–target genes and mitochondrial-related genes to construct a PPI network. GO and KEGG enrichment analyses were subsequently performed. Four machine learning algorithms—SVM-RFE, RF, GLM, and XGB—were applied to screen feature genes and establish diagnostic models. Furthermore, the correlations between feature targets and immune cell infiltration were analyzed, single-gene GSEA was conducted, and molecular docking was performed to investigate the interactions between feature targets and bioactive constituents of ZB. For experimental validation, INS-1 cells were divided into six groups: the control group, model group, metformin group, and low-, medium-, and high-dose ZB groups. Cell viability, apoptosis, ROS levels, mitochondrial membrane potential, and mitochondrial morphology and function were assessed. Western blot analysis was performed to evaluate the expression of mitochondria-related genes (BCAT2, CASP8, EPHX2, and UCP2) and components of the AMPK–SIRT1–PGC-1α signaling pathway.

**Results:**

A total of eight mitochondria-related differentially expressed genes associated with ZB treatment of T2DM were identified. GO analysis revealed enrichment in multiple biological processes, including response to nutrient levels; cellular components, such as pore complex; and molecular functions, including toxic substance binding. KEGG pathway analysis indicated significant enrichment in pathways including apoptosis, p53 signaling pathway, and necroptosis. Three key genes—BCAT2, CASP8, and EPHX2—were screened through machine learning algorithms, and the constructed T2DM diagnostic models all exhibited area under the curve (AUC) values greater than 0.7, indicating satisfactory discriminative performance. Immune infiltration analysis revealed that all three key genes were significantly correlated with immune cell populations. Molecular docking results demonstrated that the three key genes exhibited strong binding affinities (≤−5.0 kcal/mol) for their corresponding bioactive compounds derived from ZB, with the exception of the CASP8-nicotinamide combination. Experimental validation showed that ZB significantly enhanced the viability of INS-1 cells subjected to high-glucose and high-lipid conditions, inhibited apoptosis, reduced intracellular ROS generation, and ameliorated mitochondrial membrane potential, mitochondrial morphology, and respiratory function. Concurrently, the protein expression levels of UCP2 and BCAT2 were markedly upregulated, whereas those of CASP8 and EPHX2 were significantly downregulated. Additionally, ZB treatment upregulated the p-AMPK/AMPK ratio as well as the expression of SIRT1 and PGC-1α.

**Conclusion:**

The diagnostic model featuring genes BCAT2, CASP8, and EPHX2 provides new insights for T2DM diagnosis and treatment. ZB’s therapeutic mechanism involves regulating mitochondrial-related genes (BCAT2, CASP8, EPHX2, UCP2) and activating the AMPK-SIRT1-PGC-1α pathway, thereby improving mitochondrial morphology and function, reducing oxidative damage, and enhancing energy metabolism.

## 1 Introduction

Type 2 diabetes mellitus (T2DM) is a multifactorial metabolic disease that originates from insulin resistance (IR), progresses through structural deterioration of pancreatic beta cells, and ultimately culminates in insufficient insulin secretion. These pathological alterations disrupt glucose and lipid metabolism, subsequently inflicting secondary damage on multiple organ systems (Kahn et al., 2014). According to the International Diabetes Federation (IDF), the global prevalence of diabetes has surpassed 537 million individuals, with approximately 90% of cases attributed to T2DM—a figure projected to reach 783 million by 2045 (Sun et al., 2022). Despite the development of numerous hypoglycemic agents with diverse mechanisms of action, certain pathological hallmarks of T2DM—including progressive β-cell failure, persistent insulin resistance, and systemic inflammation—remain inadequately addressed. Moreover, limitations in drug delivery modalities and the burden of adverse drug reactions continue to pose substantial challenges to the effective management of T2DM (Zhao et al., 2020).

Recent studies have demonstrated that mitochondrial dysfunction plays a central role in the onset and progression of T2DM. As the principal regulators of cellular energy metabolism and redox homeostasis, mitochondria, when impaired, can directly or indirectly drive the pathological progression of T2DM by compromising pancreatic β-cell function, disrupting insulin signaling pathways, and destabilizing systemic metabolic homeostasis (Walker et al., 2025). Indeed, impaired mitochondrial quality control, diminished ATP synthesis, and elevated reactive oxygen species (ROS) levels have been widely documented in both diabetic patients and animal models. Pharmacological agents targeting these mitochondrial processes have been shown to effectively ameliorate the aforementioned indices, restore mitochondrial function, and rectify metabolic dysregulation. Notably, established mitochondrial inhibitors such as berberine and metformin have been demonstrated to activate the AMPK signaling pathway by attenuating mitochondrial ATP production, thereby improving metabolic disorders—a mechanism that may partly underlie the insulin-sensitizing effects of these agents (Xu et al., 2025). Collectively, these findings suggest that targeting mitochondrial function represents a novel and promising therapeutic strategy for T2DM.

T2DM falls within the scope of “Xiaoke” (wasting-thirst syndrome) in Traditional Chinese Medicine (TCM), characterized by the pathological pattern of spleen deficiency as the root cause, with yin deficiency and dryness-heat as the secondary manifestations. Spleen-stomach dysfunction constitutes the fundamental etiology of Xiaoke, while yin-fire represents a critical factor in glycemic fluctuations among diabetic patients (Yang et al., 2020; Yang and Ji, 2023). Mitochondria serve as crucial organelles for energy production and transformation in the human body. In TCM theory, the spleen and stomach function as the acquired foundation of life, transforming food essence into energy to maintain normal physiological activities. Therefore, the mitochondrial oxidative phosphorylation process exhibits functional coupling with the spleen-stomach’s transforming and transporting capabilities. The “spleen-mitochondria correlation” hypothesis has gained widespread recognition among TCM scholars (Tan et al., 2025). With the gradual unfolding of the multi-component, multi-pathway, multi-target, and multifactorial characteristics inherent in Chinese herbal medicine, the therapeutic benefits of TCM for T2DM are gaining increasing recognition within the scientific community (Ni et al., 2025; Ni et al., 2024). Zhimu (Anemarrhenae Rhizoma), derived from the dried rhizome of *Anemarrhena asphodeloides* Bge. (Liliaceae), possesses fire-purging, yin-nourishing, heat-clearing, and dryness-moistening properties, thereby nourishing yin and generating body fluids. Huangbai (Phellodendri Chinensis Cortex), obtained from the dried bark of *Phellodendron chinense* Schneid. (Rutaceae), exhibits heat-clearing, dampness-drying, fire-purging, and detoxifying effects. The Zhimu-Huangbai herb pair (ZB) is derived from the ancient Chinese medical text “Lan Shi Mi Cang” (Secret Book of the Orchid Chamber) written by Li Gao. When used together, these two herbs enhance the effects of nourishing yin, clearing heat, removing dampness, and detoxifying. It is an effective herb pair widely used in traditional Chinese medicine for treating T2DM (Ding, 1995).

Our preliminary studies have confirmed that ZB exhibits significant hypoglycemic and hypolipidemic effects, possesses free radical scavenging capacity, ameliorates oxidative damage in pancreatic β-cells, and reduces ROS and lipid peroxidation levels while simultaneously enhancing mitochondrial membrane potential (Jin et al., 2019; Li, 2019). Notably, our research group previously discovered that mangiferin, a bioactive constituent of ZB, predominantly localizes to the nucleus in normal rat insulinoma cells (INS-1), whereas under oxidative stress conditions, it substantially redistributes to the cytoplasm and mitochondria (Fang et al., 2021). The intracellular distribution of a drug is intimately linked to its pharmacological activity, and this compelling finding strongly suggests that ZB may exert protective effects on pancreatic β-cells through regulation of mitochondrial quality control and function. However, the specific mitochondrial genes targeted by ZB in T2DM treatment, along with the associated signaling pathways and underlying mechanisms, remain to be elucidated. Therefore, investigating the key genes and bioactive components through which ZB modulates mitochondrial function holds significant clinical implications, providing theoretical and experimental foundations for understanding ZB’s therapeutic mechanisms in T2DM and offering a modern molecular biological interpretation of the TCM principle of “nourishing yin and clearing heat.”

**FIGURE 1:**
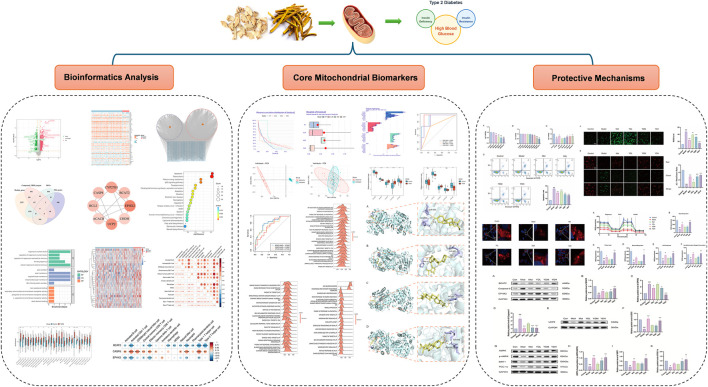
Technical workflow.

Bioinformatics, a discipline integrating biology, computer science, and information technology, has emerged as a powerful tool for analyzing biological sequencing datasets and solving complex biological problems. With the rapid advancement of high-throughput sequencing technologies, bioinformatics-based big data mining has been widely applied to identify disease-related biomarkers and elucidate underlying pathological mechanisms (Orlov et al., 2022). In this study, we employed bioinformatics approaches integrated with multiple machine learning algorithms to analyze mitochondrial gene expression data and identify key genes associated with ZB’s therapeutic effects in T2DM. Molecular docking simulations were utilized to screen the critical bioactive components of ZB, while *in vitro* experiments validated its protective effects against high glucose and high lipid (HGHL) -induced pancreatic β-cell damage, as well as its regulatory effects on mitochondrial-related genes and pathways. This study aims to understand ZB therapeutic effect in T2DM, providing mechanistic insights and establishing a scientific foundation for the clinical application of ZB. The technical workflow is illustrated in Figure 1.

## 2 Materials and methods

### 2.1 Bioinformatics analysis

#### 2.1.1 Data sources

T2DM gene expression datasets (GSE76894, GSE25724, and GSE38642) were downloaded from the Gene Expression Omnibus (GEO) database (https://www.ncbi.nlm.nih.gov/geo/). T2DM samples were derived from pancreatic islet β-cells and islet tissues. Specifically, the GSE76894 dataset comprised 103 pancreatic islet β-cell samples (19 T2DM samples and 84 healthy controls); the GSE25724 dataset included 13 islet tissue samples (6 T2DM and 7 controls); and the GSE38642 dataset contained 63 islet tissue samples (9 T2DM and 54 controls). For transcriptomic analysis, the large-scale dataset GSE76894 was designated as the training set, while GSE25724 and GSE38642 were merged after batch effect correction to serve as the validation set. A total of 1,136 mitochondria-related functional genes were obtained from the Human MitoCarta3.0 database (https://www.broadinstitute.org/files/shared/metabolism/mitocarta/human.mitocarta3.0.html).

#### 2.1.2 Screening of ZB bioactive components and prediction of potential target genes

The bioactive components of ZB were extracted from the Traditional Chinese Medicine Systems Pharmacology Database and Analysis Platform (TCMSP, https://old.tcmsp-e.com/index.php) as well as the High-throughput Experiment- and Reference-guided database of traditional Chinese medicine (HERB, http://herb.ac.cn/). Based on oral bioavailability (OB) ≥ 30% and drug-likeness (DL) ≥ 0.18, compounds were screened to identify the bioactive constituents of Zhimu and Huangbai, considering their pharmacological potential and therapeutic relevance. The target genes of these bioactive components were obtained via multiple databases, including the PubChem database (https://pubchem.ncbi.nlm.nih.gov/), Swiss Target Prediction database (http://swisstargetprediction.ch/), and UniProt database (https://www.uniprot.org/). After removing duplicate entries, the target genes of ZB bioactive components were consolidated.

#### 2.1.3 Acquisition of T2DM disease-related genes

Using “Type 2 diabetes mellitus” as the search term, T2DM-related target genes were retrieved from multiple databases including DisGeNET (https://www.disgenet.org/), OMIM (https://omim.org/), DrugBank (https://go.drugbank.com/), Comparative Toxicogenomics Database (CTD, https://ctdbase.org/), and the GeneCards (http://www.genecards.org/).

#### 2.1.4 Core target screening and construction of herb-active component-target network

After removing duplicates and merging compounds with their corresponding targets, the target compounds and target genes were obtained. Disease targets were deduplicated and merged, The “VennDiagram” package (Version1.7.3,https://cran.rproject.org/web/packages/VennDiagram/index.html) was utilized to intersect with drug targets, thereby identifying disease-associated drug targets through a more systematic analytical approach. The effective active component-target gene network was constructed using Cytoscape software (Version 3.9.1), with a focus on its robust analytical capabilities.

#### 2.1.5 Differential expression analysis

Utilizing the limma package (Version 3.63.13 https://bioconductor.org/packages//2.7/bioc/html/limma.html), differential expression analysis was conducted between T2DM and normal samples within the GSE76894 dataset, with statistical thresholds defined as P-value <0.05 and |log2FC| > 0.263 (Mao et al., 2021). Utilizing the “ComplexHeatmap” and “ggplot2” packages in R software, correlation heatmaps and volcano plots were respectively generated.

#### 2.1.6 Weighted gene Co-expression network analysis (WGCNA) and key module identification

WGCNA was conducted using the “WGCNA” package (Version 1.73, https://cran.r-project.org/web/packages/WGCNA/index.html) in R software. The “goodSamplesGenes” function was utilized for sample clustering analysis to remove outliers. The “pickSoftThreshold” function was applied to determine the optimal soft-threshold power by establishing the relationship between soft-threshold β and scale-free topology model fit R^2^, as well as the relationship between soft-threshold power and mean connectivity. Scale-free networks were constructed using the blockwiseModules function based on the selected soft threshold, with the minimum module size set to 50 genes. These genes were divided into several modules and distinguished by different colors. Subsequently, the labeledHeatmap function was employed to generate a module-trait relationship heatmap. Modules with absolute correlation values greater than 0.3 and *P* < 0.05 with T2DM were selected for subsequent analysis (Huang et al., 2023).

#### 2.1.7 Construction of protein-protein interaction (PPI) network and core gene screening

To identify intersecting targets among the aforementioned Differentially Expressed Genes (DEGs), effective active component targets, WGCNA genes, and mitochondrial genes, the “VennDiagram” package was employed. Through the STRING database (https://string-db.org/), PPI analysis was conducted with a protein interaction score threshold set at ≥0.15 (Szklarczyk et al., 2023). Imported into Cytoscape software (Version 3.9.1) for the visualization of core targets, the resulting TSV file was processed accordingly.

#### 2.1.8 Gene Ontology (GO) and KEGG enrichment analysis

The overlapping targets were subjected to GO functional annotation via the clusterProfiler package (Version 4.16.0), encompassing three categories: biological process (BP), cellular component (CC), and molecular function (MF). Pathway analysis was further carried out based on the Kyoto Encyclopedia of Genes and Genomes (KEGG) database to explore signaling pathways associated with the identified genes. Visualization of enrichment results was accomplished through the Weishengxin online platform, and statistical significance was defined as *P* < 0.05.

#### 2.1.9 Construction of predictive models based on multiple machine learning approaches

To screen mitochondria-associated diagnostic biomarkers for T2DM, four machine learning approaches were applied to the overlapping target genes: Support Vector Machine with Recursive Feature Elimination (SVM-RFE), Random Forest (RF), Generalized Linear Model (GLM), and Extreme Gradient Boosting (XGB). These algorithms were implemented through corresponding R packages, namely, “e1071”(Version 1.7.16), “randomForest” (Version 4.7.1.2), “stats” (Version 4.5.0), and “xgboost” (Version 1.7.10.1), respectively. Model interpretability was assessed via the “DALEX” package, where the “explain” function was utilized for model explanation, and variable importance was quantified accordingly. The predictive capacity of each model was evaluated by generating receiver operating characteristic (ROC) curves through the “pROC” package (Version 1.18.5), with the area under the curve (AUC) serving as the primary performance metric.

#### 2.1.10 Expression and ROC validation of feature targets

The expression profiles of candidate feature genes were retrieved from the datasets. GSE76894 was designated as the training cohort, whereas GSE25724 and GSE38642 were combined following batch effect correction via the “sva” package (Version 3.52.0) to constitute the external validation cohort. Boxplots illustrating gene expression distributions in both cohorts were visualized using the “ggplot2” package (Version 3.5.2). Statistical comparison between control and T2DM groups was conducted through the Wilcoxon rank-sum test, with *P* < 0.05 regarded as significant. Only genes demonstrating concordant expression patterns and statistical significance across both training and validation cohorts were retained as final feature targets. The diagnostic value of these targets was further evaluated by ROC curve analysis via the “pROC” package, with AUC values computed to quantify their predictive capability.

#### 2.1.11 Immune infiltration analysis

Immune infiltration analysis was performed on the GSE76894 dataset using the single-sample Gene Set Enrichment Analysis (ssGSEA) algorithm embedded in the “GSVA” package (Version 2.2.0). This approach enabled quantification of immune activity levels across individual samples, and subsequent comparisons of immune cell infiltration between T2DM patients and healthy controls were conducted. Spearman correlation analysis was implemented via the “psych” package (Version 2.5.3) to evaluate inter-relationships among infiltrating immune cell populations and to assess associations between feature target expression levels and immune cell abundance. Correlation patterns were visualized through heatmaps constructed with the “pheatmap” package (Version 1.0.12), facilitating systematic interpretation of the results. Correlations reaching *P* < 0.05 were considered statistically significant.

#### 2.1.12 Gene set enrichment analysis (GSEA) of core genes

To elucidate the signaling pathways associated with feature targets during T2DM progression, Gene Set Enrichment Analysis (GSEA) was performed on all samples from the training dataset GSE76894. The reference gene set “c2. cp.kegg.v7.4. symbols.gmt” was first acquired from the Molecular Signatures Database (MSigDB, http://software.broadinstitute.org/gsea/msigdb/). Spearman correlation analysis between each biomarker and all other genes across the training samples was carried out using the “psych” package. Subsequently, genes were ranked in descending order according to their correlation coefficients to generate ranked gene lists corresponding to each feature target. GSEA was then implemented via the “clusterProfiler” package to uncover potential biological functions and pathway involvement of the identified targets, with statistical significance defined as *P* < 0.05.

#### 2.1.13 Molecular docking

Molecular docking analysis was conducted to explore the interactions between bioactive compounds and the identified feature targets. Three-dimensional structures of small molecule ligands were obtained from the PubChem database (https://pubchem.ncbi.nlm.nih.gov). For protein receptors, high-resolution crystallographic structures were retrieved from the Protein Data Bank (PDB, http://www.rcsb.org/) based on appropriate selection criteria. Prior to docking, water molecules and phosphate groups were eliminated from protein structures using PyMOL software, and the processed files were exported in PDB format. Docking simulations were executed with AutoDock Vina 1.5.6, and binding free energies were documented. A binding energy threshold of ≤ −5.0 kcal/mol was adopted as indicative of favorable binding affinity, with more negative values representing stronger interactions. This approach enabled preliminary verification of ligand-target interactions, and the top four docking conformations with optimal binding profiles were subsequently visualized in PyMOL (Eberhardt et al., 2021).

### 2.2 *In Vitro* experimental validation

#### 2.2.1 Cells and reagents

INS-1 cells and their specific culture medium were acquired from Shanghai Subcone Biotechnology Co., Ltd., while PBS, 0.25% trypsin, and D-glucose solution were provided by Wuhan Pricella Biotechnology Co., Ltd. The PA-BSA high-fat cell supplement was sourced from Xi’an Kunchuang Technology Development Co., Ltd. For cellular assays, the CCK-8 kit and ROS detection kit were supplied by Shanghai Beyotime Biotechnology Co., Ltd., which also provided the MitoTracker Deep Red fluorescent probe. The JC-1 mitochondrial membrane potential detection kit was obtained from Beijing Solarbio Science & Technology Co., Ltd., and the Seahorse XF Cell Mito Stress Test Kit was purchased from Guangzhou Huanghe Instrument Technology Co., Ltd. Antibodies against BCAT2, CASP8, EPHX2, UCP2, AMPK, p-AMPK, SIRT1, and PGC-1α were all procured from Abcam. Compound C was purchased from MCE.

#### 2.2.2 Drug preparation

Zhimu (Anemarrhena asphodeloides Bge., dried rhizome, Batch No. 240902851) and Huangbai (Phellodendron chinense Schneid., dried bark, Batch No. 241004141) were purchased from Guangdong Kangmei Pharmaceutical Co., Ltd. Both herbal materials were authenticated by Chief Chinese Pharmacist Yuanhui Deng at the Central Laboratory of Guangdong Provincial Hospital of Chinese Medicine, in accordance with the Chinese Pharmacopoeia standards. All pharmacological experiments were performed using a single batch of extract, ensuring internal consistency and comparability of reported results.

In our previous study, a systematic chemical characterization of the aqueous decoction prepared from the same source materials was performed using UPLC-LTQ-Orbitrap XL in both positive and negative ion modes. A total of 55 chemical constituents were identified, including 18 from Zhimu (e.g., timosaponins, mangiferin) and 37 from Huangbai (e.g., berberine, jatrorrhizine, phellodendrine) (Sun et al., 2017). The detailed mass spectrometric data of the identified constituents are presented in Supplementary Table S1.

The ZB lyophilized powder was prepared in-house as follows: 100 g of Anemarrhena asphodeloides (Zhimu) and 100 g of Phellodendron chinense (Huangbai) were soaked in 1600 mL purified water for 30 min, followed by reflux heating for 1 h. The filtrate was gathered, and the extraction procedure was carried out again. The combined filtrates were concentrated via rotary evaporation, pre-cooled at −80 °C for 24 h, and subsequently lyophilized for 24 h. The lyophilized powder yields from three independent preparations were 25.981 g, 25.765 g, and 26.346 g (RSD = 1.13%), demonstrating good reproducibility of the extraction process. Prior to use, an appropriate amount was dissolved in phosphate-buffered saline (pH = 7.4) to prepare solutions at different concentrations for subsequent experiments.

#### 2.2.3 Cell culture and model establishment

INS-1 cells were propagated in complete RPMI 1640 medium (supplemented with 10% fetal bovine serum, 1% sodium pyruvate, and 0.05 mM β-mercaptoethanol) at 37 °C in a 5% CO_2_ humidified incubator. Cells approaching confluence were trypsinized and passaged at a 1:3 ratio. For HGHL modeling, cells were seeded into 96-well plates (1 × 10^4^ cells/well) and cultured for 24 h to permit attachment. Subsequently, the medium was exchanged with glucose (33.3 mmol/L) and palmitic acid (200–1000 μmol/L) containing medium for 24 h. Cell viability was quantified by CCK-8 assay, wherein CCK-8 solution was added at a 1:10 ratio and incubated for 2 h at 37 °C in the dark. Absorbance values at 490 nm were detected using a microplate reader. The palmitic acid concentration corresponding to approximately 50% viability (LC50) was selected as the working concentration for establishing the HGHL injury model.

#### 2.2.4 Toxicity assessment of ZB and its effects on HGHL -induced INS-1 cell viability

INS-1 cells at logarithmic growth phase were plated in 96-well plates at 1 × 10^4^ cells per well. Following 24-h adherence, the medium was removed for subsequent experiments.

For cytotoxicity evaluation, cells were incubated with escalating concentrations of ZB herb pair extract (ranging from 12.5 to 1600 μg/mL) in complete medium for 24 h, with six replicates per concentration and untreated cells as controls. For the cytoprotection assay, experimental groups comprised: control, HGHL model, metformin (Met)-treated positive control (1 mmol/L), and multiple ZB extract treatment groups. Cell viability was assessed via CCK-8 assay. The reagent was applied at a 1:10 dilution in culture medium and incubated for 2 h at 37 °C in darkness. Absorbance at 450 nm was recorded on a microplate reader, and viability was computed as: Cell viability (%) = [(A−B)/(C−B)] × 100%, where A = absorbance of treated samples, B = blank absorbance, and C = absorbance of untreated controls.

#### 2.2.5 Experimental grouping and drug administration

Based on the CCK-8 results, no significant cytotoxicity was observed when the ZB concentration was ≤200 μg/mL with a 24-h treatment duration. The EC50 of ZB for cytoprotection against HGHL-induced injury was determined to be 151.4 μg/mL by nonlinear regression analysis (Figure 10D). Accordingly, three concentrations were selected for subsequent experiments: low dose at 50 μg/mL (≈0.3×EC50), medium dose at 100 μg/mL (≈0.7×EC50), and high dose at 200 μg/mL (≈1.3×EC50), to capture the dose–response relationship across sub- and supra-EC50 ranges. Metformin (1 mmol/L) was selected as the positive control drug. This concentration was determined based on a previous study demonstrating that metformin at this dose protects INS-1 cells from high-glucose-induced injury via activation of the AMPK/SIRT1/PGC-1α signaling pathway (Li et al., 2019), and was further validated by our preliminary experimental results (Ou et al., 2025). Six experimental groups were established: control, model, positive drug control (Met, 1 mmol/L), and ZB low, medium, and high dose groups, with six replicate wells per group.

#### 2.2.6 Apoptosis analysis by flow cytometry

INS-1 cells in the exponential growth phase were plated into 6-well plates at 4 × 10^5^ cells/well and incubated for 24 h to allow attachment. Following group allocation as outlined in Section 2.2.5, cells were subjected to the respective treatments for an additional 24 h. Upon completion of treatment, cells were detached using EDTA-free trypsin, rinsed with PBS, and suspended in Binding Buffer. Sequential staining with Annexin V-FITC and propidium iodide (PI) was performed, followed by a 15-min incubation at room temperature under light-protected conditions. Apoptotic cell populations were quantified via flow cytometry. Early apoptotic cells were identified as Annexin V^+^/PI^−^, whereas late apoptotic cells exhibited an Annexin V^+^/PI^+^ phenotype. The overall apoptosis rate was derived by combining both subpopulations.

#### 2.2.7 Intracellular ROS measurement

INS-1 cells at exponential growth phase were plated into 24-well plates (1 × 10^5^ cells/well) and allowed to adhere for 24 h. Following group assignment as specified in Section 2.2.5, cells received the designated treatments for an additional 24 h. For intracellular ROS detection, the DCFH-DA fluorescent probe was diluted at a ratio of 1:1000 in serum-free medium and applied to the cells. Plates were incubated at 37 °C for 20 min, with gentle agitation performed at 3–5 min intervals to facilitate optimal probe-cell interaction. Thereafter, cells underwent three sequential washes with serum-free medium to eliminate residual extracellular probe. Intracellular ROS accumulation was visualized and captured using fluorescence microscopy.

#### 2.2.8 Mitochondrial membrane potential assessment

INS-1 cells at exponential growth phase were plated into 24-well plates (1 × 10^5^ cells/well) and allowed to adhere for 24 h. Following group allocation and intervention as outlined in Section 2.2.5 for 24 h, mitochondrial membrane potential was evaluated using the JC-1 fluorescent probe. The JC-1 working solution was freshly prepared per the manufacturer’s protocol and applied to the culture medium. After a 30-min incubation at 37 °C, the supernatant was removed and cells were rinsed twice with JC-1 staining buffer (1×). Fresh culture medium (2 mL) was then supplemented, and fluorescence signals were captured using a laser confocal microscope.

#### 2.2.9 Visualization of mitochondrial morphology using mito tracker Deep Red

The MitoTracker Deep Red stock solution was reconstituted and subsequently diluted to prepare the working concentration, with all procedures conducted under light-protected conditions. INS-1 cells at exponential growth phase were plated into 24-well plates (1 × 10^5^ cells/well) and allowed to attach for 24 h. Following group assignment and corresponding interventions as specified in Section 2.2.5 for 24 h, cells were loaded with MitoTracker Deep Red working solution and maintained at 37 °C for 15–30 min. Upon completion of staining, the probe solution was aspirated and replaced with fresh culture medium. Mitochondrial morphology was subsequently visualized and documented using a laser confocal microscope.

#### 2.2.10 Mitochondrial respiratory function analysis

Mitochondrial respiratory function was assessed using the Agilent Seahorse XFe24 Extracellular Flux Analyzer, with oxygen consumption rate (OCR, pmol·min^−1^) serving as the primary readout. Key respiratory parameters evaluated included basal respiration, maximal respiratory capacity, proton leak, ATP-linked respiration, and non-mitochondrial oxygen consumption. INS-1 cells from each experimental group were plated onto XF24 cell culture microplates and grown until reaching 80%–90% confluence. Subsequently, 250 μL of medium was supplemented, and cells were maintained for an additional 24 h under standard culture conditions. The sensor cartridge was hydrated overnight prior to the assay. On the measurement day, the assay medium was prepared using XF base medium adjusted to pH 7.4. Approximately 200 μL of spent medium was aspirated from each well and replaced with 450 μL of assay medium to achieve a final volume of 500 μL. Cells were then transferred to a CO_2_-free incubator at 37 °C and equilibrated for 60 min before initiating the measurement. The mitochondrial modulators oligomycin, carbonyl cyanide-4-(trifluoromethoxy) phenylhydrazone (FCCP), and rotenone/antimycin A (Rot/AA) were sequentially injected at final concentrations of 1.5, 0.5, and 0.5 μmol L^−1^, respectively.

#### 2.2.11 Western blot analysis

Cells were seeded in 6-well plates (4 × 10^5^ cells/well, 2 mL) and collected after treatment. Total protein was extracted with RIPA buffer (30 min on ice), followed by centrifugation (12,000 rpm, 20 min, 4 °C) to obtain the supernatant. Samples were mixed with 5× loading buffer and heat-denatured at 100 °C for 10 min. Equal protein quantities were separated by SDS-PAGE using 15-well precast gels and electrotransferred to PVDF membranes. Membranes were blocked for 30 min, then incubated overnight at 4 °C with primary antibodies against BCAT2, CASP8, EPHX2, UCP2, AMPK, p-AMPK, SIRT1, PGC-1α (all at 1:1000 dilution), and GAPDH (1:5000). Following five TBST washes (6 min each), HRP-conjugated secondary antibodies (1:5000) were applied for 1 h at room temperature. After additional washing, bands were visualized by ECL and captured using a chemiluminescence imaging system. Band intensities were quantified with ImageJ. To further validate the causal role of the AMPK signaling pathway in mediating ZB’s therapeutic effects, an additional AMPK inhibition experiment was conducted. Cells were pre-treated with the selective AMPK inhibitor Compound C for 1 h prior to HGHL induction and drug administration. The following groups were established: control, model, ZB high dose (200 μg/mL + HGHL), Compound C (10 μM + HGHL), Compound C + ZB high dose (10 μM + 200 μg/mL + HGHL), and Compound C + Met (10 μM+1 mmol/L + HGHL).

#### 2.2.12 Statistical analysis

All experimental data are presented as mean ± standard deviation (SD). Statistical comparisons among multiple groups were performed using one-way analysis of variance (ANOVA) followed by Tukey’s *post hoc* test in GraphPad Prism 10.1.2. Differences were considered statistically significant at *P* < 0.05.

## 3 Results

### 3.1 Construction of ZB target network

**FIGURE 2:**
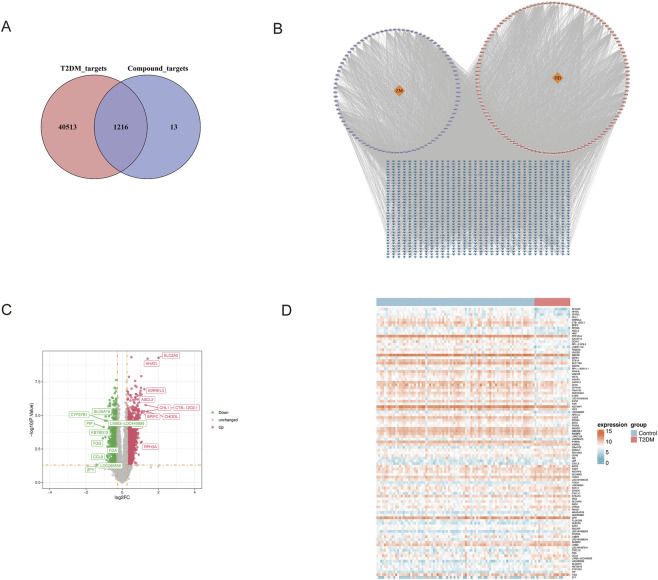
Target network construction and differential gene expression analysis. **(A)** Venn diagram showing the intersection of Zhimu and Huangbai targets with T2DM targets. **(B)** Traditional Chinese medicine-active component-target gene network, where orange represents herbs, purple represents Zhimu active components, pink represents Huangbai active components, and blue represents target genes. **(C)** Volcano plot of differentially expressed genes. **(D)** Heatmap of the top 50 upregulated and top 50 downregulated genes.

A total of 89 Zhimu components and 121 Huangbai components were retrieved from the TCMSP and HERB databases, yielding 205 components after merging and removing duplicates. A total of 916 Zhimu targets and 968 Huangbai targets were obtained, resulting in 1229 targets after merging and deduplication. The intersection of 1229 ZB targets with 41,730 T2DM targets was taken as potential therapeutic targets, yielding 1216 common target genes (Figure 2A). Subsequently, a traditional Chinese medicine-active component-target gene network was constructed using Cytoscape software (Figure 2B).

### 3.2 Identification of DEGs

Differential gene expression analysis was conducted on the GSE76894 training dataset to compare T2DM patients with healthy controls. The limma package was employed for this analysis, applying stringent criteria of *P* < 0.05 and |log2FC| > 0.263. This approach identified 2359 genes exhibiting significant differential expression, of which 1388 were upregulated and 971 were downregulated in the T2DM group (Figures 2C,D).

### 3.3 Identification of disease-related key module genes by WGCNA

**FIGURE 3:**
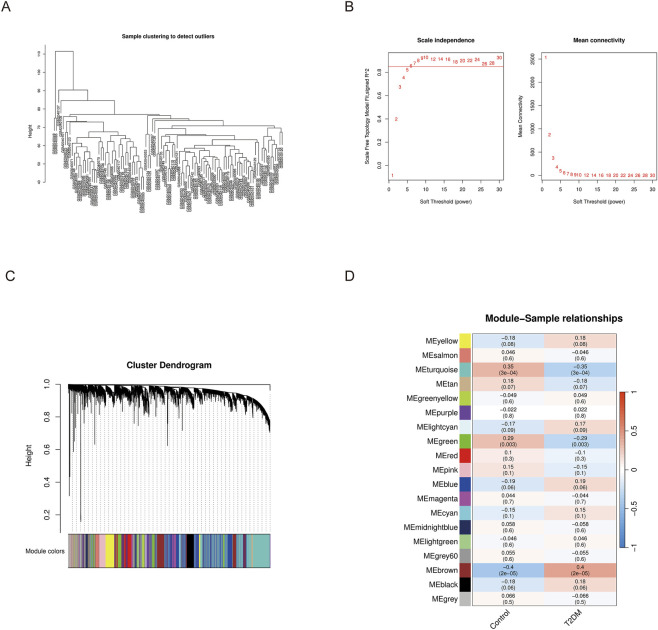
WGCNA analysis for identification of disease-related modules. **(A)** Sample clustering dendrogram. **(B)** Soft-threshold power selection. **(C)** Gene dendrogram and module assignment. **(D)** Module-trait relationship heatmap showing correlations between gene modules and traits.

To systematically dissect the transcriptomic architecture underlying T2DM, we deployed weighted correlation network analysis on the training cohort. Our methodological framework commenced with constructing a hierarchical dendrogram across all specimens, thereby validating dataset homogeneity and inter-specimen concordance (Figure 3A). Selection of an appropriate adjacency function parameter necessitated evaluation of multiple soft-thresholding exponents; empirical testing revealed that β = 7 yielded superior approximation to scale-free network topology (Figure 3B). Leveraging a dynamic hybrid branch-cutting methodology with a lower boundary of 50 transcripts per cluster, we successfully decomposed the entire expression landscape into 19 distinct co-regulatory clusters, while orphan transcripts exhibiting ambiguous clustering patterns were consolidated into the grey compartment (Figure 3C). Our subsequent objective centered on distinguishing modules exhibiting robust phenotypic associations with diabetic pathology. We quantified Pearson correlation coefficients linking each module’s representative eigengene—operationally defined as the first principal component capturing that module’s expression variability—to binary disease classification. Modules demonstrating both correlation strength (absolute magnitude surpassing 0.3) and statistical robustness (P-value inferior to 0.05) were earmarked as pathophysiologically salient. This dual-threshold filtering strategy converged on two cardinal modules: the turquoise assembly, harboring 2825 gene constituents, and the brown assembly, containing 1404 molecular components. The cumulative repertoire of 4229 transcripts derived from these assemblies was subsequently promoted as a high-confidence molecular signature warranting deeper mechanistic dissection (Figure 3D).

### 3.4 PPI network construction and core gene screening

**FIGURE 4:**
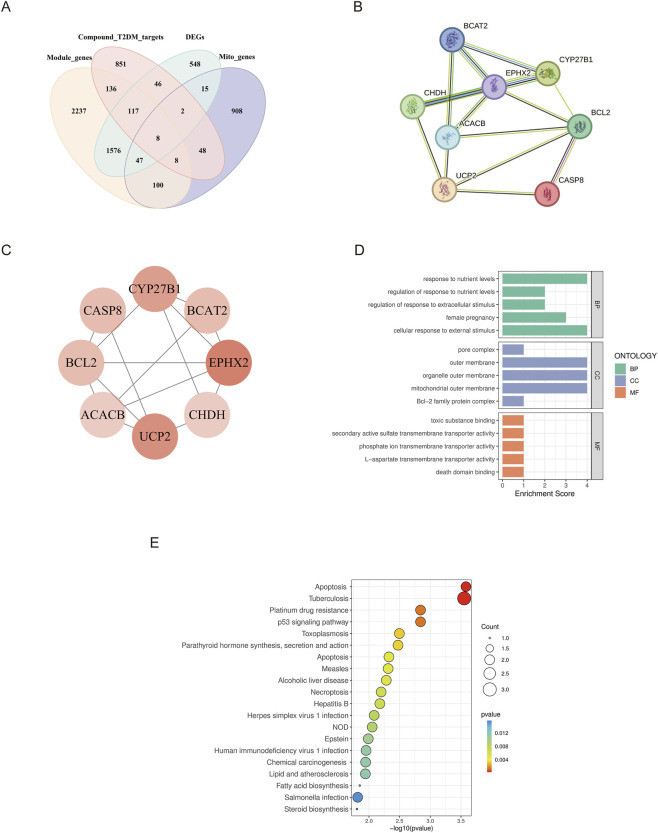
Identification of core targets and functional enrichment analysis. **(A)** Venn diagram of the intersection of DEGs (green), T2DM and active component intersection targets (red), WGCNA key module genes (yellow), and mitochondrial genes (purple). **(B)** PPI network of key targets. **(C)** Protein interaction network visualized by Cytoscape. **(D)** Bar plot of GO enrichment analysis. **(E)** Bubble plot of KEGG enrichment analysis.

A Venn diagram was used to identify the intersection among DEGs, T2DM and active component intersection targets, WGCNA key module genes, and mitochondrial genes, yielding 8 intersecting targets: BCAT2, BCL2, CASP8, CYP27B1, ACACB, CHDH, EPHX2, and UCP2 (Figure 4A). A PPI network was constructed based on these 8 intersecting target genes (Figure 4B). Using degree value as the screening criterion, a protein interaction network was generated using Cytoscape (Figure 4C), where nodes with more edges are colored in deeper red. Notably, although UCP2 was not selected as a feature target in subsequent machine learning models, it was included in subsequent Western blot validation due to its interactions with multiple core targets in the PPI network, preliminary experimental evidence from our research group, and extensive literature reports demonstrating the critical role of UCP2 in mitochondrial function regulation (Jin et al., 2025; Wen et al., 2025).

### 3.5 GO functional and KEGG enrichment analysis

GO and KEGG enrichment analyses were performed on the 8 intersecting target genes, yielding 46 GO terms comprising 19 BP, 9 CC, and 18 MF. For BP, genes were significantly enriched in nutrient metabolism regulation and cellular stress response pathways, particularly response to nutrient levels and cellular response to external stimulus. CC analysis revealed predominant localization to the mitochondrial outer membrane, organelle outer membrane, and Bcl-2 family protein complex. MF enrichment included various transmembrane transporter activities and death domain binding. The top 5 terms for each GO category are shown in Figure 4D. KEGG pathway analysis identified 82 significantly enriched pathways, with the top 20 (Figure 4E) primarily associated with apoptosis (Apoptosis, p53 signaling pathway, Necroptosis), inflammatory response (NOD-like receptor signaling pathway, Tuberculosis, Hepatitis B), and lipid metabolism (Lipid and atherosclerosis, Fatty acid biosynthesis, Steroid biosynthesis). These findings suggest that the target genes regulate T2DM pathogenesis through multiple interconnected biological pathways.

### 3.6 Machine learning screening results

**FIGURE 5:**
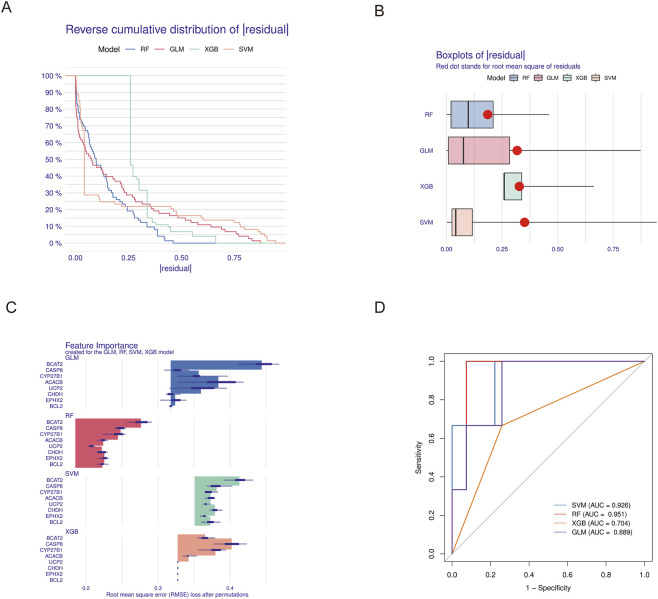
Machine learning model performance evaluation. **(A)** Cumulative residual distribution for each machine learning model. **(B)** Boxplots showing residuals for each machine learning model, with red dots representing root mean square error (RMSE). **(C)** Important features in SVM, RF, GLM, and XGBoost machine learning models. **(D)** ROC analysis of the four machine learning models.

Based on the 8 intersecting targets obtained above, prediction functions were constructed using four machine learning models, and the residual distribution and feature importance among models were visualized. The RF model exhibited relatively lower residuals (Figures 5A–C). Subsequently, ROC curves for the four models were constructed, showing AUC values of 0.926 for the SVM model, 0.951 for the RF model, 0.704 for the XGBoost model, and 0.889 for the GLM model. All AUC values exceeded 0.7, indicating good predictive performance for all four models (Figure 5D), with the RF model achieving the highest AUC value and best predictive performance. Considering both model residuals and ROC curve results, the RF model demonstrated optimal performance in discriminating between disease and control states. Therefore, the top 5 most important variables from the RF model were selected as candidate targets for further validation: BCAT2, CASP8, CYP27B1, ACACB, and EPHX2.

### 3.7 Expression and ROC validation of feature targets

**FIGURE 6:**
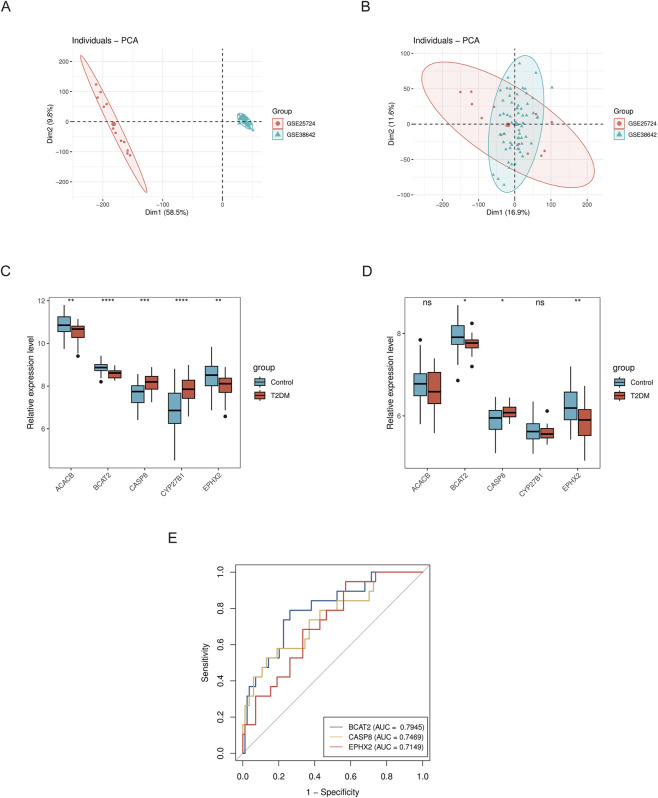
Expression validation and diagnostic performance of feature targets. **(A)** PCA plot showing sample distribution of GSE25724 and GSE38642 datasets before batch effect removal. **(B)** PCA plot showing sample distribution of GSE25724 and GSE38642 datasets after batch effect removal. **(C)** Boxplots of candidate target expression levels in the training set. **(D)** Boxplots of candidate target expression levels in the validation set. **(E)** ROC curves of feature targets in the training set.

To clarify the expression profiles of candidate targets in disease and control samples, GSE76894 was used as the training set, while GSE25724 and GSE38642 were merged after batch effect removal to serve as the validation set (Figures 6A,B). Wilcoxon test was employed to analyze the expression of candidate targets between disease and control groups in both training and validation sets. The results revealed that BCAT2, CASP8, and EPHX2 showed consistent expression trends with statistical significance in both the training and validation sets (Figures 6C,D), these three genes were designated as feature targets. ROC curves for the feature targets in the training set were calculated, revealing AUC values of 0.7945 for BCAT2, 0.7469 for CASP8, and 0.7149 for EPHX2. All three targets exceeded the AUC threshold of 0.7, meeting the diagnostic performance criterion, and thus these three genes were confirmed as feature targets (Figure 6E).

### 3.8 Correlation between immune cells and feature targets

**FIGURE 7:**
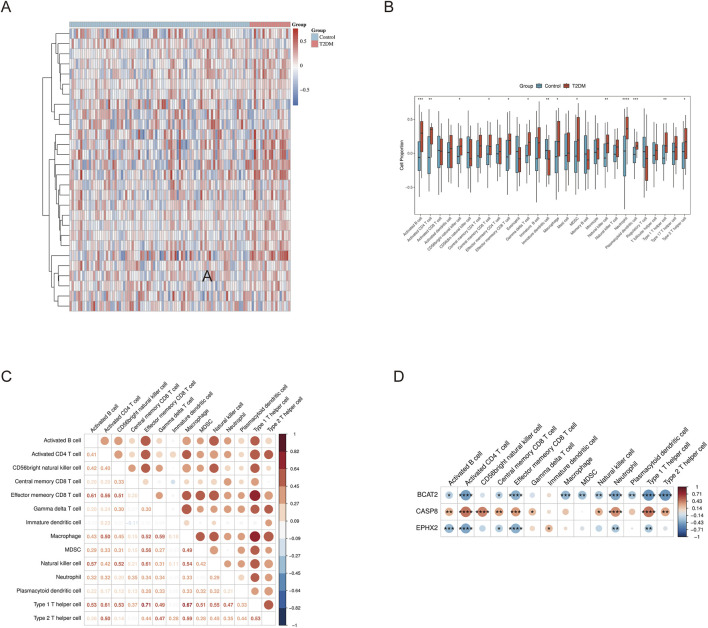
Immune infiltration analysis and correlation with feature targets. **(A)** Heatmap of immune activity for each sample in control and T2DM groups. **(B)** Boxplots showing differences in immune infiltration between T2DM and control groups. **(C)** Heatmap showing correlations among differentially infiltrated immune cells. **(D)** Heatmap showing correlations between feature target expression and differentially infiltrated immune cells.

Given the established contribution of immunological perturbations to diabetic pathogenesis, we sought to characterize the immune cell landscape within our cohorts. Single-sample gene set enrichment analysis (ssGSEA) was deployed to quantify the enrichment scores for distinct immune cell populations across individual specimens (Figure 7A). Comparative profiling between diabetic patients and healthy subjects unveiled heterogeneity in immune cell abundance (Figure 7B), with cellular subsets exhibiting statistically robust inter-group discrepancies (*P* < 0.05) being operationally classified as differentially abundant immune populations. Our analysis converged on fourteen immunological lineages demonstrating significant enrichment disparities across clinical phenotypes. To elucidate potential interrelationships within this immunological milieu, we constructed a Spearman rank-order correlation matrix encompassing all differentially abundant populations (Figure 7C). Subsequently, we interrogated the associations linking our identified molecular signatures to these immunological constituents, with the correlation architecture visualized through hierarchical clustering heatmaps. This integrative analysis revealed that the tripartite biomarker panel exhibited statistically significant associations with six discrete immune subpopulations: B lymphocytes in activated states, CD4^+^ T lymphocytes displaying activation markers, CD8^+^ T cells exhibiting central memory phenotypes, CD8^+^ T cells with effector memory characteristics, neutrophilic granulocytes, and polarized Th1 helper lymphocytes (Figure 7D). Intriguingly, the directional nature of these associations bifurcated along molecular lines: BCAT2 and EPHX2 manifested inverse relationships with the aforementioned immunological activities—implying suppressive regulatory influences—whereas CASP8 demonstrated concordant positive associations, suggesting facilitative immunomodulatory functions. Collectively, these correlation patterns underscore the pivotal immunoregulatory capacities harbored by our feature targets within the T2DM immune microenvironment.

### 3.9 GSEA analysis

**FIGURE 8:**
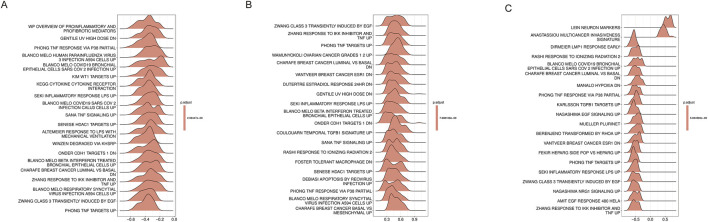
Ridge plots displaying the top 20 enriched pathways from single-gene GSEA analysis. **(A)** BCAT2. **(B)** CASP8. **(C)** EPHX2.

To further elucidate the molecular mechanisms through which the feature targets exert their effects on T2DM, single-gene GSEA enrichment analysis was performed for each key target to identify the associated signaling pathways. The top 20 enriched pathways for each feature target are presented in Figures 8A–C.

### 3.10 Molecular docking

**FIGURE 9:**
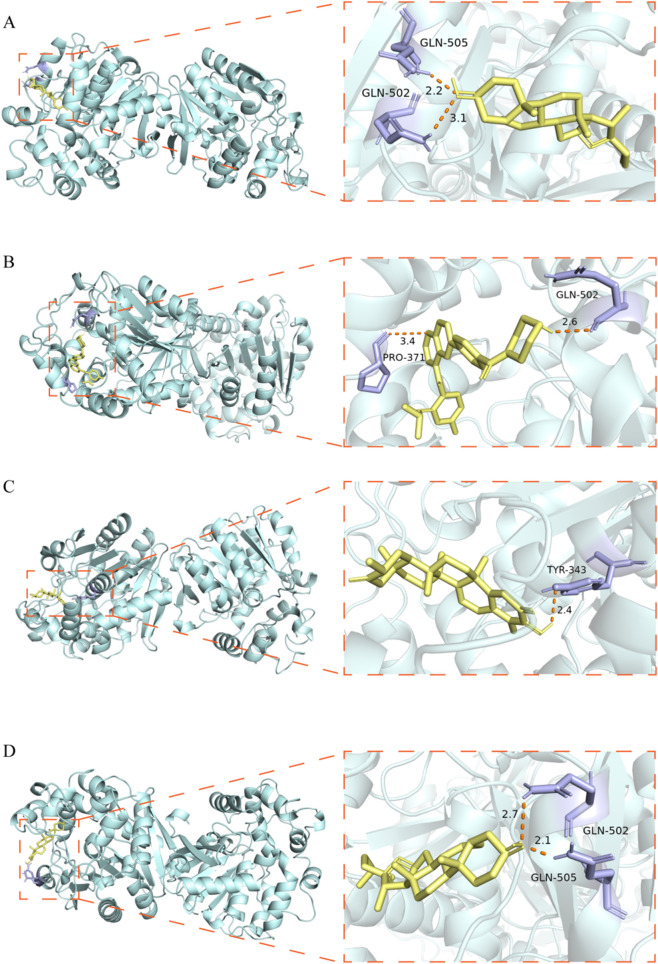
Molecular docking of EPHX2 with active compounds. **(A)** EPHX2-smilagenin. **(B)** EPHX2-Mnk. **(C)** EPHX2-Tingenone. **(D)** EPHX2-Sarsapogenine.

Computational docking analyses assessed the interaction profiles of 3 identified targets against 20 phytochemical components from ZB (Table 1). Binding configurations achieving energy thresholds ≤-5.0 kcal/mol signify robust intermolecular affinity. The results revealed that all target-compound combinations exhibited tight binding except for the CASP8-nicotinamide pair. Subsequently, the four combinations with the lowest binding energies were visualized using PyMOL (Figures 9A–D).

**TABLE 1:** Receptor-ligand binding affinities.

Protein (PDB) Affinity (kcal/mol) Compound	BCAT2 (5mpr)	CASP8 (4jj7)	EPHX2 (3ans)
Anhydroicaritin	−7.1	−7.2	−7.9
Aurantiamide	−8.7	−6.5	−8.5
Auraptene	−7.8	−5.8	−6.6
Beta-sitosterol	−8.0	−7.2	−8.2
Chelerythrine	−7.6	−7.3	−7.9
cyclo (Tyr-Leu)	−6.2	−7.0	−7.2
D-Nicotine	−6.2	−5.0	−6.7
Menisperine	−6.1	−7.1	−7.3
Mnk	−8.8	−7.9	−9.6
Neomangiferin	−8.0	−7.3	−8.4
Nicotinamide	−5.5	−4.5	−5.7
Nicotine	−5.2	−5.0	−6.6
Nomilin	−7.9	−8.3	−9.0
Palmatine	−7.3	−6.3	−7.0
Phellavin	−7.7	−7.7	−8.0
quercetin	−7.3	−7.4	−7.7
Sarsapogenine	−8.9	−8.0	−9.6
Smilagenin	−8.4	−7.5	−9.8
Tigogenin	−8.6	−7.8	−9.4
Tingenone	−8.2	−8.1	−9.6

### 3.11 Determination of HGHL concentrations for INS-1 cell injury induction

**FIGURE 10:**
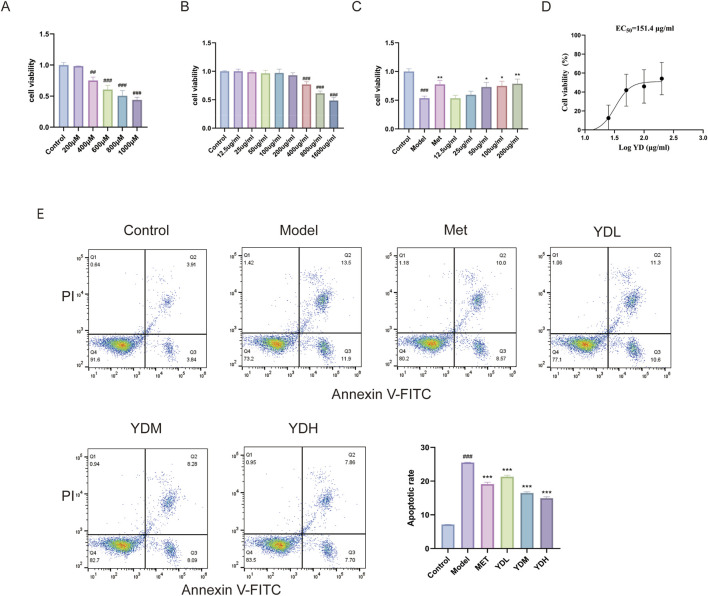
Effects of ZB on INS-1 cells under HGHL conditions. **(A)** Cell viability of INS-1 cells under HGHL. **(B)** Cytotoxicity of ZB at different concentrations on INS-1 cells. **(C)** Protective effects of ZB against HGHL-induced INS-1 cell injury. **(D)** EC50 curve of ZB cytoprotection against HGHL-induced INS-1 cell injury (EC50 = 151.4 μg/mL). **(E)** Effects of ZB on HGHL-induced apoptosis in INS-1 cells. Data are presented as mean ± SD (n = 3). ^###^ *P* < 0.001 vs. control group; ^**^ *P* < 0.01, ^***^ *P* < 0.001 vs. model group.

As shown in Figure 10A, following a 24-h incubation with 33.3 mmol/L glucose and different concentrations of palmitic acid. Exposure to palmitic acid at escalating doses (400, 600, 800, and 1000 μmol/L) elicited progressive reductions in INS-1 cellular survival relative to untreated controls, demonstrating dose-dependent cytotoxicity (*P* < 0.001). Notably, when the palmitic acid concentration reached 600 μmol/L, the cell survival rate was approximately 50%. Therefore, 33.3 mmol/L glucose combined with 600 μmol/L palmitic acid was selected as the optimal concentration for establishing the injury model in subsequent experiments.

### 3.12 Cytotoxicity assessment of the ZB and its effects on cell viability in HGHL-Induced INS-1 cells

After 24-h intervention with different concentrations of ZB, cellular cytotoxicity was evaluated. Initial cytotoxicity screening revealed that ZB extract across a dosage spectrum of 12.5–200 μg/mL exerted negligible impact on INS-1 cellular metabolic activity (Figure 10B), thereby establishing a biocompatibility threshold at or below 200 μg/mL. HGHL insult precipitated substantial compromise of cellular survival relative to vehicle-treated specimens (*P* < 0.001). Notably, ZB supplementation elicited dose-responsive amelioration of this injury phenotype. Modest but statistically robust restoration of viability emerged at intermediate concentrations (50 and 100 μg/mL, *P* < 0.05), while the highest tested dose (200 μg/mL) conferred markedly superior cytoprotection (*P* < 0.01) when benchmarked against untreated injury controls (Figure 10C). Nonlinear regression of the cytoprotection data yielded an EC50 of 151.4 μg/mL for ZB against HGHL-induced injury (Figure 10D). The three experimental concentrations (50, 100, and 200 μg/mL) thus spanned approximately 0.3× to 1.3× EC50, enabling characterization of the dose–response relationship. Collectively, these findings substantiate the reparative capacity of ZB against lipoglucotoxic cellular stress.

### 3.13 Effects of ZB on HGHL-Induced apoptosis in INS-1 cells

Lipoglucotoxic insult markedly elevated the proportion of apoptotic INS-1 cells (*P* < 0.001). Intervention with either the reference compound. Met or graded ZB concentrations orchestrated substantial attenuation of programmed cell death, exhibiting concentration-calibrated rescue efficacy (*P* < 0.001 versus injury-only controls). These observations substantiate that ZB confers cellular protection through suppression of apoptotic pathways in a dosage-responsive fashion (Figure 10E).

### 3.14 Effects of the ZB on intracellular ROS in HGHL-Induced INS-1 cells

**FIGURE 11:**
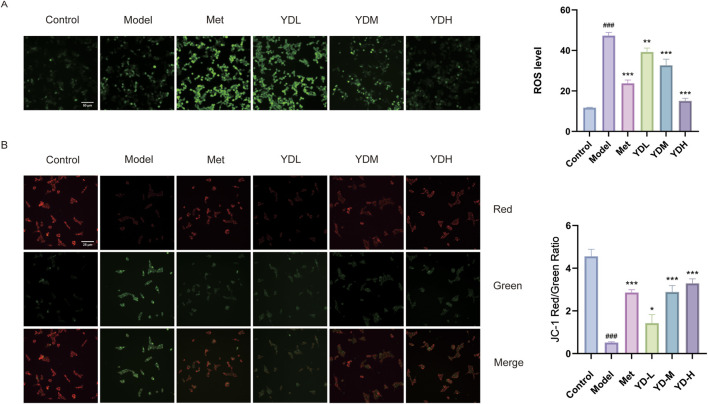
Effects of ZB on ROS levels and mitochondrial membrane potential in HGHL-induced INS-1 cells. **(A)** Effect of ZB on ROS expression levels in INS-1 cells induced by HGHL. **(B)** Effect of ZB on mitochondrial membrane potential in INS-1 cells induced by HGHL. Data are presented as mean ± SD (n = 3). ^###^ *P* < 0.001 vs. control group; ^**^ *P* < 0.01, ^***^ *P* < 0.001 vs. model group.

Cellular oxidative stress burden was quantified employing the DCFH-DA fluorogenic probe, with ROS signals captured via fluorescence microscopy and subjected to densitometric quantification. As depicted in Figure 11A, the model group demonstrated substantial elevation of intracellular oxidant load relative to control specimens (*P* < 0.001). Conversely, intervention with either metformin or tiered ZB formulations (low/medium/high dosages) elicited robust suppression of oxidative stress versus the model group (*P* < 0.01 and *P* < 0.001), with the maximal ZB concentration conferring superior antioxidant efficacy. These findings suggest that ZB protects against HGHL-induced INS-1 cell injury by suppressing intracellular ROS generation.

### 3.15 Effects of ZB on mitochondrial membrane potential in HGHL-Induced INS-1 cells

The JC-1 probe was employed to quantify Mitochondrial membrane potential. JC-1 emits red fluorescence under normal conditions but shifts to green upon membrane depolarization, with the red/green ratio indicating mitochondrial health. As shown in Figure 11B, the model group exhibited a significantly decreased red/green ratio compared with the control group (*P* < 0.001), indicating mitochondrial depolarization. Treatment with the positive control drug and all doses of ZB significantly increased the red/green ratio (*P* < 0.05 and *P* < 0.001) compared with the model group, demonstrating that ZB effectively restores mitochondrial membrane potential in INS-1 cells under HGHL conditions.

### 3.16 Effects of ZB on mitochondrial morphology in HGHL-induced INS-1 cells

**FIGURE 12:**
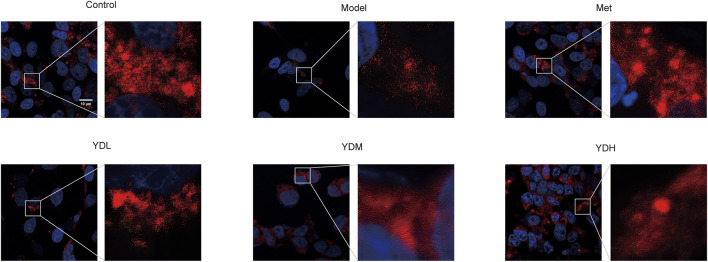
ZB restores mitochondrial morphology in HGHL-induced INS-1 cells.

Mitochondrial morphology and distribution were assessed using MitoTracker Deep Red probe and confocal microscopy. As MitoTracker Deep Red accumulates in mitochondria in a membrane potential–dependent manner, the fluorescence intensity reflects both mitochondrial mass and functional status. Under normal culture conditions, INS-1 cells exhibited abundant and densely distributed mitochondrial fluorescence signals, with mitochondria forming well-organized perinuclear networks indicative of healthy mitochondrial function. In contrast, the model group displayed markedly diminished fluorescence intensity with sparse and diffusely scattered mitochondrial signals, suggesting a substantial reduction in mitochondrial mass and impaired mitochondrial membrane potential following HGHL stimulation. Treatment with metformin and all doses of ZB notably restored mitochondrial fluorescence intensity and distribution density compared with the model group, approaching patterns observed in the control group, with high-dose ZB exhibiting the most pronounced restorative effect (Figure 12). These findings suggest that ZB protects pancreatic β-cells by preserving mitochondrial mass and functional integrity against HGHL-induced mitochondrial damage in INS-1 cells.

### 3.17 Effects of ZB on mitochondrial respiratory function in HGHL-induced INS-1 cells

**FIGURE 13:**
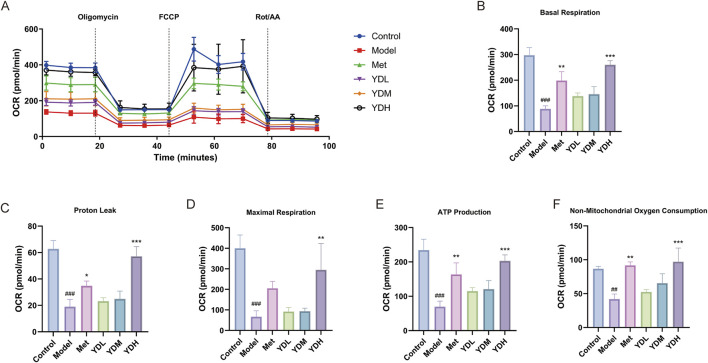
Effects of ZB on mitochondrial respiratory function in HGHL-induced INS-1 cells. **(A)** Real-time OCR profiles of INS-1 cells in each group. **(B)** Basal respiration. **(C)** Proton leak. **(D)** Maximal respiration. **(E)** ATP production. **(F)** Non-mitochondrial oxygen consumption. Data are expressed as mean ± SD (n = 3). ^###^ *P* < 0.001 vs. Control group; ^*^ *P* < 0.05, ^**^ *P* < 0.01, ^***^ *P* < 0.001 vs. Model group.

Mitochondrial OCR serves as a key indicator of mitochondrial respiratory function. Through analysis of OCR profiles in each group (Figure 13A), We conducted comprehensive profiling of five mitochondrial bioenergetic parameters: baseline oxidative phosphorylation, membrane proton conductance, ATP synthesis capacity, extramitochondrial oxygen utilization, and peak respiratory reserves (Figures 13B–F). Relative to controls, the model group demonstrated substantial decrements across all measured indices—encompassing baseline respiration, proton flux, maximal oxygen consumption rate, ATP-linked respiration, and non-mitochondrial oxidative activity (*P* < 0.001)—thereby establishing profound mitochondrial energetic collapse under HGHL stress. Following ZB treatment, low and medium doses demonstrated an improving trend in basal respiration, proton leak, maximal respiratory capacity, ATP production, and non-mitochondrial oxygen consumption, though without reaching statistical significance, whereas high-dose treatment significantly elevated all these mitochondrial respiratory parameters compared to the model group (*P* < 0.001). These results demonstrate that ZB ameliorates HGHL induced mitochondrial dysfunction by enhancing mitochondrial respiratory function and optimizing energy metabolism, thereby improving INS-1 cell survival under HGHL stress conditions and exerting cytoprotective effects.

### 3.18 Effects of ZB on BCAT2, CASP8, EPHX2, UCP2 protein expression and AMPK-SIRT1-PGC-1α signaling pathway in HGHL-induced INS-1 cells

**FIGURE 14:**
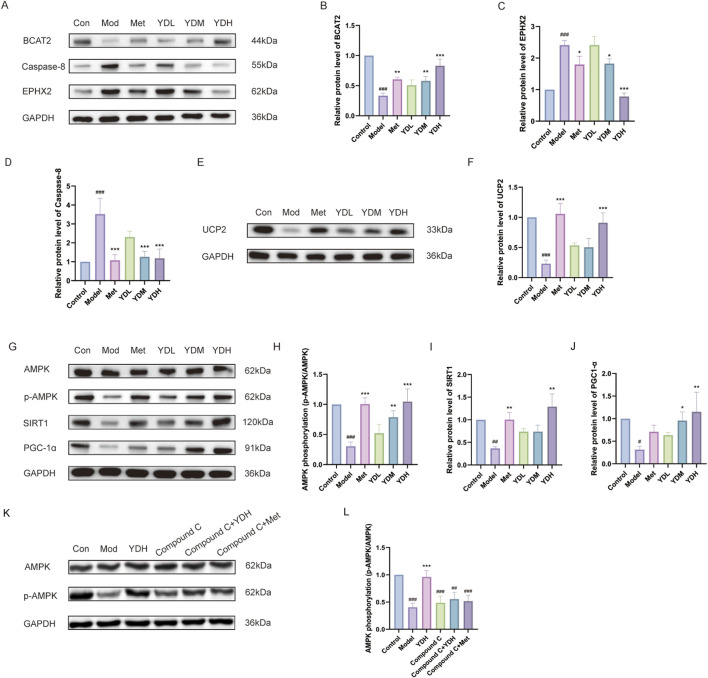
Effects of ZB on key protein expression and AMPK-SIRT1-PGC-1α signaling pathway in HGHL-induced INS-1 cells. **(A)** Representative Western blots of BCAT2, Caspase-8 and EPHX2. **(B–D)** Quantification of BCAT2, EPHX2, and Caspase-8 protein levels. **(E)** Western blots of UCP2. **(F)** Quantification of UCP2 protein levels. **(G)** Western blots of AMPK, p-AMPK, SIRT1 and PGC-1α. **(H–J)** Quantification of p-AMPK/AMPK ratio, SIRT1, and PGC-1α protein levels. **(K)** Representative Western blots of AMPK and p-AMPK following treatment with the AMPK inhibitor Compound C. **(L)** Quantification of p-AMPK/AMPK ratio in the presence of Compound C. Data are presented as mean ± SD (n = 3). ^###^ *P* < 0.001 vs. control group; ^**^ *P* < 0.01, ^***^ *P* < 0.001 vs. model group.

Western blot profiling demonstrated that the model group exhibited depressed UCP2 and BCAT2 coupled with elevated CASP8 and EPHX2 versus controls (all *P* < 0.001). Graded ZB intervention dose-dependently normalized these perturbations: medium/high doses increased BCAT2 (*P* < 0.01–0.001), high dose elevated UCP2 (*P* < 0.001), while both concentrations suppressed CASP8 and EPHX2 (*P* < 0.05–0.001) relative to the model group (Figures 14A–F). Simultaneously, the model group displayed impaired AMPK phosphorylation, SIRT1, and PGC-1α expression (*P* < 0.001–0.05), reflecting pathway inactivation. Medium/high-dose ZB restored p-AMPK/AMPK ratios (*P* < 0.01–0.001) and PGC-1α levels (*P* < 0.05–0.01), with SIRT1 upregulation at the highest dose (*P* < 0.01). The positive control Met, a well-established AMPK activator, exhibited similar regulatory effects, which confirmed the reliability of the detection system (Figures 14G–K).

To confirm the involvement of the AMPK pathway, the inhibitor Compound C was used. As shown in Figures 14K,L, HGHL reduced AMPK phosphorylation (*P* < 0.001), which was reversed by ZB (*P* < 0.001). Importantly, Compound C pretreatment abolished the AMPK-activating effect of ZB (*P* < 0.01), and similar inhibition was observed in the Compound C + Met group (*P* < 0.001). These results indicate that ZB improves HGHL-induced mitochondrial and metabolic dysfunction in INS-1 cells by activating the AMPK signaling pathway.

## 4 Discussion

In TCM, T2DM falls within the category of “Xiaoke”, with its core pathogenesis characterized by spleen deficiency as the root cause and yin deficiency with dryness-heat as the manifestation. The condition is predominantly attributed to excessive consumption of rich and greasy foods, which impairs spleen function in transportation and transformation, thereby engendering dampness-heat accumulation and depleting body fluids—a paradigm encapsulated as “spleen deficiency leading to wasting.” Modern medical research has revealed that the yin-deficient state may correlate with pathological mechanisms such as insulin resistance, enhanced oxidative stress, and chronic low-grade inflammation. Yin-nourishing and heat-clearing medicinals can regulate metabolic homeostasis, improve insulin sensitivity, and thereby modulate blood glucose levels (Peng et al., 2024). Wang Xingxin et al. analyzed 171 prescriptions from 835 publications and identified that yin-nourishing and heat-clearing herbs—such as Shanyao (Dioscorea opposita) and Shengdihuang (Rehmannia glutinosa)—were the most frequently prescribed medications, underscoring the predominant role of the yin-nourishing and heat-clearing therapeutic strategy in the management of Xiaoke disease (Wang and Zhao, 2022). ZB, a classic combination for nourishing yin, clearing heat, and drying dampness, has been widely employed in the treatment of T2DM. Mitochondrial dysfunction constitutes a pivotal pathogenic mechanism in T2DM, and the “spleen–mitochondria correlation” hypothesis serves as a conceptual bridge linking TCM theory with modern mechanistic understanding. This hypothesis posits that the spleen’s function of transportation and transformation is highly congruent with mitochondrial energy metabolism—namely, the spleen’s role in processing and distributing dietary essence corresponds to cellular energy conversion, with mitochondria being the central organelles responsible for this process. Within this framework, the pathogenesis of T2DM can be interpreted as follows: spleen deficiency leads to impaired transportation and transformation, triggering mitochondrial dysfunction; yin deficiency with dryness-heat corresponds to a state of mitochondrial oxidative stress, wherein yin deficiency reflects insufficient energy substrates and pathological heat corresponds to excessive generation of reactive oxygen species (ROS) via the electron transport chain, resulting in oxidative damage. This establishes a vicious cycle of “spleen deficiency → mitochondrial dysfunction → yin deficiency with dryness-heat → oxidative damage → metabolic dysregulation.” The yin-nourishing and heat-clearing therapeutic approach may interrupt this pathological axis by simultaneously targeting energy metabolism and oxidative stress. Nevertheless, whether the ZB herb pair exerts its therapeutic effects in T2DM through modulation of mitochondrial function, as well as the underlying molecular mechanisms, remains to be clarified. Therefore, the present study focuses on mitochondria as the entry point, employing bioinformatics and machine learning to identify mitochondria-related key genes in T2DM and to investigate the potential mechanism of action of the ZB herb pair, thereby providing novel scientific insights into the yin-nourishing and heat-clearing strategy for T2DM treatment.

In this study, bioinformatics and network pharmacology approaches were employed to identify the intersection among differentially expressed genes (DEGs) from GEO datasets (GSE76894, GSE25724, and GSE38642), T2DM–active component overlapping targets, WGCNA-derived module genes, and mitochondrial genes, successfully yielding eight genes with significant differential expression. Gene Ontology (GO) analysis revealed that these DEGs were predominantly involved in multiple biological processes, including response to nutrient levels, regulation of response to nutrient levels, and regulation of response to extracellular stimulus. KEGG pathway analysis indicated that the DEGs were primarily enriched in the apoptosis, p53 signaling, and necroptosis pathways. Apoptosis is a distinctive form of programmed cell death, generally regarded as a self-defense mechanism during immune responses or upon exposure to pathological insults or toxic stimuli. Studies have demonstrated that mitochondrial outer membrane permeabilization (MOMP) is the central event in the intrinsic apoptotic pathway; increased mitochondrial membrane permeability triggers the release of pro-apoptotic factors such as cytochrome c, which activates the caspase cascade and ultimately leads to cell death, thereby impairing insulin secretion and contributing to the onset and progression of T2DM-related complications (Tomita, 2016; Ma et al., 2012). The p53 signaling pathway can be activated by mitochondrial stress signals, including mitochondrial DNA damage and ROS accumulation. Activated p53 signaling promotes inflammatory responses and apoptosis, thereby exacerbating insulin resistance and tissue injury. Necroptosis, a recently identified form of regulated cell death, is mechanistically distinct from apoptosis and represents a unique programmed cell death pathway. Receptor-interacting protein kinases 1 and 3 (RIPK1/3) and the downstream effector protein mixed lineage kinase domain-like protein (MLKL) serve as key signaling molecules in this pathway. Necroptosis may contribute to insulin resistance and the pathogenesis of diabetes and its chronic complications—including retinal, cardiomyocyte, renal, and cerebral injury—through mediating necroinflammation. Inhibiting necroptosis and targeting the necroptotic signaling pathway to block cell death signals may offer novel therapeutic strategies for the prevention and treatment of diabetes and its associated complications (Li et al., 2022).

In recent years, bioinformatics integrated with machine learning has emerged as a powerful tool for target gene analysis (Saelens et al., 2025). In the present study, four machine learning algorithms—SVM-RFE, RF, GLM, and XGB—were employed to screen candidate genes, ultimately identifying BCAT2, CASP8, and EPHX2 as three biomarkers with diagnostic value for T2DM. A diagnostic model was subsequently constructed based on these biomarkers and rigorously validated using an independent validation cohort. The results were consistent with expectations, further confirming the diagnostic potential of these three markers. Receiver operating characteristic (ROC) curve analysis revealed that the area under the curve (AUC) values for BCAT2, CASP8, and EPHX2 reached 0.7945, 0.7469, and 0.7149, respectively, all exceeding the 0.7 threshold, indicating satisfactory diagnostic accuracy for T2DM. Moreover, the validation cohort results demonstrated high concordance with those of the training cohort, further consolidating the diagnostic reliability of these three biomarkers.

Branched-chain amino acid transaminase 2 (BCAT2) is a mitochondria-localized aminotransferase that plays a pivotal role in branched-chain amino acid (BCAA) catabolism. Its core function is intimately linked to amino acid metabolism: BCAT2 catalyzes the transamination of BCAAs to their corresponding α-keto acids, which subsequently enter the tricarboxylic acid (TCA) cycle to supply cellular energy and metabolic intermediates (Wang et al., 2025). Dysregulated BCAA metabolism represents a hallmark feature of T2DM. It has been reported that plasma BCAA concentrations are elevated approximately 1.6-fold in T2DM patients, and the levels of their metabolic intermediates, such as 3-hydroxyisobutyrate, are significantly correlated with insulin resistance (Holeček, 2018). In the present study, BCAT2 was found to be significantly downregulated in T2DM samples, suggesting a deficiency in the BCAA metabolic pathway that may result in the systemic accumulation of BCAAs and their metabolic byproducts (e.g., 3-hydroxyisobutyrate), thereby compromising insulin sensitivity and glucose metabolism. These findings provide a novel molecular mechanistic explanation for the metabolic dysregulation observed in T2DM. Modulating BCAT2 expression or activity may therefore represent a promising therapeutic strategy for T2DM by improving BCAA metabolism and alleviating insulin resistance.

Caspase-8 (CASP8) is a cysteine-aspartic protease that functions as an initiator caspase in the apoptotic cascade. Upon receiving stimulatory signals, caspase-8 is activated and cleaves receptor-interacting serine/threonine-protein kinase 1 (RIPK1), followed by the recruitment of TNF receptor-associated death domain protein (TRADD), which in turn activates executioner caspases to initiate apoptosis (Fritsch et al., 2019). Notably, activation of the RIPK1–caspase-8 cell death complex can trigger multiple forms of cell death, including apoptosis, necroptosis, and pyroptosis. Our results demonstrated that CASP8 was significantly upregulated in T2DM samples, which is consistent with the KEGG pathway enrichment findings. This suggests that CASP8 may play a critical role in the onset and progression of T2DM, potentially through mechanisms involving the activation of inflammatory responses, metabolic dysregulation, immune cell infiltration and activation, and multiple cell death pathways.

Soluble epoxide hydrolase (EPHX2) is a key enzyme in epoxy fatty acid metabolism, primarily catalyzing the hydrolysis of epoxyeicosatrienoic acids (EETs) and other epoxy fatty acids (EPFAs) into their corresponding dihydroxyeicosatrienoic acids (DHETs), thereby modulating intracellular levels of lipid metabolism-related substrates (Guan et al., 2025). EETs exert multiple beneficial effects, including vasodilation, anti-inflammation, anti-apoptosis, and improvement of insulin sensitivity. The present study revealed that EPHX2 was significantly upregulated in T2DM samples, suggesting that excessive degradation of EETs may attenuate their protective effects on insulin sensitization, anti-inflammatory activity, and anti-apoptotic capacity. Notably, EPHX2 plays an important role in the regulation of mitochondrial function. EETs can directly establish a mitochondrial protective barrier by activating mitochondrial large-conductance calcium-activated potassium channels (BKCa), scavenging reactive oxygen species (ROS), and stabilizing mitochondrial membrane potential. Furthermore, inhibition of EPHX2 has been shown to effectively alleviate oxidative stress-induced endoplasmic reticulum stress (ERS), thereby maintaining cellular redox homeostasis through multidimensional pathways (Lin et al., 2025).

Furthermore, GSEA was performed on the key targets. The results demonstrated that BCAT2 was predominantly enriched in the KEGG CYTOKINE CYTOKINE RECEPTOR INTERACTION pathway, CASP8 was mainly enriched in the PHONG TNF RESPONSE VIA P38 PARTIAL pathway, and EPHX2 was primarily enriched in the MANALO HYPOXIA DN pathway. These findings indicate that, although the three biomarkers do not share direct upstream–downstream regulatory relationships, they may collectively participate in the onset and progression of T2DM by converging on core pathological processes, including inflammatory responses, apoptosis, oxidative stress, and mitochondrial dysfunction. Nevertheless, it should be noted that despite the preliminary achievements of the present study in biomarker screening and diagnostic model construction, more in-depth experimental investigations are warranted to elucidate the specific molecular mechanisms through which these biomarkers contribute to T2DM pathogenesis, thereby providing a more robust theoretical foundation and practical guidance for the early prediction and treatment of T2DM.

The core pathophysiology of T2DM encompasses two principal aspects: insulin resistance and pancreatic β-cell secretory dysfunction, both of which involve inflammatory responses, immune dysregulation, and oxidative stress. Previous studies have demonstrated that prolonged chronic inflammation inflicts damage on pancreatic β-cells, subsequently triggering the development of insulin resistance in T2DM (Bao et al., 2025). Emerging evidence has revealed that mitochondria serve not only as the cellular energy powerhouses but also as critical hubs of the immune system. Residing within the cytoplasm, mitochondria possess a relatively independent signaling repertoire, encompassing mitochondrial DNA (mtDNA), proteins, nucleic acids, phospholipids, metabolites, and reactive oxygen species (ROS). These mitochondria-derived signals have been shown to activate diverse immune signaling pathways, playing pivotal roles in immune cell activation, cytokine production, and the orchestration of inflammatory responses (Xu et al., 2024; Marchi et al., 2023; Mills et al., 2017; Yang et al., 2022).

Mitochondrial dysfunction may lead to increased reactive oxygen species (ROS) production and metabolic abnormalities, thereby affecting the activity and function of immune cells. Conversely, signaling molecules such as cytokines secreted by immune cells can in turn provide feedback regulation of mitochondrial function and metabolism. Accordingly, the present study further explored the performance of differentially expressed genes in the context of immune infiltration by comparing the expression profiles of various immune cell types between the disease and control groups. The results revealed that multiple immune cell populations—including activated B cells, activated CD4 T cells, effector memory CD8 T cells, gamma delta T cells, macrophages, natural killer cells, natural killer T cells, type 1 T helper cells, and type 2 T helper cells—exhibited significant differences between the T2DM and control groups. These findings suggest that the aforementioned immune cells may play important roles in the pathogenesis of T2DM, further corroborating that immune dysregulation is a hallmark feature of this disease. Subsequent correlation analysis between the expression of feature targets and differentially infiltrated immune cells demonstrated that all three feature targets were significantly correlated with activated B cells, activated CD4 T cells, CD56-bright natural killer cells, effector memory CD8 T cells, neutrophils, and type 1 T helper cells. Specifically, BCAT2 and EPHX2 were negatively correlated with the activity of these immune cells, whereas CASP8 was positively correlated, suggesting that these targets may participate in the pathological progression of T2DM through the modulation of immune cell function. Based on these observations, it can be inferred that targeting these genes or regulating the activity of the associated immune cells may contribute to restoring immune homeostasis and ameliorating the disease state. Future studies are warranted to further validate the therapeutic potential of these targets, thereby providing a basis for precision medicine approaches in T2DM management.

Molecular docking is a widely used computational approach for predicting the binding affinity between bioactive components of traditional Chinese medicine and their potential therapeutic targets, with lower binding energies indicating a higher likelihood of binding. In the present study, molecular docking results demonstrated that the three key genes exhibited strong binding affinities (≤−5.0 kcal/mol) for their corresponding bioactive compounds derived from ZB, with the exception of the CASP8-nicotinamide combination. Analysis of binding energy data further revealed that BCAT2 exhibited relatively lower binding energies with sarsapogenine and aurantiamide, EPHX2 with smilagenin and neomangiferin, and CASP8 with sarsapogenine and nomilin, suggesting potential direct interactions between these compounds and their respective targets. Neomangiferin and mangiferin are both derived from Anemarrhena asphodeloides (Zhimu) and share structural similarity, differing only in their substituent groups. Mangiferin possesses a broad spectrum of physiological activities, including involvement in critical metabolic pathways such as the tricarboxylic acid cycle, lipid and amino acid metabolism, glycolysis, and energy biosynthesis (Du et al., 2018). Furthermore, studies have demonstrated that mangiferin protects mitochondrial function by preserving mitochondrial hexokinase-II in vascular endothelial cells (Song et al., 2017). Neomangiferin exhibits unique bioactivity in anti-aging and the regulation of autophagy-related signaling pathways, such as the IIS and MAPK pathways. Recent research has focused on its potential role in lifespan extension and healthy aging (Wu et al., 2025). Quercetin, one of the bioactive constituents of Phellodendron chinense (Huangbai), has been shown to play a significant role in modulating inflammation and glucose metabolism, and may exert important therapeutic effects in metabolic diseases (Wang et al., 2024). Additionally, quercetin has been demonstrated to ameliorate diabetic kidney disease by activating the PPARA/PPARG–UCP1 signaling axis, thereby constructing a renoprotective network encompassing “metabolic regulation–mitochondrial repair–antioxidant defense” (Yong et al., 2025). However, the roles of sarsasapogenin, aurantiamide, and other bioactive constituents in regulating mitochondrial function remain largely unexplored. This, on one hand, reflects the inherent complexity of traditional Chinese medicine compound formulations and suggests that the herb pair operates through a “multi-component, multi-target” synergistic mechanism. Indeed, our previous study has confirmed that compatibility with Huangbai significantly promotes the cellular uptake of mangiferin, a key active constituent of Zhimu, in INS-1 cells (Fang et al., 2021). On the other hand, this also highlights the advantages of network pharmacology approaches, which are capable of mining potential novel bioactive molecules from large-scale datasets and generating new hypotheses and research directions for traditional Chinese medicine studies. The strong binding affinities of the aforementioned components with core targets position them as priority candidates for subsequent experimental validation, and their potential functions and underlying mechanisms warrant further in-depth investigation.

To further explore the regulatory effects of ZB on mitochondria in T2DM, the present study employed *in vitro* experiments to investigate the molecular mechanisms by which ZB ameliorates glucolipotoxicity in pancreatic β-cells through targeted modulation of mitochondrial dysfunction. A high glucose (33.3 mmol/L) combined with palmitic acid (600 μmol/L)-induced INS-1 cell injury model was successfully established. Compared with the normal control group, the model group exhibited significantly reduced cell viability, markedly elevated apoptosis rates, and characteristic manifestations of mitochondrial dysfunction, including substantially increased ROS levels, mitochondrial membrane potential depolarization, compromised mitochondrial morphology, and impaired mitochondrial respiratory function. These findings demonstrate that the glucolipotoxic milieu induced by the combined high glucose and high lipid conditions can trigger oxidative stress and mitochondrial dysfunction, supporting the notion that mitochondrial dysfunction represents one of the core mechanisms underlying pancreatic β-cell impairment in T2DM. Experimental results further revealed that ZB intervention significantly enhanced INS-1 cell viability and reduced apoptotic rates, indicating that the ZB herb pair possesses cytoprotective properties. Moreover, ZB intervention not only attenuated intracellular ROS generation, restored mitochondrial membrane potential, and improved mitochondrial morphology, but also demonstrated comprehensive improvements in mitochondrial bioenergetics as assessed by Seahorse metabolic analysis, encompassing basal oxygen consumption, proton leak, ATP production capacity, non-mitochondrial respiration, spare respiratory capacity, and maximal respiratory capacity—collectively confirming the definitive mitochondrial protective efficacy of ZB.

At the mechanistic level, Western blot experiments provided critical evidence for elucidating the molecular mechanisms of ZB. The results showed that, compared with the control group, the model group exhibited significantly decreased BCAT2 protein expression and markedly elevated CASP8 and EPHX2 protein expression levels. Following intervention with the positive control drug or ZB, BCAT2 protein expression was significantly upregulated, while CASP8 and EPHX2 expression levels were substantially downregulated compared with the model group. These regulatory trends were highly consistent with the bioinformatics predictions, which not only experimentally validated the pathological significance of these targets under glucolipotoxic conditions, but also revealed the molecular mechanisms through which ZB exerts its therapeutic effects in T2DM via multi-target regulation—potentially through improving amino acid metabolism, inhibiting apoptotic initiation, ameliorating lipid metabolism, and attenuating oxidative stress in a synergistic manner. These findings further substantiate the validity and reliability of the bioinformatics- and machine learning-based screening strategy employed in this study, while underscoring the scientific value and translational potential of multi-target intervention strategies in the treatment of complex metabolic diseases.

A notable highlight of the present study is that, beyond the three feature targets identified through machine learning, we further investigated UCP2,which ranked second in degree centrality within the PPI network,and the AMPK–SIRT1–PGC-1α signaling pathway it mediates. As a core node in the protein–protein interaction network, UCP2 plays a potentially pivotal regulatory role in mitochondrial function, and its critical importance has been supported by both our previous research (Li, 2019) and prior literature. Although the current machine learning models and immune infiltration analyses primarily focused on feature screening and immune phenotype associations and did not encompass this node, the present study supplemented *in vitro* experimental validation of UCP2 expression changes and the aforementioned signaling pathway, aiming to provide more comprehensive experimental evidence for the mechanistic exploration of ZB and multi-target intervention strategies. UCP2 is a proton carrier protein located on the inner mitochondrial membrane that is widely distributed across multiple tissues. UCP2 can reduce membrane potential through mild uncoupling, suppress ROS bursts, and consequently delay the opening of the mitochondrial permeability transition pore (mPTP), thereby exerting a protective effect against mitochondrial dysfunction (Donadelli et al., 2014). AMPK is a highly conserved protein kinase that regulates a series of intracellular signaling cascades. Upon phosphorylation-mediated activation, AMPK participates in multiple energy metabolic processes and plays a critical role in maintaining cellular homeostasis (Herzig and Shaw, 2018). Sirtuin 1 (SIRT1) is a class III histone deacetylase that is broadly expressed across various tissues, including the liver and skeletal muscle, and is associated with gene regulation and maintenance of cellular function. The transcriptional coactivator PGC-1α regulates mitochondrial energy metabolism and is modulated by its upstream molecules AMPK and SIRT1, indirectly promoting transcription and governing mitochondrial function. During mitochondrial energy metabolism, downregulation of PGC-1α can lead to mitochondrial dysfunction and metabolic dysregulation (Cantó and Auwerx, 2009). As a key downstream effector molecule of this pathway, UCP2 is subject to transcriptional regulation by the upstream signaling cascade: upon exposure to stress signals such as energy deprivation, AMPK is activated and subsequently upregulates SIRT1 activity. Activated SIRT1, in turn, deacetylates and activates PGC-1α. The activated PGC-1α, functioning as a transcriptional coactivator, binds to the promoter region of the UCP2 gene, thereby promoting UCP2 transcriptional expression and ultimately enhancing mitochondrial function and conferring resistance to oxidative stress. The results of the present study demonstrated that, compared with the control group, UCP2 protein expression levels were significantly reduced in INS-1 cells of the model group, accompanied by markedly decreased p-AMPK/AMPK ratios and SIRT1 and PGC-1α protein expression levels, indicating impaired mitochondrial function and disrupted energy metabolism in high glucose- and high lipid-induced INS-1 cells. Compared with the model group, ZB intervention significantly increased the expression levels of proteins in this pathway. This was further corroborated by Compound C pretreatment, which abolished the AMPK-activating effect of ZB, confirming the pathway dependence of its therapeutic action, suggesting that ZB enhances the antioxidant capacity of INS-1 cells, augments mitochondrial function, and improves energy metabolism, thereby achieving therapeutic efficacy in T2DM. Notably, although BCAT2, CASP8, and EPHX2 are not direct upstream or downstream regulators of the AMPK–SIRT1–PGC-1α signaling cascade, they are intricately linked to this pathway through their involvement in mitochondrial homeostasis, metabolic substrate flux, and oxidative stress responses. BCAT2 governs branched-chain amino acid catabolism, a process that fuels mitochondrial oxidative phosphorylation and influences the AMPK/mTOR balance (Zheng et al., 2025). CASP8, as a key executioner of apoptosis, is functionally antagonized by AMPK-mediated survival signaling. EPHX2 is the primary enzyme that metabolizes EETs, and EETs can regulate cellular energy metabolism by activating the AMPK signaling pathway (Wang et al., 2021). Thus, these three targets may engage in indirect crosstalk with the AMPK–SIRT1–PGC-1α axis. Such multi-layered “pathway–target” interactions exemplify the multi-target regulatory advantage of ZB in the treatment of T2DM. In contrast to the single-target intervention characteristic of conventional pharmacotherapy, this herb pair exerts its therapeutic effects through synergistic regulation across multiple hierarchical levels and signaling pathways. Moreover, this integrative regulatory paradigm provides a molecular-level interpretation of TCM concept of “spleen deficiency leading to consumptive thirst” (pi xu zhi xiao), furnishing modern biological evidence for the emerging TCM theory linking spleen function to mitochondrial homeostasis.

However, this study has several limitations. First, the bioinformatics analysis was based on publicly available datasets and has not been validated through expression profiling in independent clinical cohorts or multi-center samples, which may limit the extrapolability and generalizability of the findings. Second, The regulatory effect of ZB on mitochondrial function in T2DM has only been validated in in vitro cellular models. There is a lack of validation in in vivo animal models (e.g., T2DM rats) under pathological conditions, as well as a lack of studies incorporating pharmacokinetic-pharmacodynamic (PK-PD) modeling. Consequently, a comprehensive assessment of the agent’s actual therapeutic efficacy in a pathological milieu cannot be achieved. Third, the *in vitro* validation was performed using the total extract of ZB rather than individual active compounds, as ZB functions clinically as an integrated herb pair characterized by multi-component synergy. Although this design more closely reflects clinical practice, it did not allow for the delineation of the specific contributions of individual constituents. Fourth, although mitochondrial morphology and function were assessed using JC-1 fluorescent probes, MitoTracker Deep Red fluorescent probes, and the Seahorse extracellular flux analyzer, transmission electron microscopy was not employed to examine mitochondrial ultrastructural alterations.

Future studies may advance this work in the following directions: (1) Establishing multi-center gene validation cohorts by collecting samples from T2DM patients across diverse geographic regions and ethnic backgrounds to verify the expression differences of key target genes and their associations with the traditional Chinese medicine pattern of yin deficiency, thereby clarifying their potential as cross-population biomarkers. (2) Constructing T2DM animal models (e.g., high-fat diet combined with streptozotocin (STZ)-induced T2DM rat models) and conducting pharmacokinetic-pharmacodynamic (PK-PD) studies to validate the regulatory effects of ZB on mitochondrial function at both the whole-organism level and the pharmacokinetic level. (3) Developing a standardized HPLC/DAD chemical fingerprint method for the ZB extract and incorporating multi-batch consistency evaluation into the quality control workflow, so as to further ensure the reproducibility of the extract’s chemical profile and strengthen the reliability of pharmacological findings across independent studies. (4) Employing network pharmacology-based prediction or bioactivity-guided fractionation strategies to screen the principal bioactive monomeric constituents of the ZB herb pair and design combinatorial intervention groups, thereby elucidating the functional differences and synergistic mechanisms among the key active components of ZB and defining its material basis and mechanisms of action. (5) Establishing a more comprehensive mitochondrial function evaluation system by incorporating transmission electron microscopy to observe mitochondrial morphological changes, including cristae structural integrity, mitochondrial swelling, autophagosome formation, and organelle interactions, thus providing a novel theoretical foundation and experimental basis for the precise intervention of T2DM with ZB and facilitating the translational application of these findings into clinical practice.

## 5 Conclusion

In the present study, BCAT2, CASP8, and EPHX2 were identified as mitochondria-related feature genes of T2DM through bioinformatics combined with machine learning algorithms, and a diagnostic model was subsequently constructed. *In vitro* experiments confirmed that ZB significantly enhanced cell viability, markedly suppressed apoptosis, reduced intracellular ROS generation, and improved mitochondrial membrane potential, mitochondrial morphology, and respiratory function in high glucose- and high lipid-induced INS-1 cells. The underlying mechanisms were associated with the regulation of BCAT2, CASP8, EPHX2, and UCP2 expression, as well as the activation of the AMPK–SIRT1–PGC-1α signaling pathway. This study elucidates the therapeutic mechanisms of ZB in treating T2DM through ameliorating mitochondrial dysfunction, attenuating oxidative damage, and improving energy metabolism, thereby providing novel experimental evidence for the prevention and treatment of T2DM with traditional Chinese medicine.

## Data Availability

The original contributions presented in the study are included in the article/Supplementary Material, further inquiries can be directed to the corresponding authors.
