# Supplementary figures and images for: Identification of mitochondria-related key genes in type 2 diabetes mellitus and elucidation of the Zhimu-Huangbai herb Pair’s mechanism: an integrated approach of bioinformatics, machine learning, and experimental validation (part 1 of 2)
Source: Front Cell Dev Biol. 2026 Mar 13;14:1763178. doi: 10.3389/fcell.2026.1763178 (PMC13021634; doi:10.3389/fcell.2026.1763178)

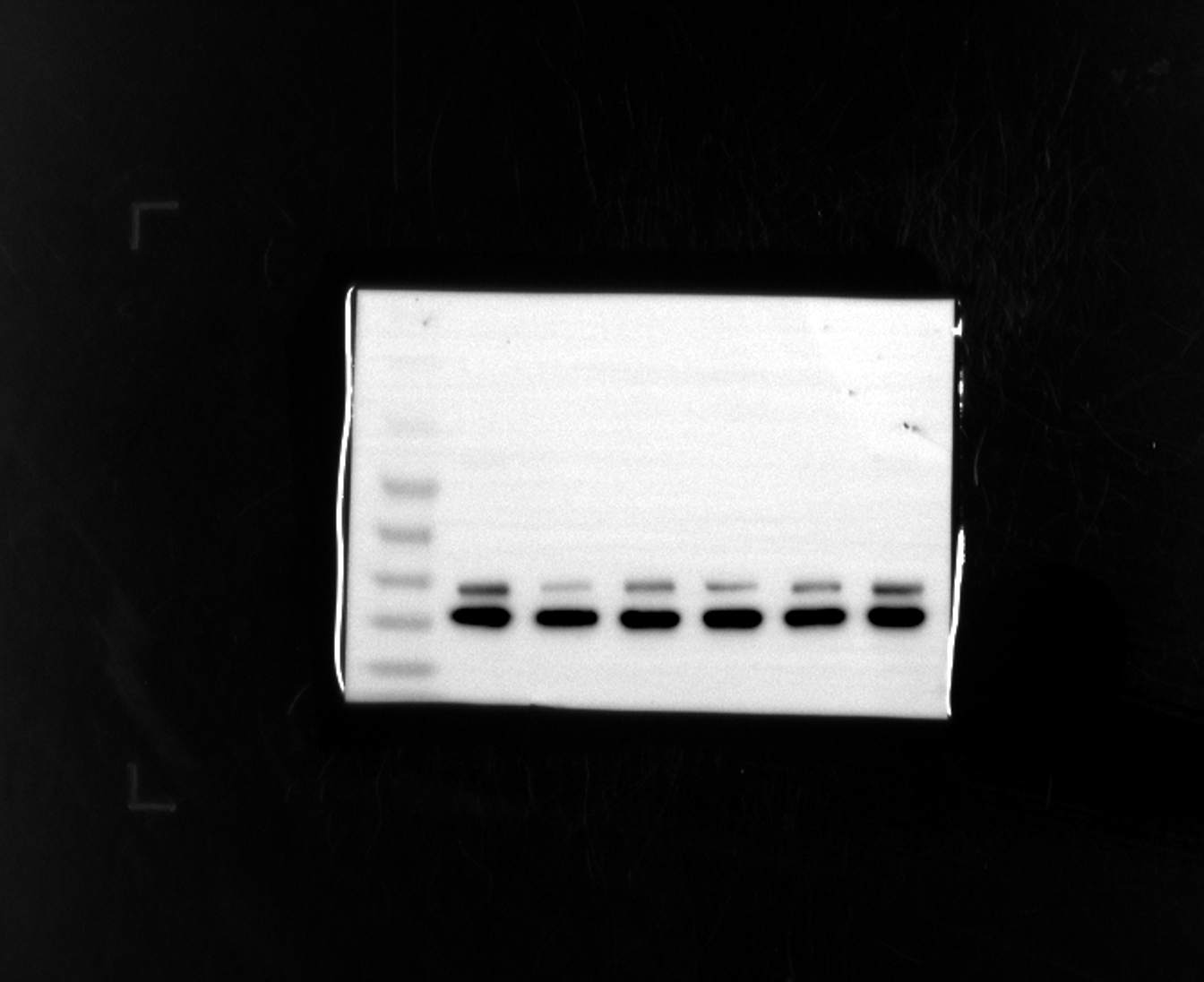

Supplement: Supplementary file 1 [file DataSheet3.zip › 1/BCAT2/BCAT2 1s.Tif]

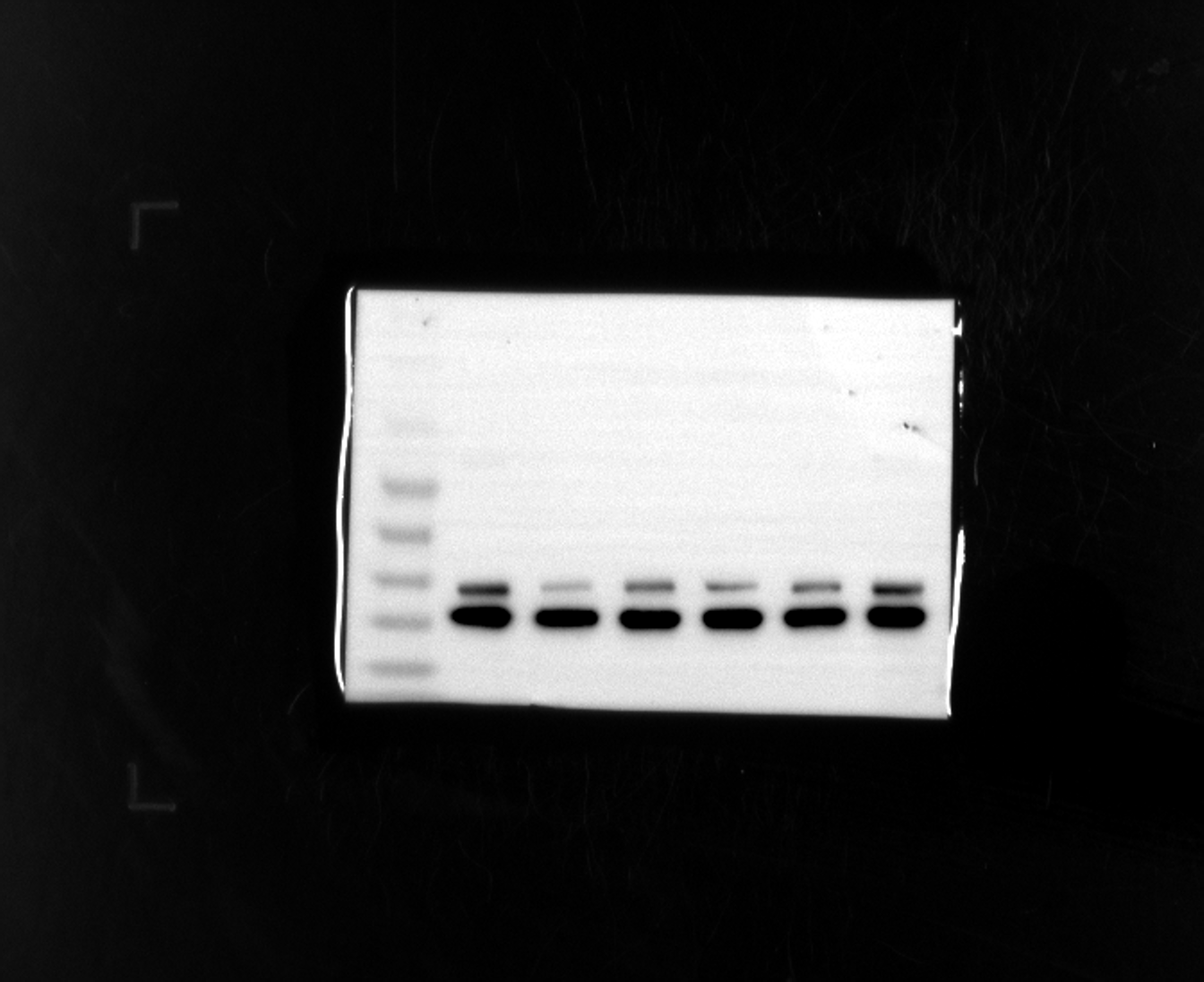

Supplement: Supplementary file 1 [file DataSheet3.zip › 1/BCAT2/BCAT2 3s.Tif]

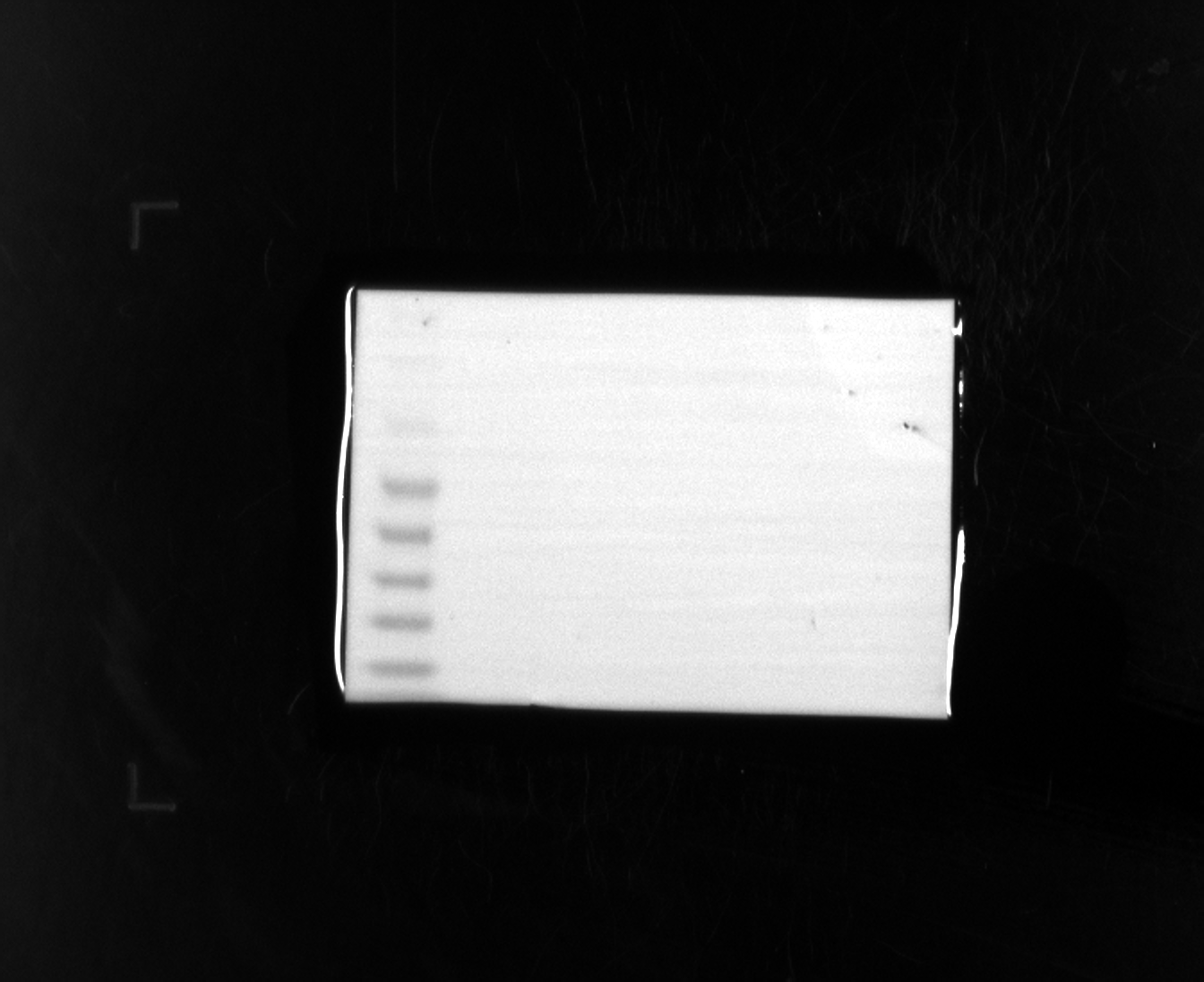

Supplement: Supplementary file 1 [file DataSheet3.zip › 1/BCAT2/marker.Tif]

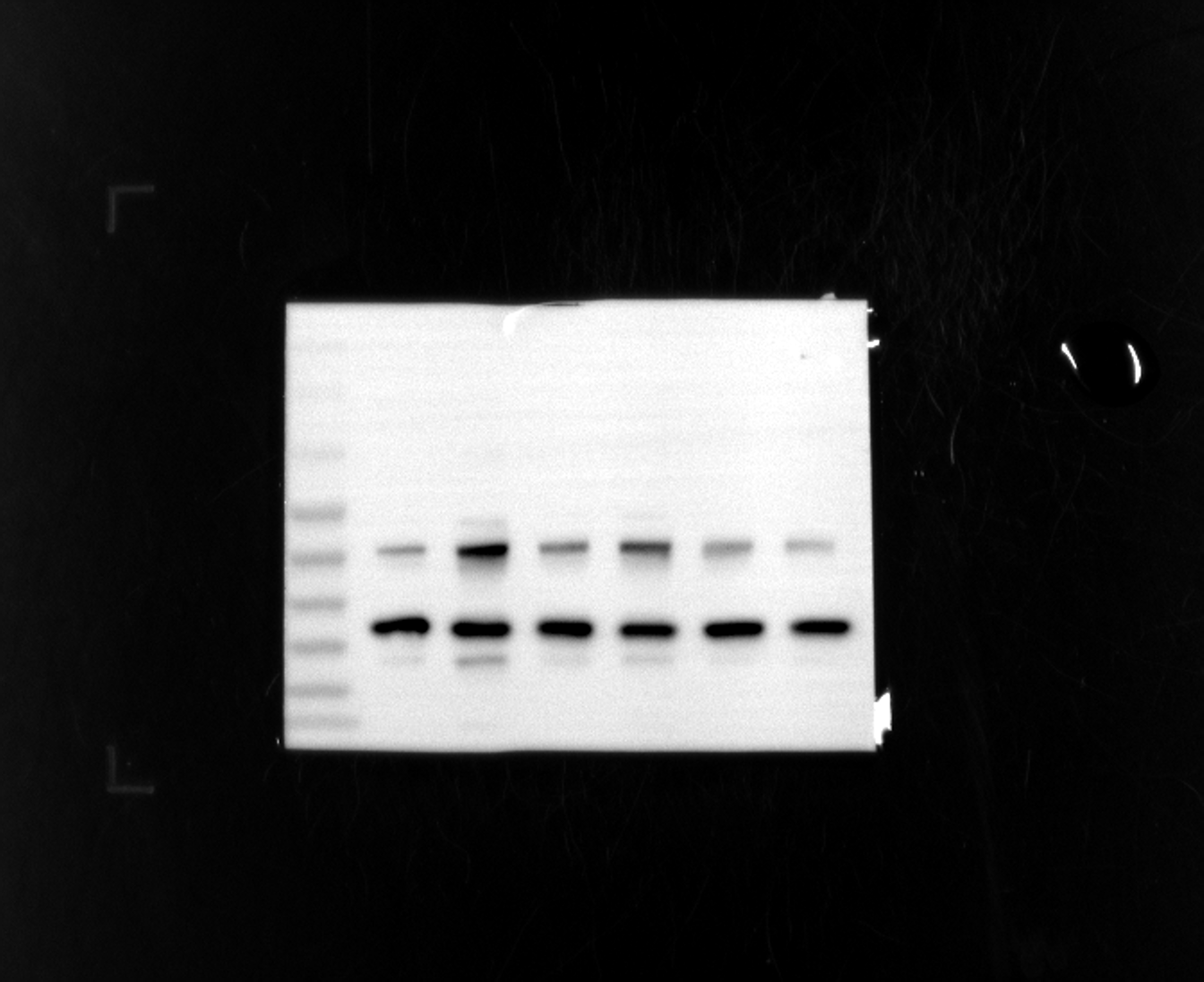

Supplement: Supplementary file 1 [file DataSheet3.zip › 1/Caspase-8/Caspase-8 1S.Tif]

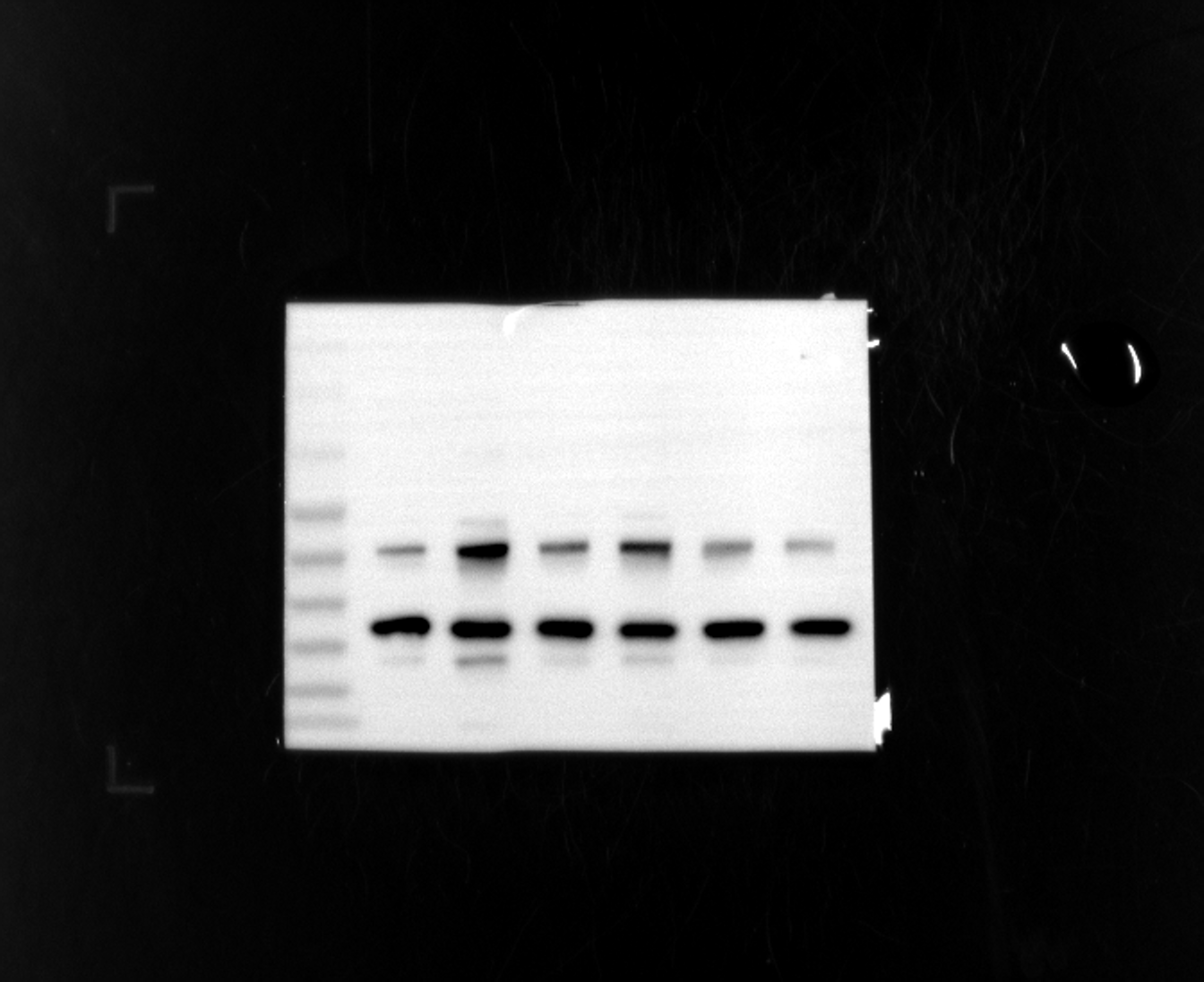

Supplement: Supplementary file 1 [file DataSheet3.zip › 1/Caspase-8/Caspase-8 3S.Tif]

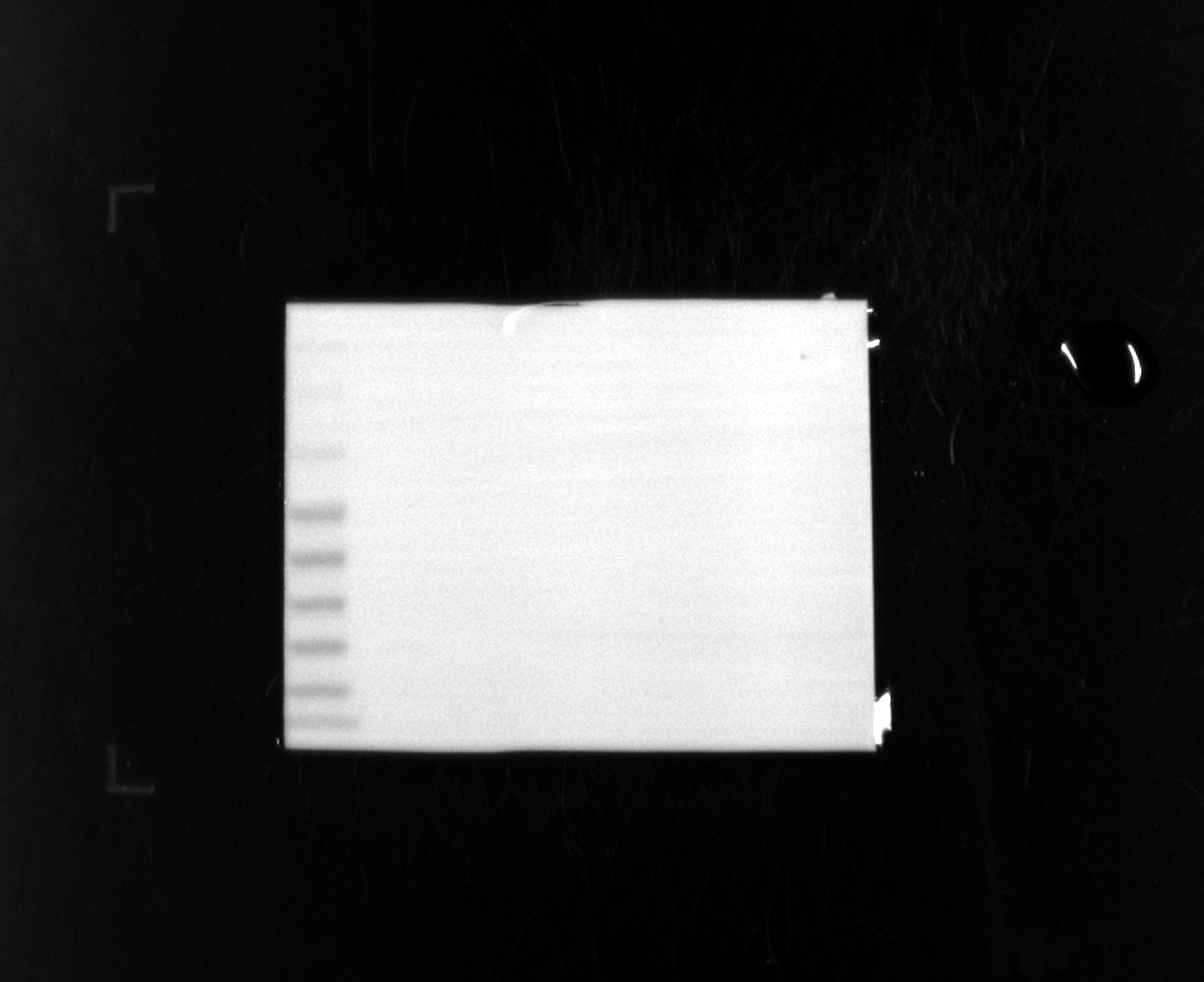

Supplement: Supplementary file 1 [file DataSheet3.zip › 1/Caspase-8/marker.Tif]

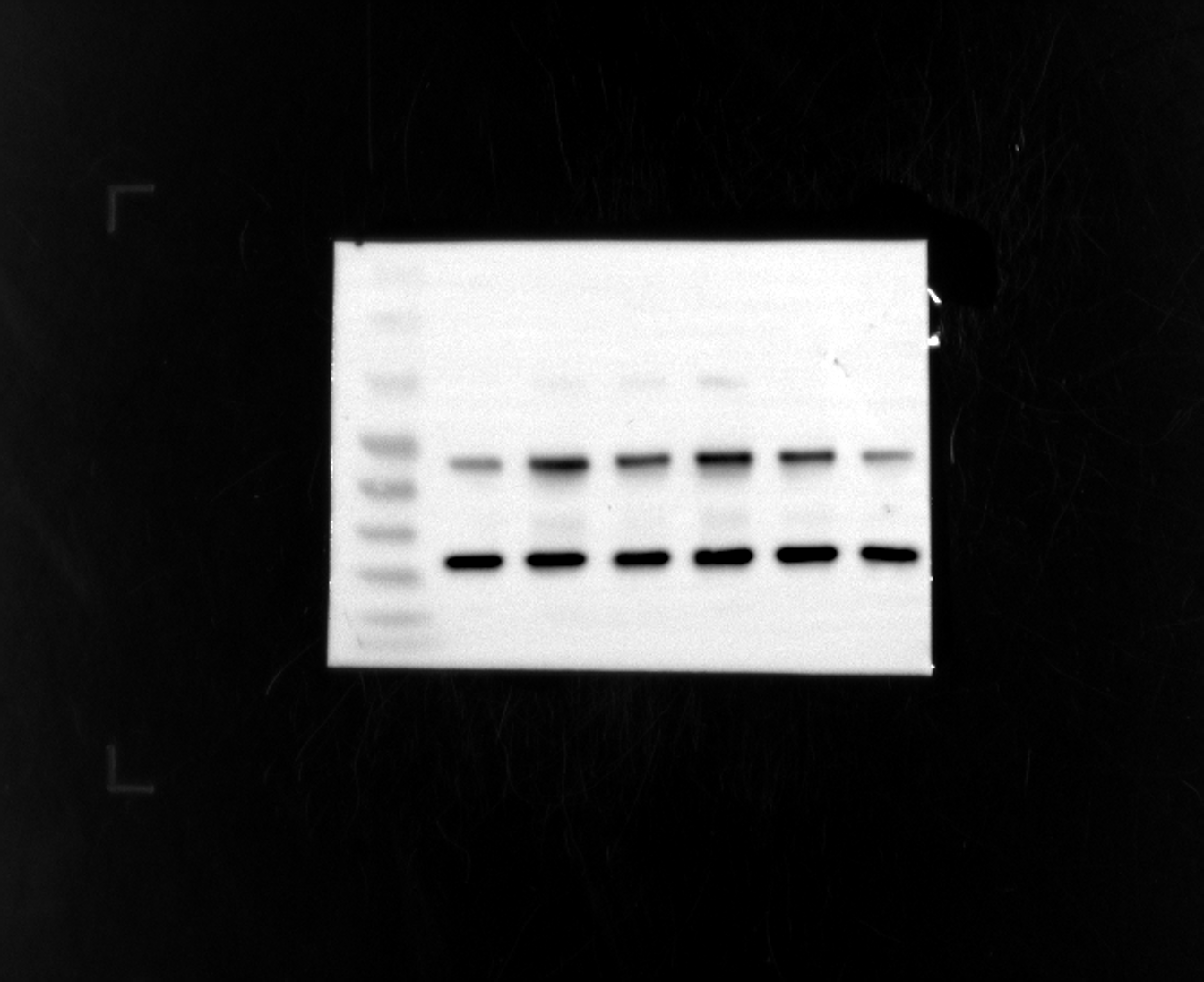

Supplement: Supplementary file 1 [file DataSheet3.zip › 1/EPHX2/EPHX2 1s.Tif]

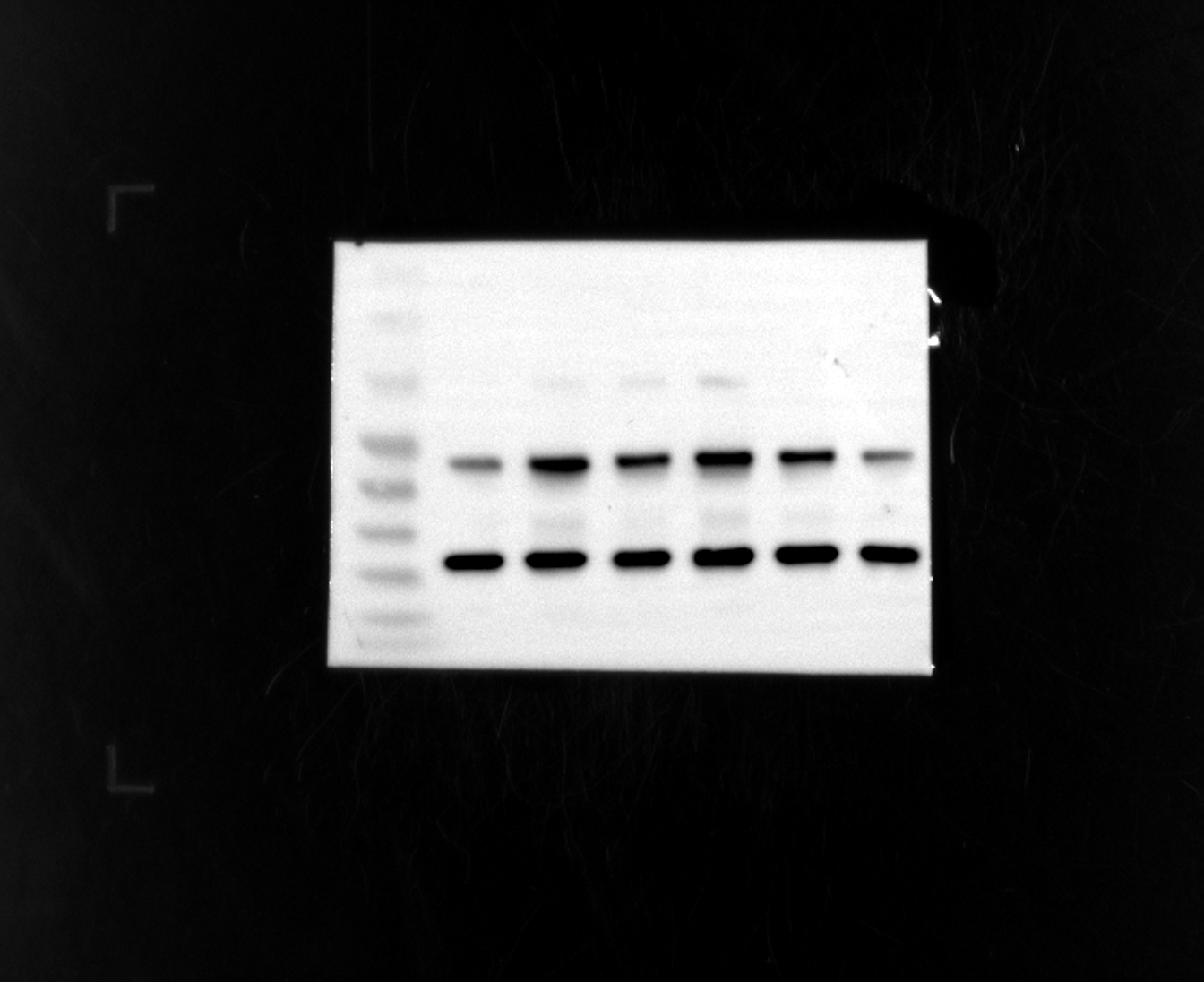

Supplement: Supplementary file 1 [file DataSheet3.zip › 1/EPHX2/EPHX2 3s.Tif]

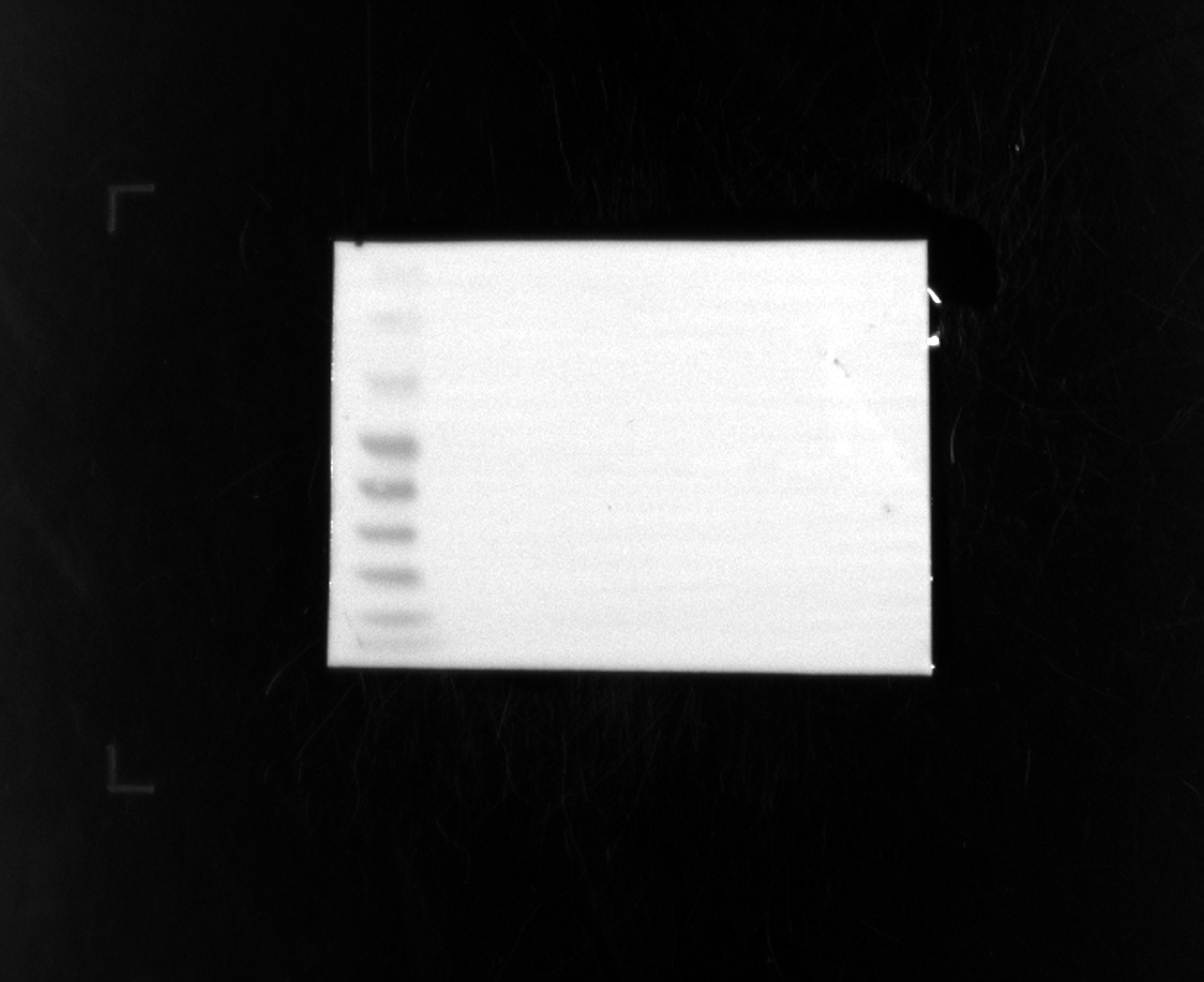

Supplement: Supplementary file 1 [file DataSheet3.zip › 1/EPHX2/marker.Tif]

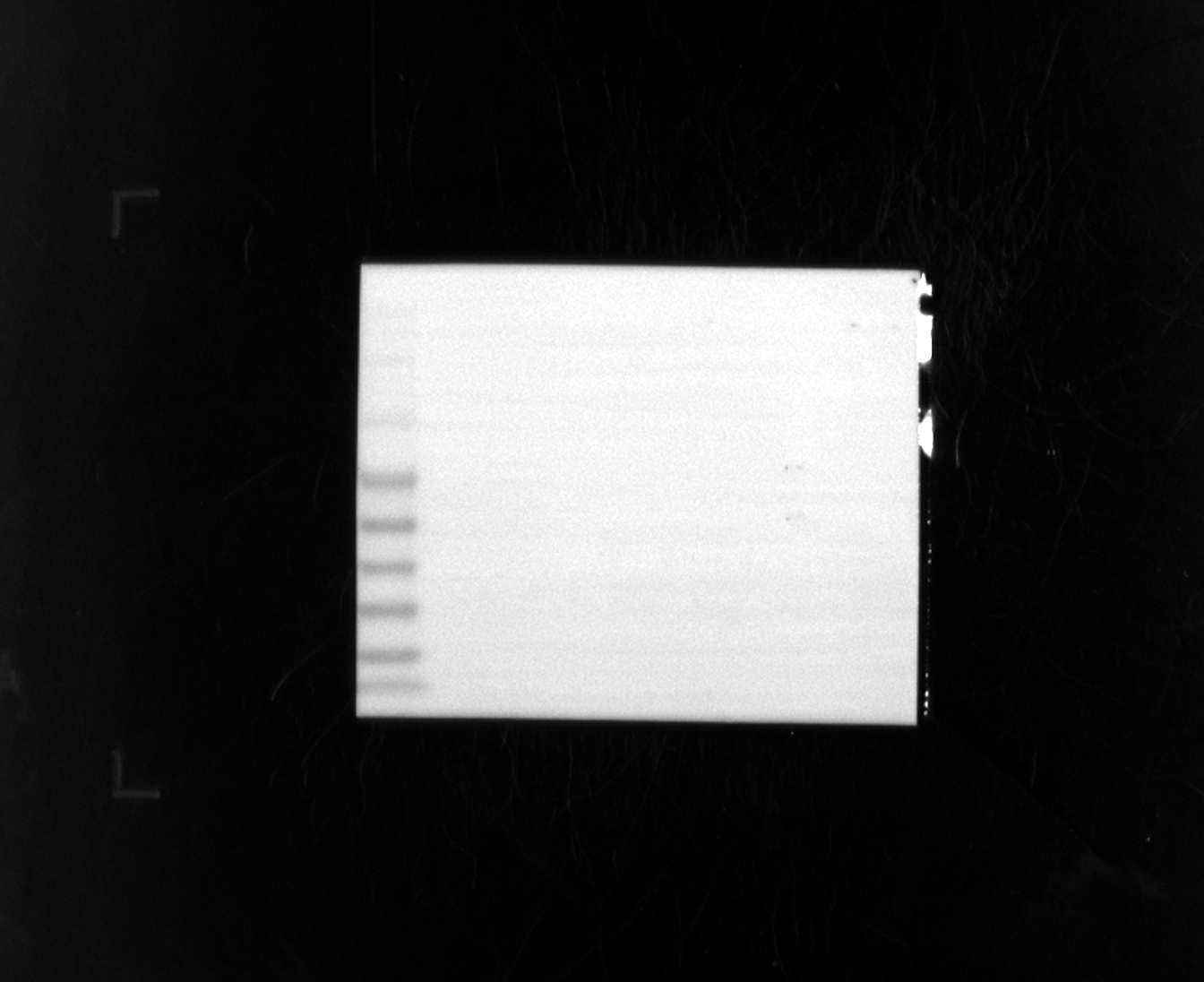

Supplement: Supplementary file 1 [file DataSheet3.zip › 1/UCP2/marker.Tif]

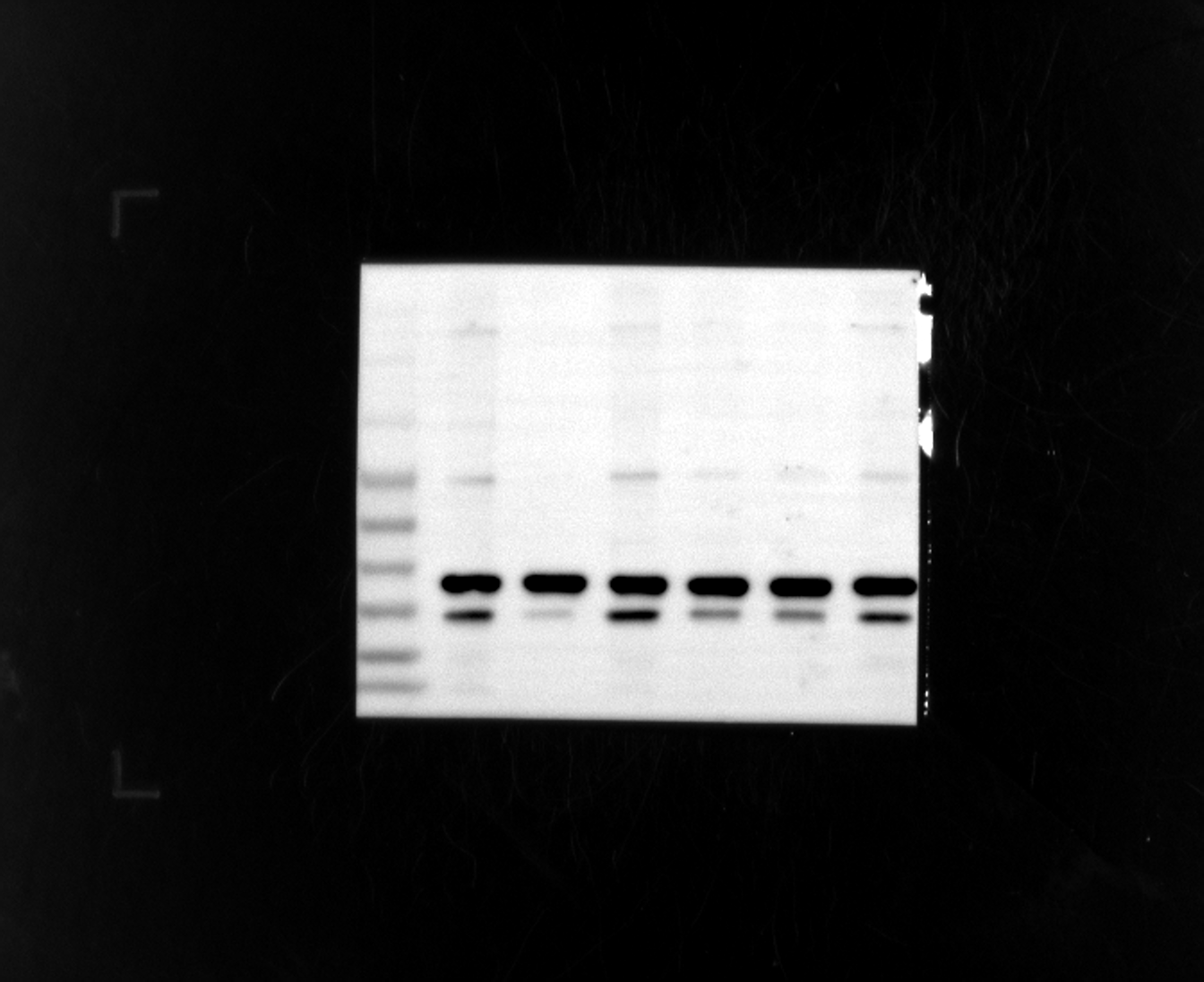

Supplement: Supplementary file 1 [file DataSheet3.zip › 1/UCP2/UCP2 1s.Tif]

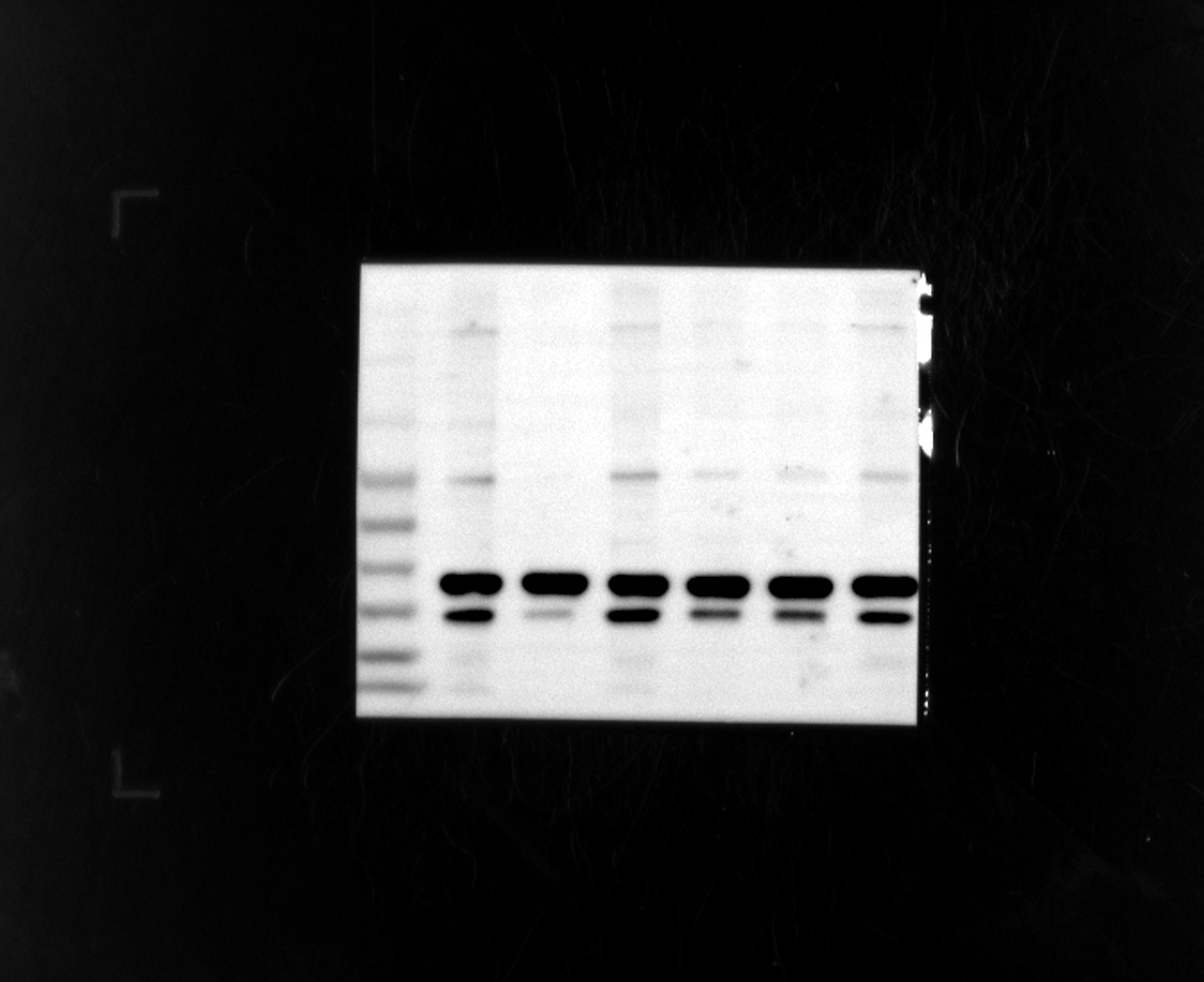

Supplement: Supplementary file 1 [file DataSheet3.zip › 1/UCP2/UCP2 3s.Tif]

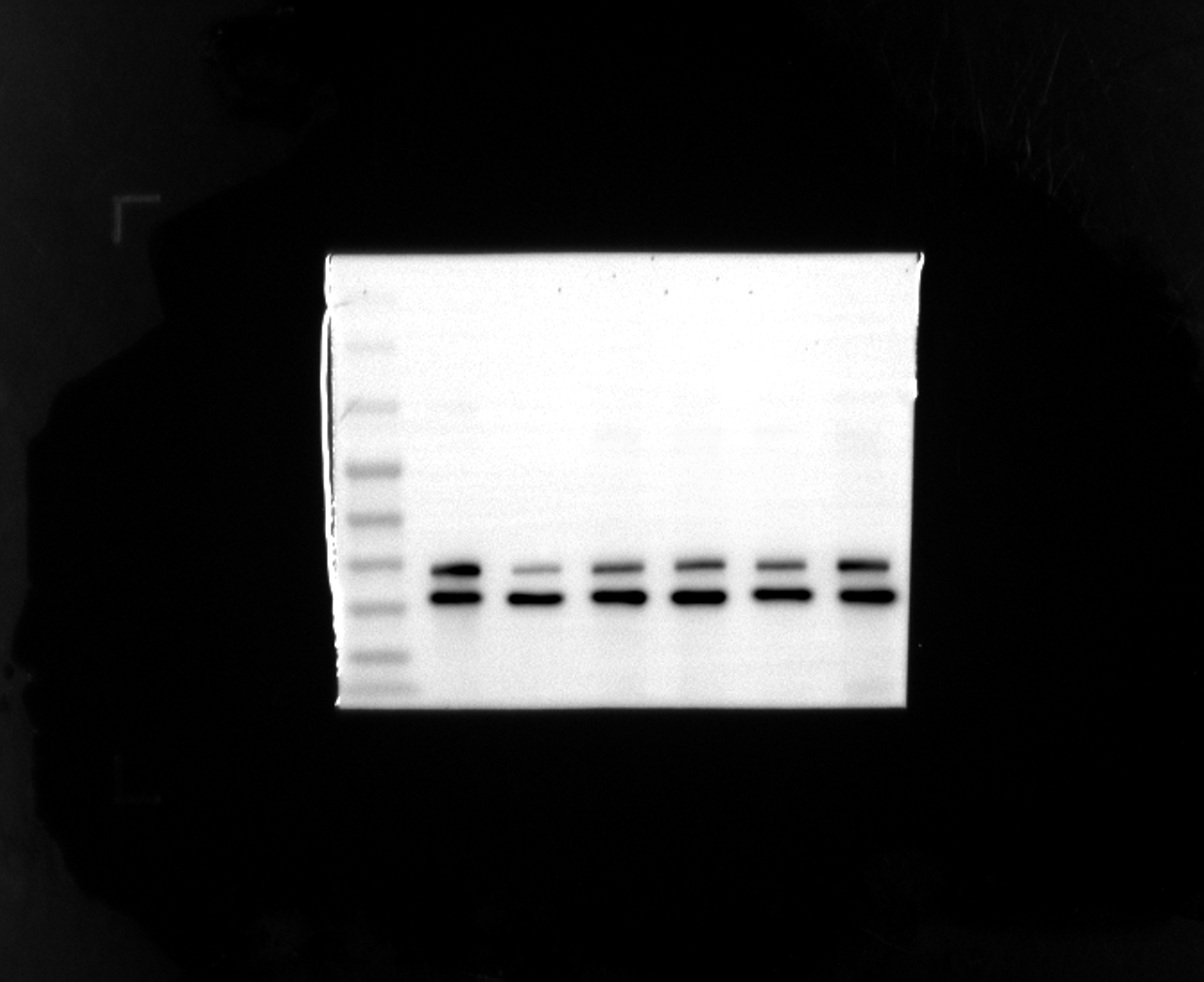

Supplement: Supplementary file 1 [file DataSheet3.zip › 2/BCAT2/BCAT2 1s.Tif]

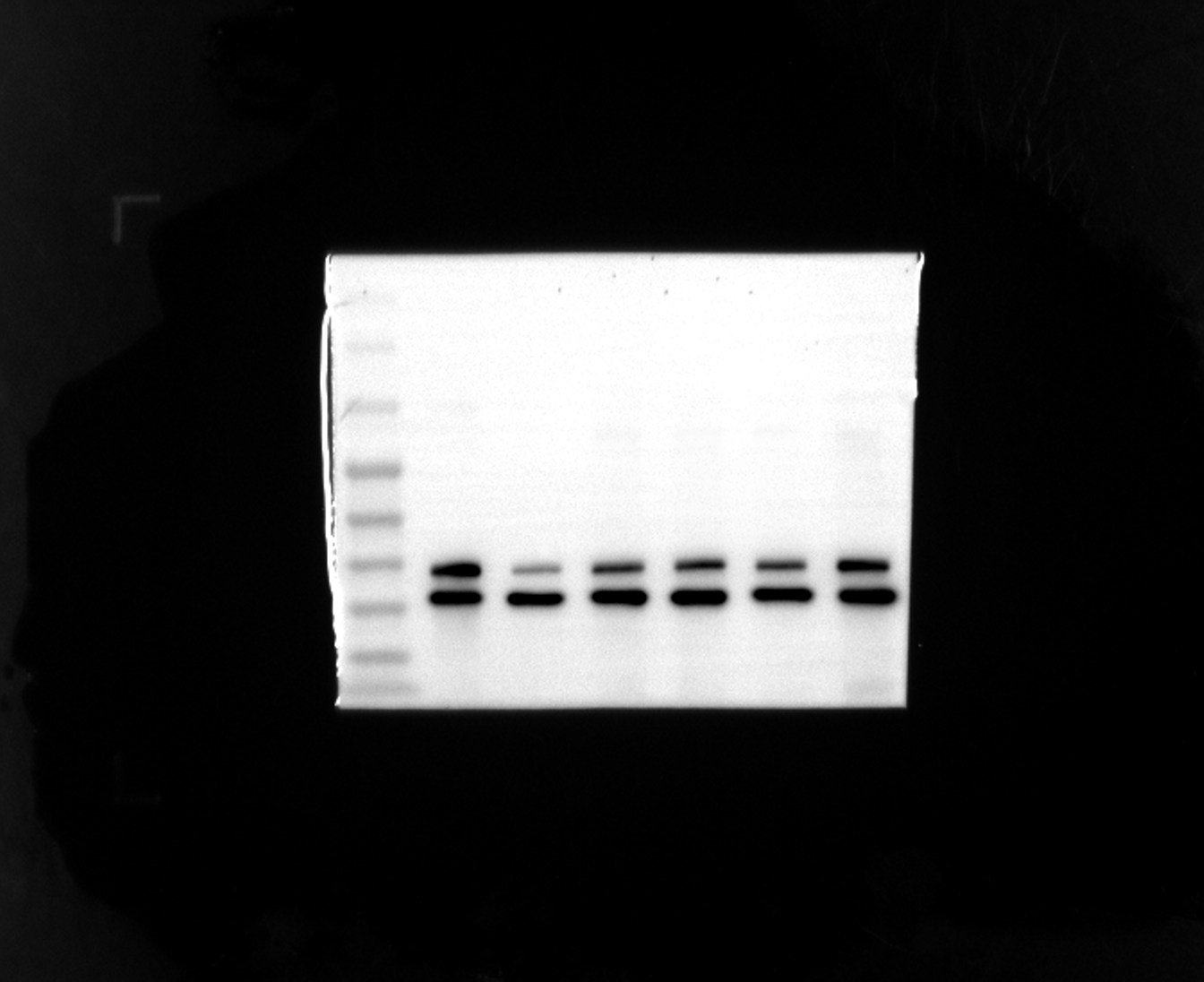

Supplement: Supplementary file 1 [file DataSheet3.zip › 2/BCAT2/BCAT2 3s.Tif]

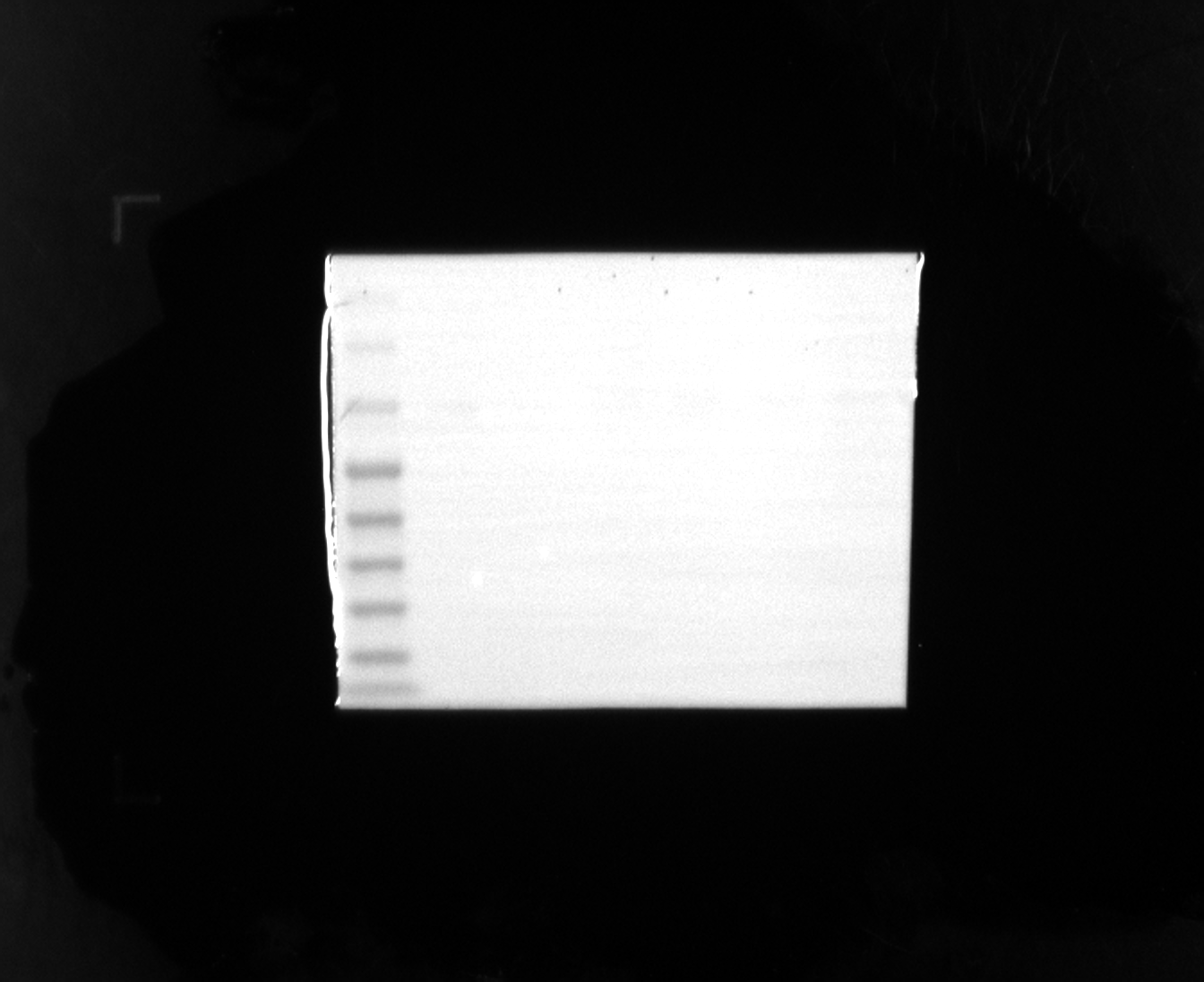

Supplement: Supplementary file 1 [file DataSheet3.zip › 2/BCAT2/marker.Tif]

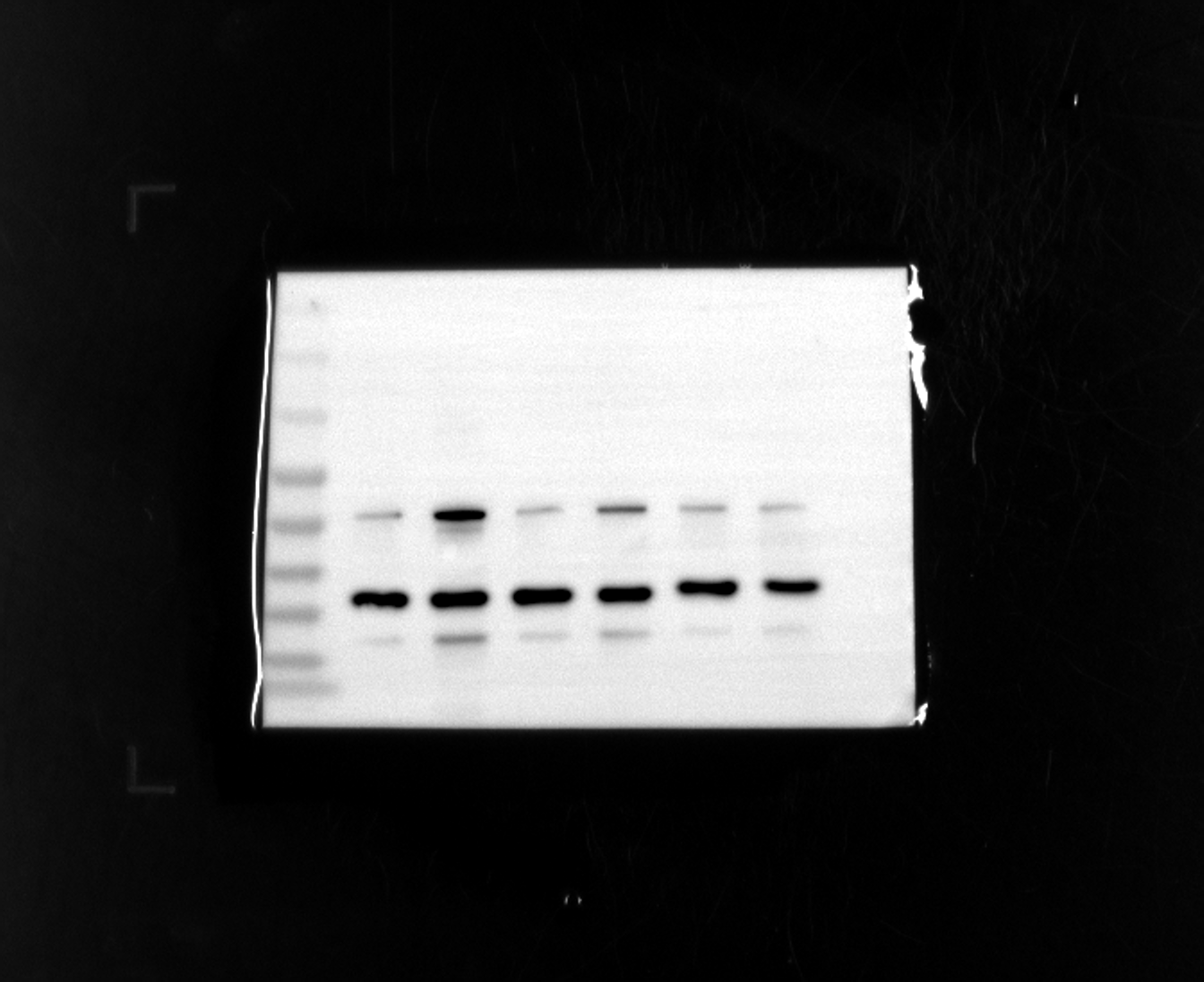

Supplement: Supplementary file 1 [file DataSheet3.zip › 2/Caspase-8/Caspase-8 1s.Tif]

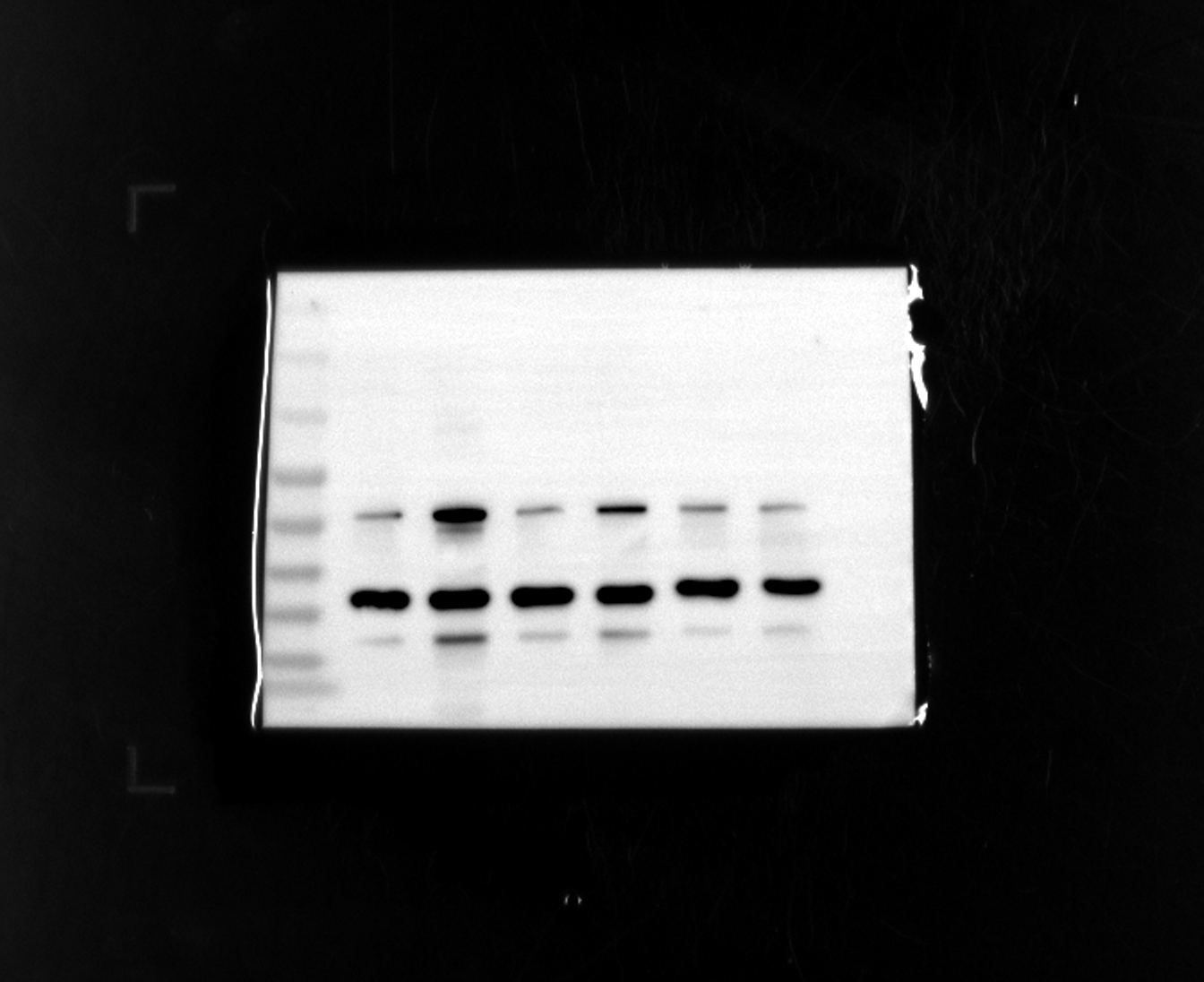

Supplement: Supplementary file 1 [file DataSheet3.zip › 2/Caspase-8/Caspase-8 3s.Tif]

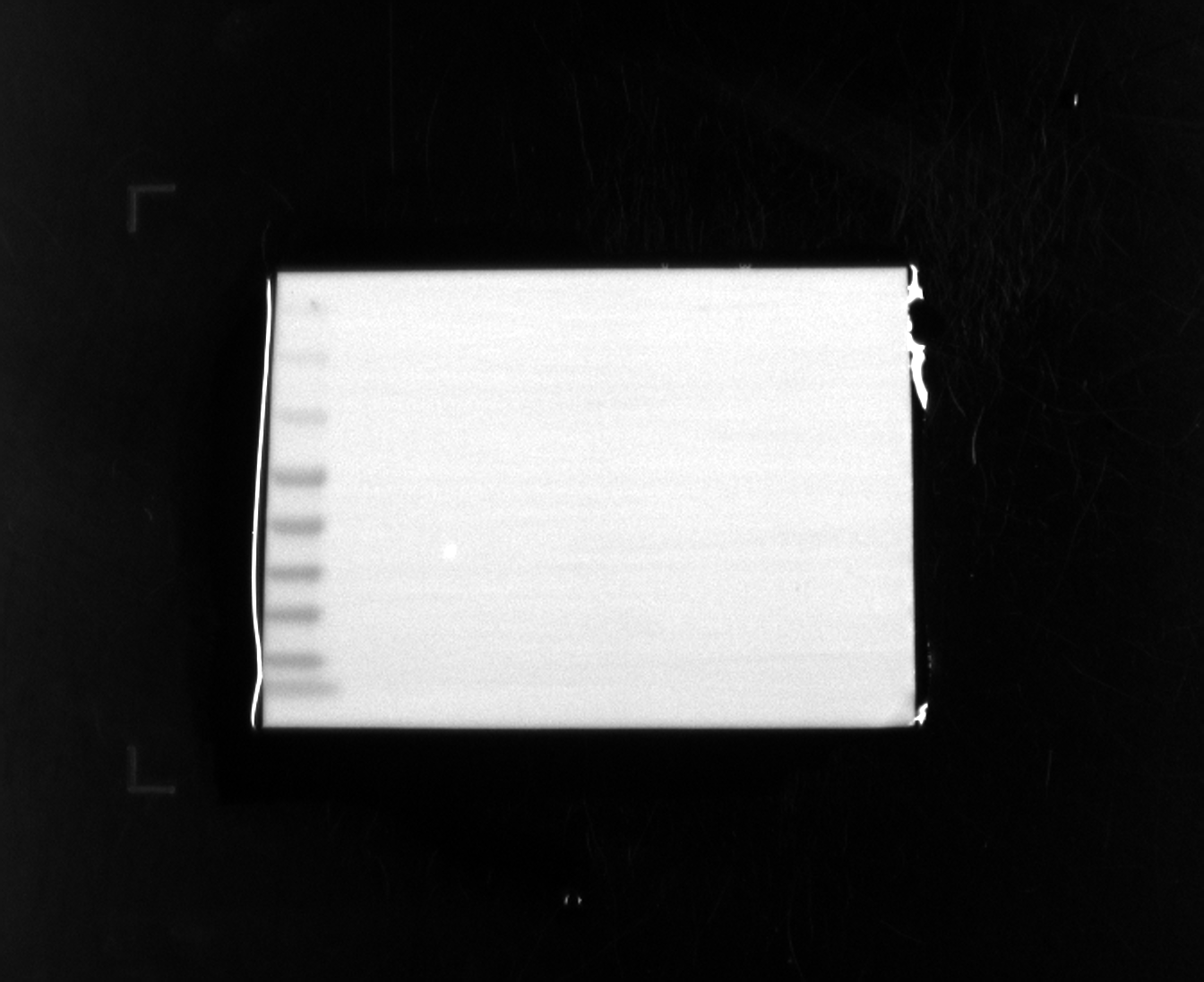

Supplement: Supplementary file 1 [file DataSheet3.zip › 2/Caspase-8/marker.Tif]

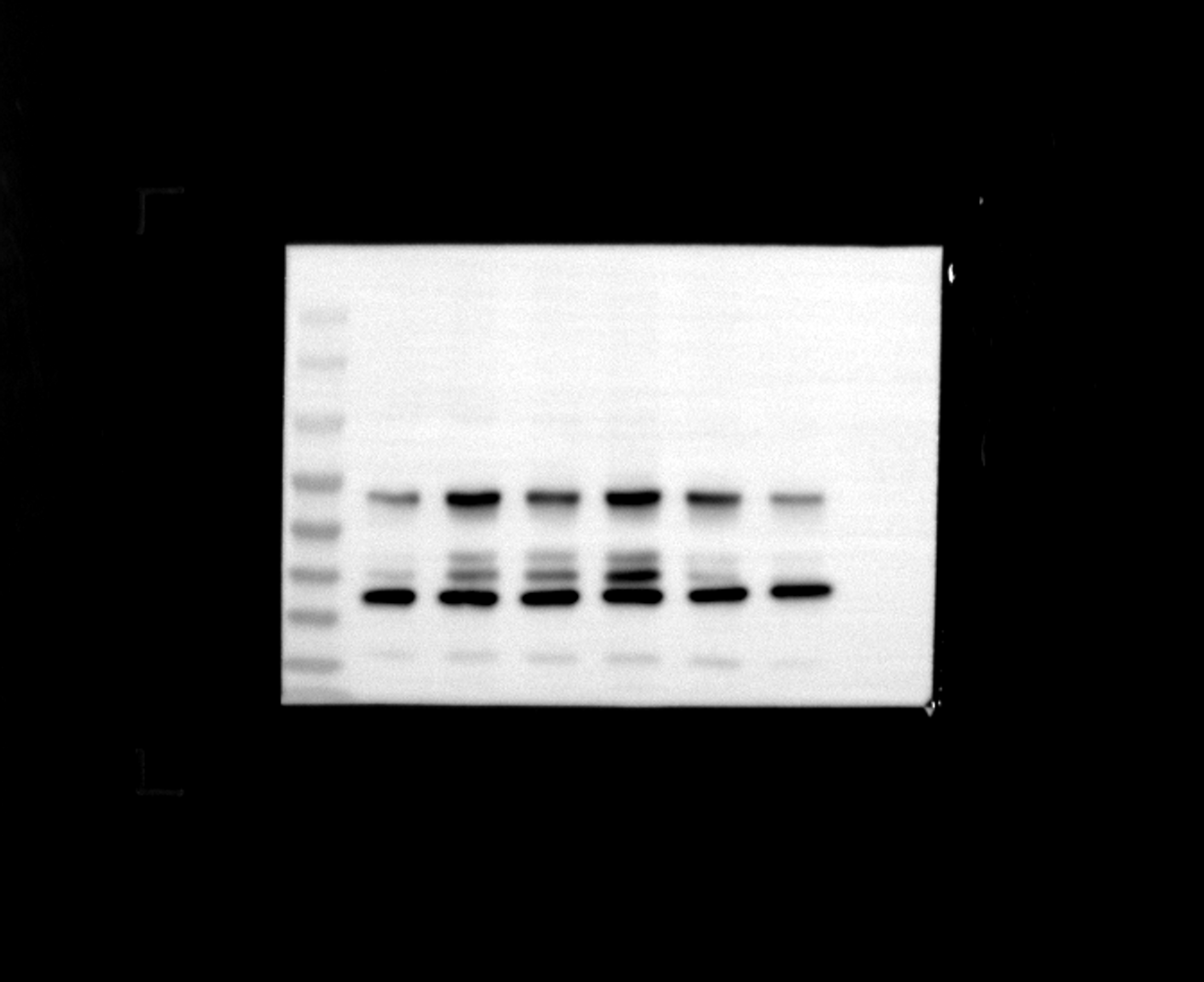

Supplement: Supplementary file 1 [file DataSheet3.zip › 2/EPHX2/EPHX2 1s.Tif]

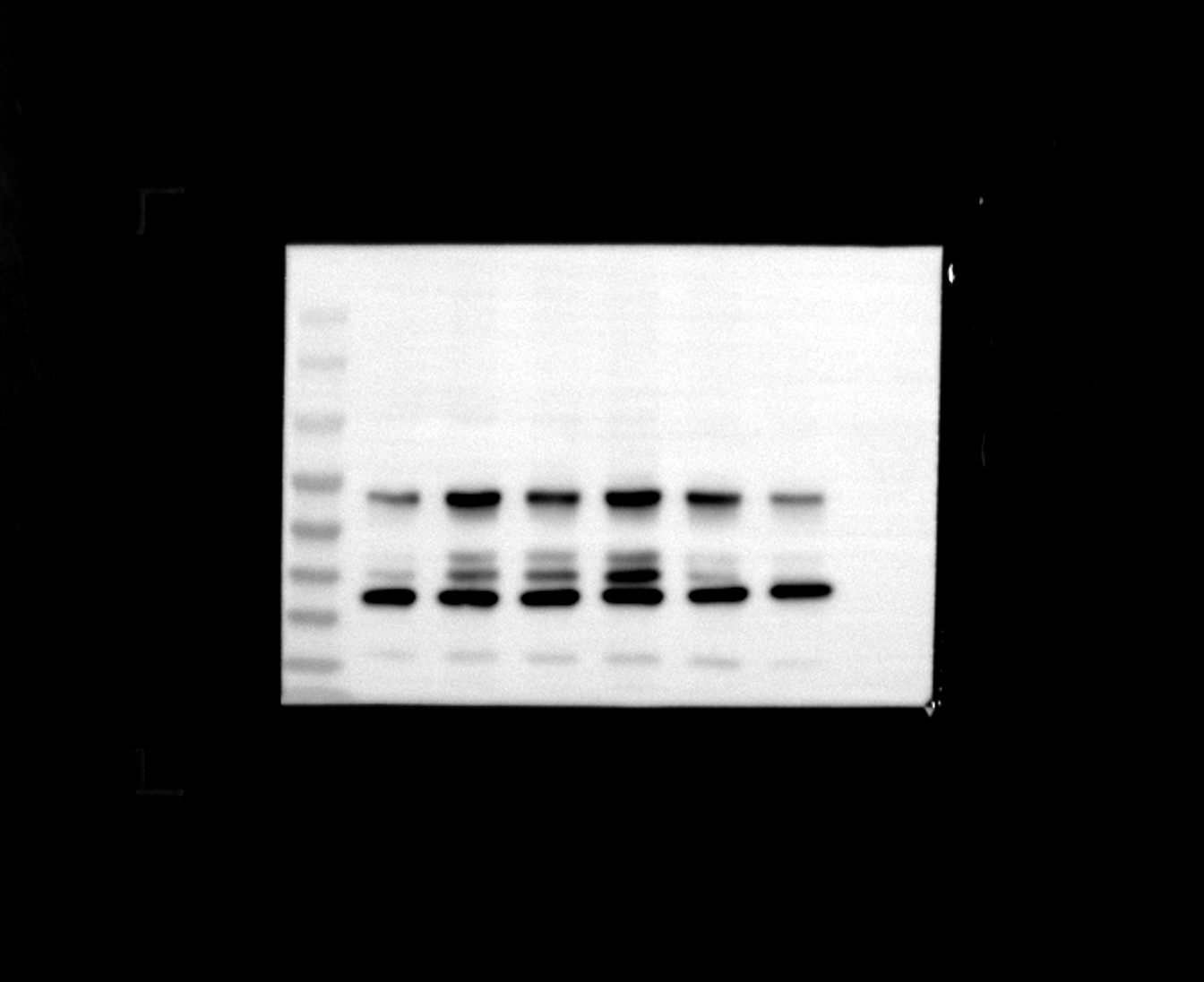

Supplement: Supplementary file 1 [file DataSheet3.zip › 2/EPHX2/EPHX2 3s.Tif]

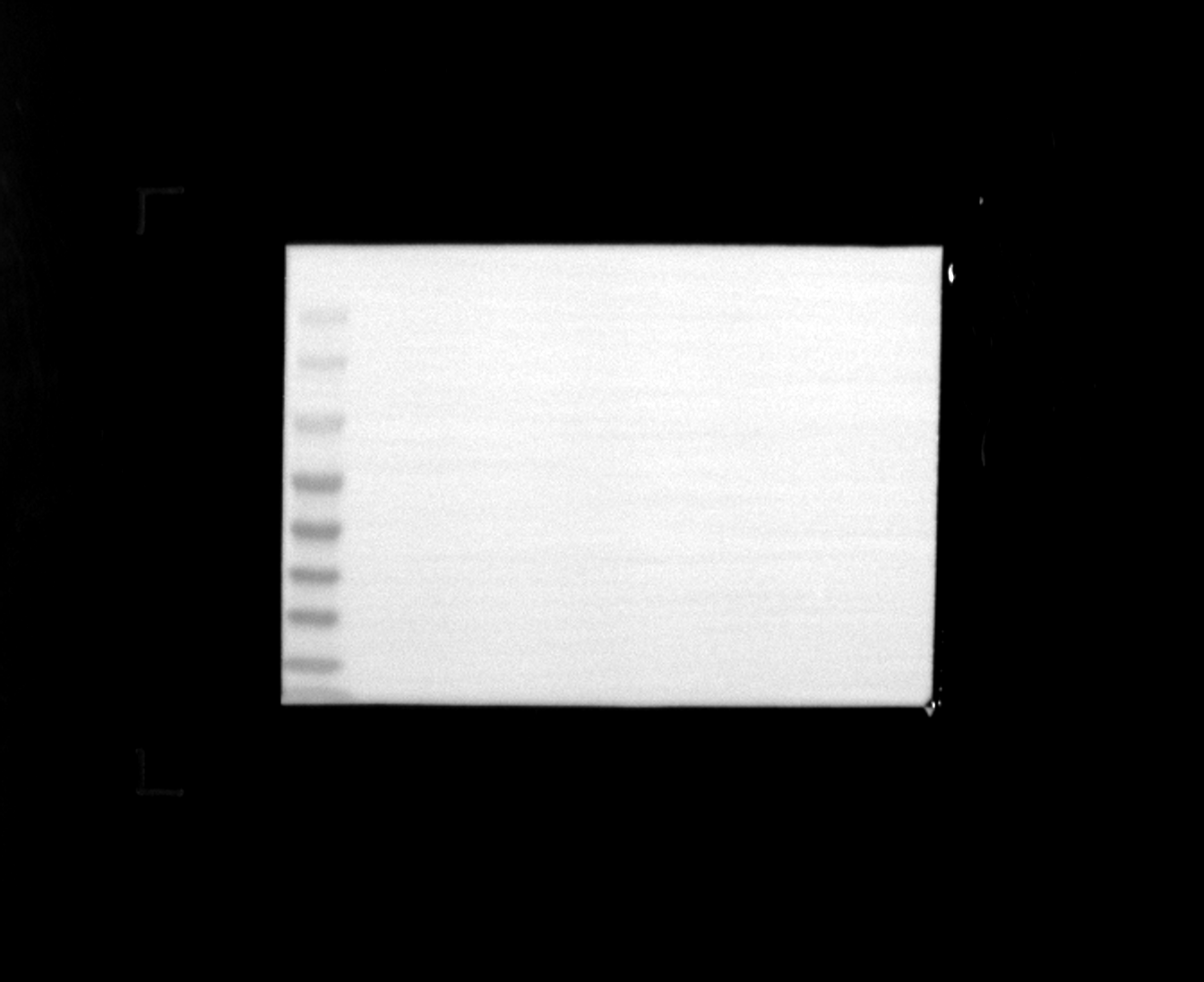

Supplement: Supplementary file 1 [file DataSheet3.zip › 2/EPHX2/marker.Tif]

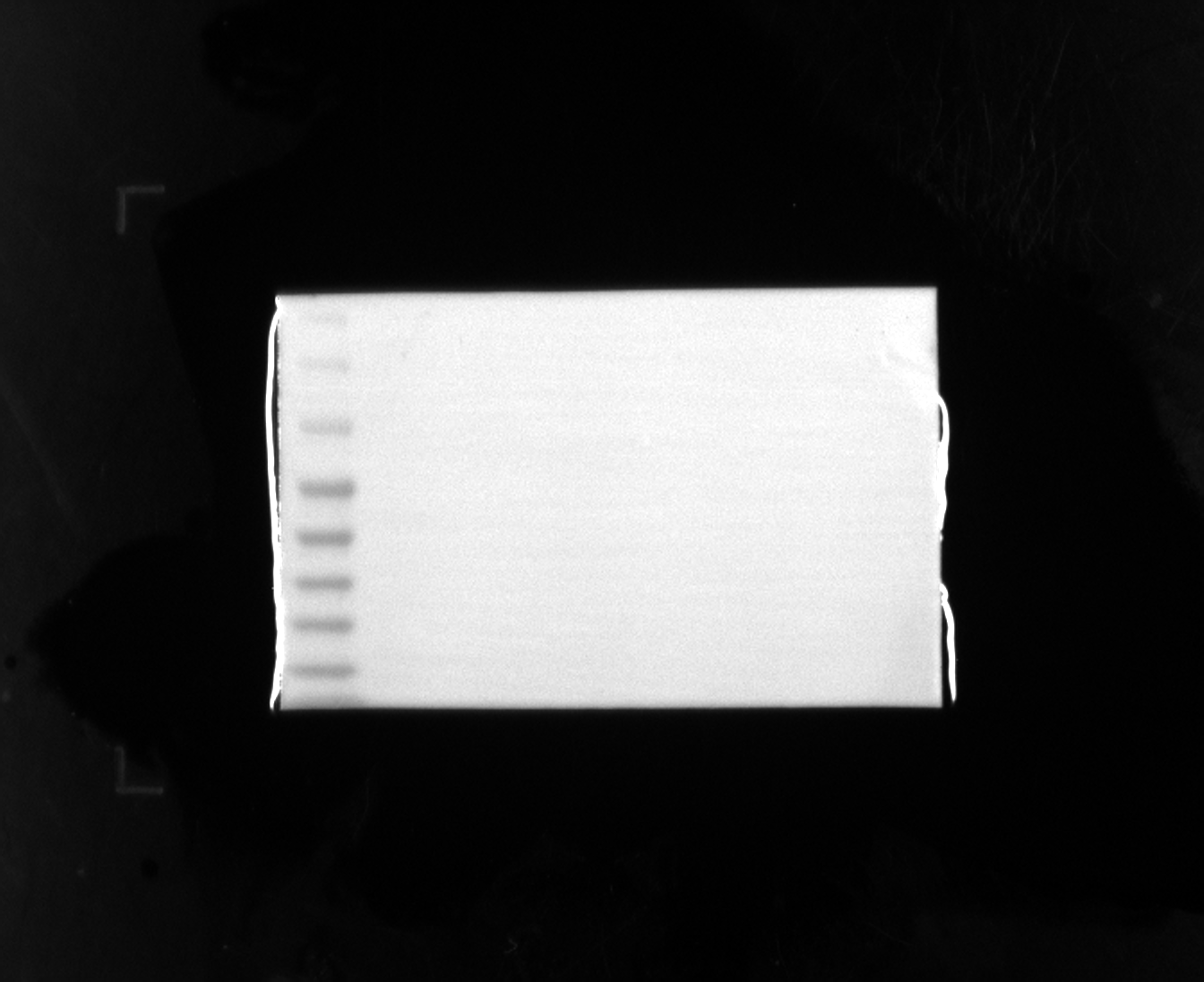

Supplement: Supplementary file 1 [file DataSheet3.zip › 2/UCP2/marker.Tif]

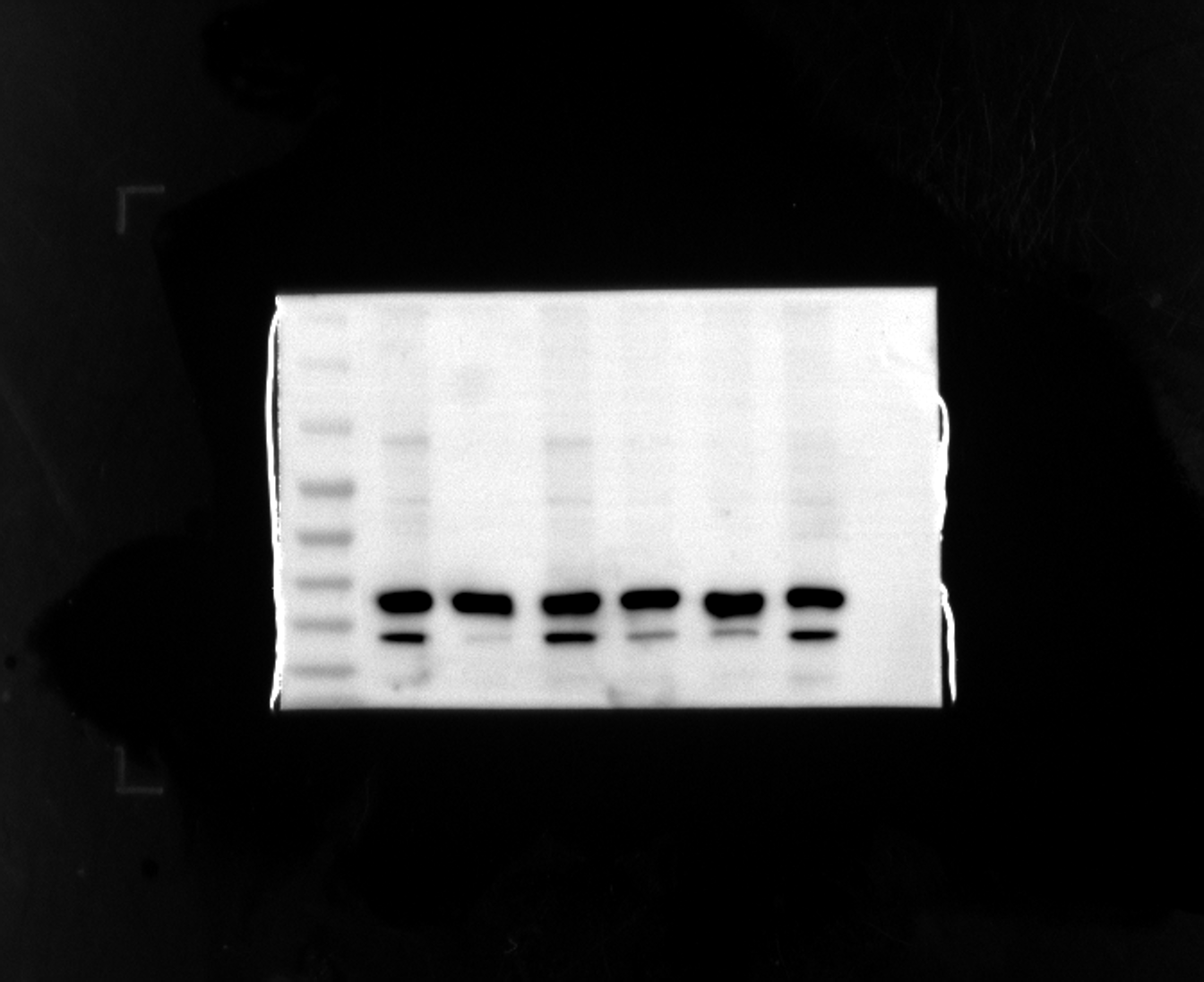

Supplement: Supplementary file 1 [file DataSheet3.zip › 2/UCP2/UCP2 1s.Tif]

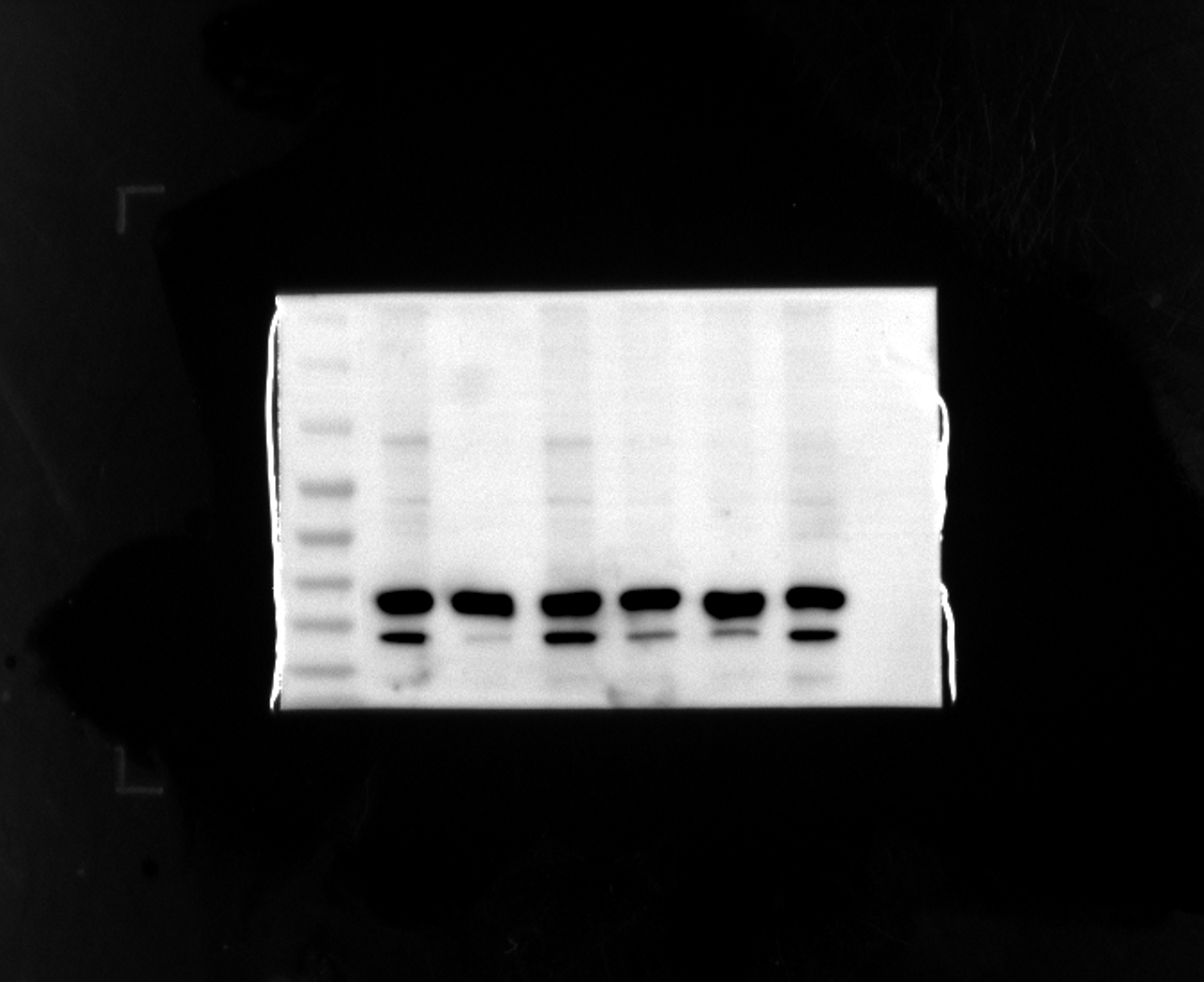

Supplement: Supplementary file 1 [file DataSheet3.zip › 2/UCP2/UCP2 3s.Tif]

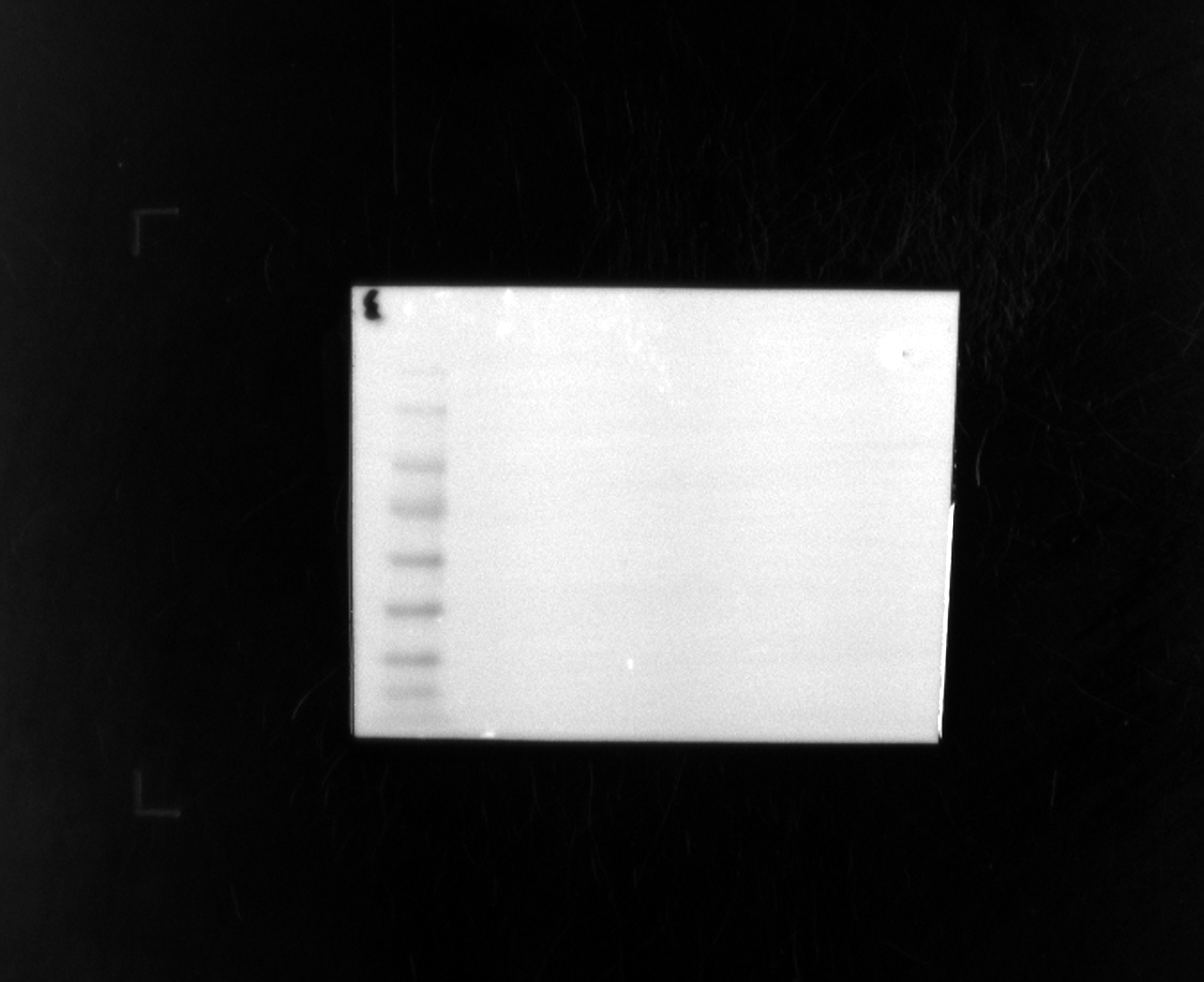

Supplement: Supplementary file 1 [file DataSheet3.zip › 3/BCAT2/marker.Tif]

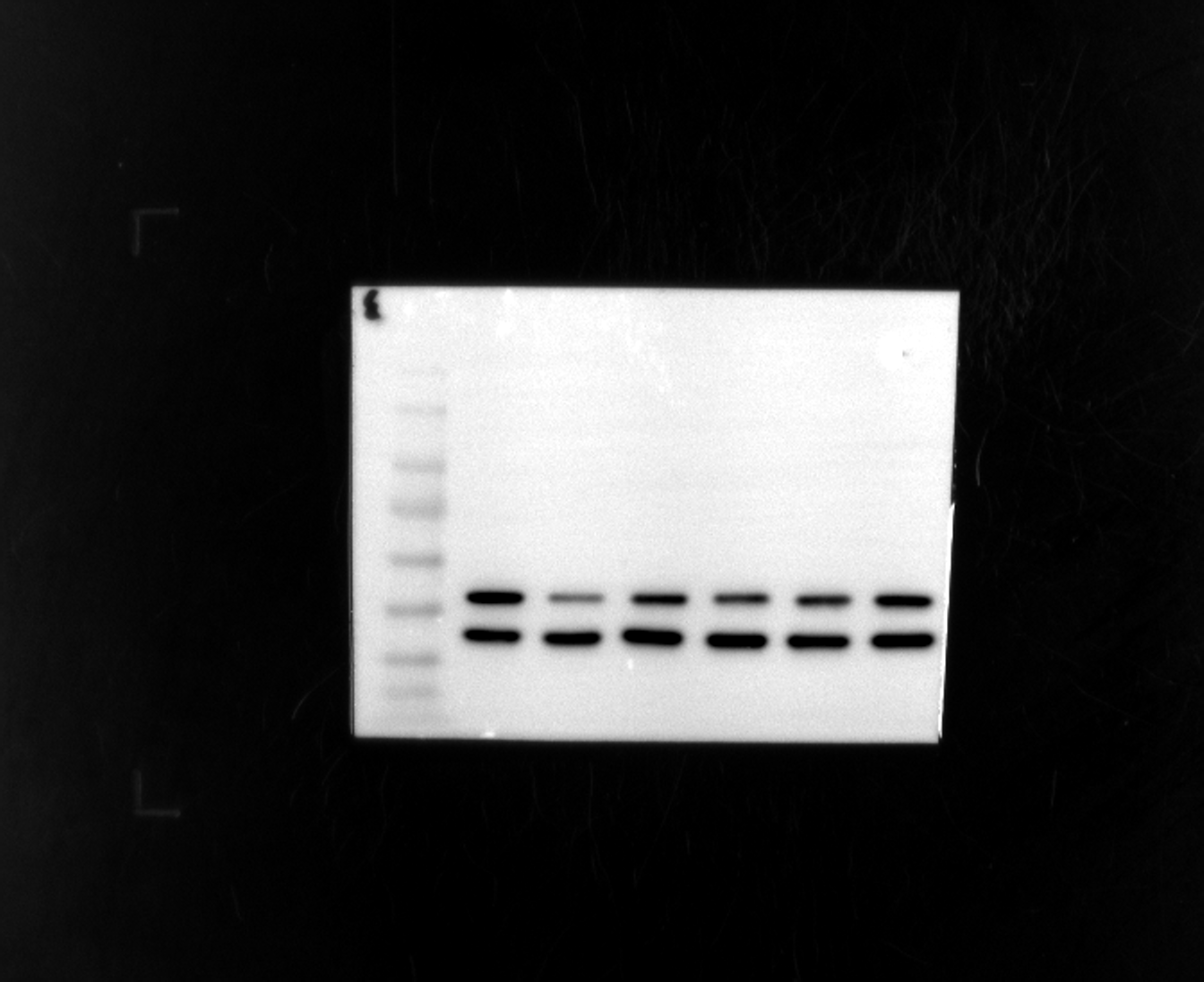

Supplement: Supplementary file 1 [file DataSheet3.zip › 3/BCAT2/merged 1s.Tif]

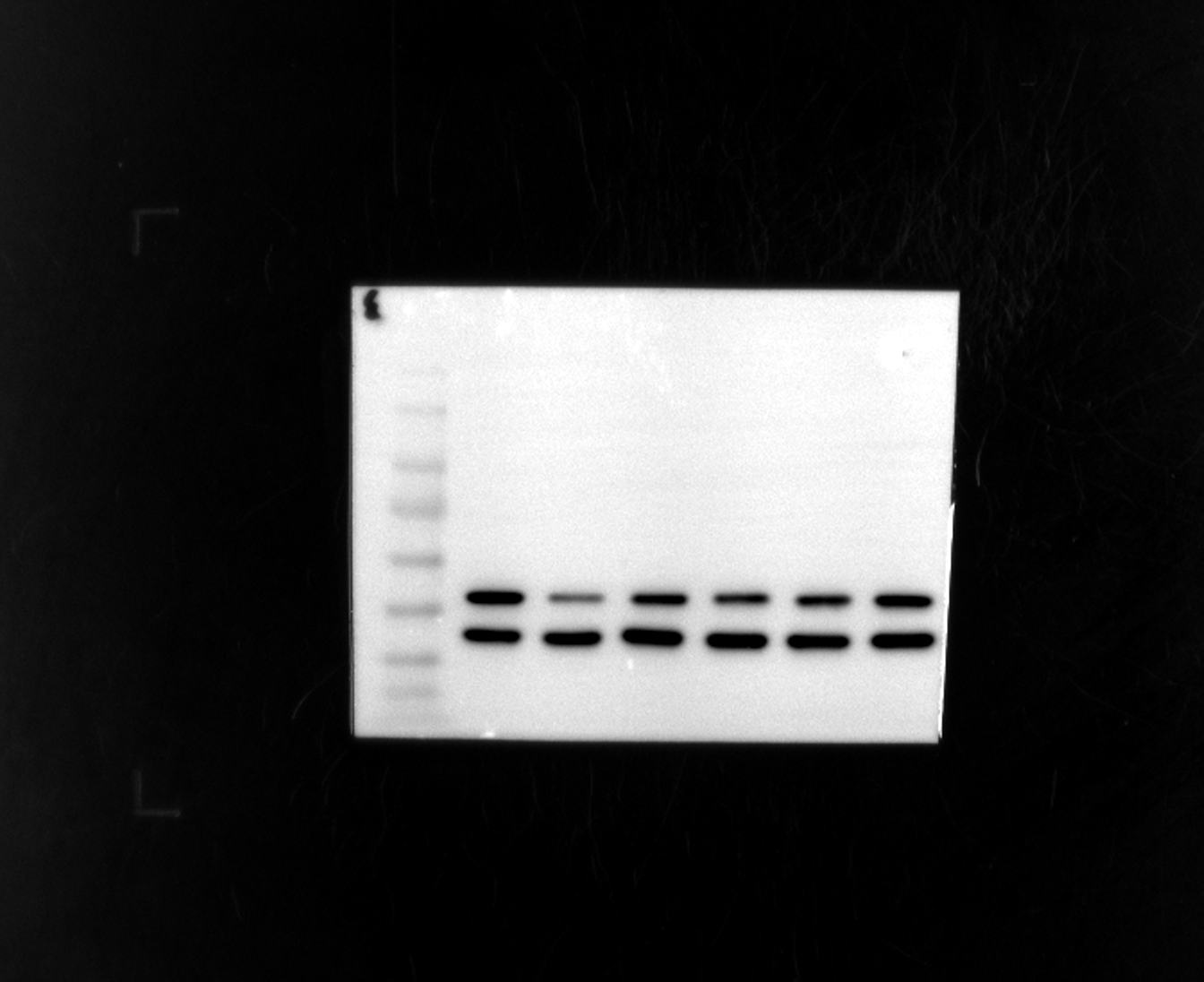

Supplement: Supplementary file 1 [file DataSheet3.zip › 3/BCAT2/merged 3s.Tif]

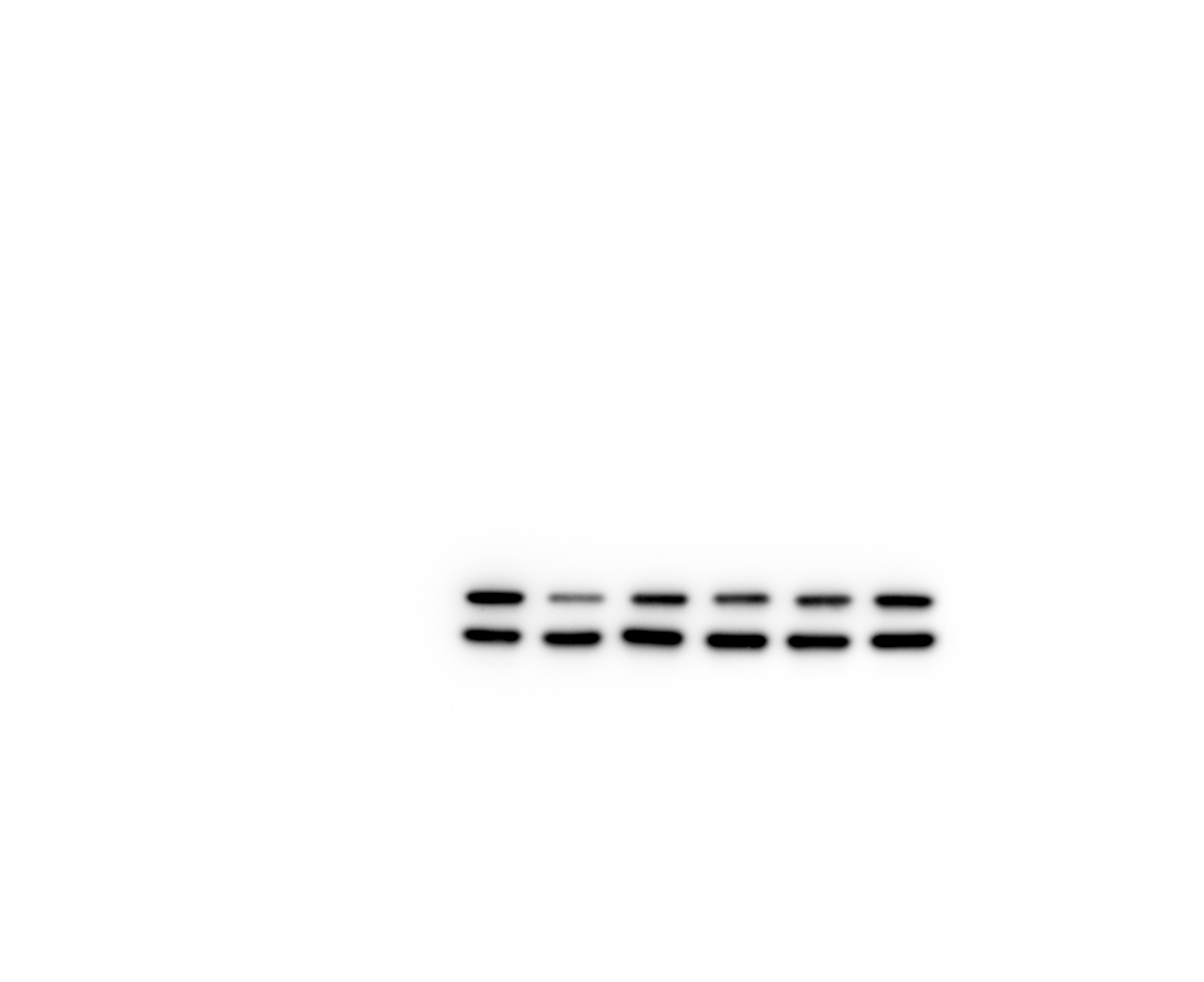

Supplement: Supplementary file 1 [file DataSheet3.zip › 3/BCAT2/UCP2 1s.Tif]

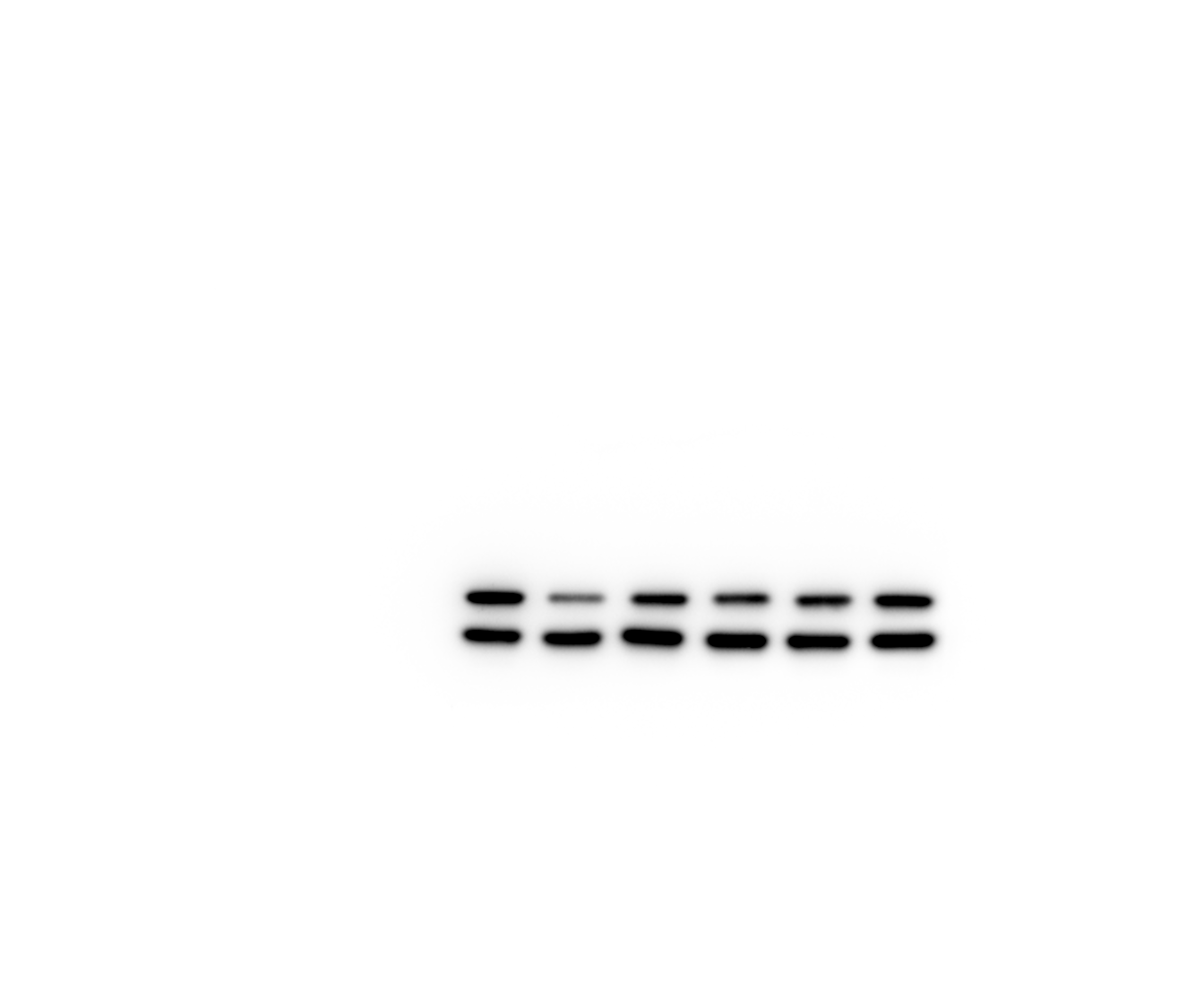

Supplement: Supplementary file 1 [file DataSheet3.zip › 3/BCAT2/UCP2 3s.Tif]

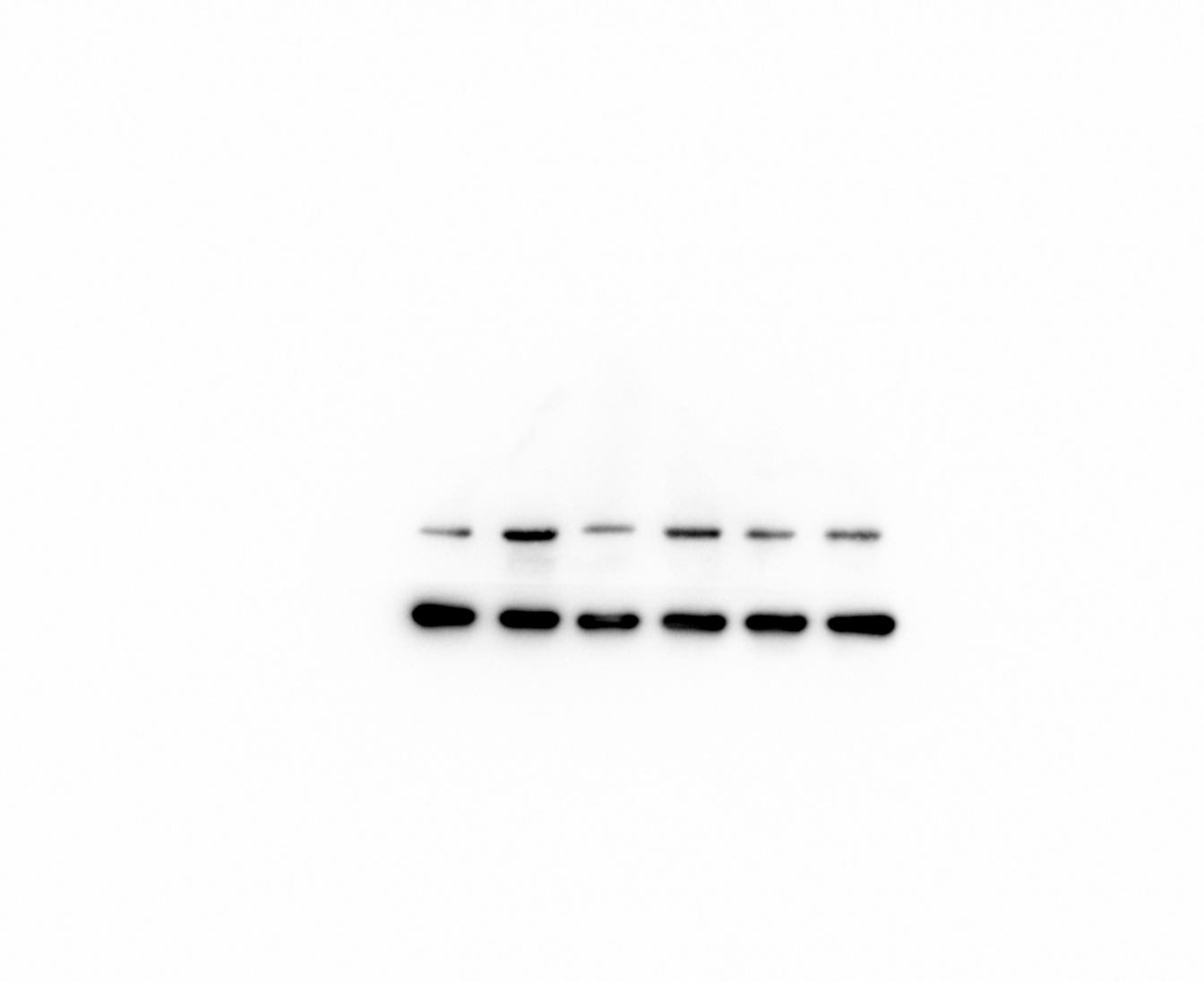

Supplement: Supplementary file 1 [file DataSheet3.zip › 3/Caspase-8/Caspase-8 1s.Tif]

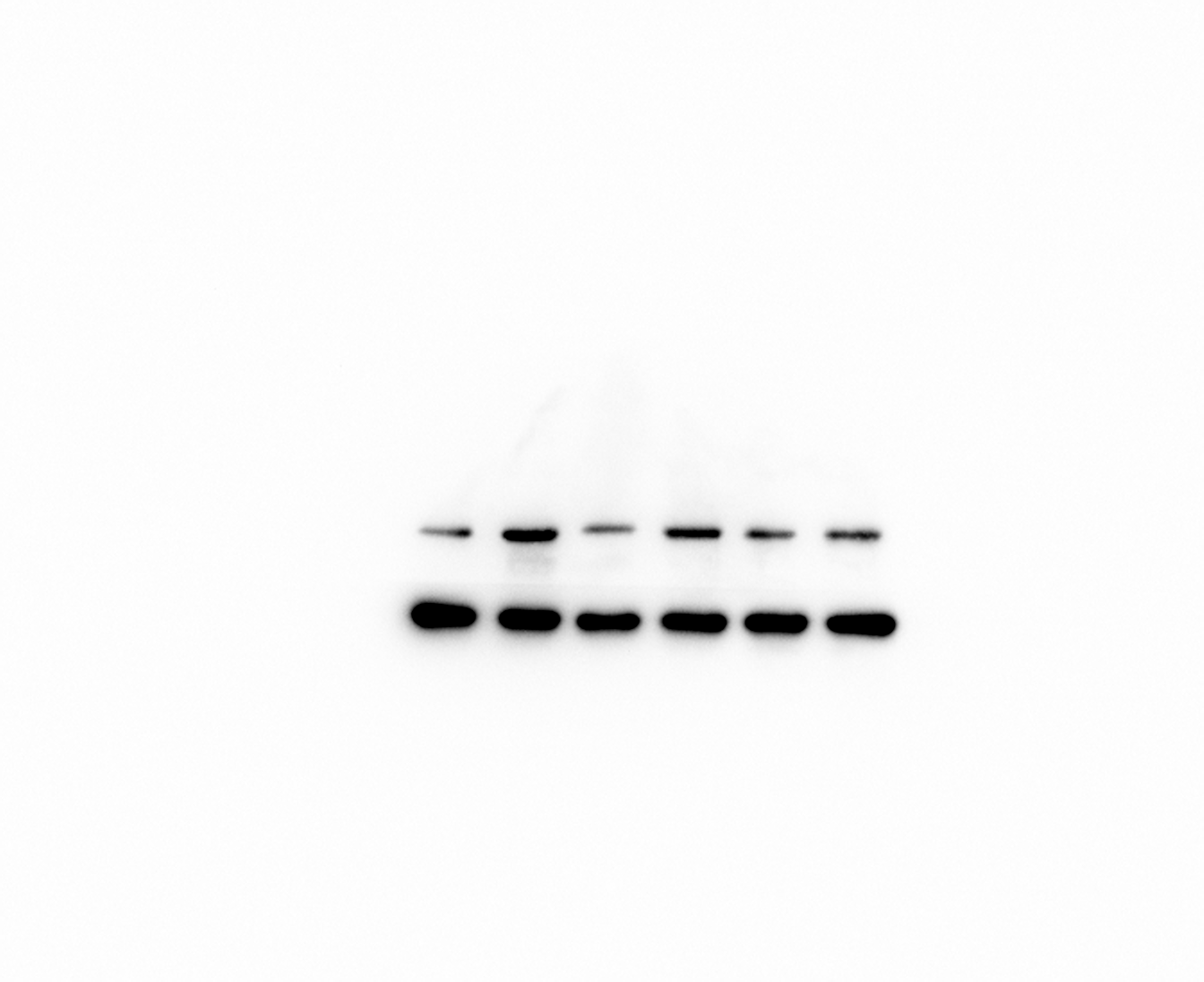

Supplement: Supplementary file 1 [file DataSheet3.zip › 3/Caspase-8/Caspase-8 3s.Tif]

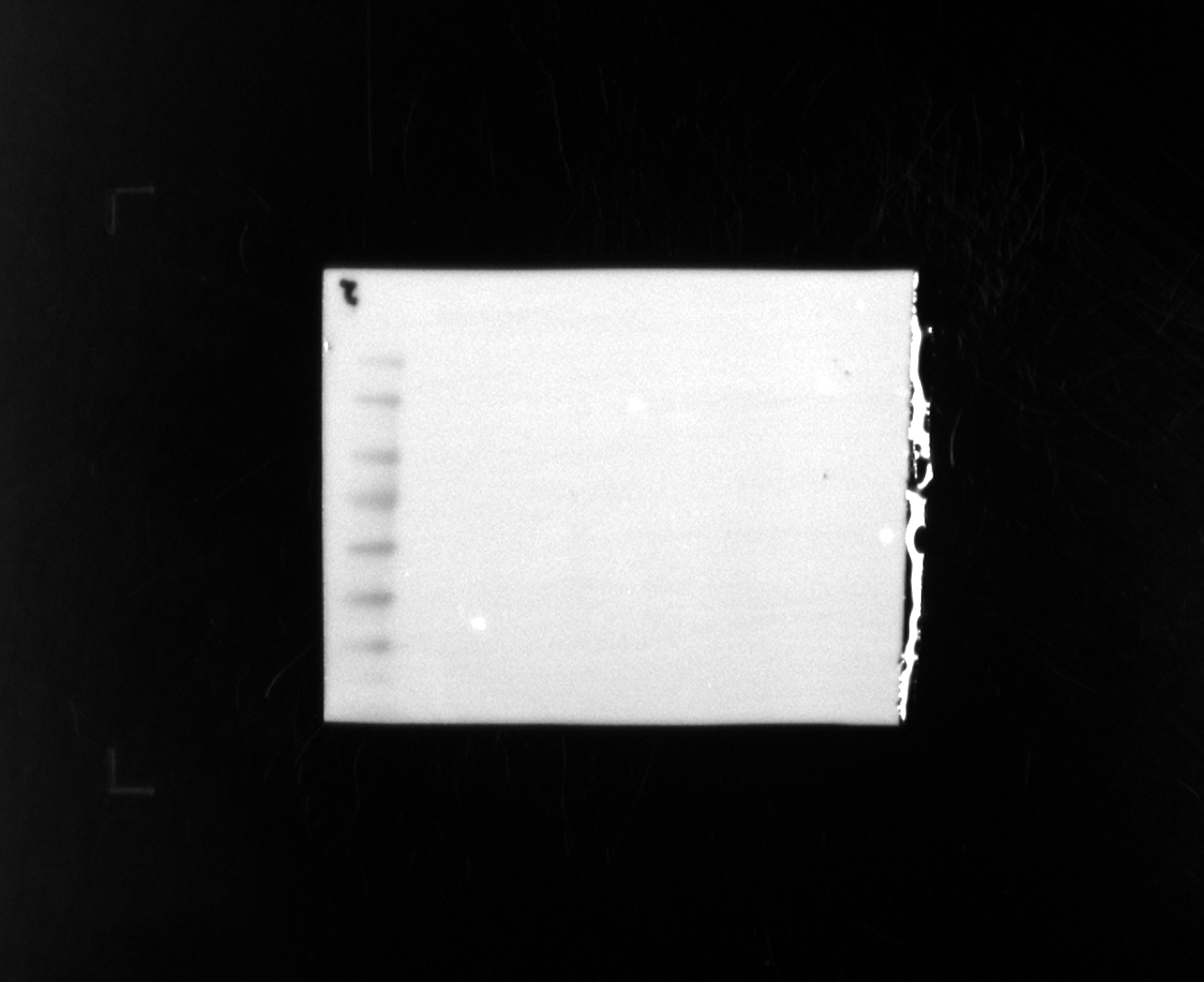

Supplement: Supplementary file 1 [file DataSheet3.zip › 3/Caspase-8/marker.Tif]

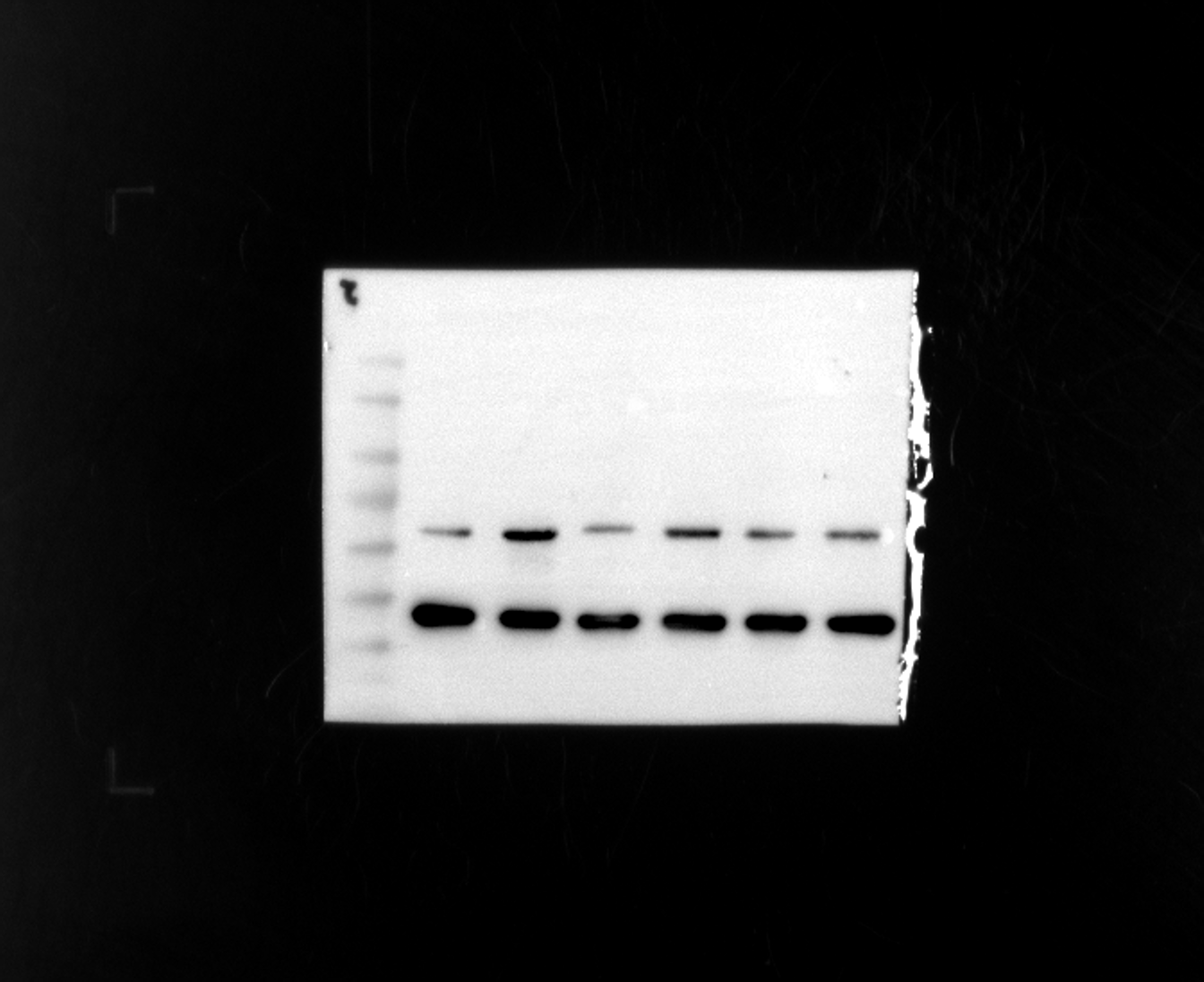

Supplement: Supplementary file 1 [file DataSheet3.zip › 3/Caspase-8/merged 1s.Tif]

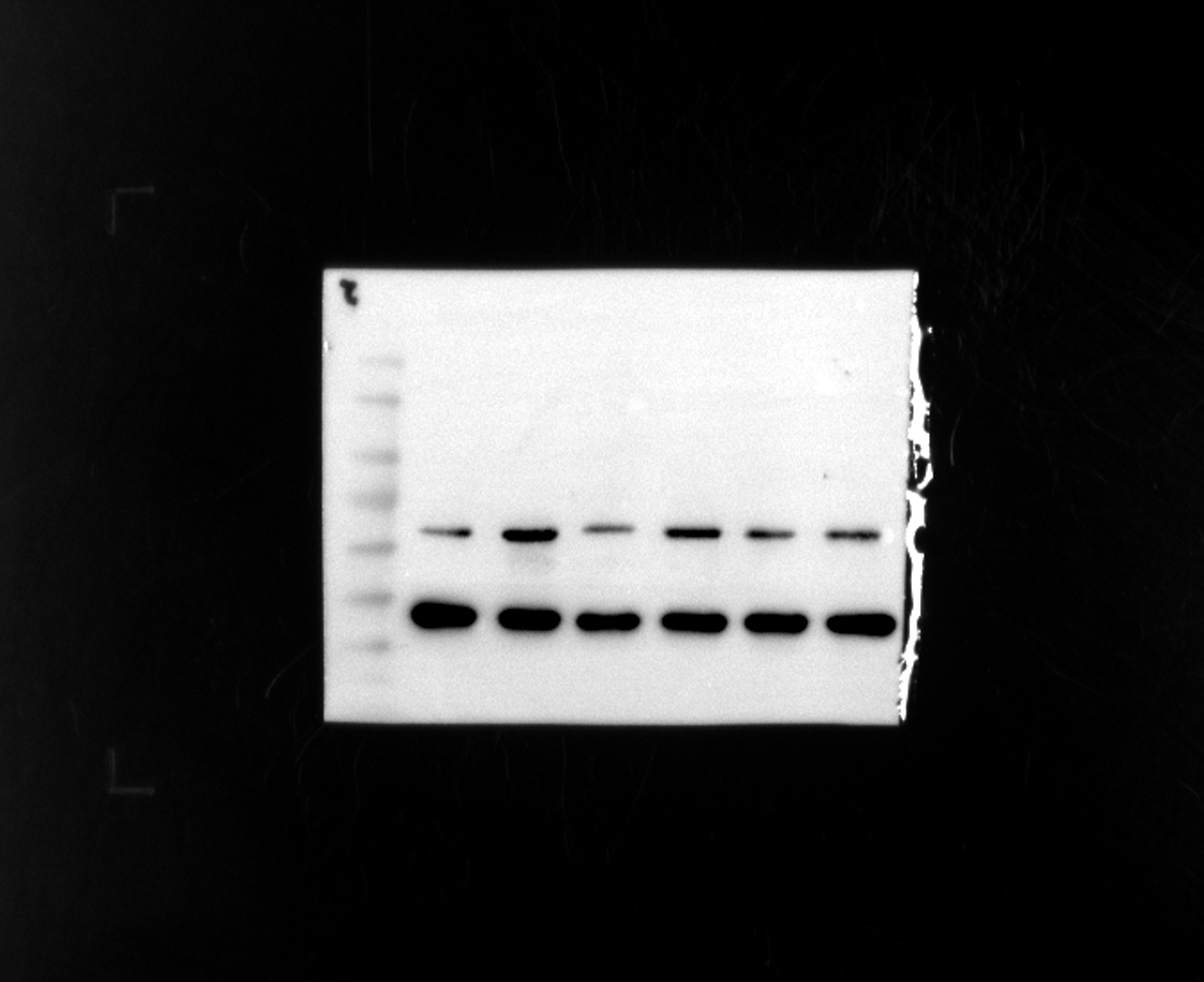

Supplement: Supplementary file 1 [file DataSheet3.zip › 3/Caspase-8/merged 3s.Tif]

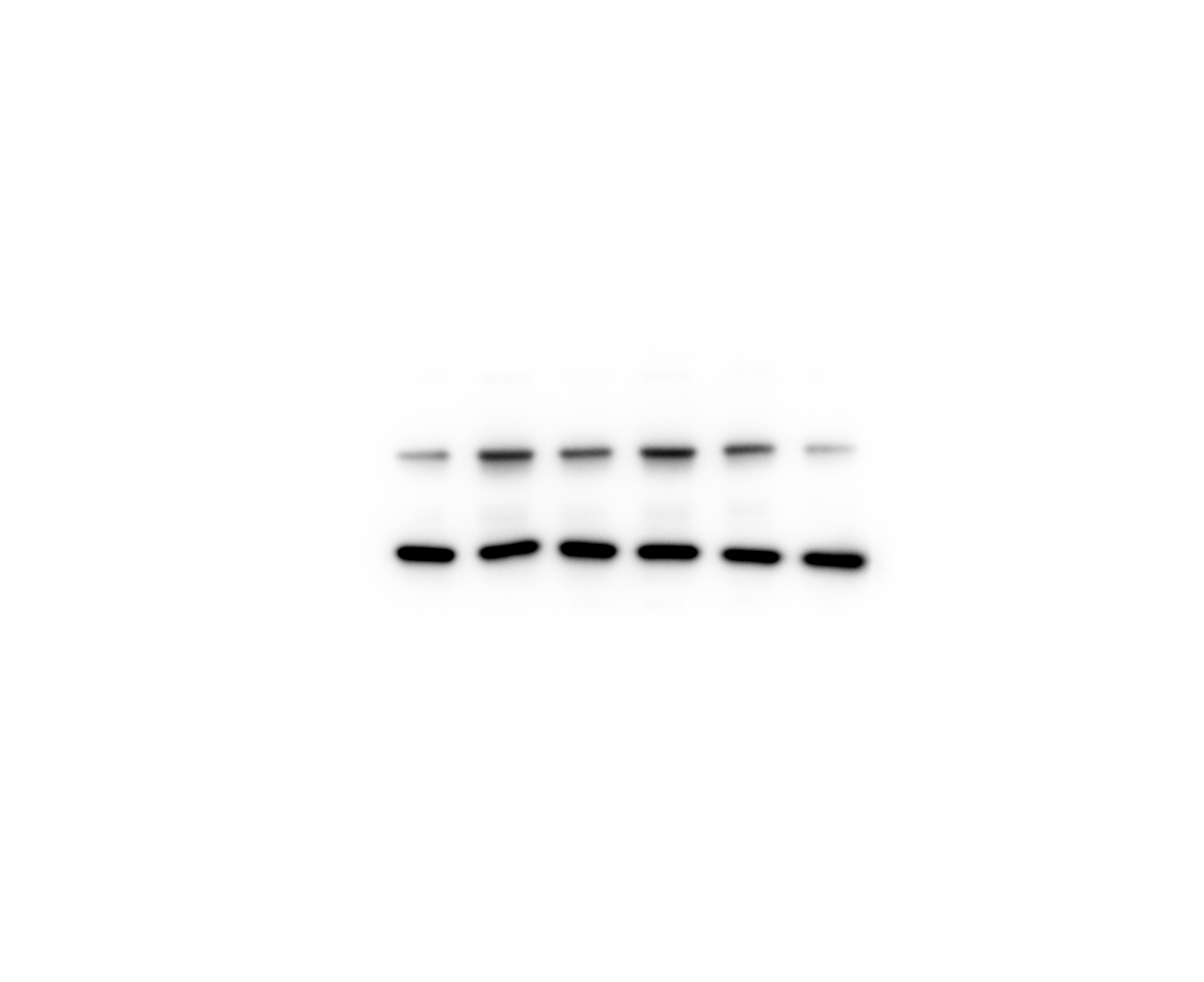

Supplement: Supplementary file 1 [file DataSheet3.zip › 3/EPHX2/EPHX2 1s.Tif]

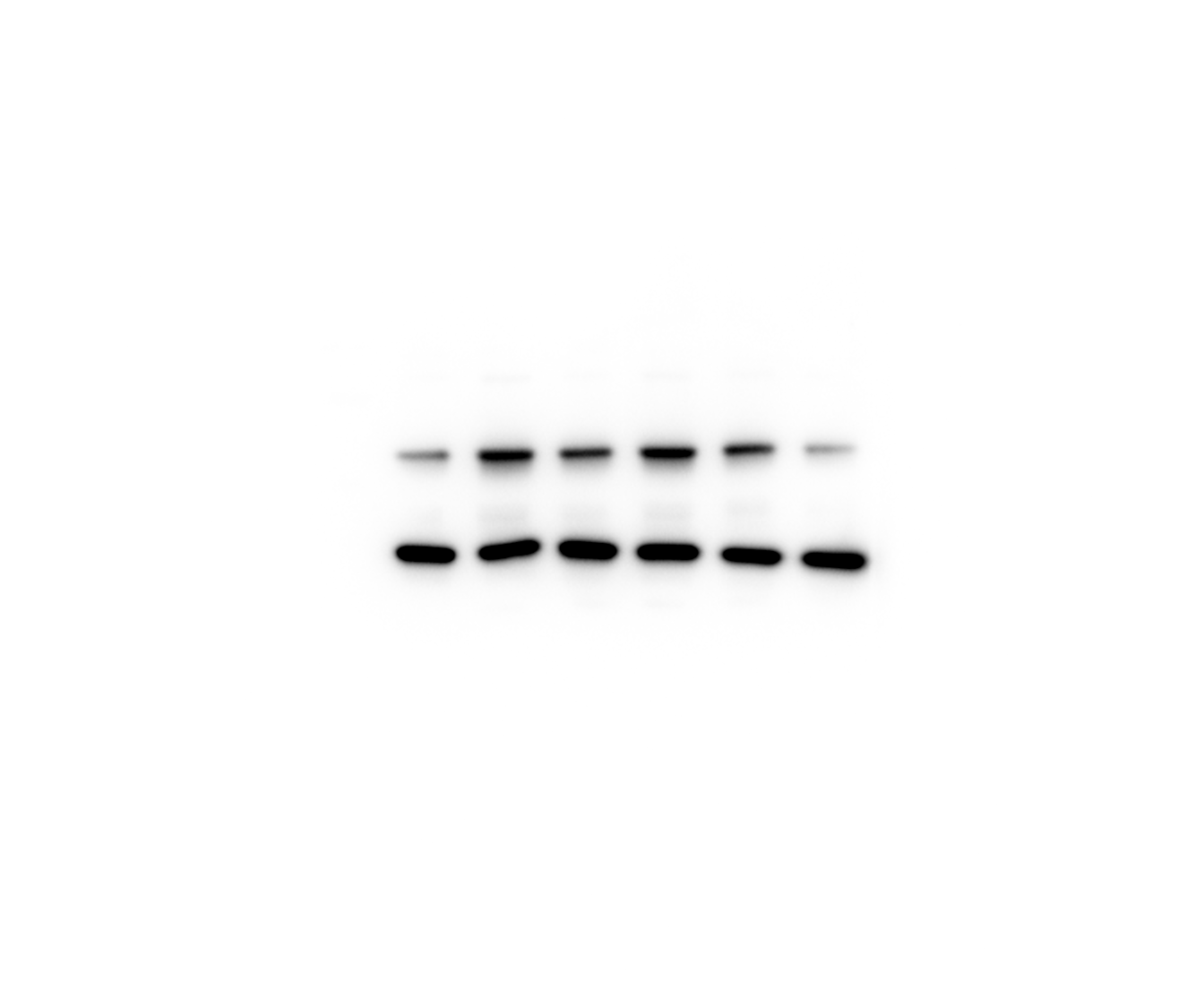

Supplement: Supplementary file 1 [file DataSheet3.zip › 3/EPHX2/EPHX2 3s.Tif]

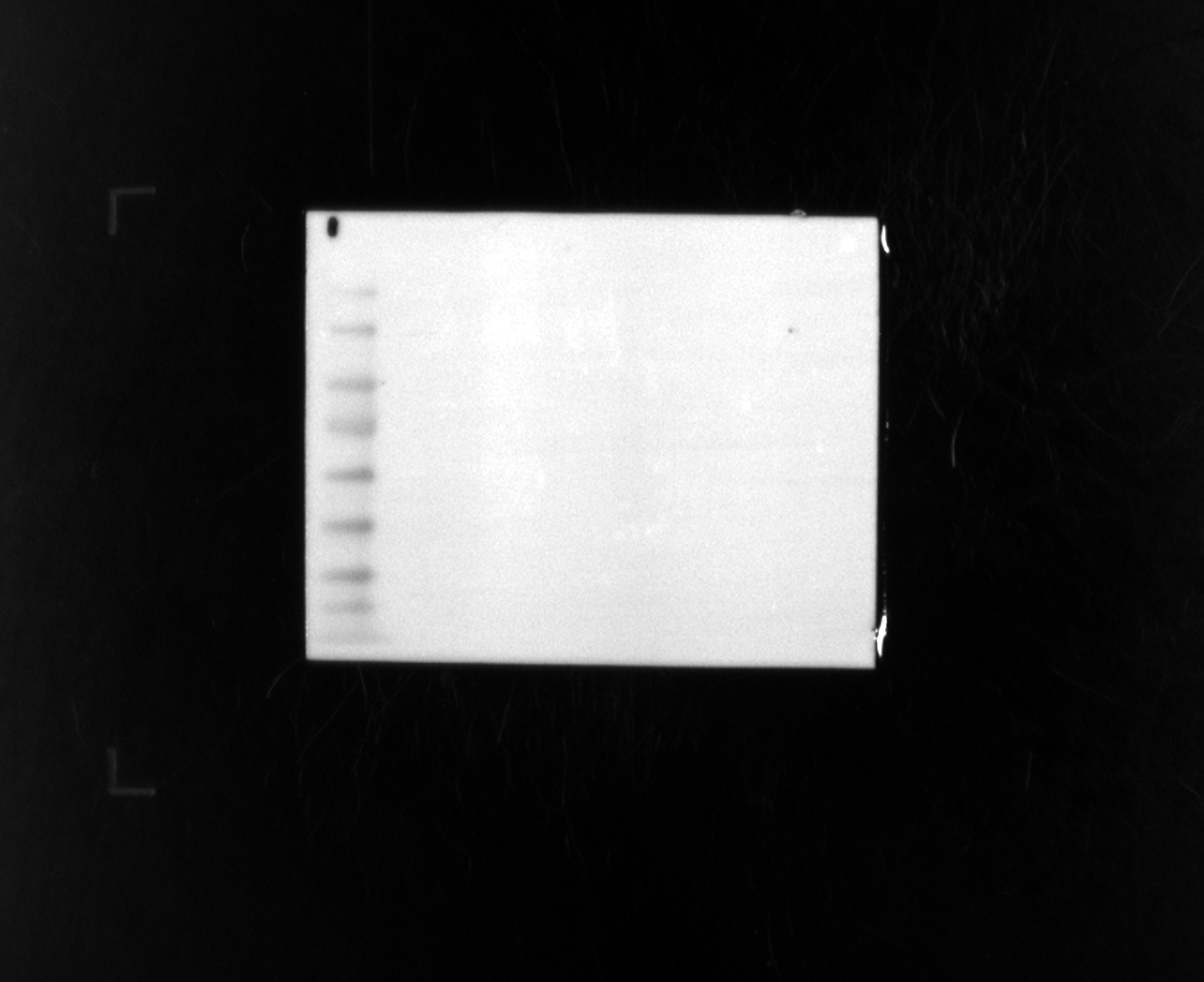

Supplement: Supplementary file 1 [file DataSheet3.zip › 3/EPHX2/marker.Tif]

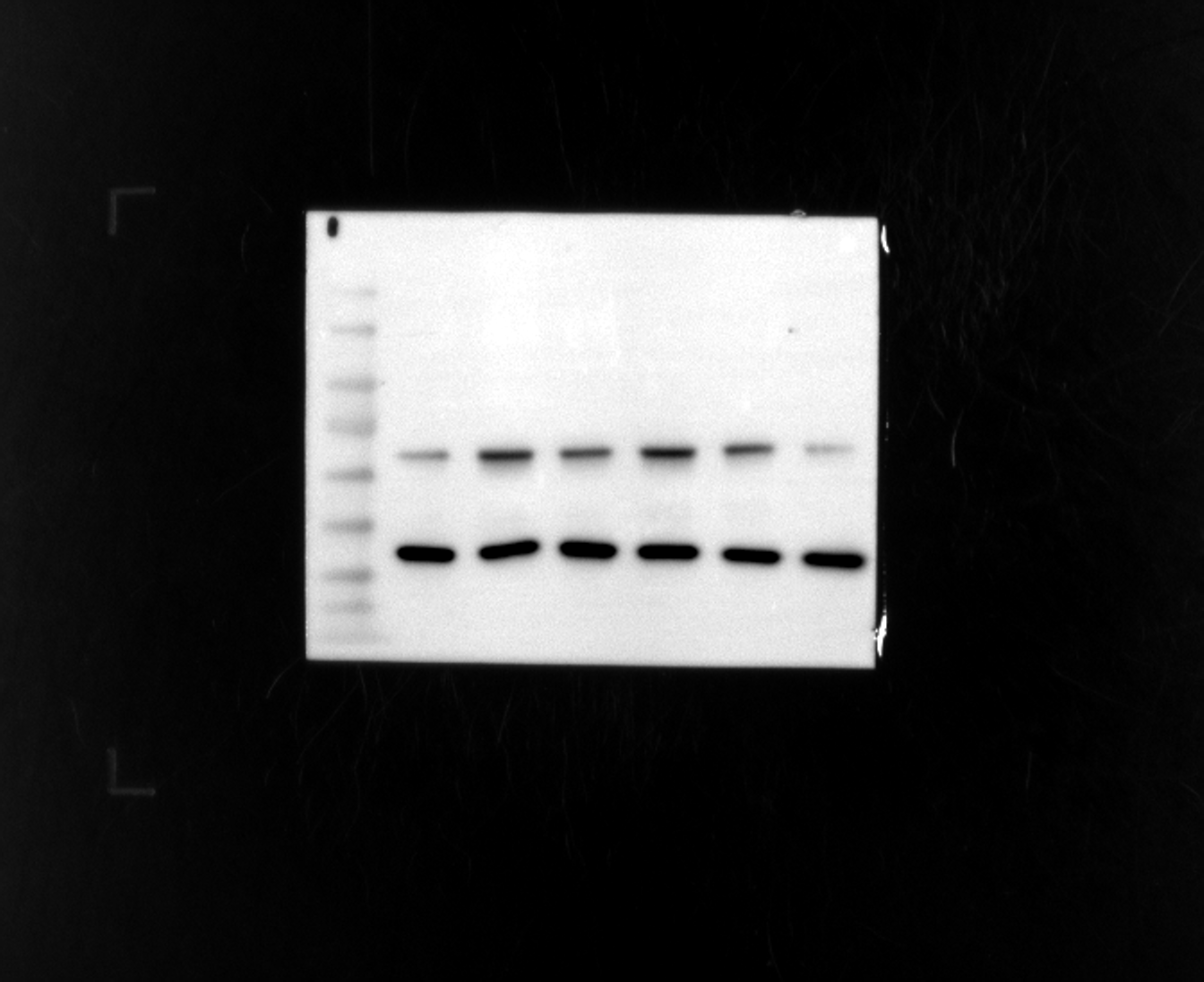

Supplement: Supplementary file 1 [file DataSheet3.zip › 3/EPHX2/merged 1s.Tif]

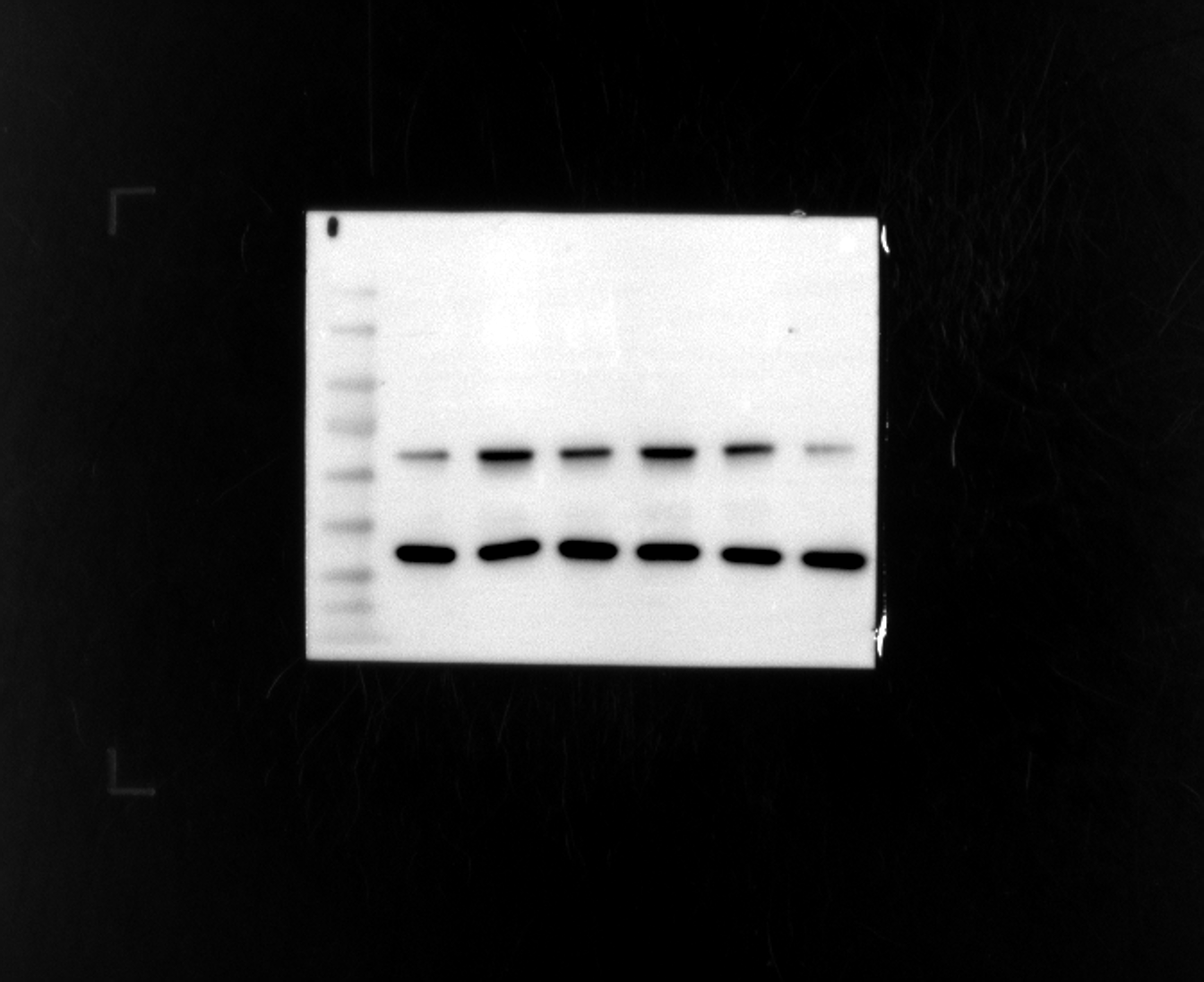

Supplement: Supplementary file 1 [file DataSheet3.zip › 3/EPHX2/merged 3s.Tif]

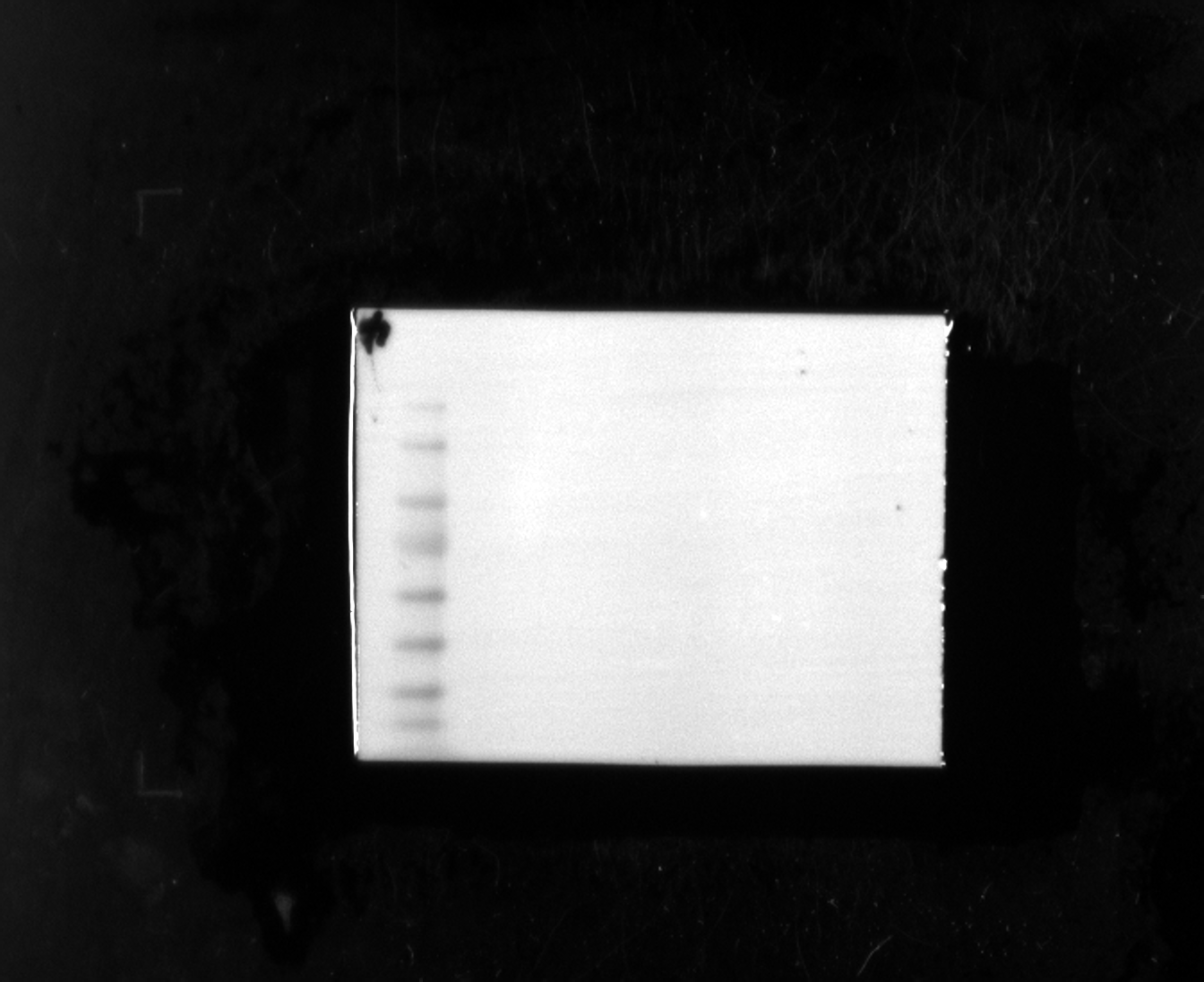

Supplement: Supplementary file 1 [file DataSheet3.zip › 3/UCP2/marker.Tif]

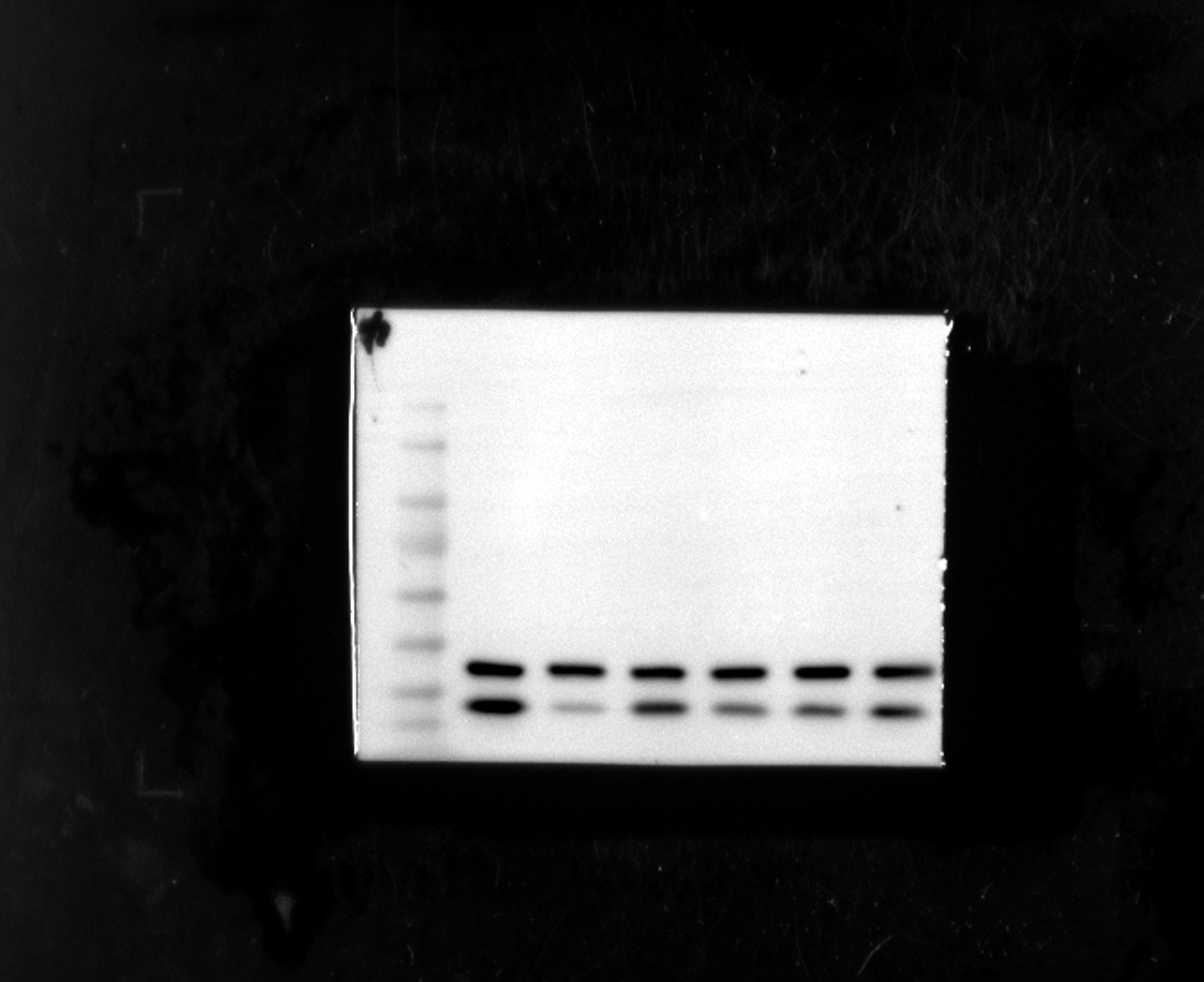

Supplement: Supplementary file 1 [file DataSheet3.zip › 3/UCP2/merged 1s.Tif]

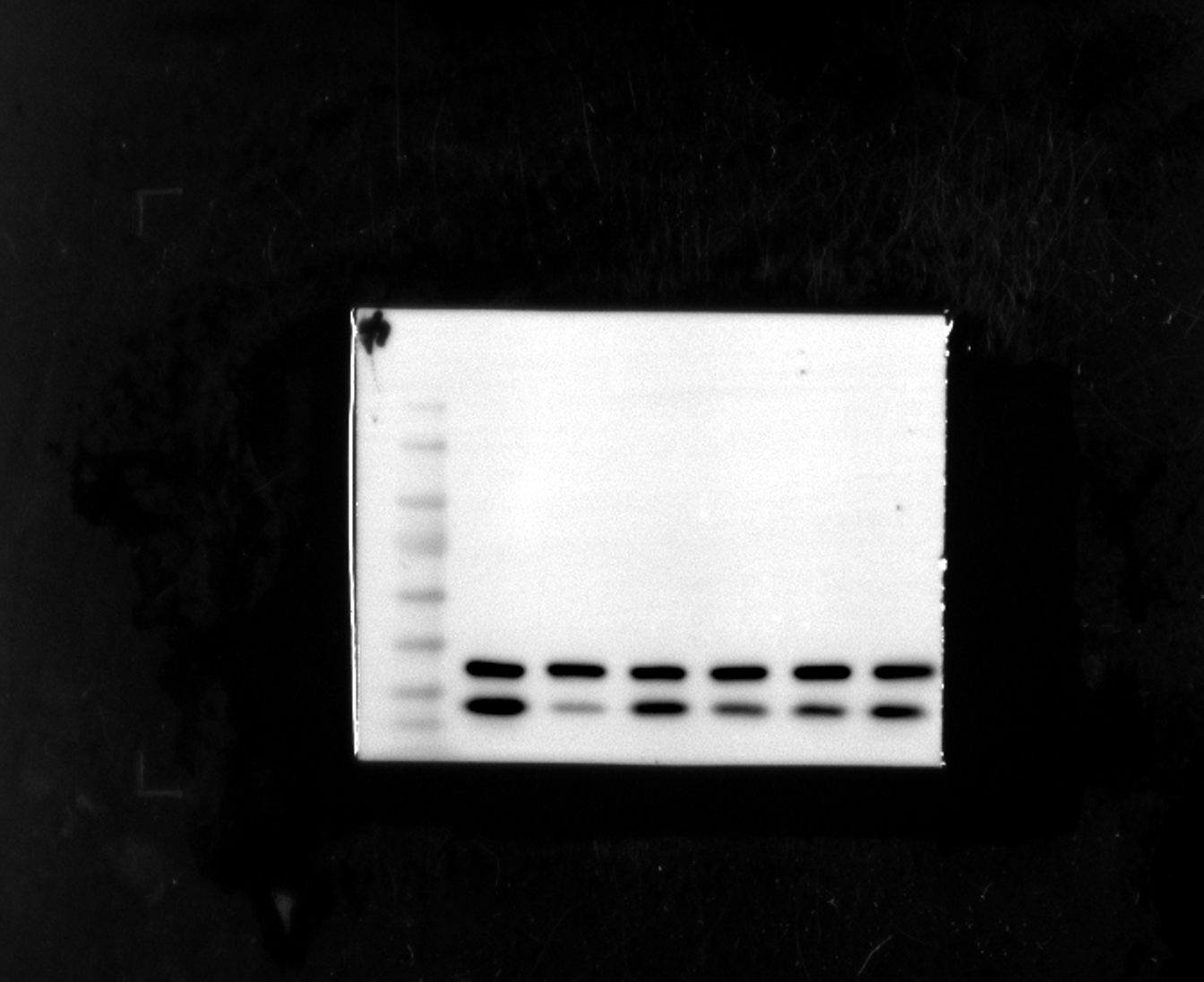

Supplement: Supplementary file 1 [file DataSheet3.zip › 3/UCP2/merged 3s.Tif]

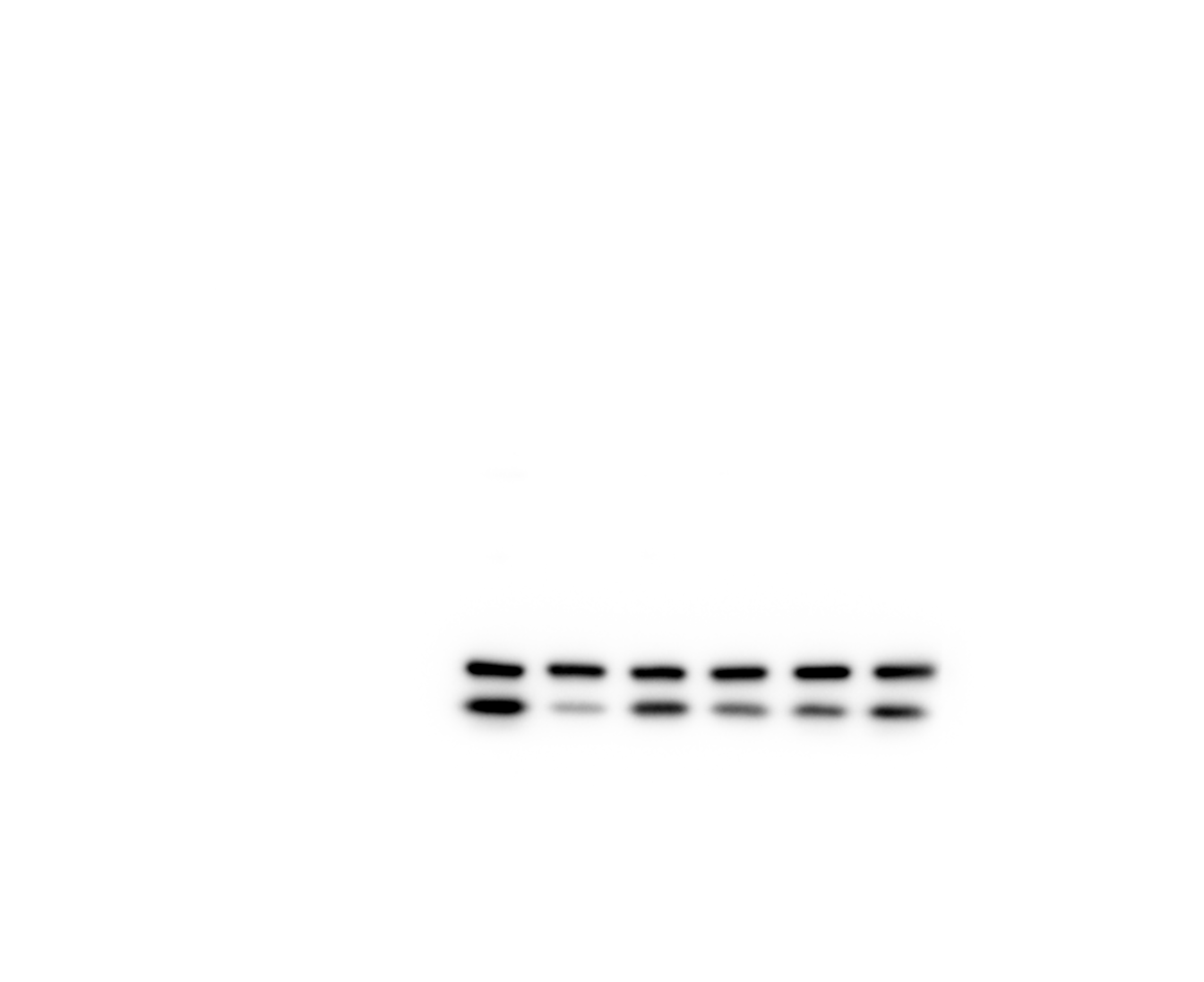

Supplement: Supplementary file 1 [file DataSheet3.zip › 3/UCP2/UCP2 1s.Tif]

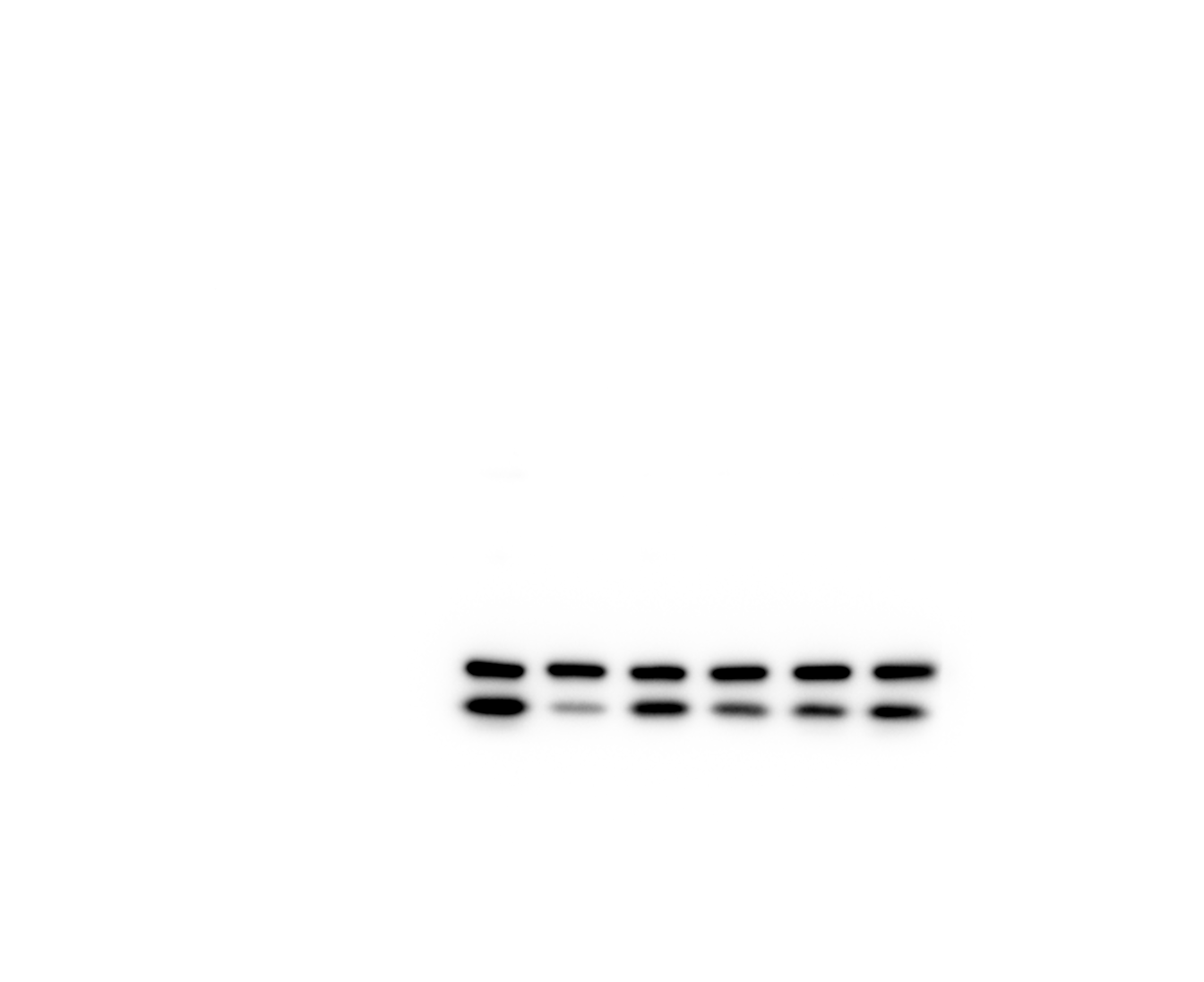

Supplement: Supplementary file 1 [file DataSheet3.zip › 3/UCP2/UCP2 3s.Tif]

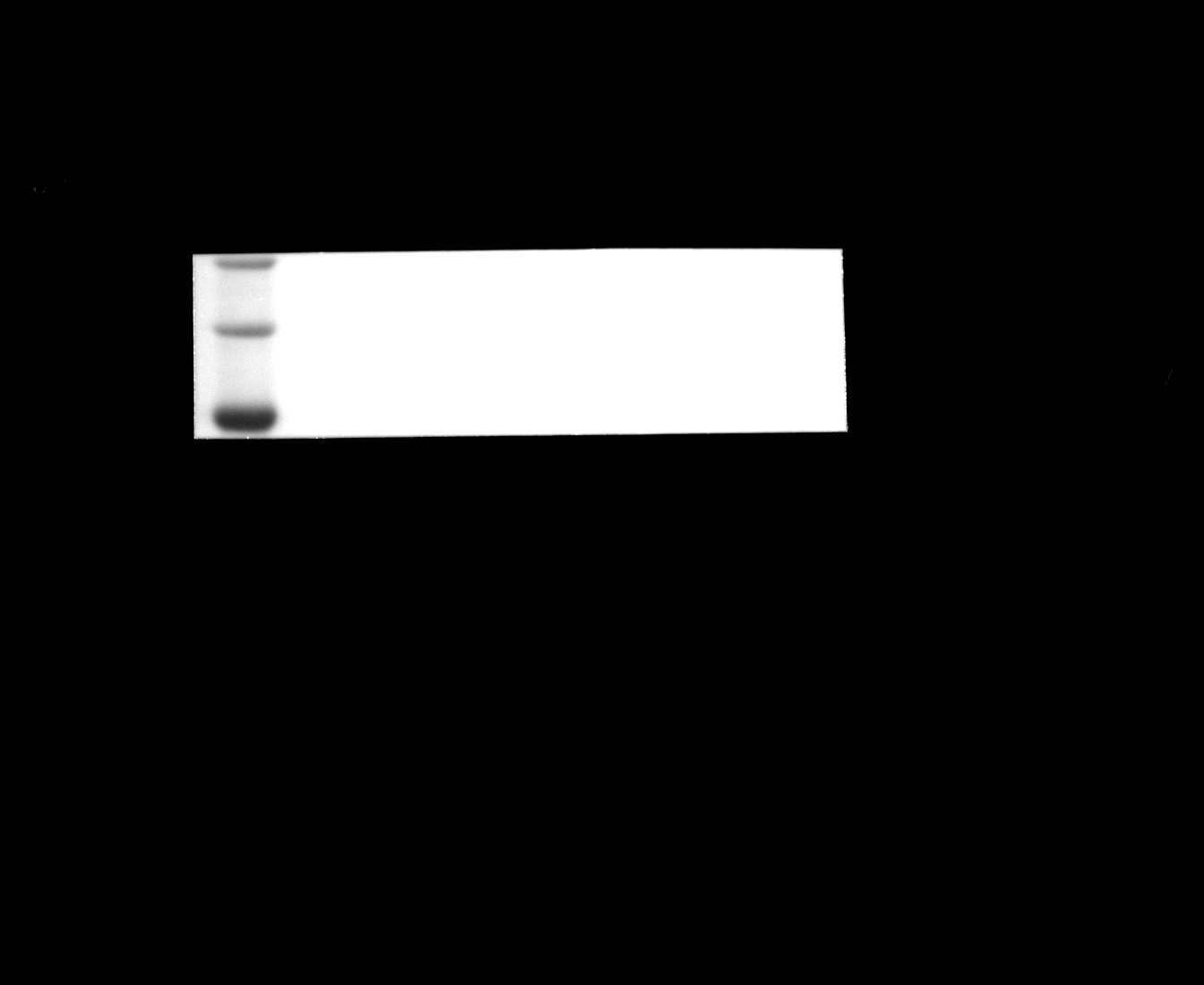

Supplement: Supplementary file 1 [file DataSheet3.zip › WB1/1/1-GAPDH/0.Tif]

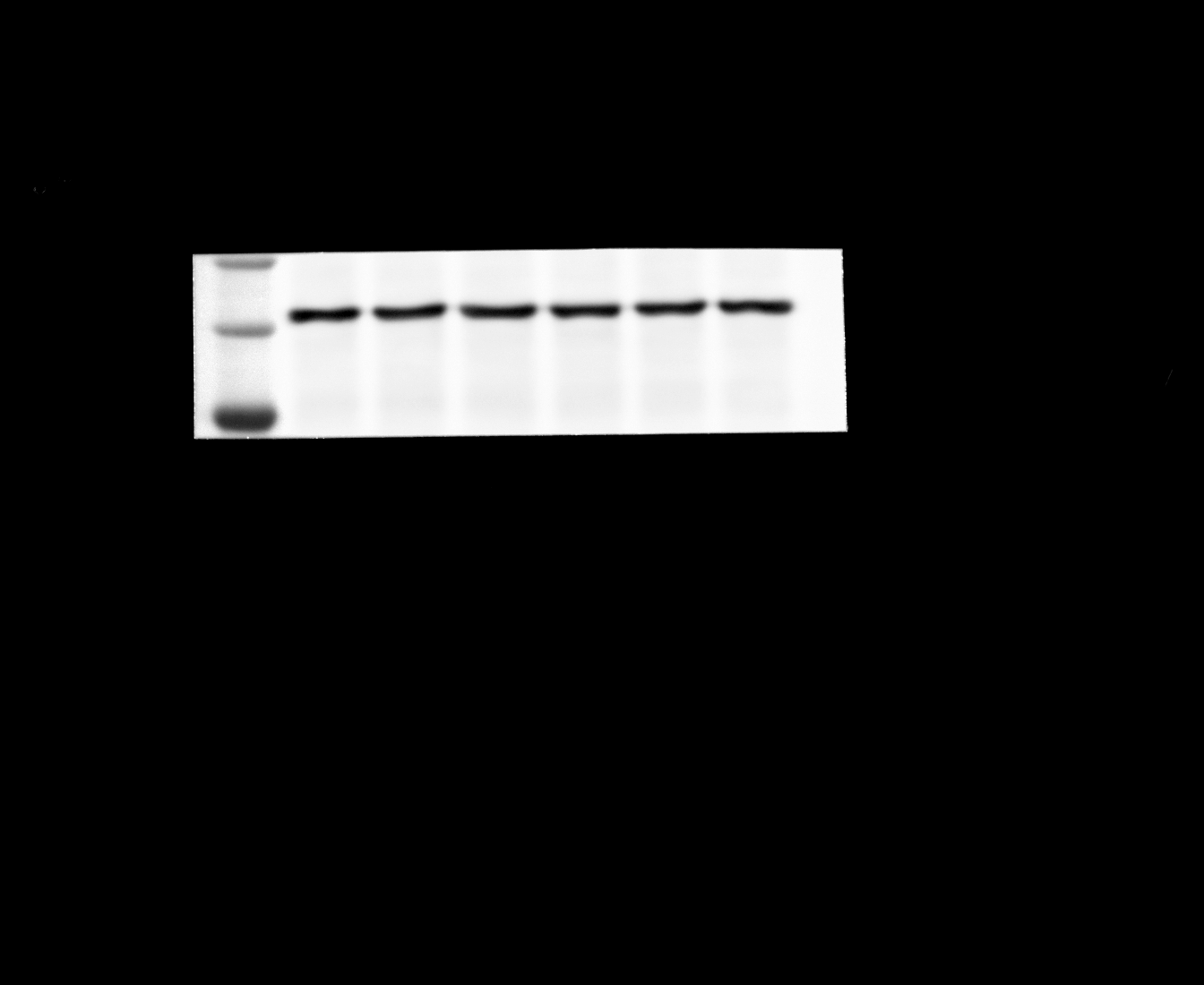

Supplement: Supplementary file 1 [file DataSheet3.zip › WB1/1/1-GAPDH/1.Tif]

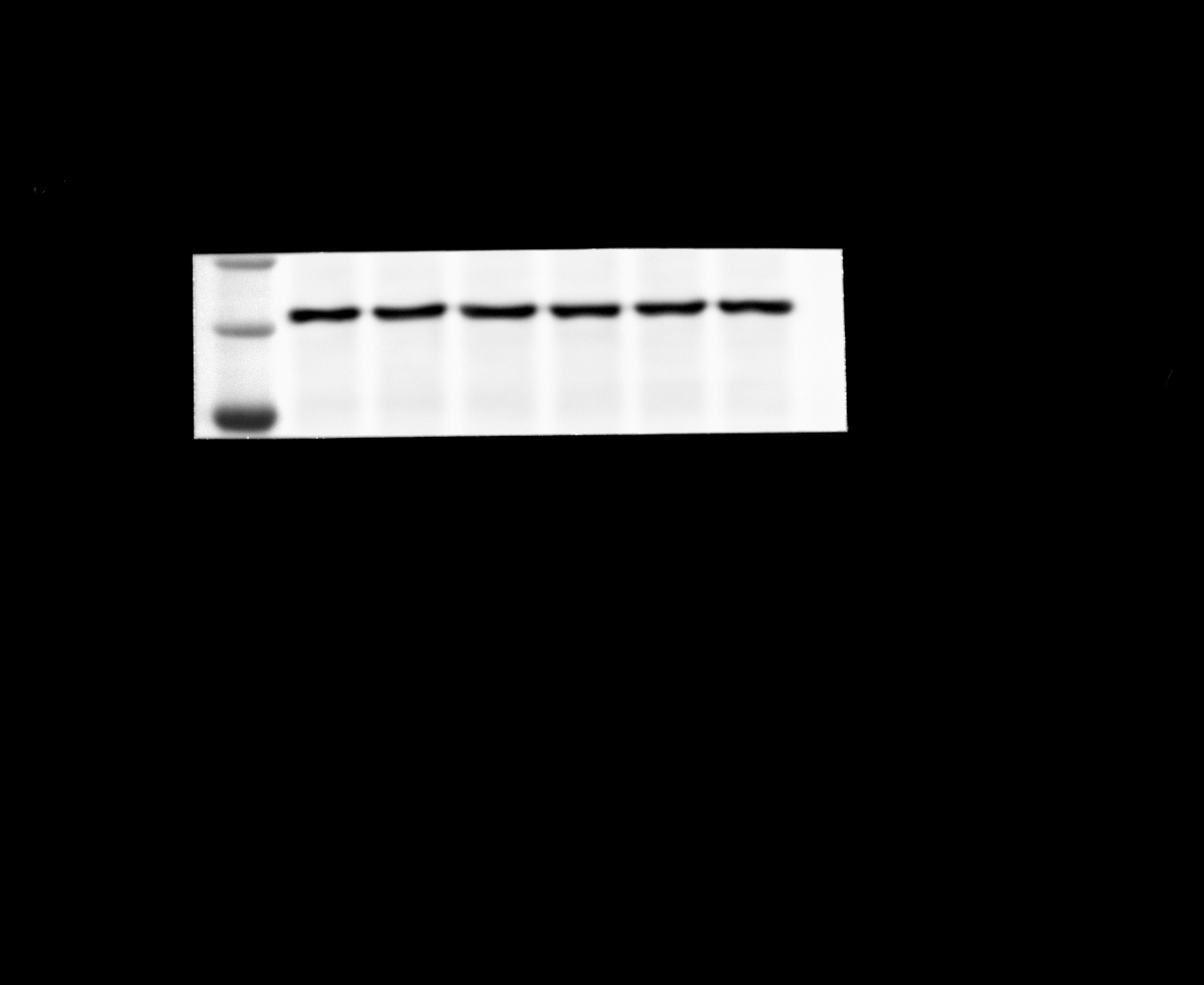

Supplement: Supplementary file 1 [file DataSheet3.zip › WB1/1/1-GAPDH/2.Tif]

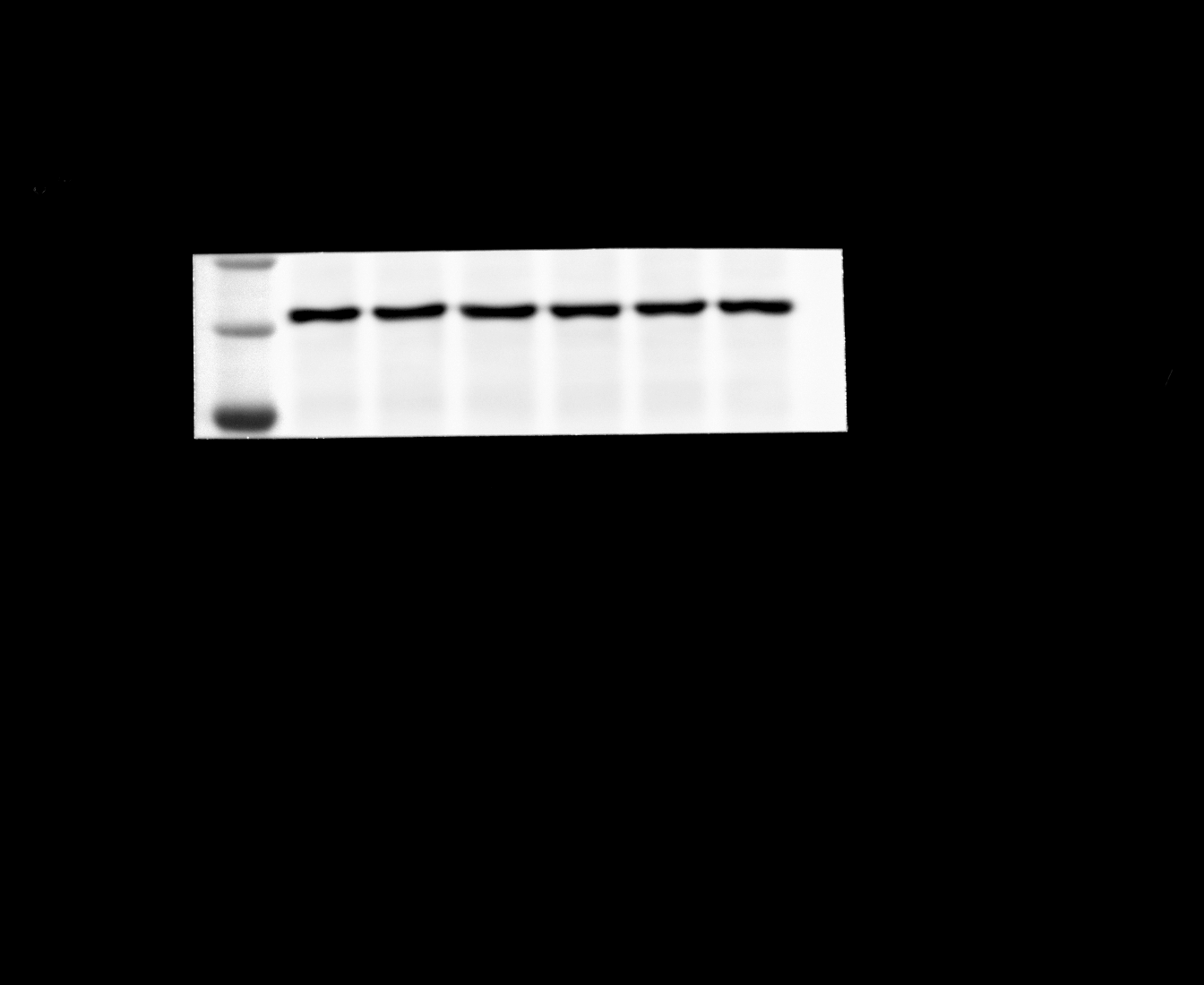

Supplement: Supplementary file 1 [file DataSheet3.zip › WB1/1/1-GAPDH/3.Tif]

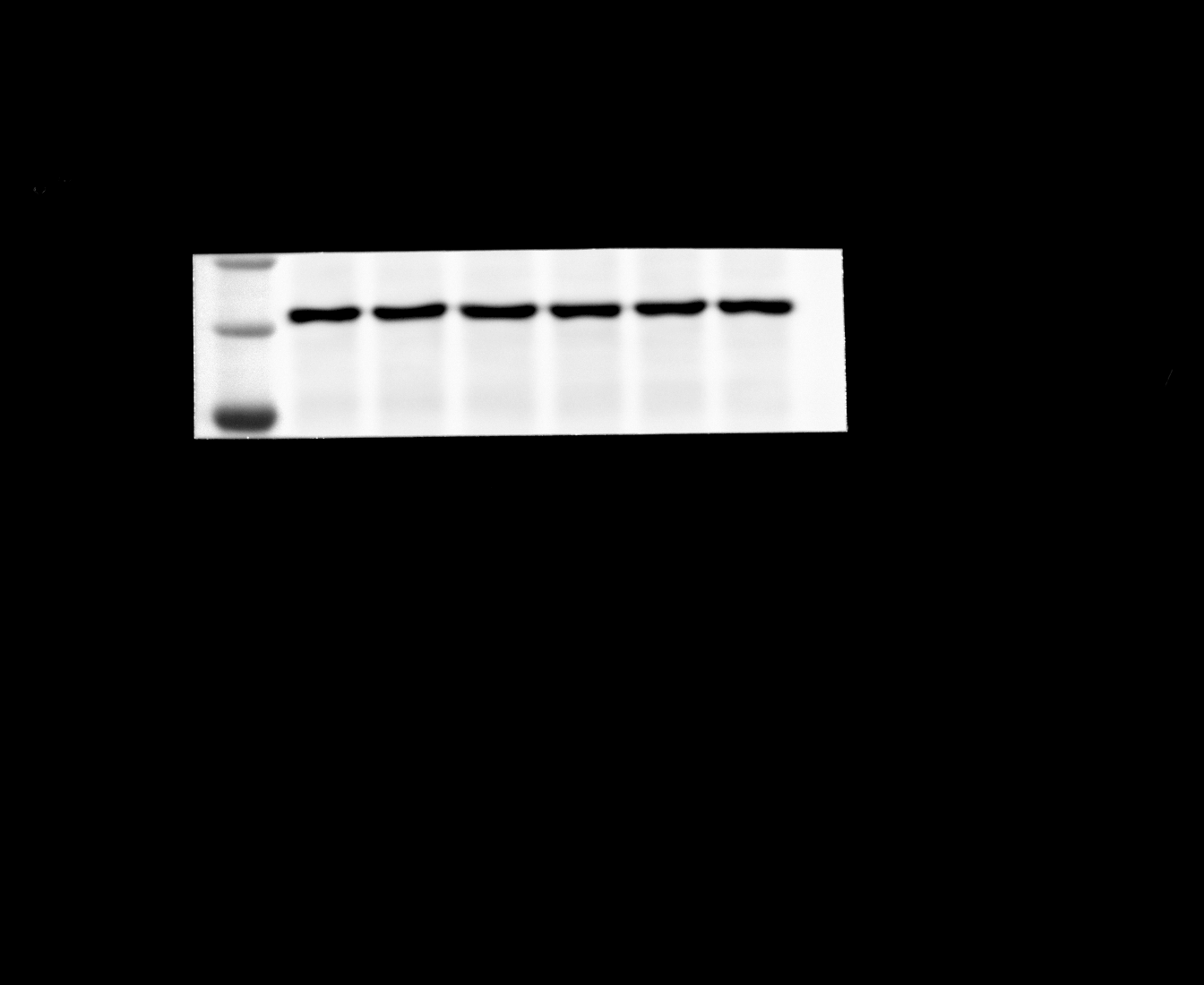

Supplement: Supplementary file 1 [file DataSheet3.zip › WB1/1/1-GAPDH/4.Tif]

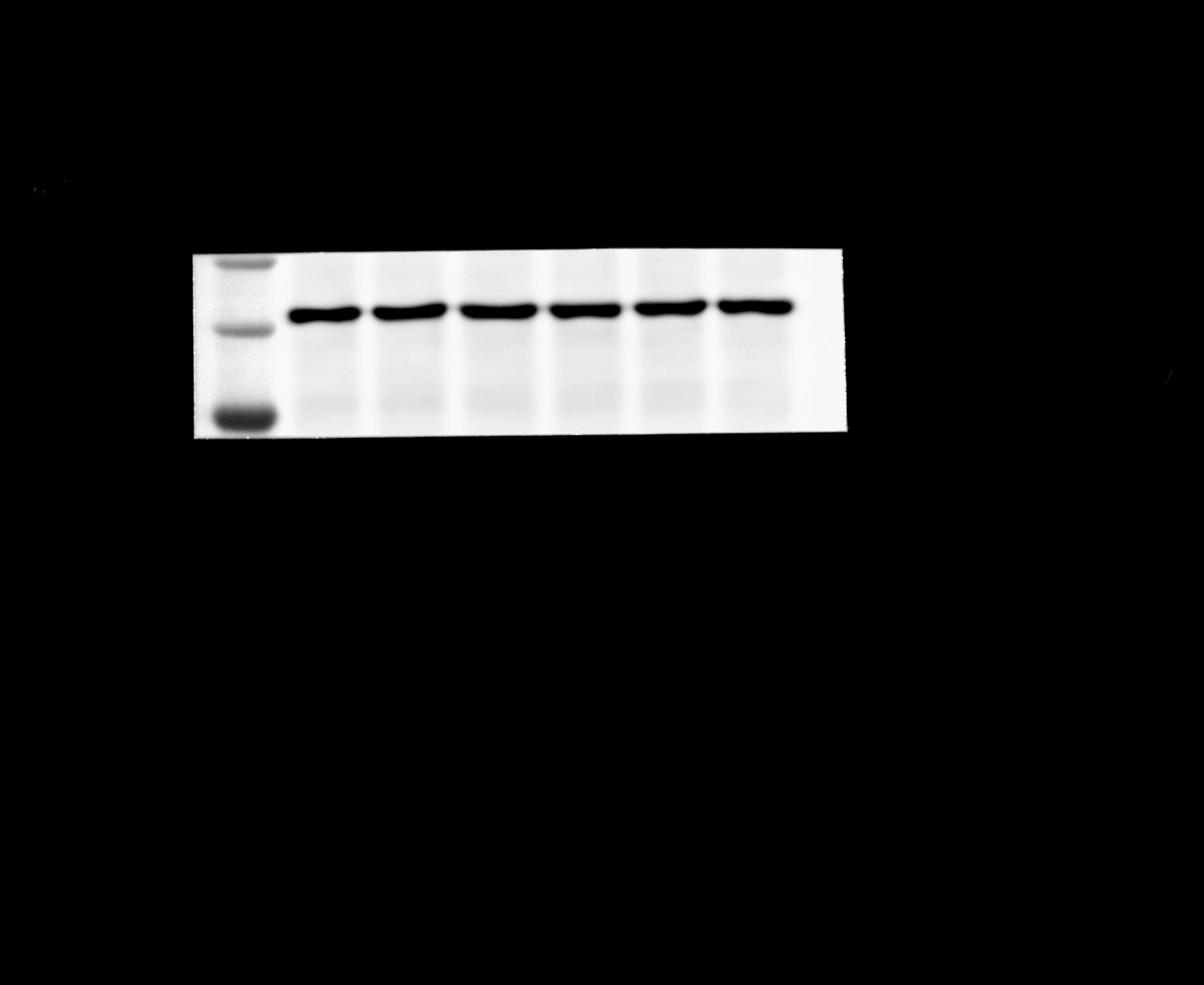

Supplement: Supplementary file 1 [file DataSheet3.zip › WB1/1/1-GAPDH/5.Tif]

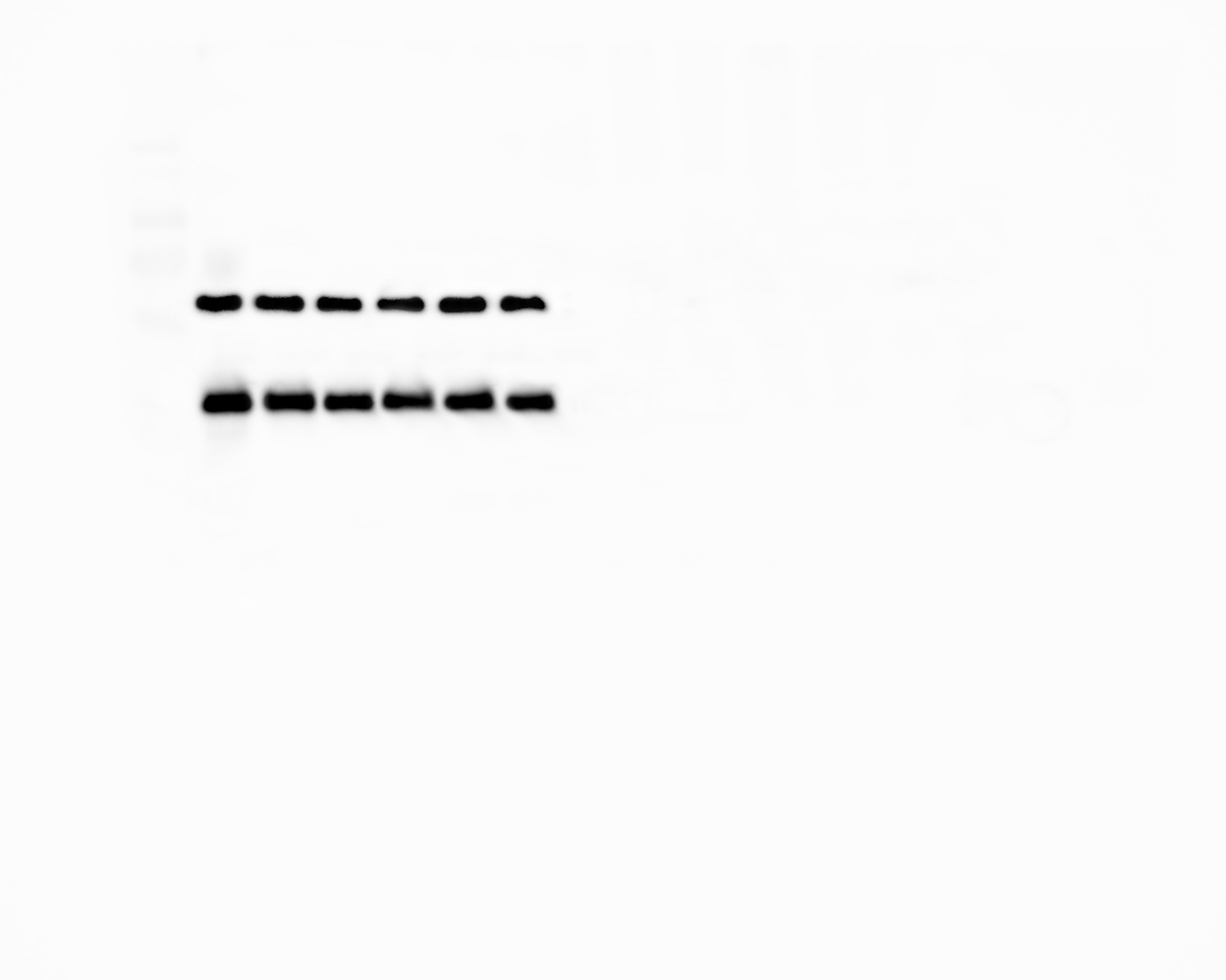

Supplement: Supplementary file 1 [file DataSheet3.zip › WB1/1/2/AMPK (1).tif]

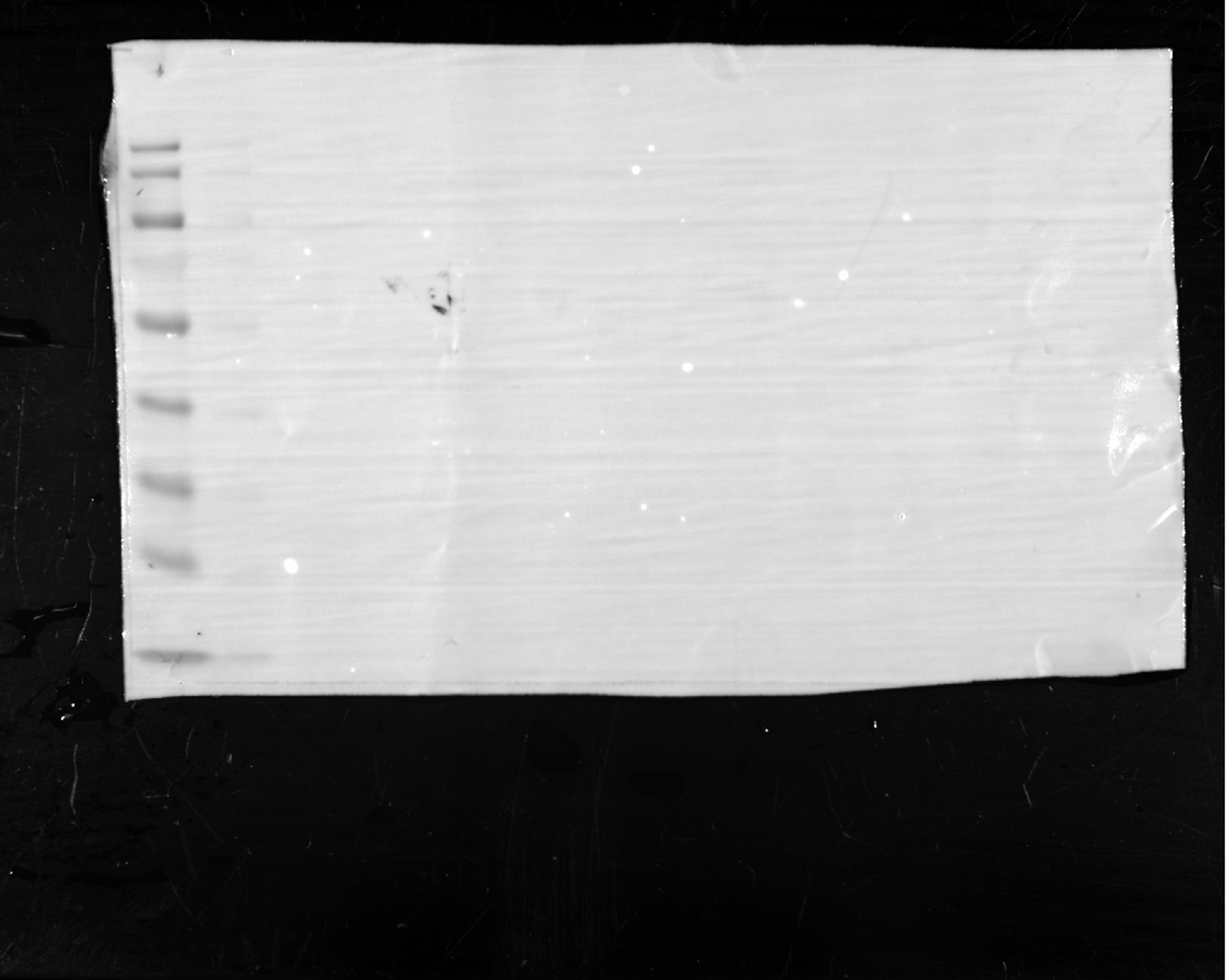

Supplement: Supplementary file 1 [file DataSheet3.zip › WB1/1/2/AMPK (2).tif]

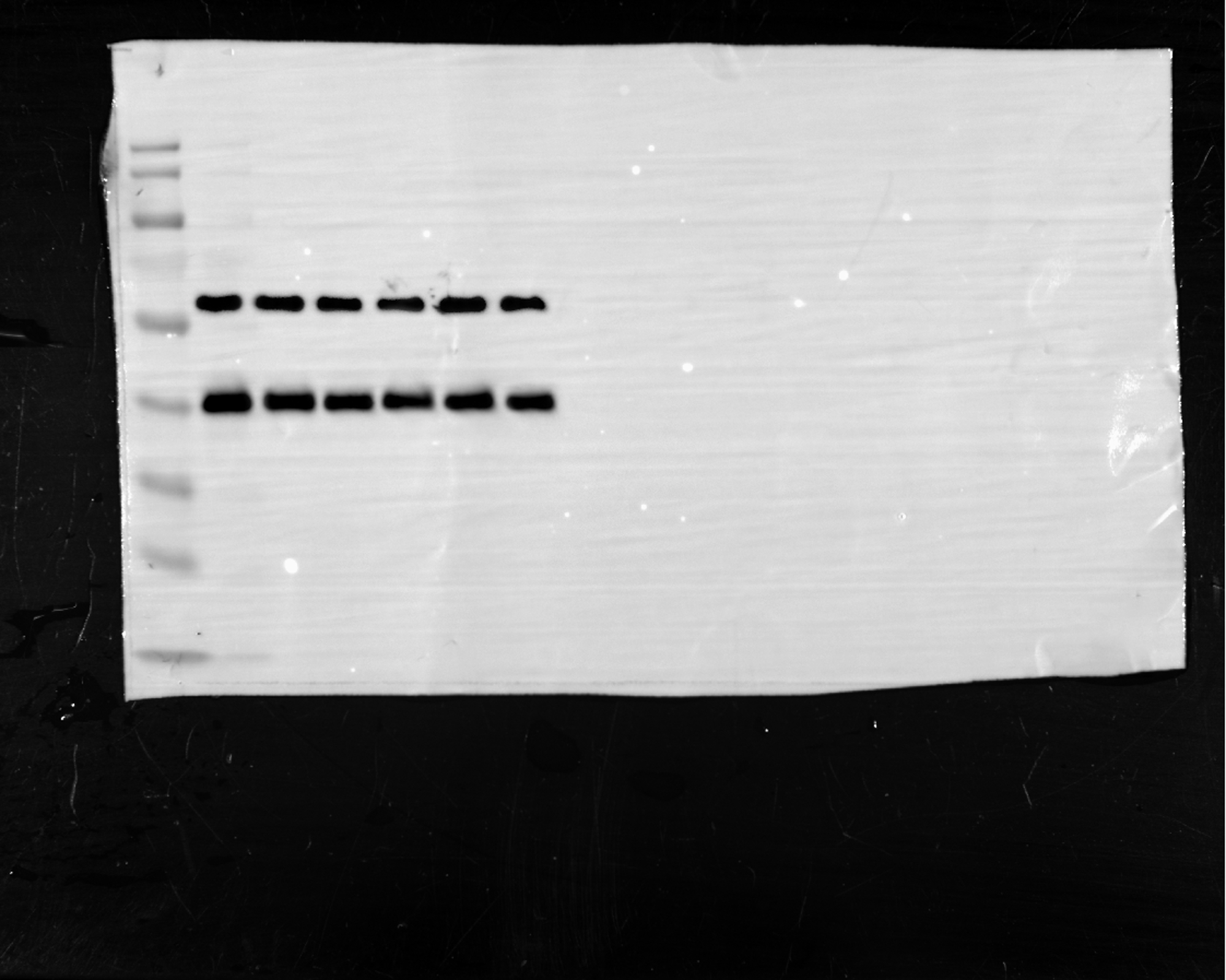

Supplement: Supplementary file 1 [file DataSheet3.zip › WB1/1/2/AMPK (3).tif]

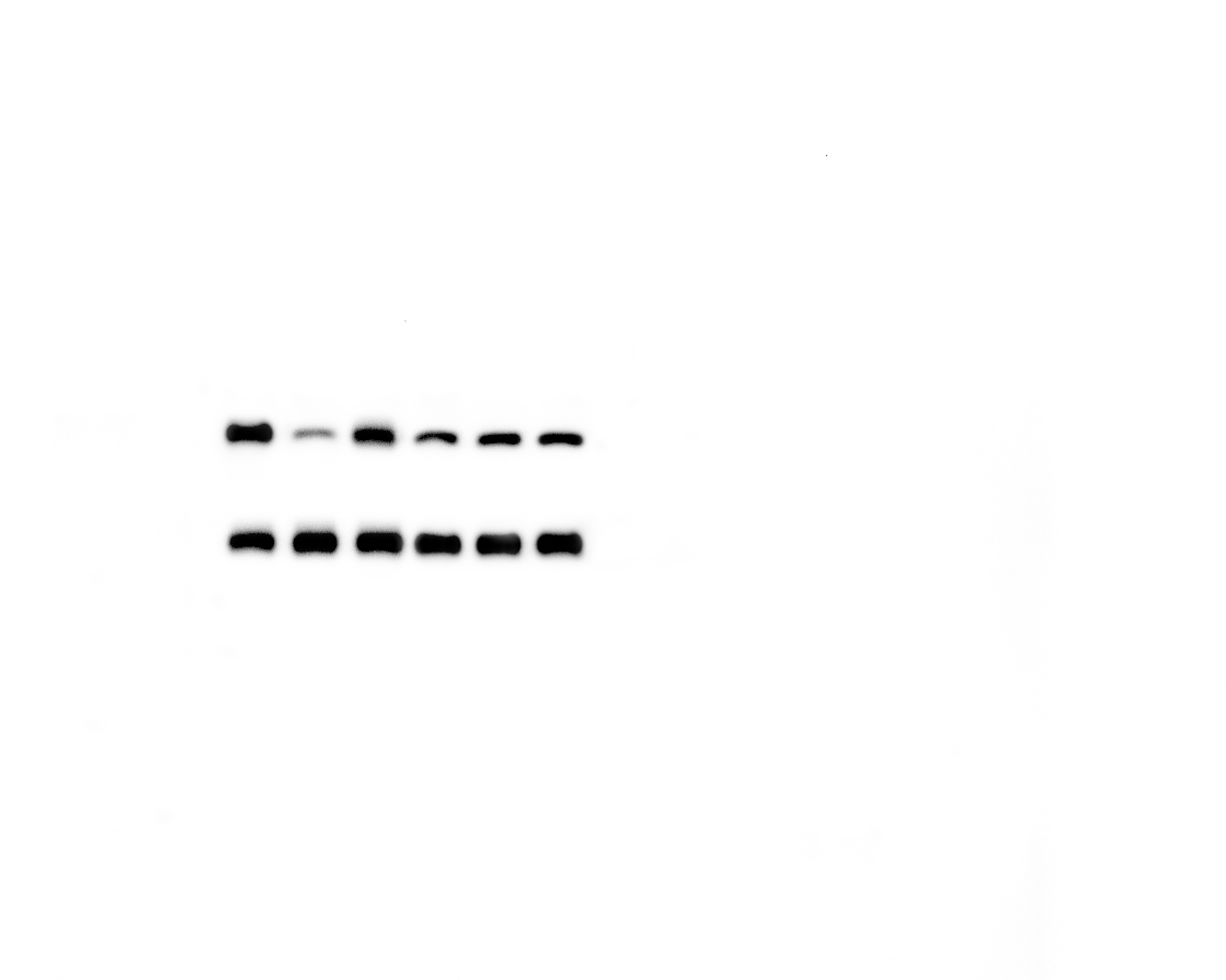

Supplement: Supplementary file 1 [file DataSheet3.zip › WB1/1/2/p-AMPK (1).tif]

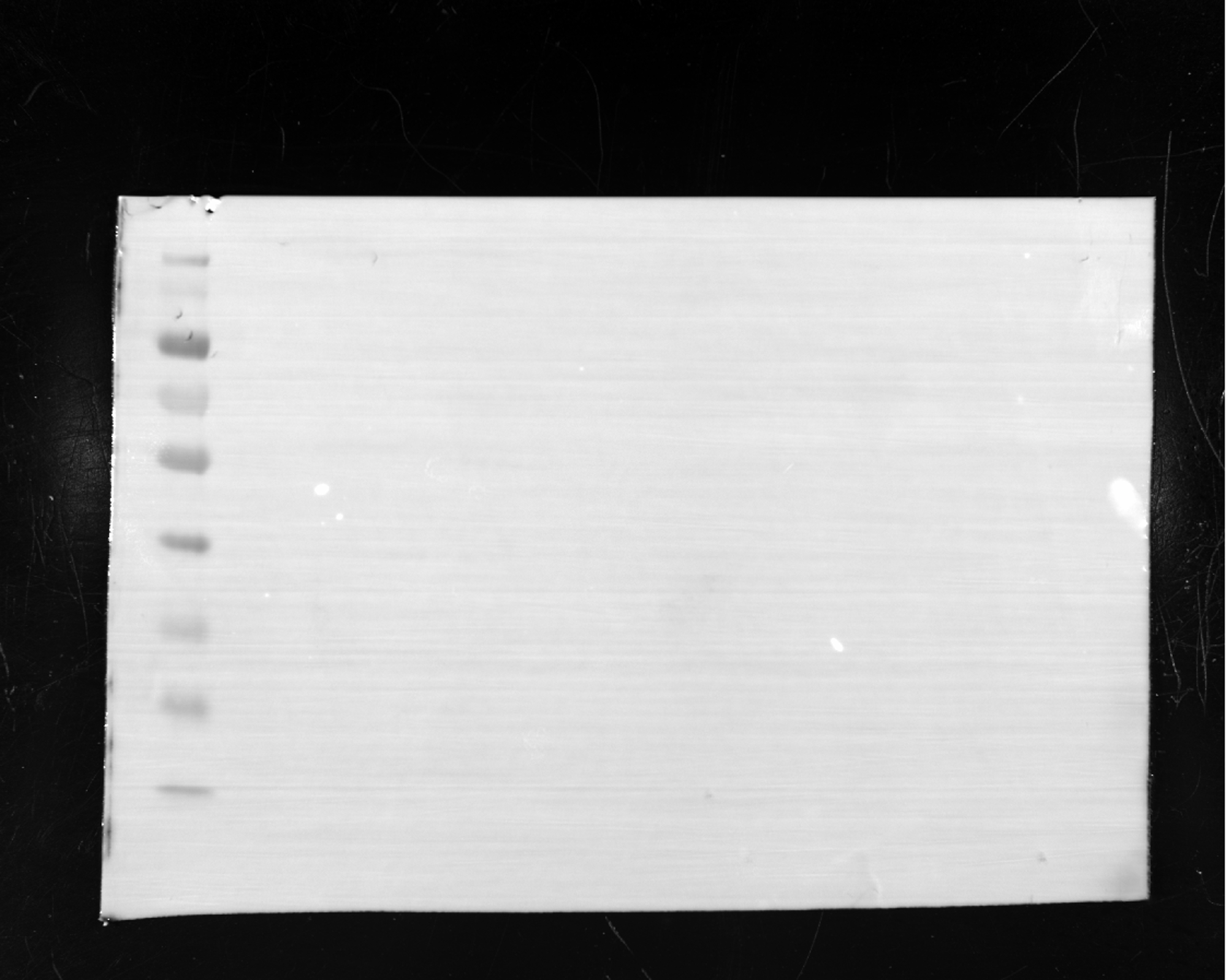

Supplement: Supplementary file 1 [file DataSheet3.zip › WB1/1/2/p-AMPK (2).tif]

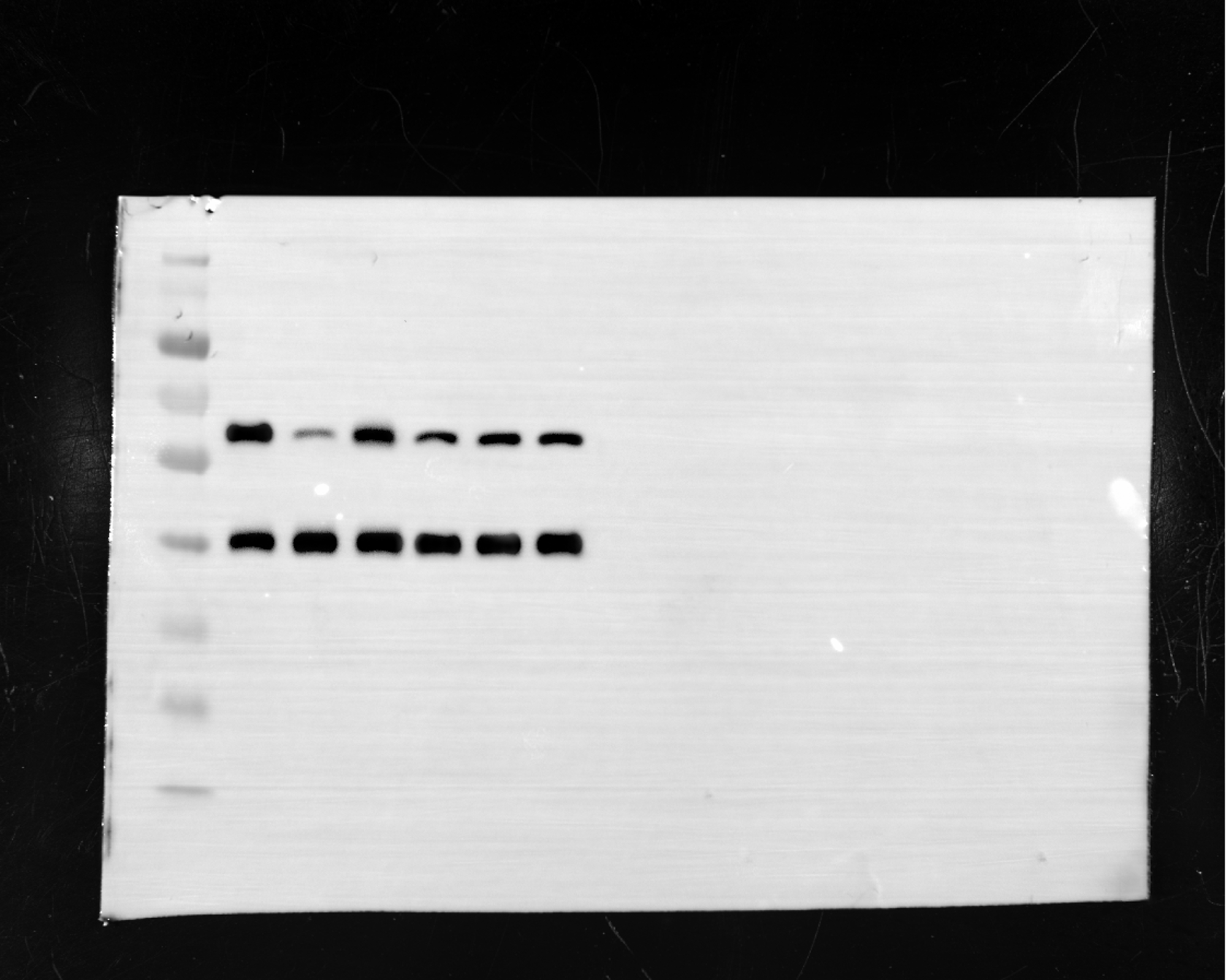

Supplement: Supplementary file 1 [file DataSheet3.zip › WB1/1/2/p-AMPK (3).tif]

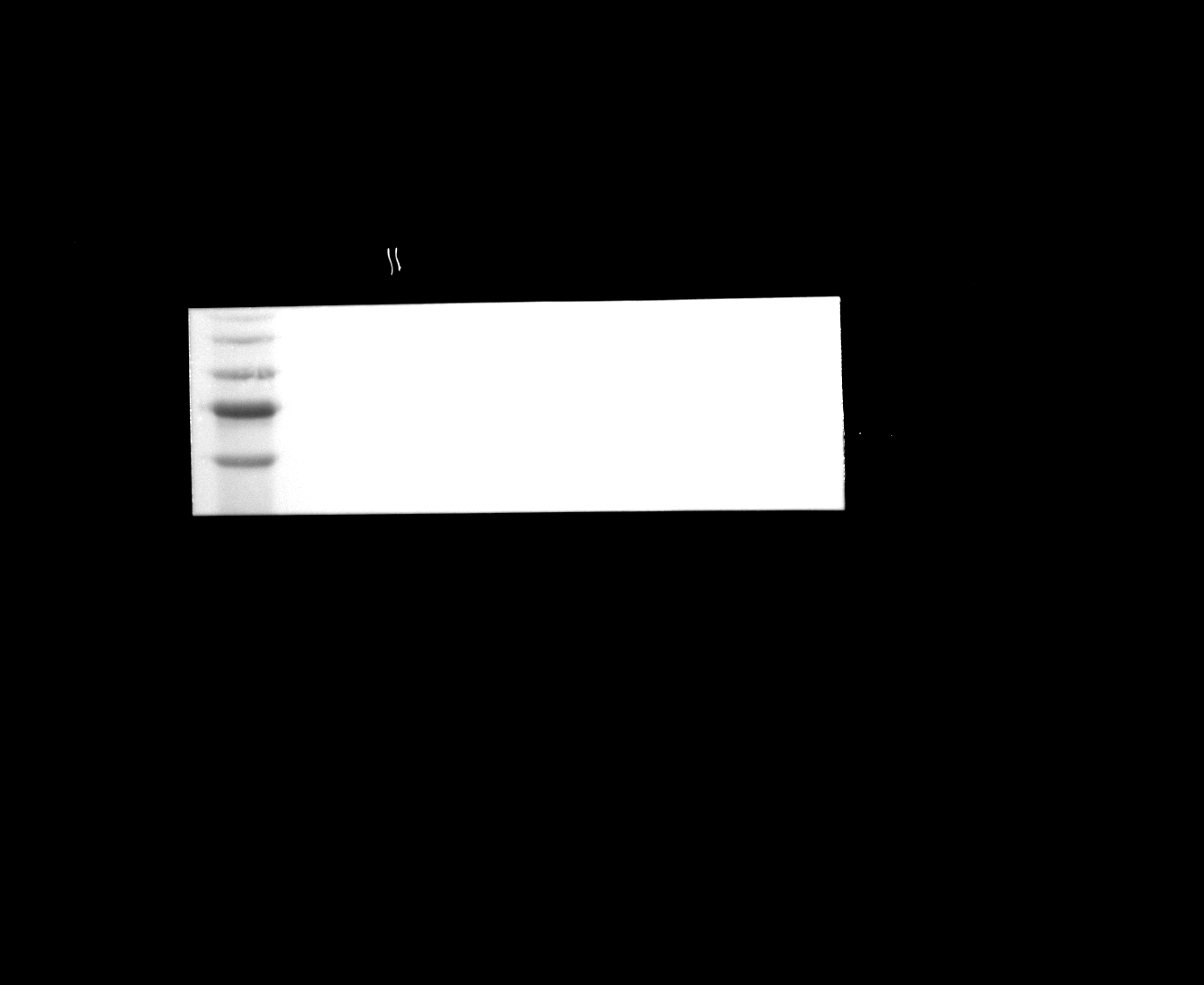

Supplement: Supplementary file 1 [file DataSheet3.zip › WB1/1/AMPK/0.Tif]

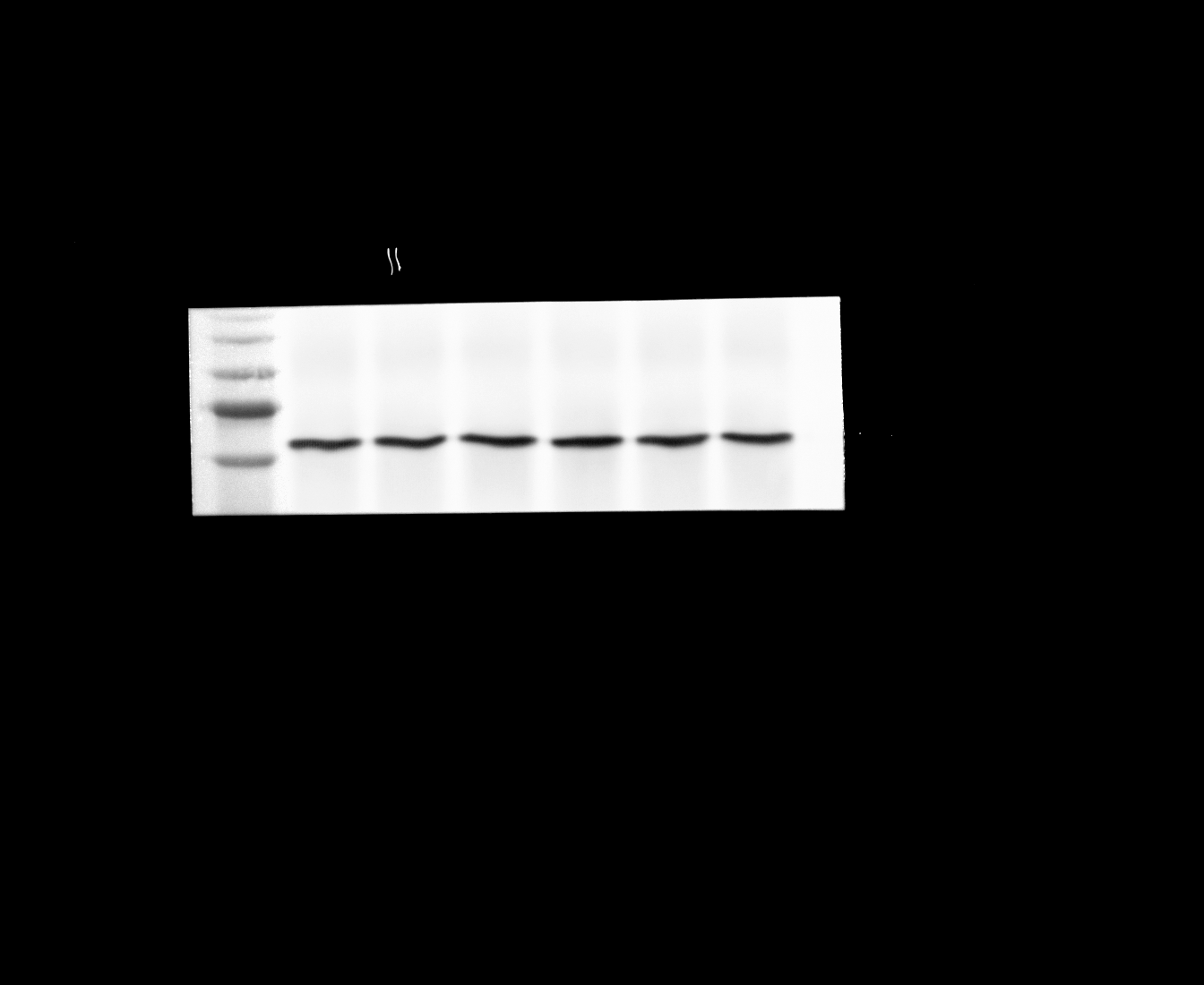

Supplement: Supplementary file 1 [file DataSheet3.zip › WB1/1/AMPK/1.Tif]

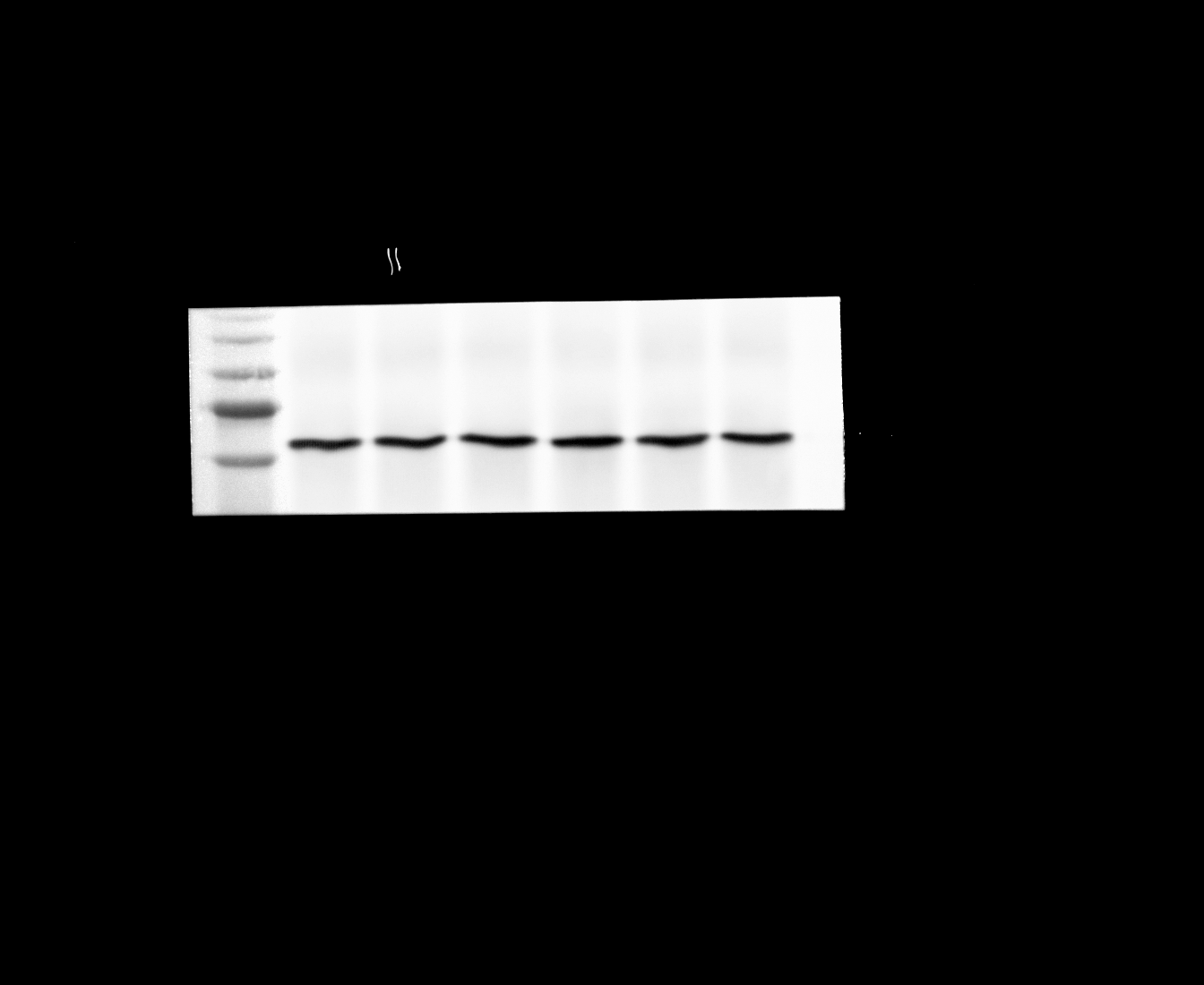

Supplement: Supplementary file 1 [file DataSheet3.zip › WB1/1/AMPK/2.Tif]

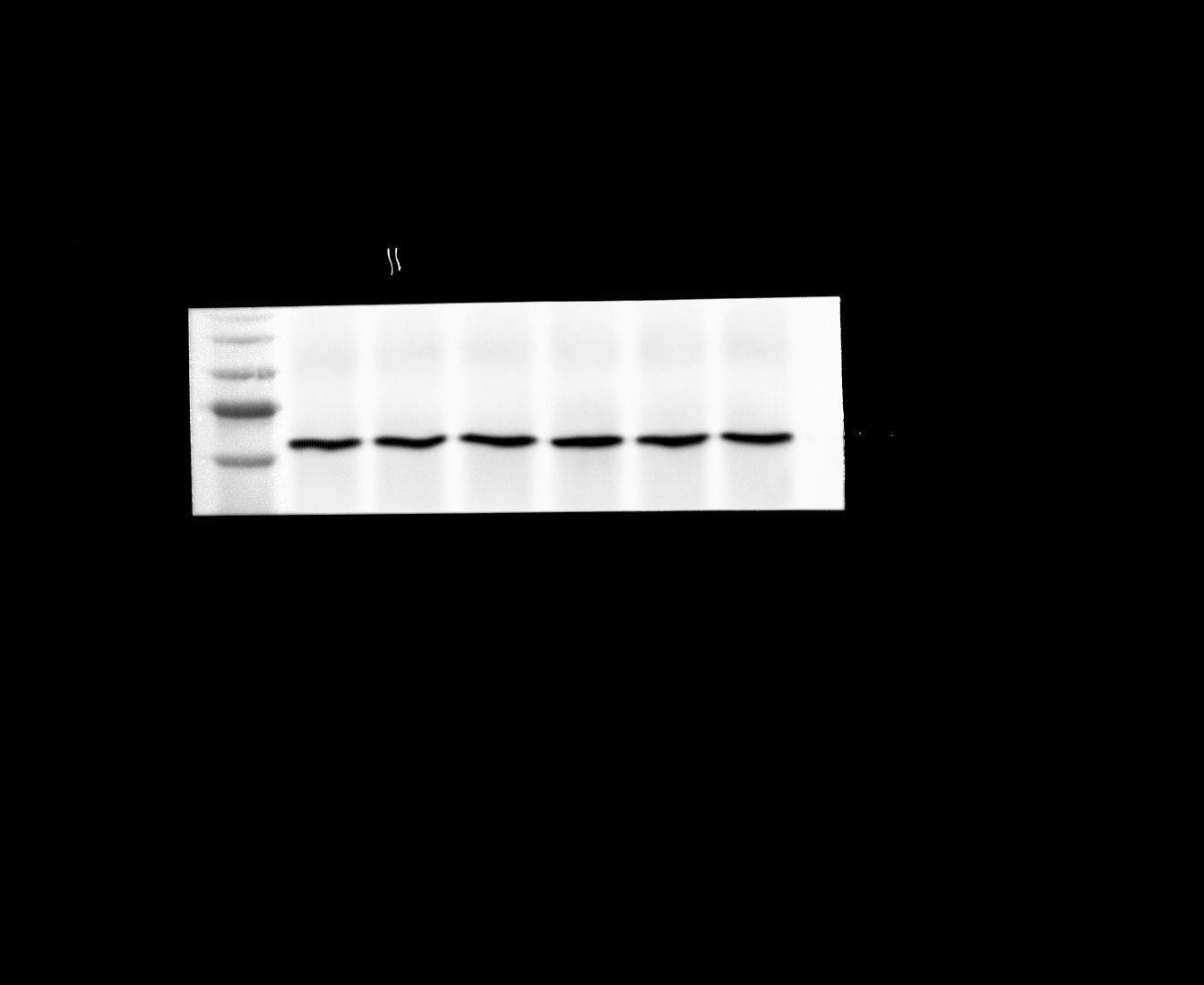

Supplement: Supplementary file 1 [file DataSheet3.zip › WB1/1/AMPK/3.Tif]

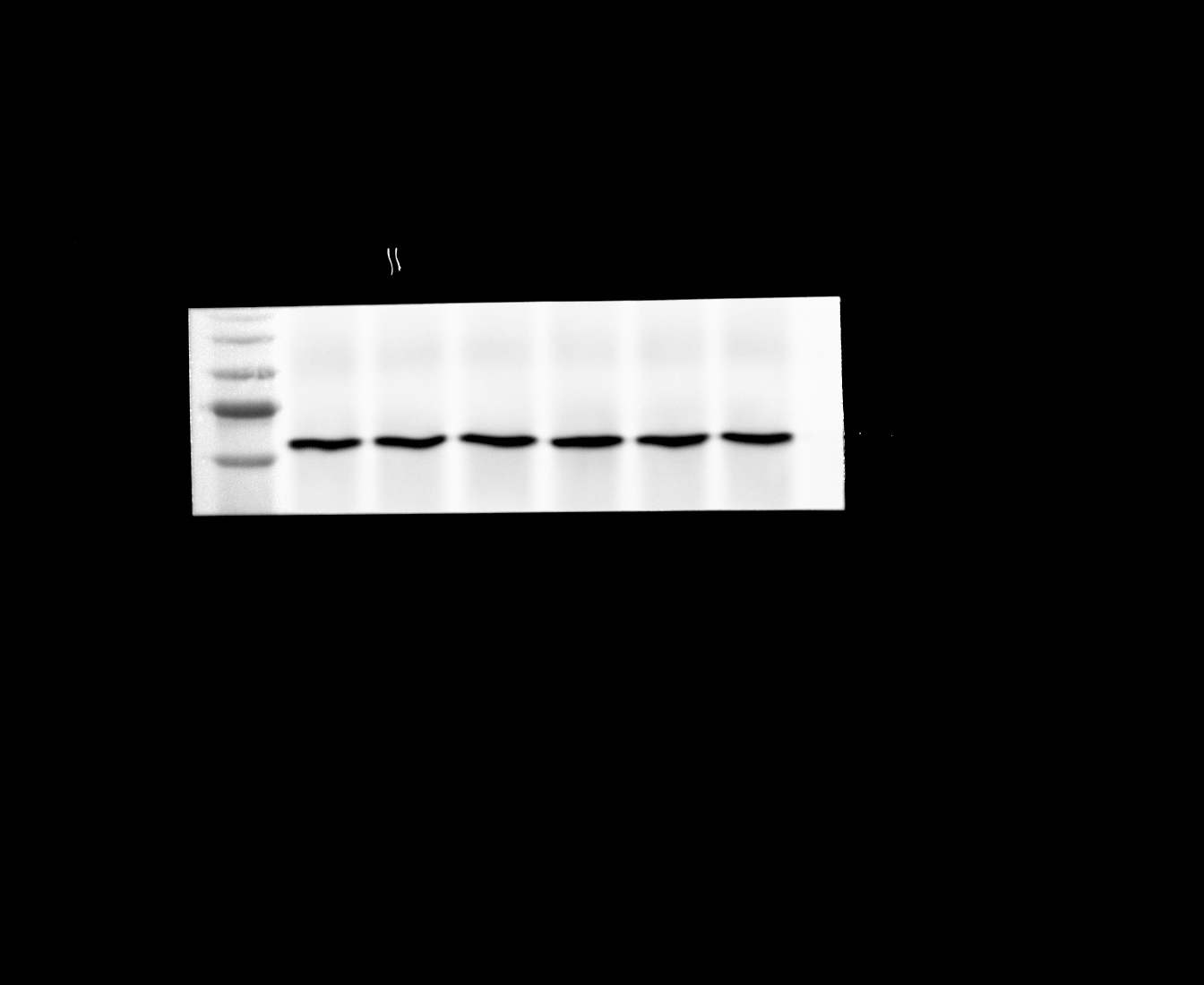

Supplement: Supplementary file 1 [file DataSheet3.zip › WB1/1/AMPK/4.Tif]

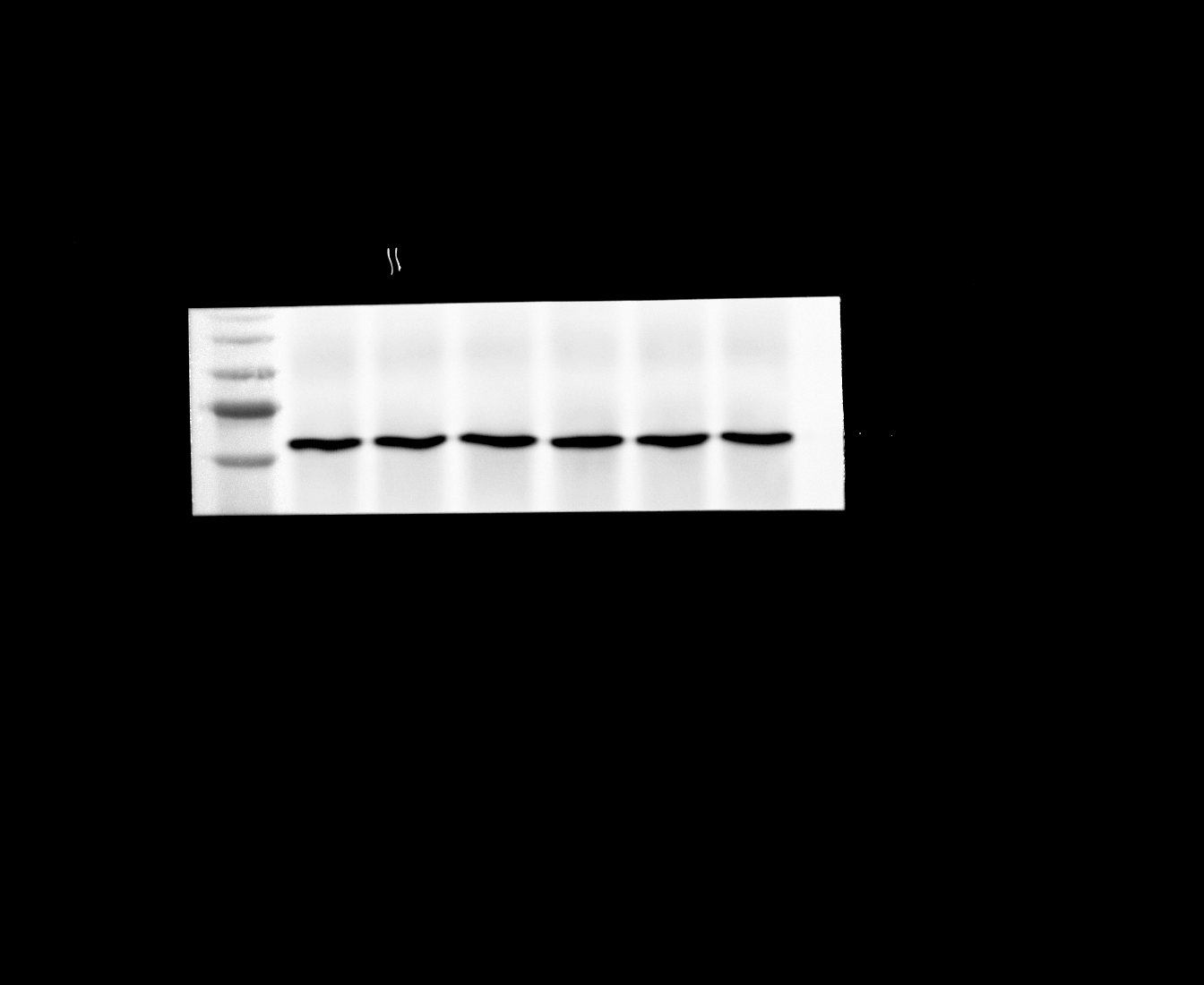

Supplement: Supplementary file 1 [file DataSheet3.zip › WB1/1/AMPK/5.Tif]

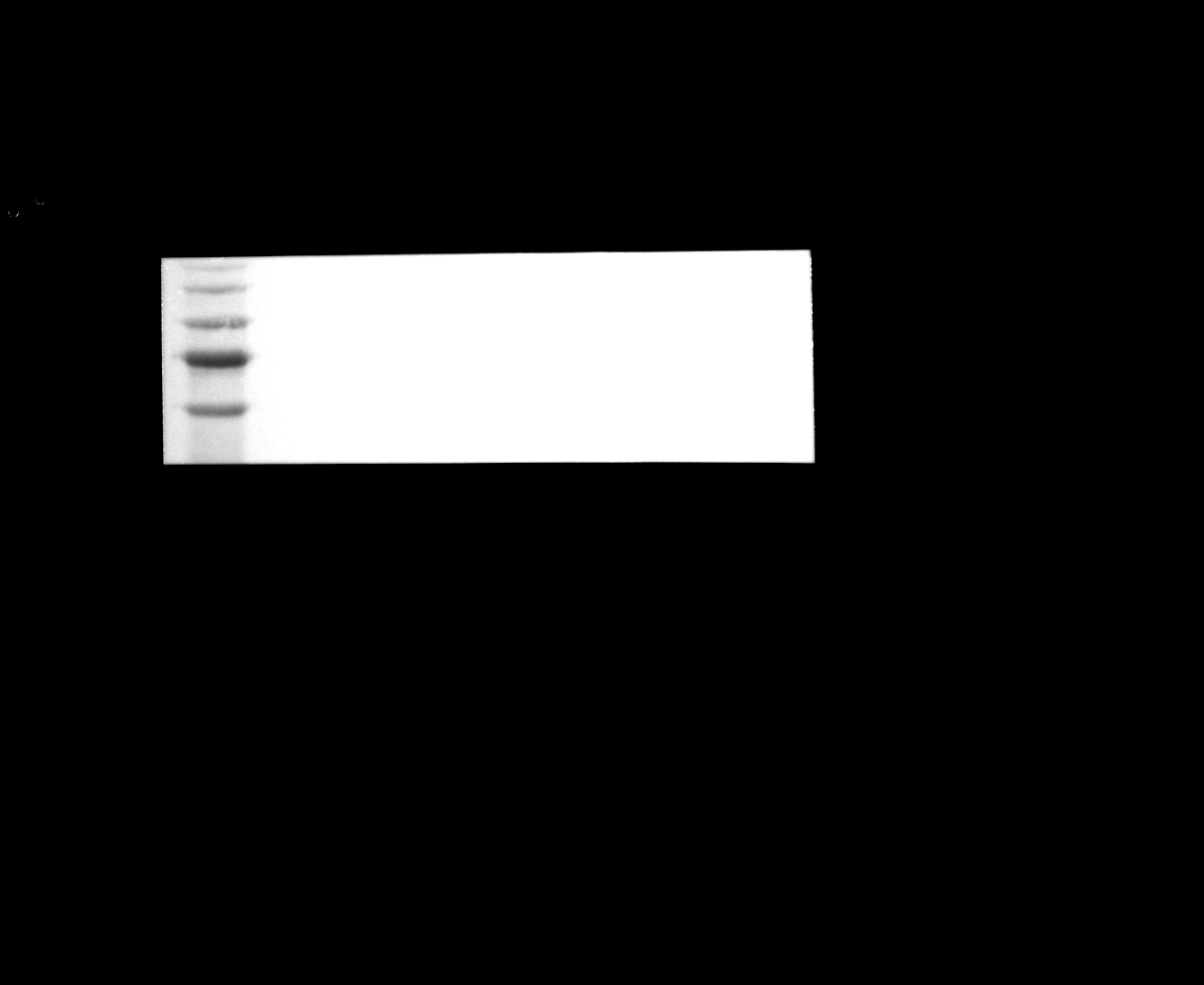

Supplement: Supplementary file 1 [file DataSheet3.zip › WB1/1/p-ampk/0.Tif]

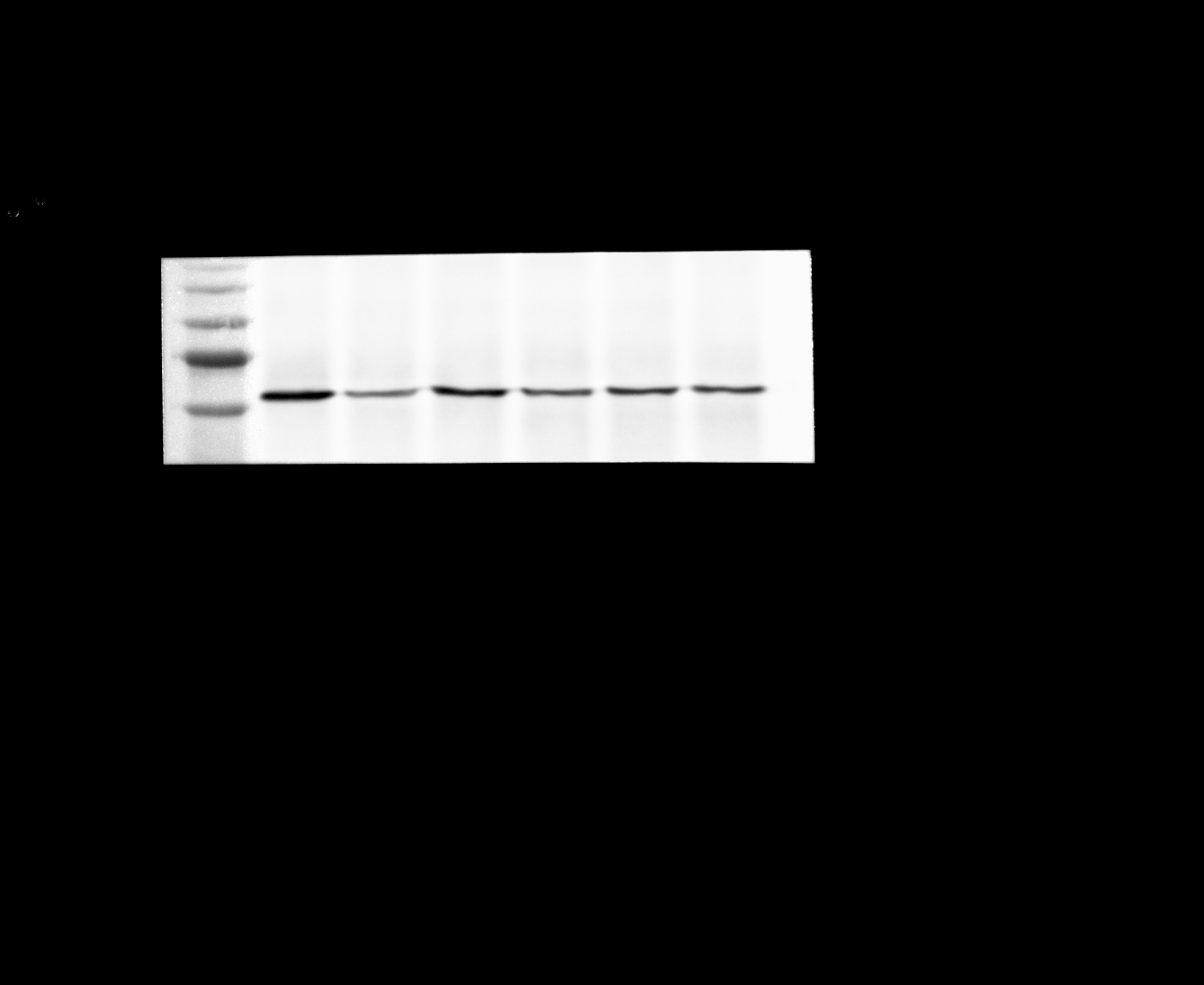

Supplement: Supplementary file 1 [file DataSheet3.zip › WB1/1/p-ampk/1.Tif]

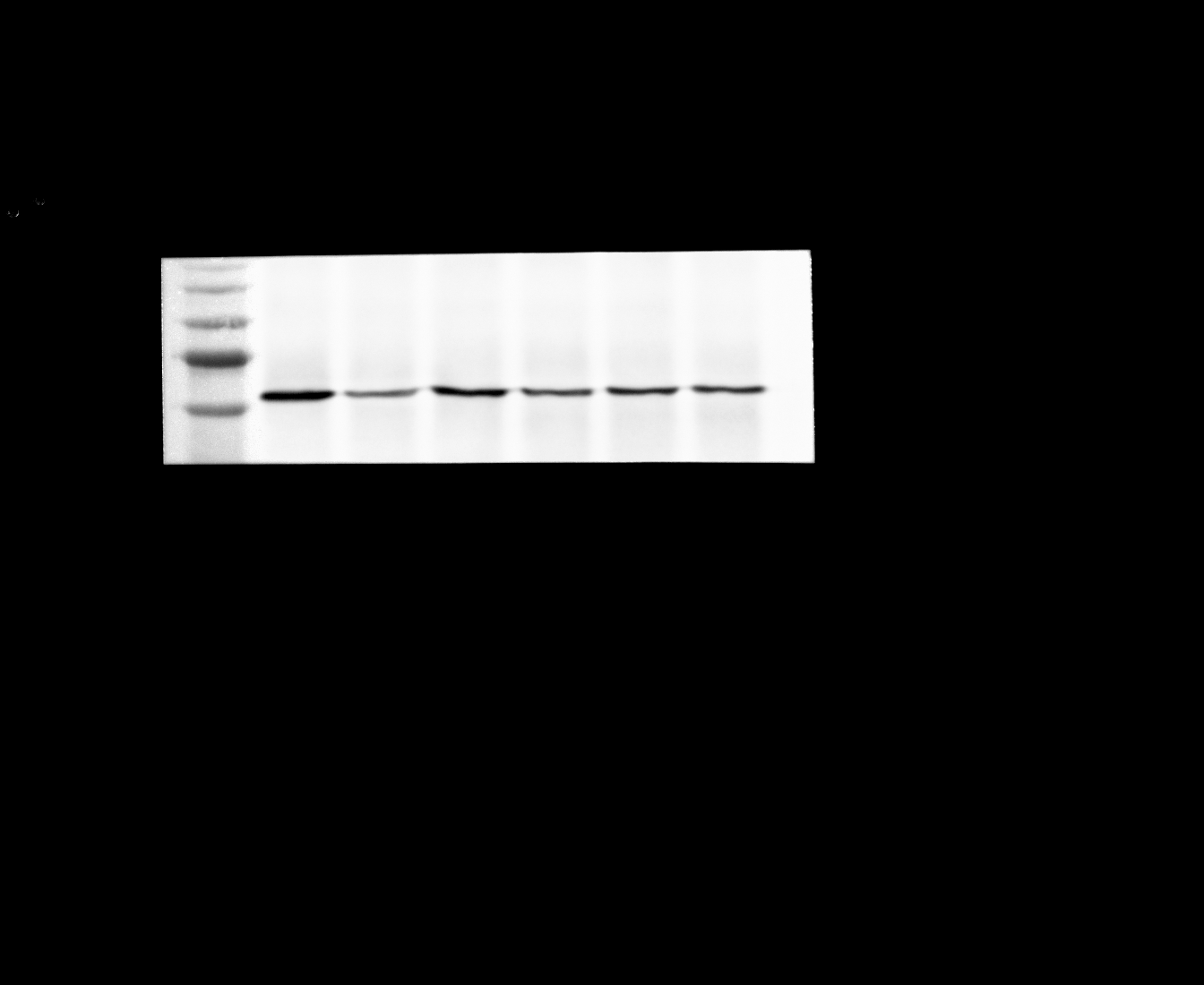

Supplement: Supplementary file 1 [file DataSheet3.zip › WB1/1/p-ampk/2.Tif]

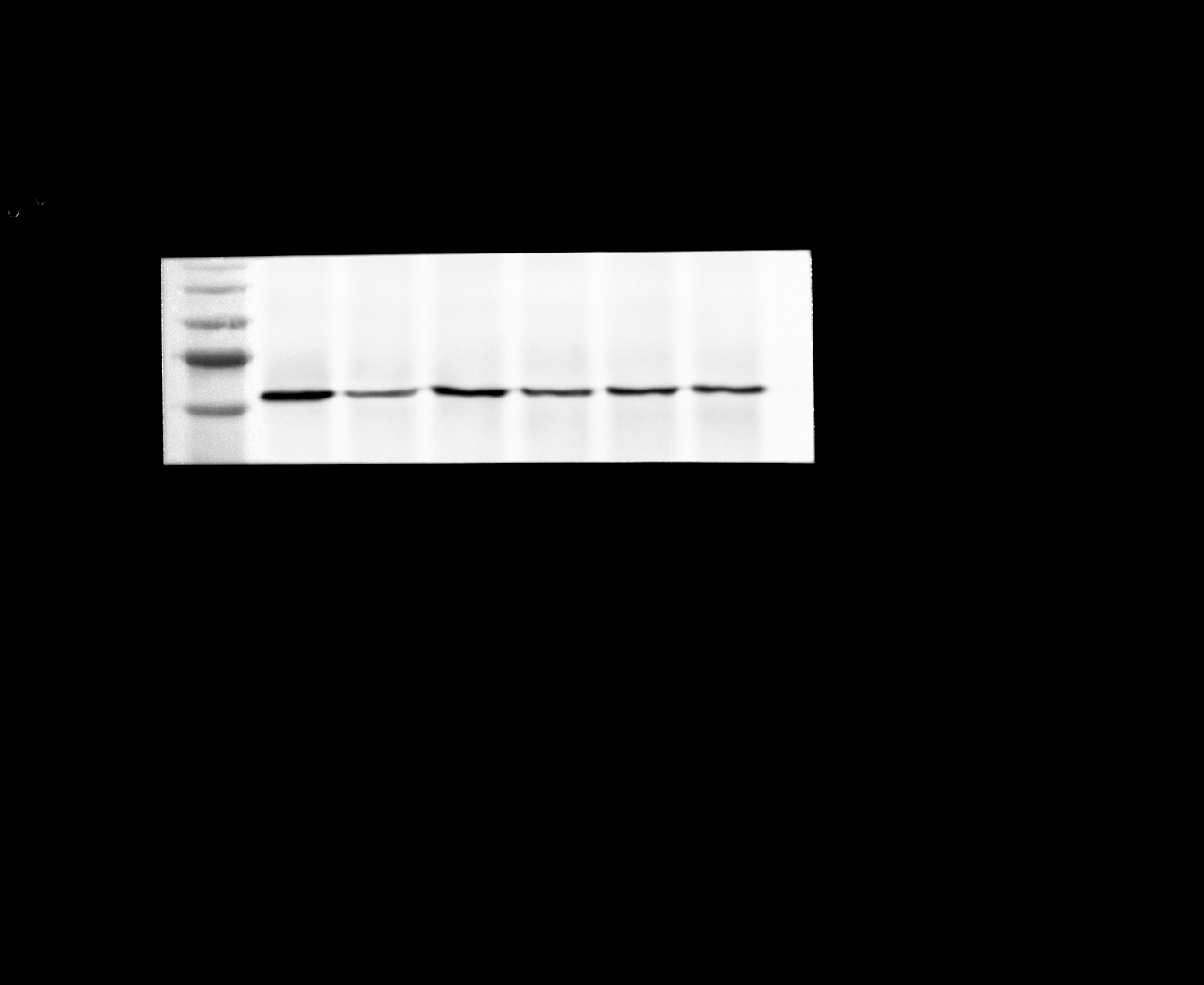

Supplement: Supplementary file 1 [file DataSheet3.zip › WB1/1/p-ampk/3.Tif]

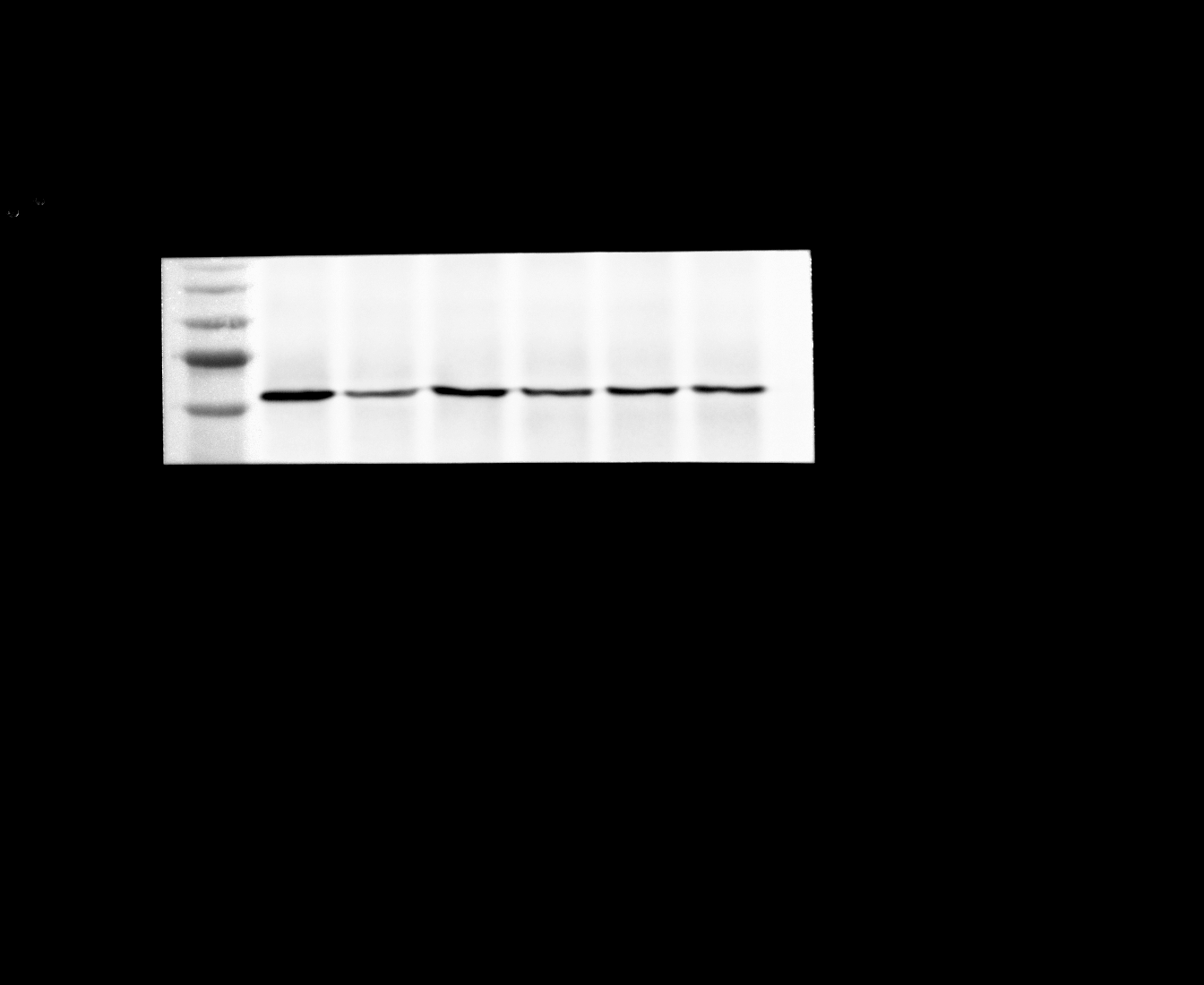

Supplement: Supplementary file 1 [file DataSheet3.zip › WB1/1/p-ampk/4.Tif]

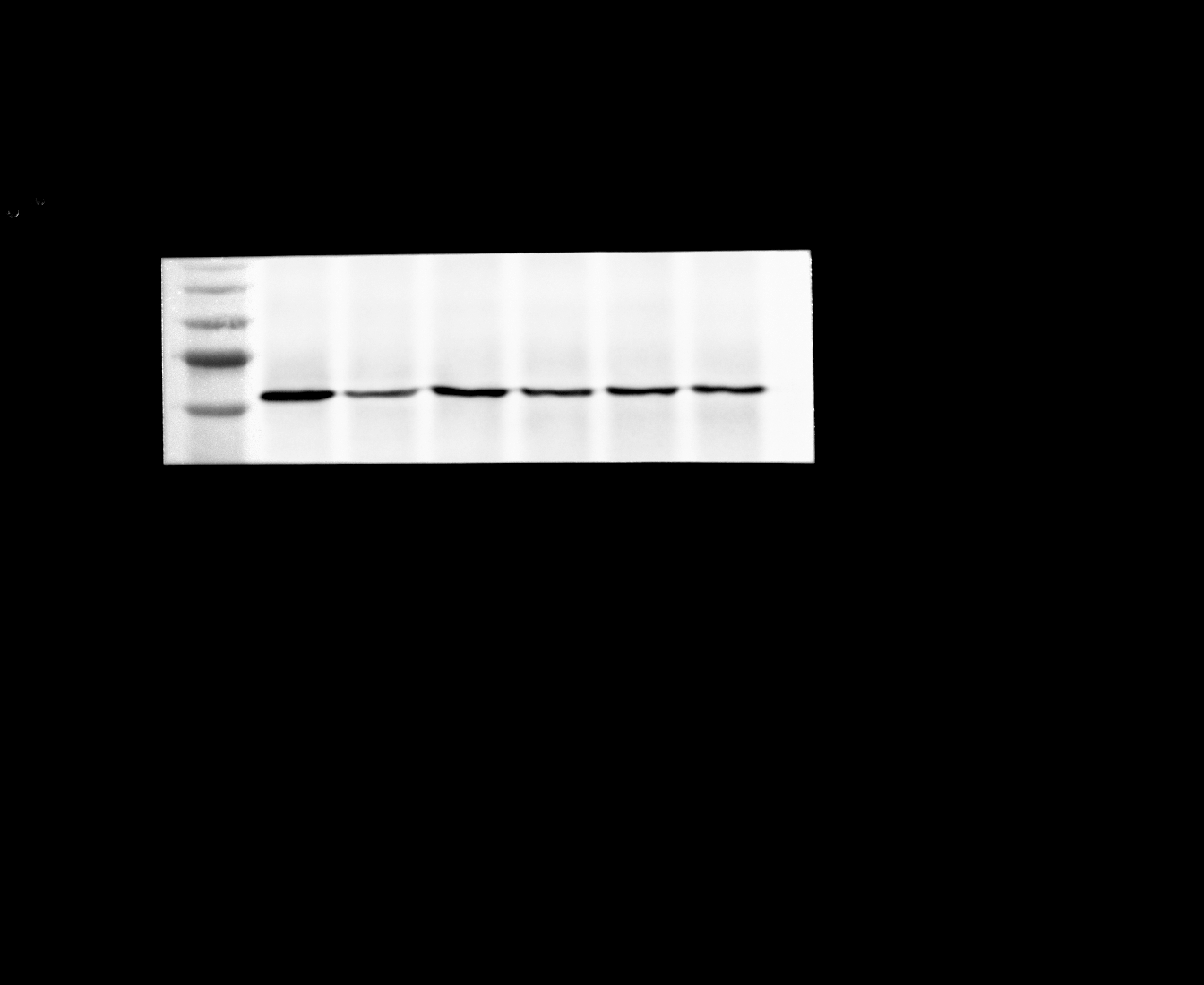

Supplement: Supplementary file 1 [file DataSheet3.zip › WB1/1/p-ampk/5.Tif]

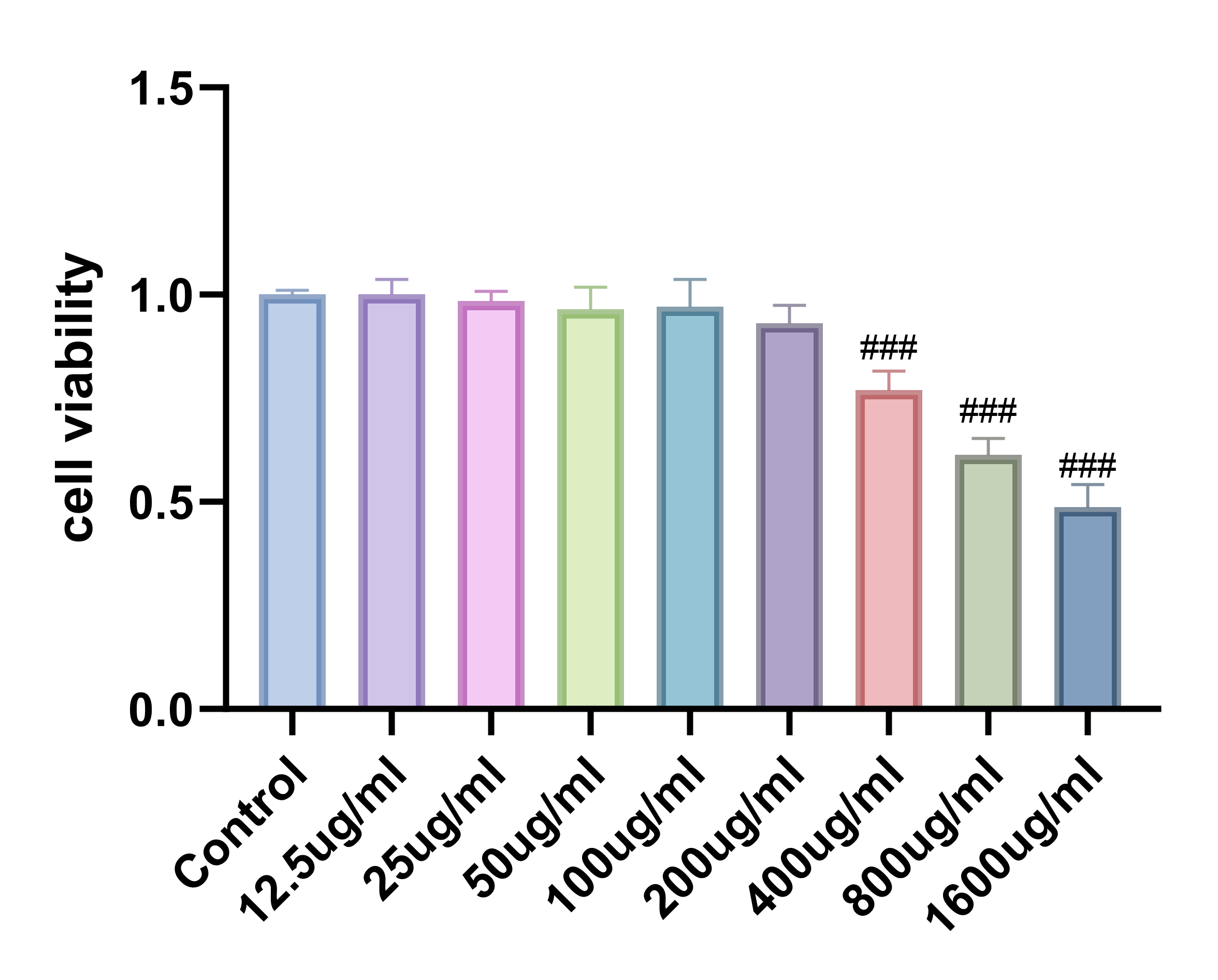

Supplement: Supplementary file 1 [file DataSheet3.zip › 药对毒性/药对毒性c.tif]

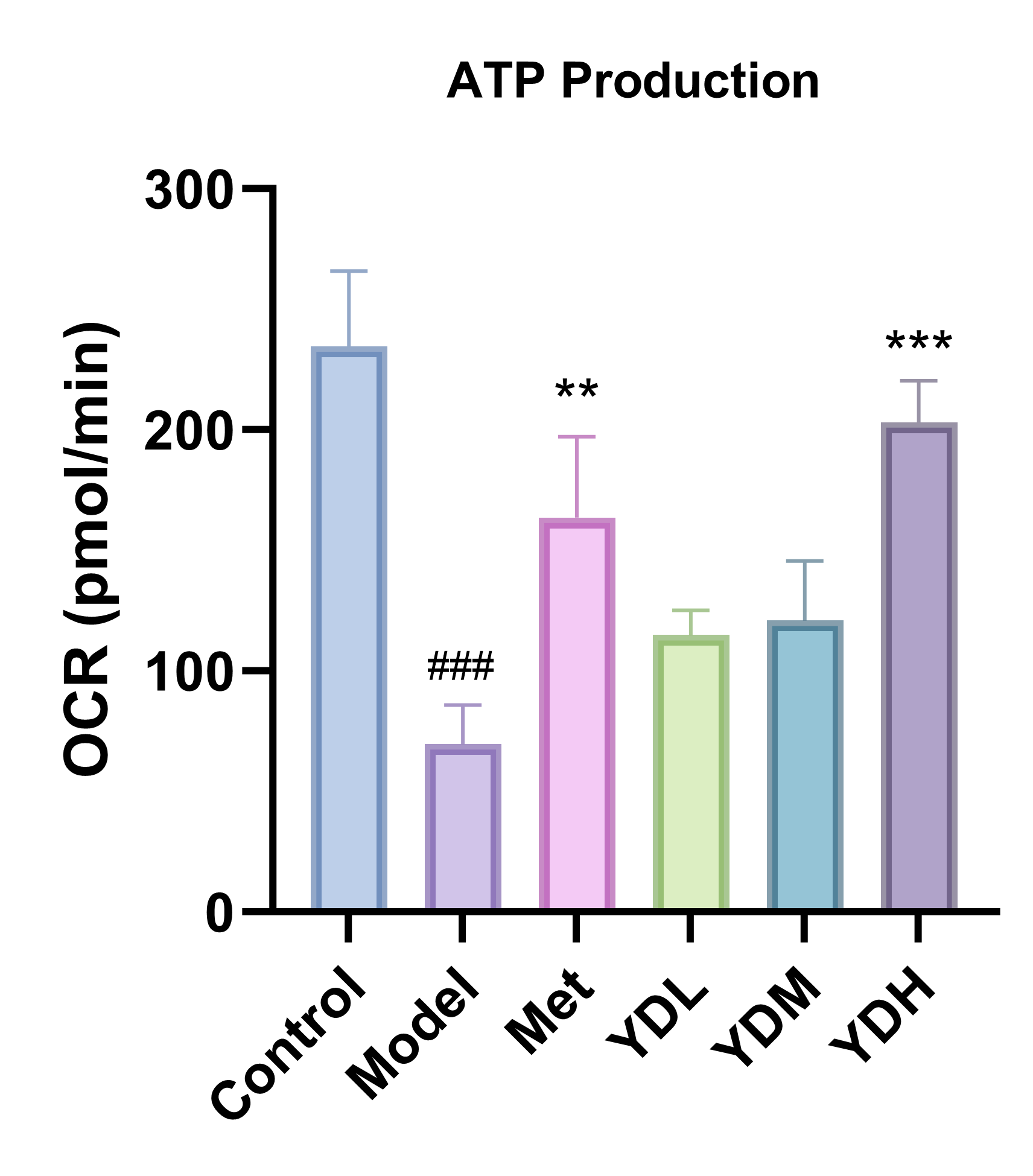

Supplement: Supplementary file 2 [file DataSheet1.zip › 细胞额能量代谢/ATP Production.tif]

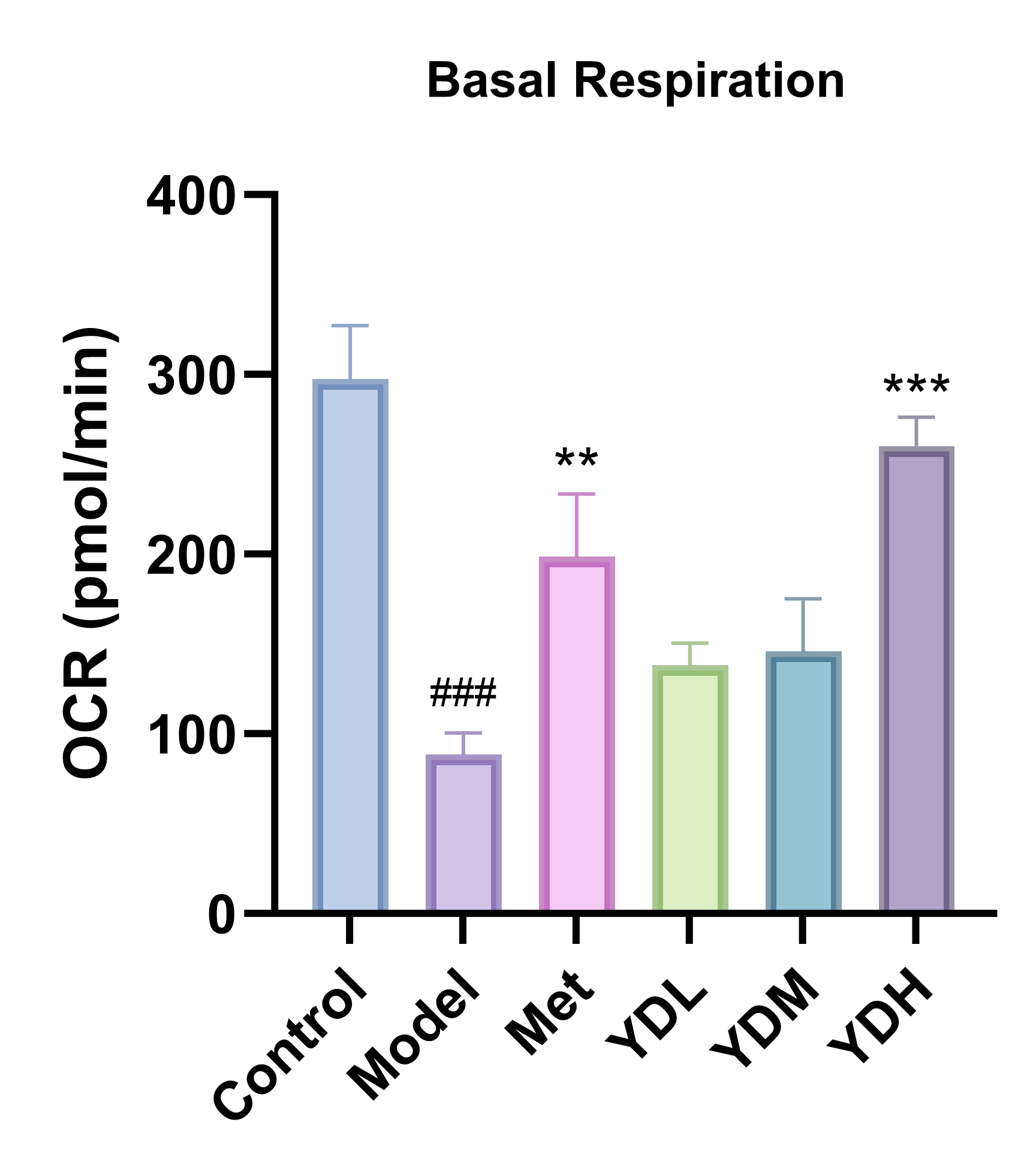

Supplement: Supplementary file 2 [file DataSheet1.zip › 细胞额能量代谢/Basal Respiration.tif]

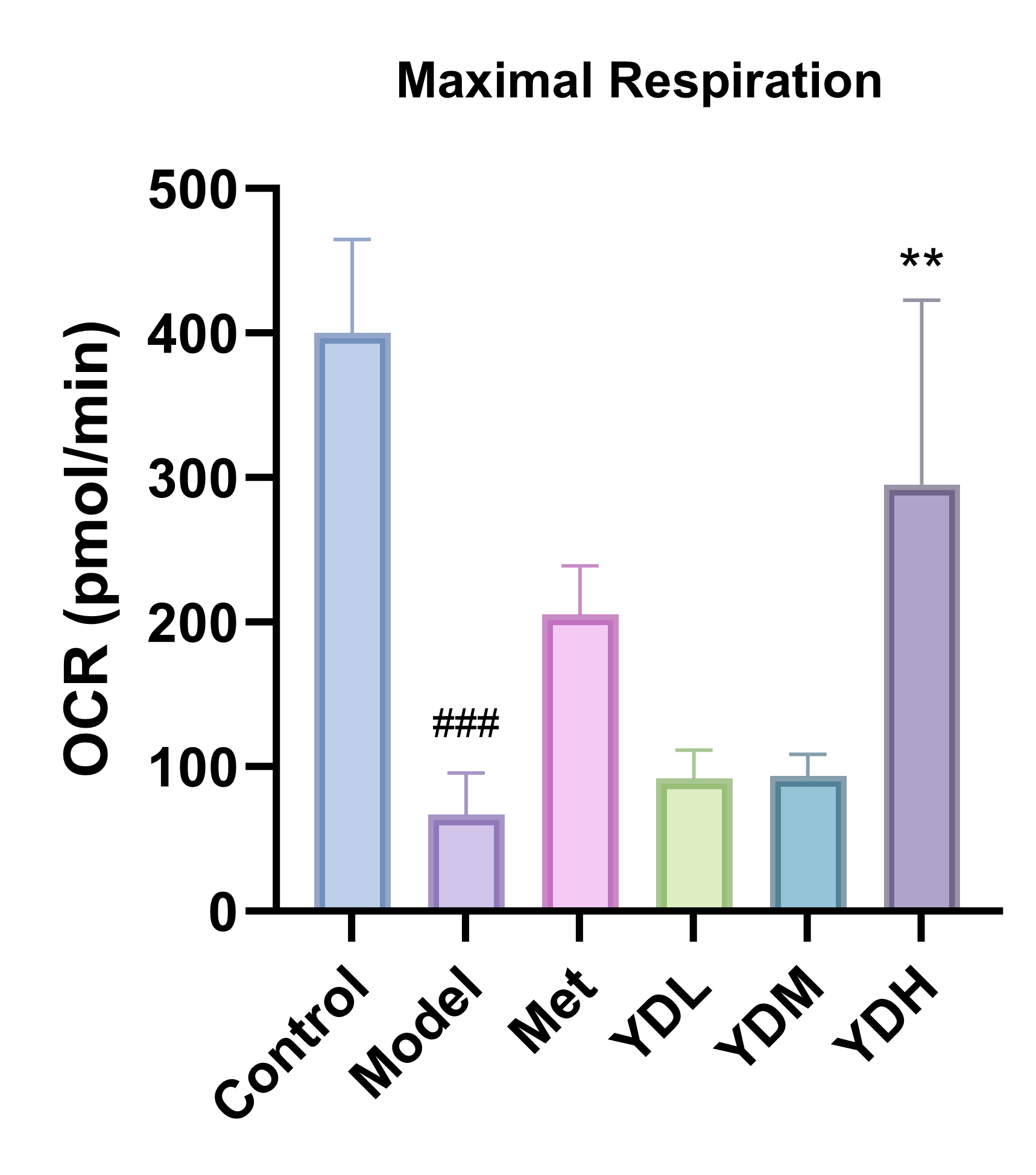

Supplement: Supplementary file 2 [file DataSheet1.zip › 细胞额能量代谢/Maximal Respiration.tif]

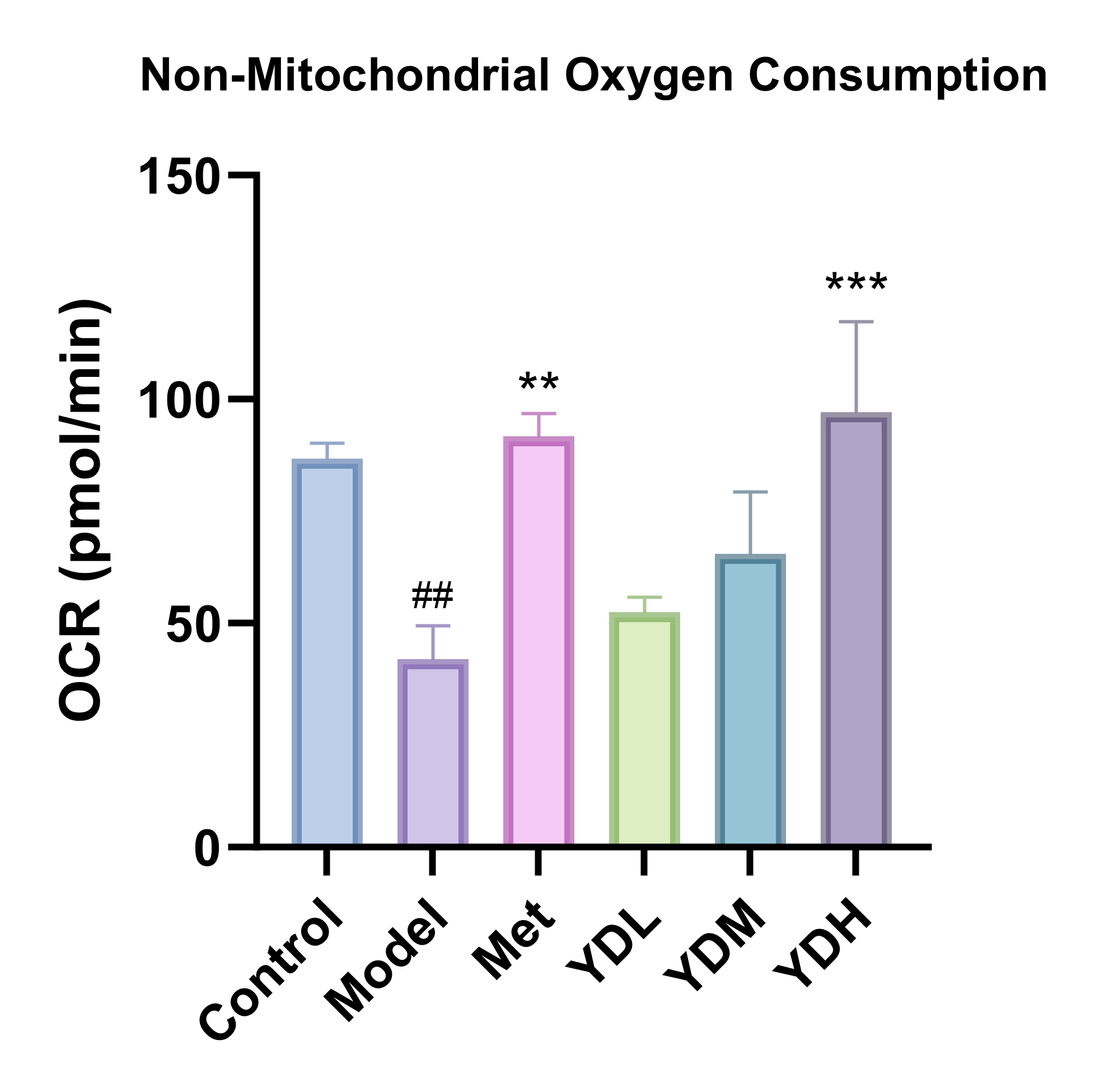

Supplement: Supplementary file 2 [file DataSheet1.zip › 细胞额能量代谢/Non-Mitochondrial Oxygen Consumption.tif]

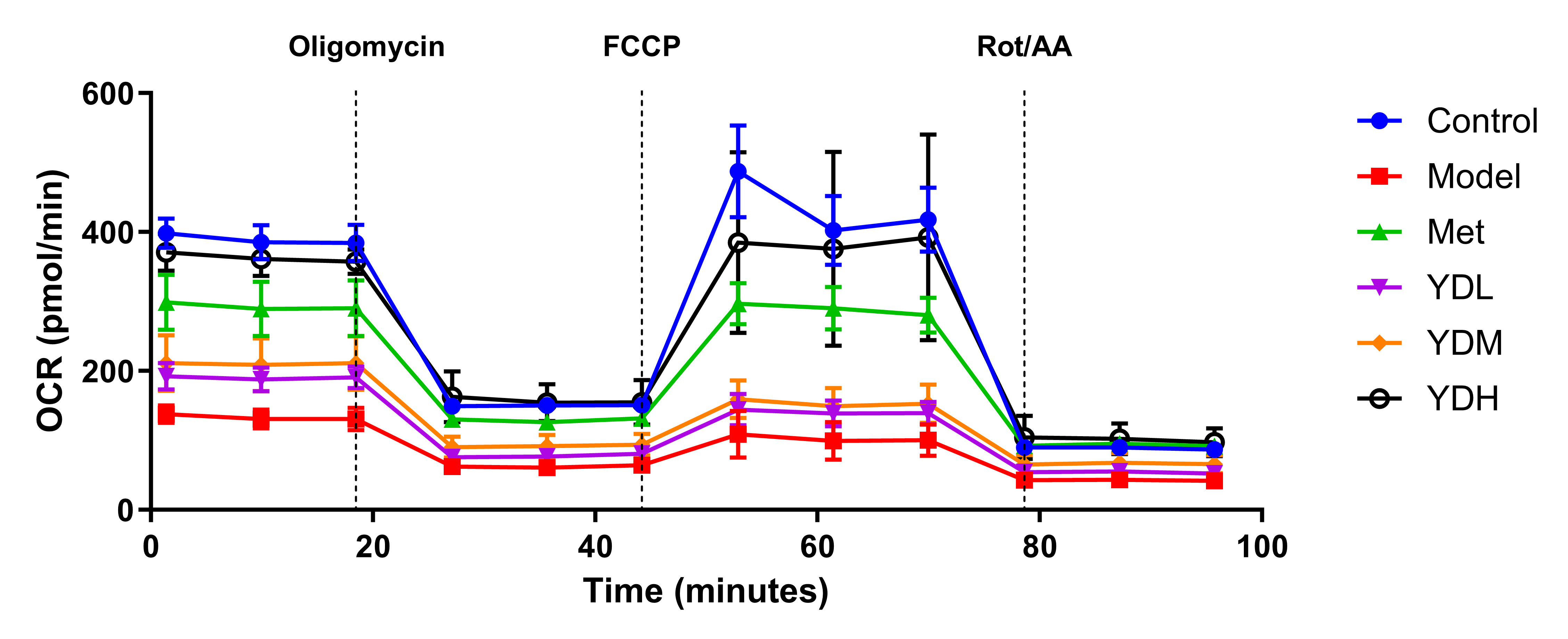

Supplement: Supplementary file 2 [file DataSheet1.zip › 细胞额能量代谢/OCR Data.tif]

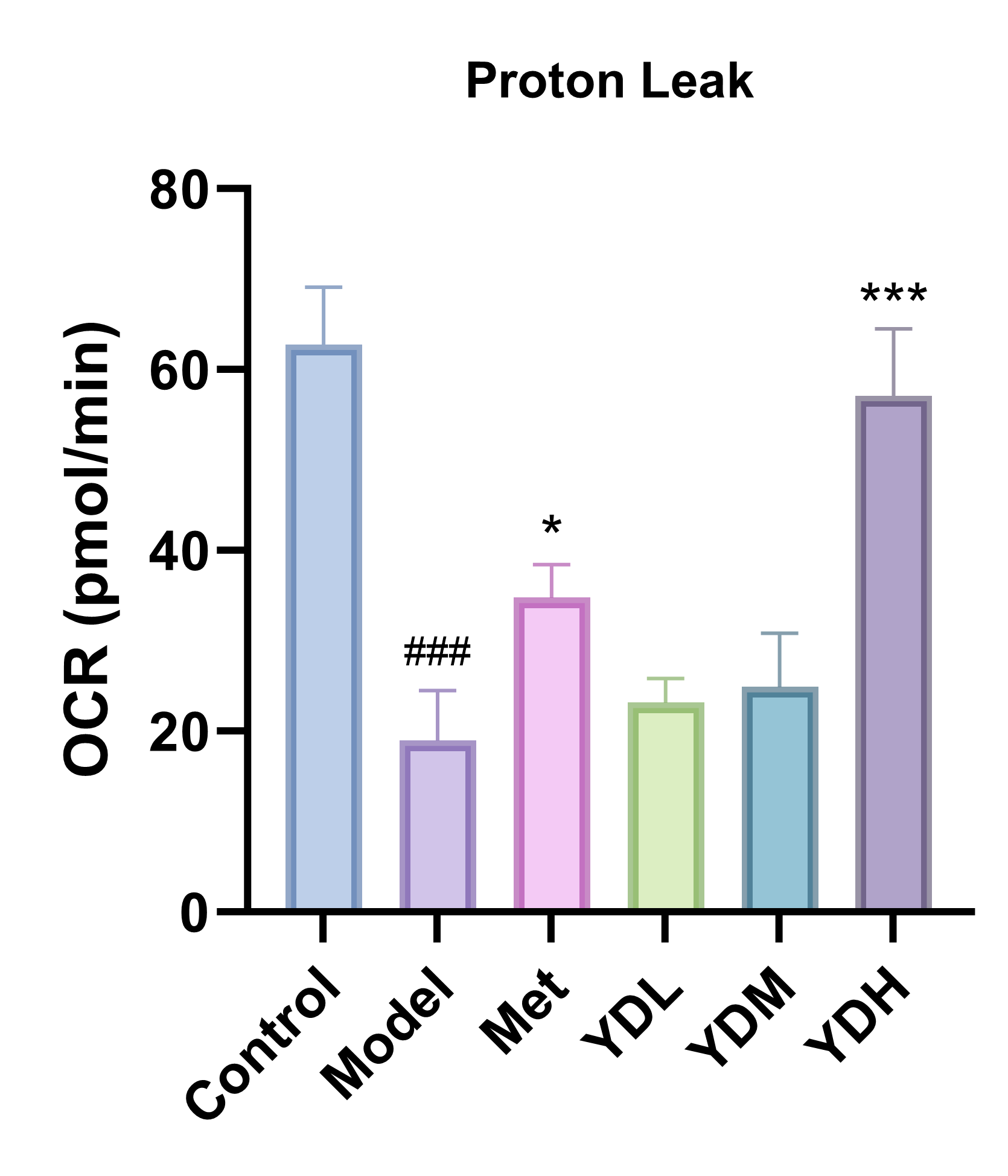

Supplement: Supplementary file 2 [file DataSheet1.zip › 细胞额能量代谢/Proton Leak.tif]

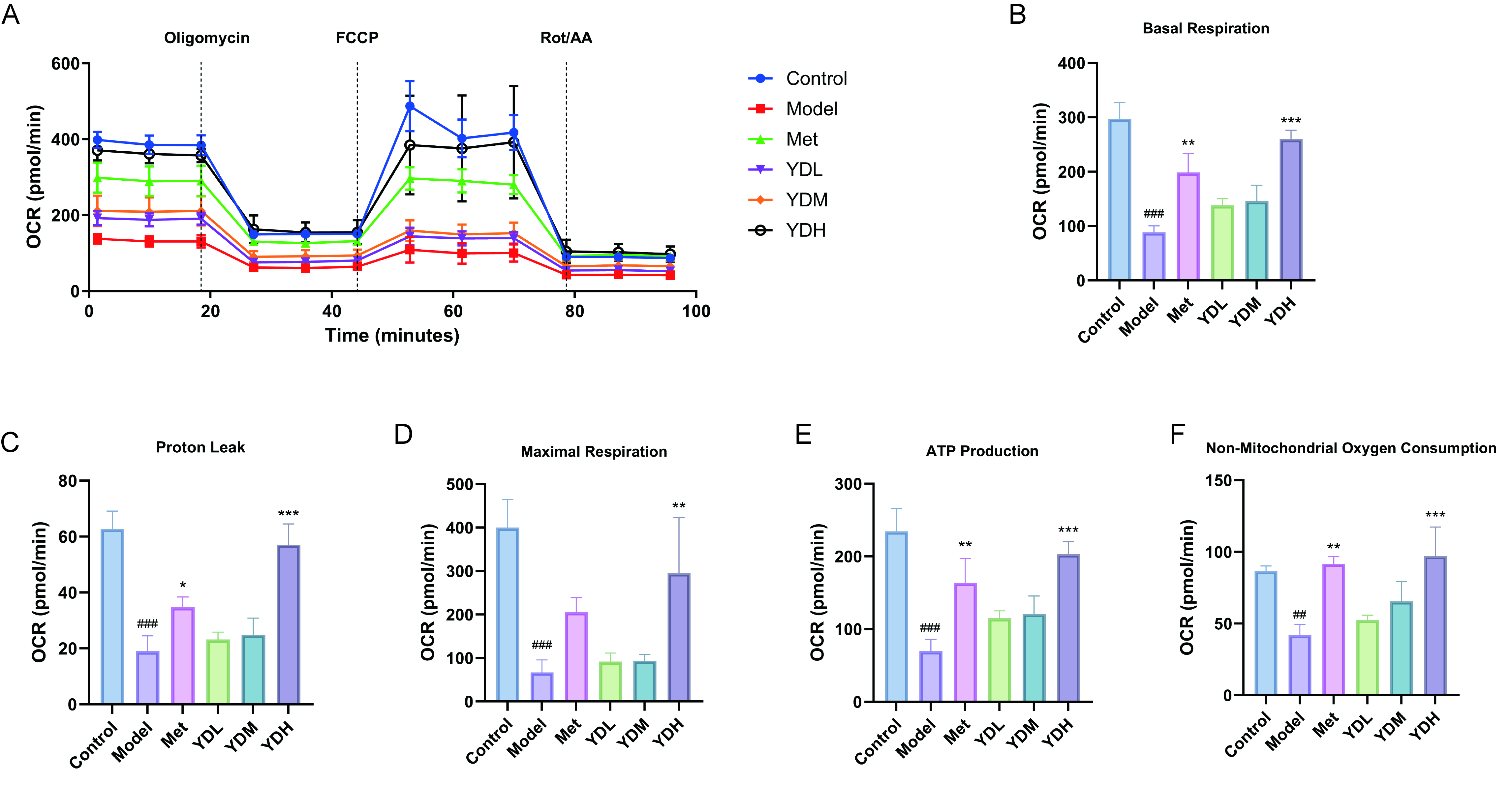

Supplement: Supplementary file 2 [file DataSheet1.zip › 细胞额能量代谢/未标题-1.tif]

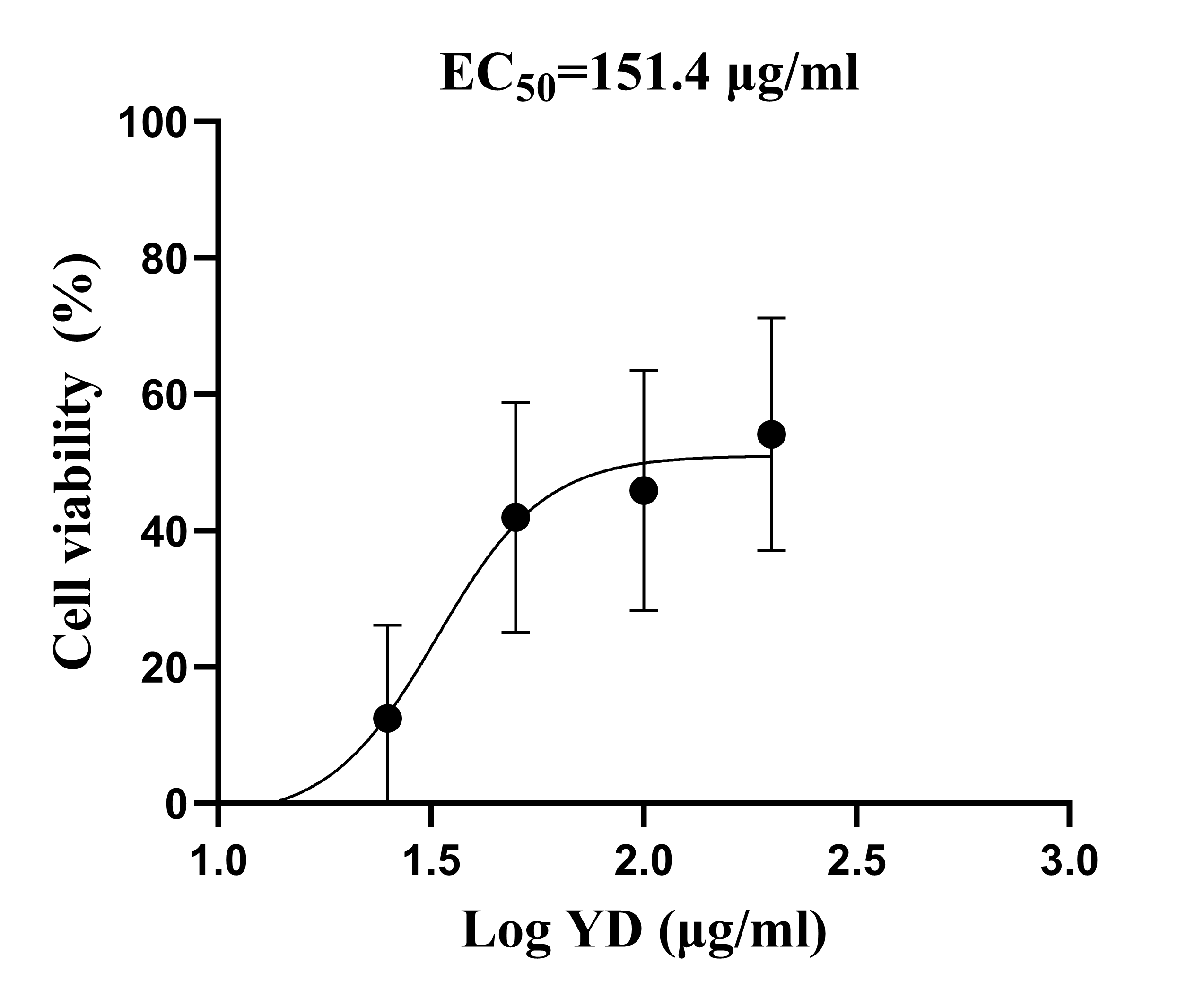

Supplement: Supplementary file 2 [file DataSheet1.zip › 药对保护/罗格列酮曲线.tif]

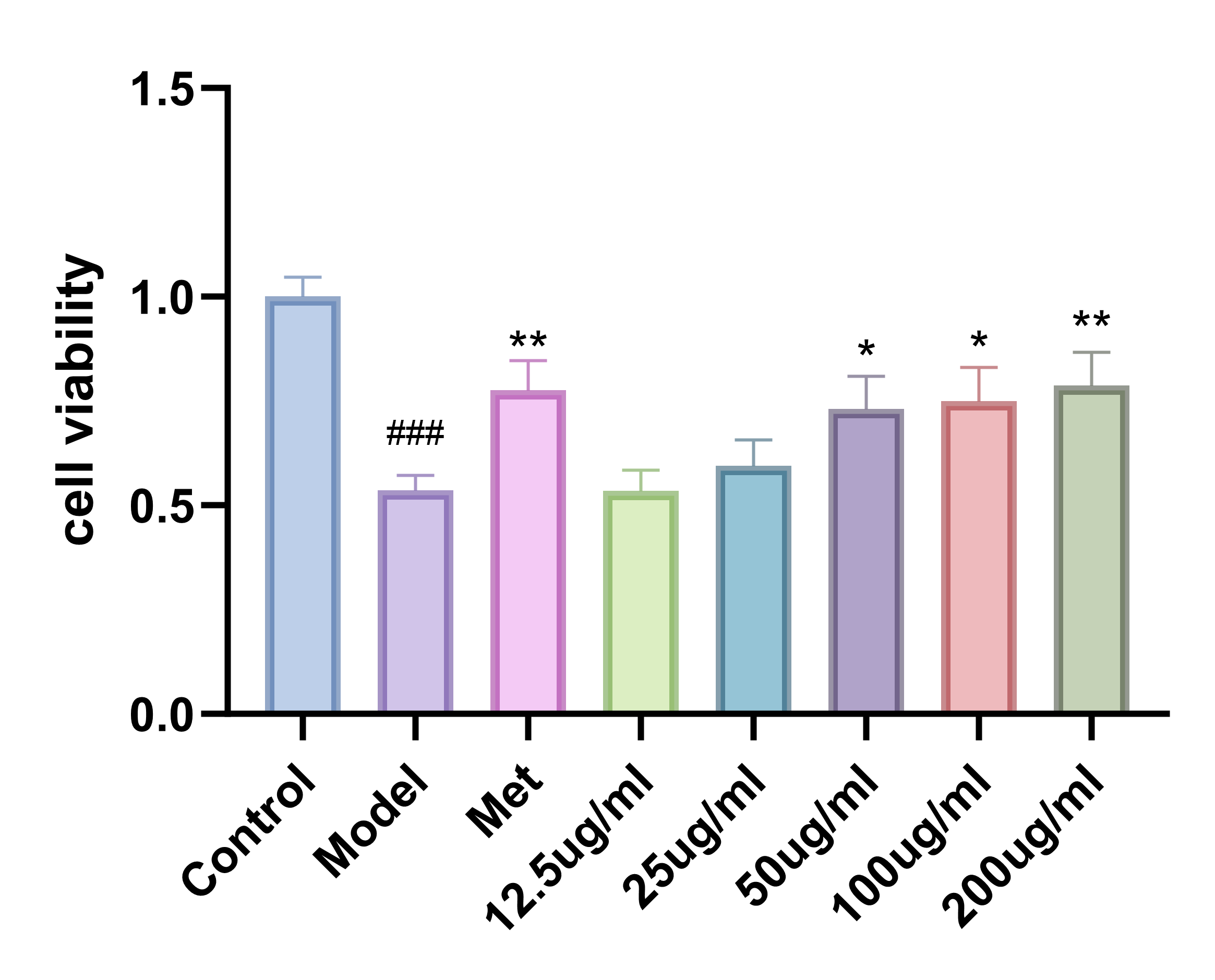

Supplement: Supplementary file 2 [file DataSheet1.zip › 药对保护/药对保护.tif]

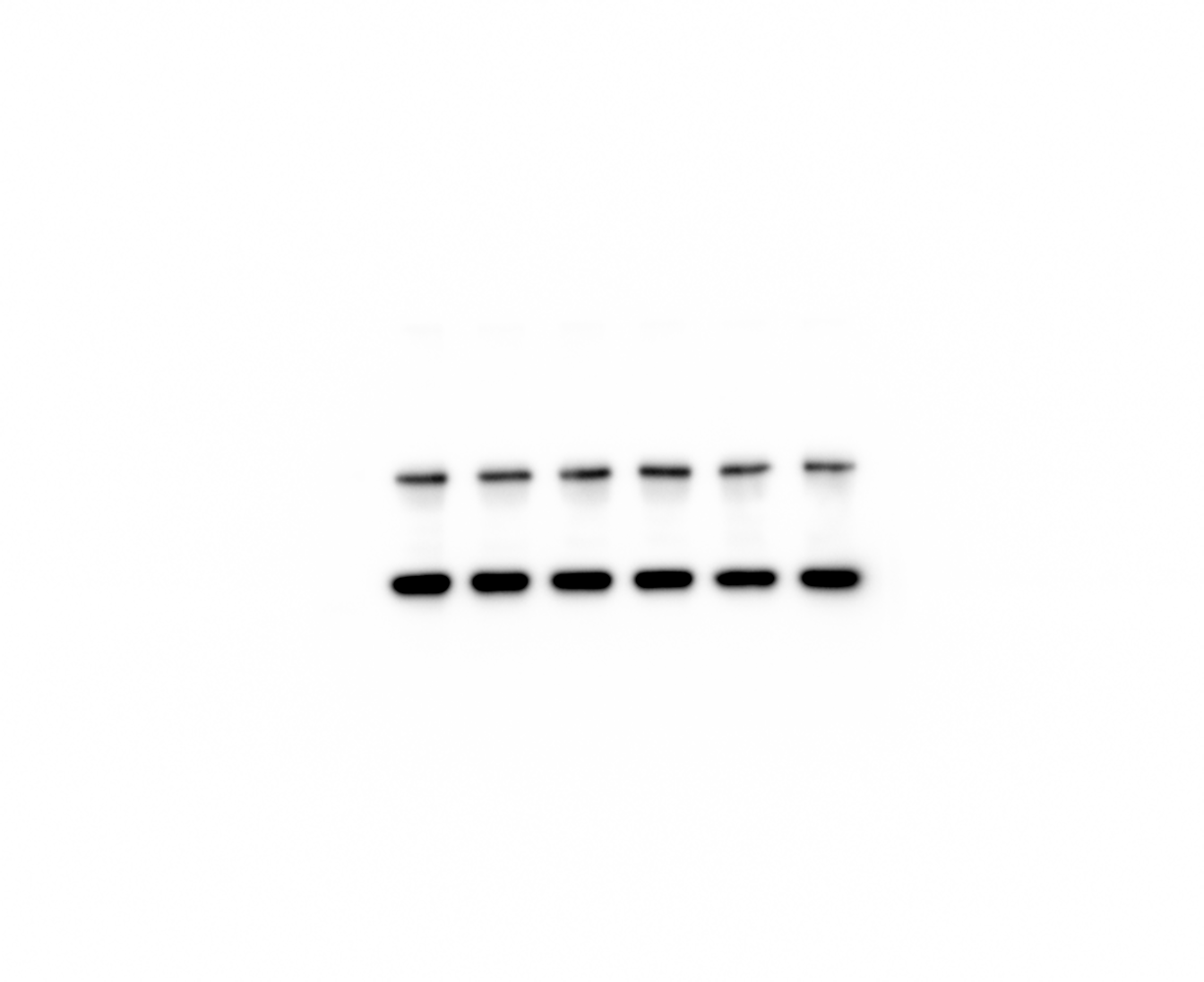

Supplement: Supplementary file 3 [file DataSheet2.zip › 4/ampk/ampk 1s.Tif]

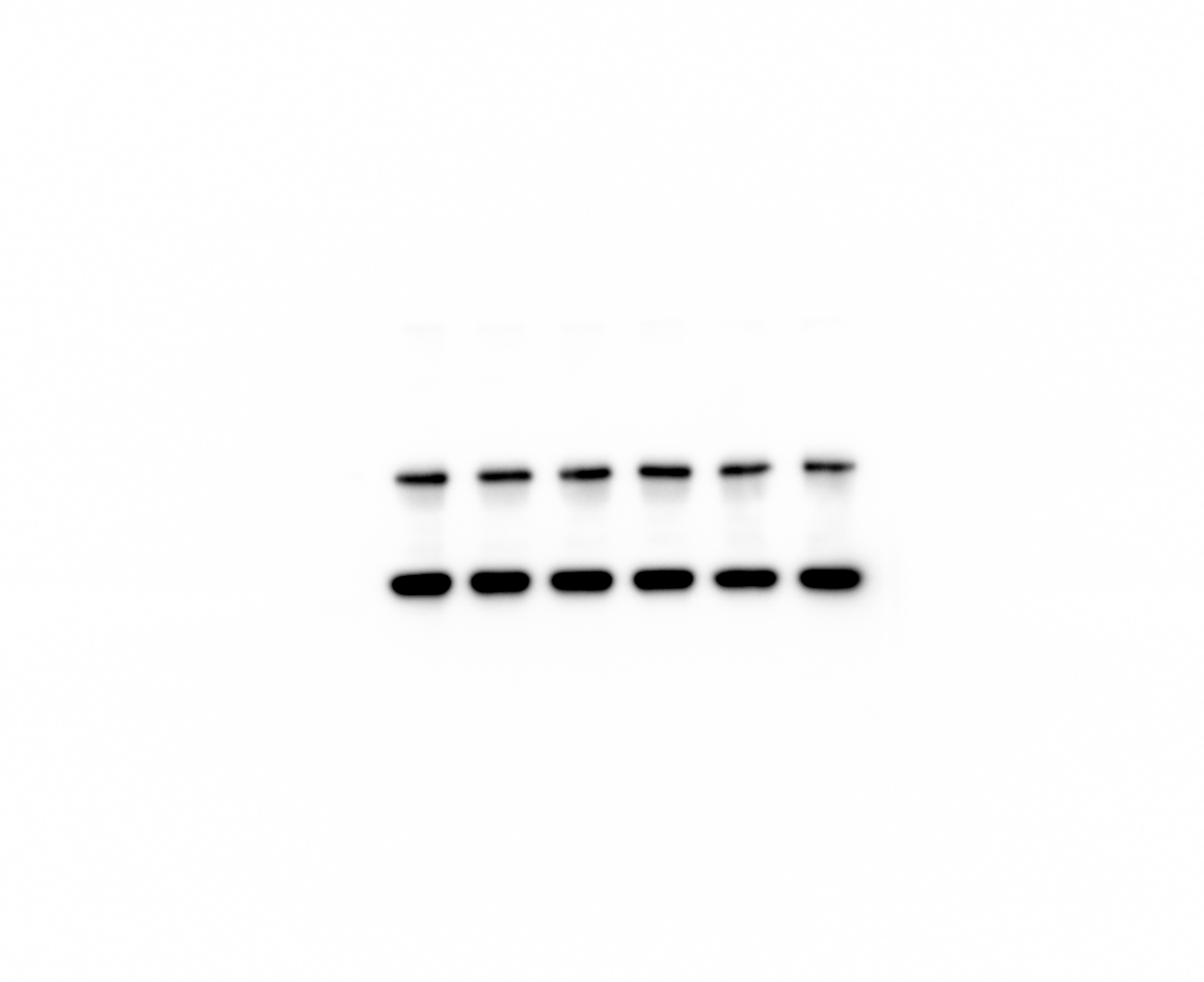

Supplement: Supplementary file 3 [file DataSheet2.zip › 4/ampk/ampk 3s.Tif]

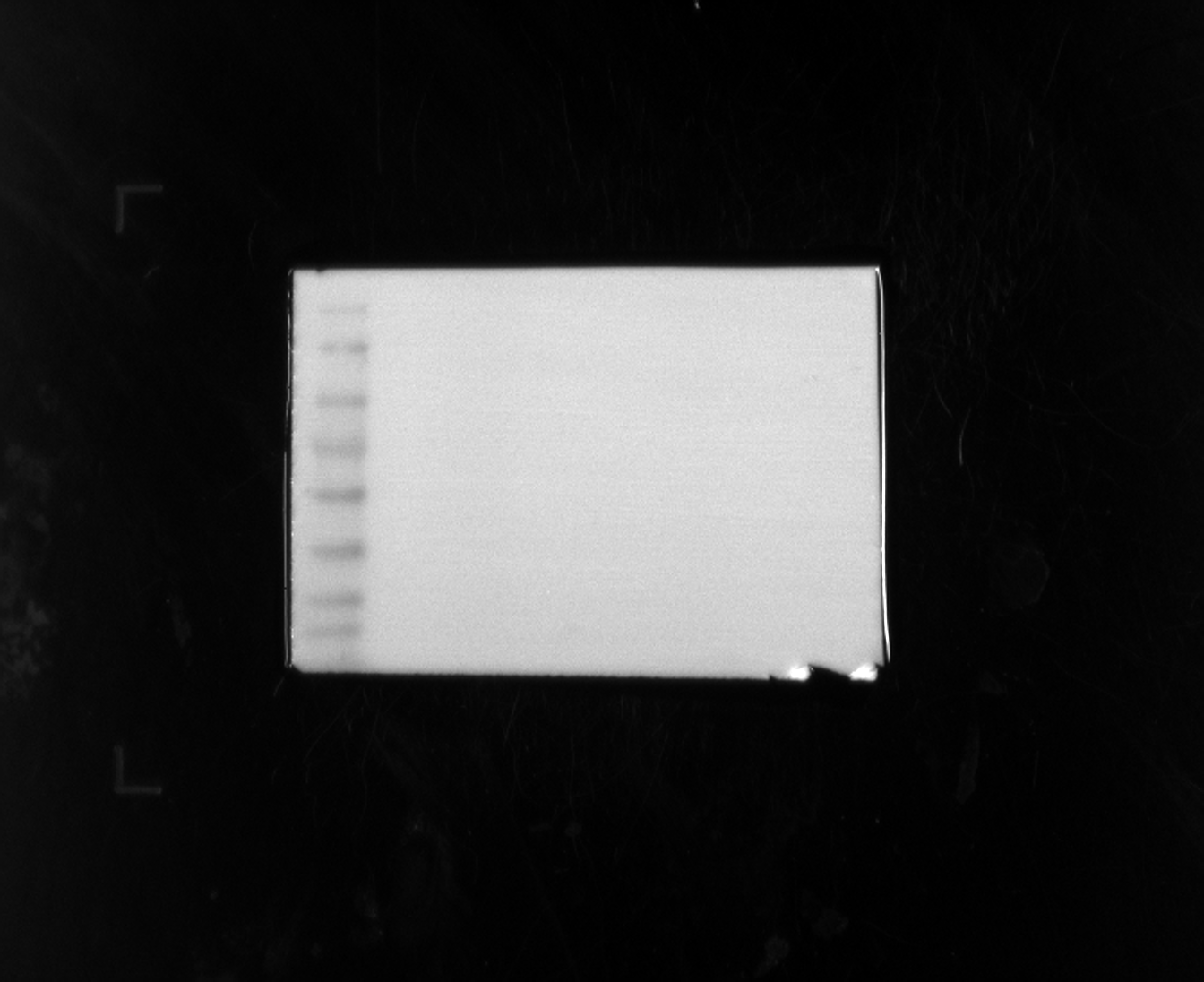

Supplement: Supplementary file 3 [file DataSheet2.zip › 4/ampk/marker.Tif]

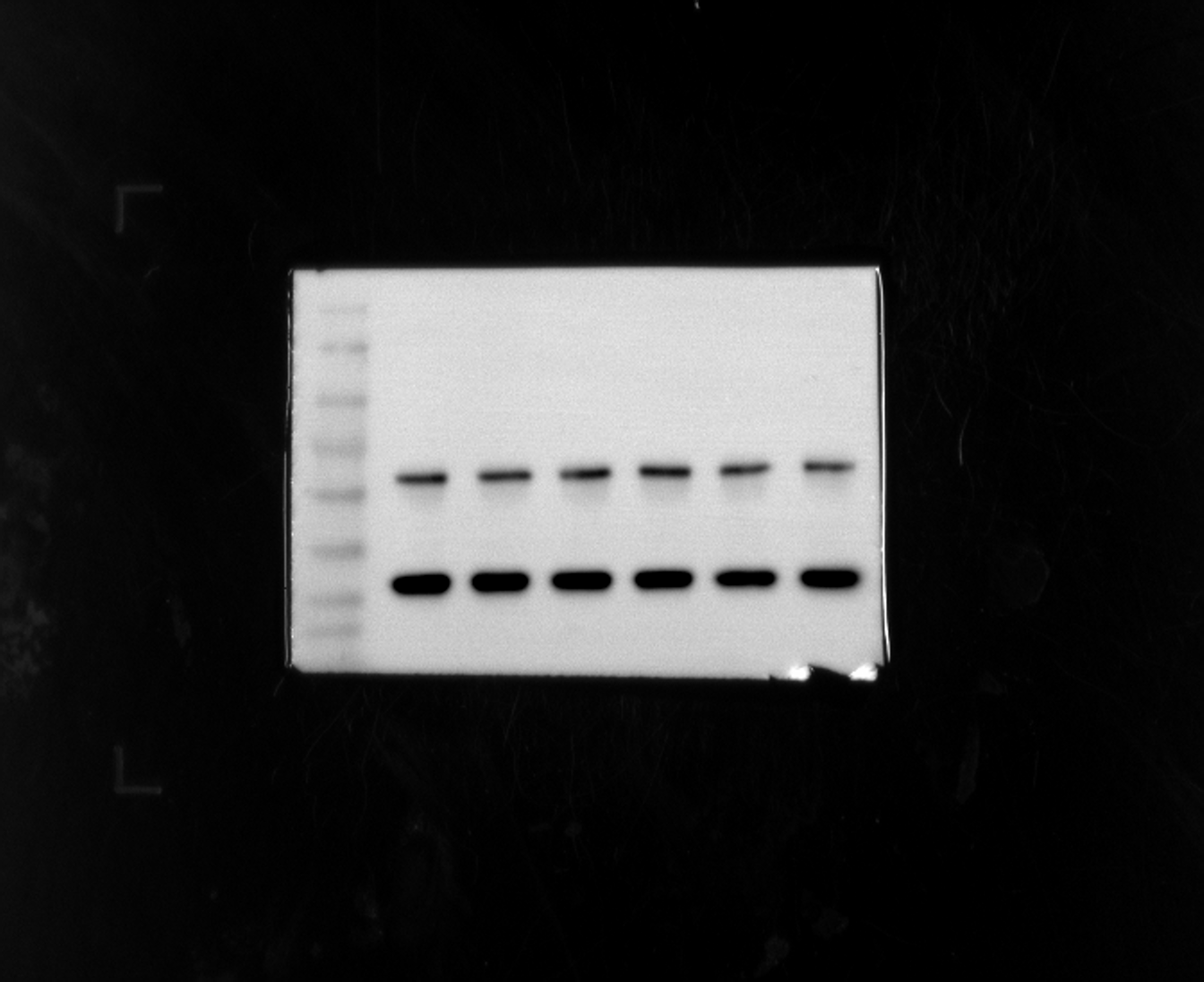

Supplement: Supplementary file 3 [file DataSheet2.zip › 4/ampk/merged 1s.Tif]

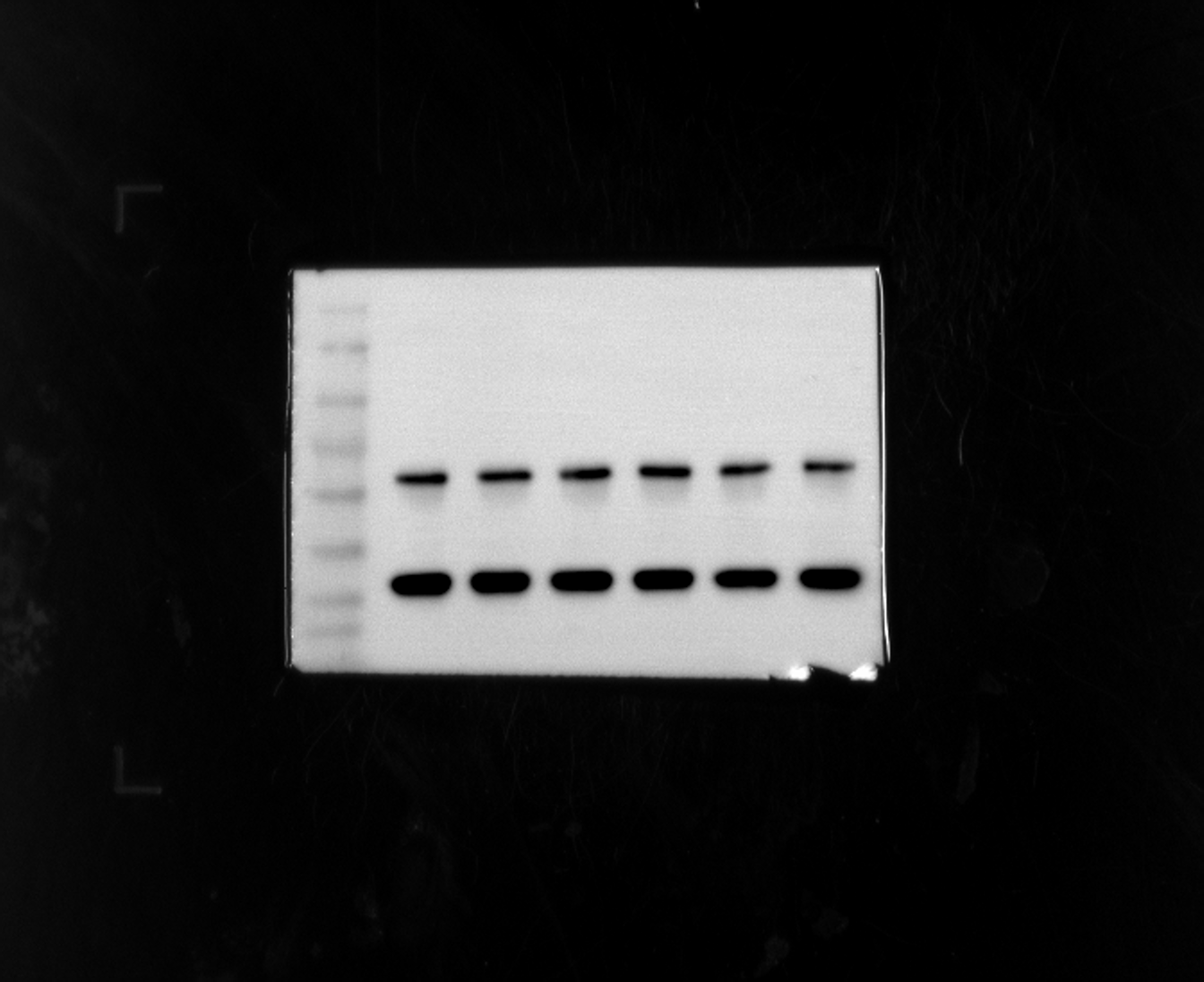

Supplement: Supplementary file 3 [file DataSheet2.zip › 4/ampk/merged 3s.Tif]

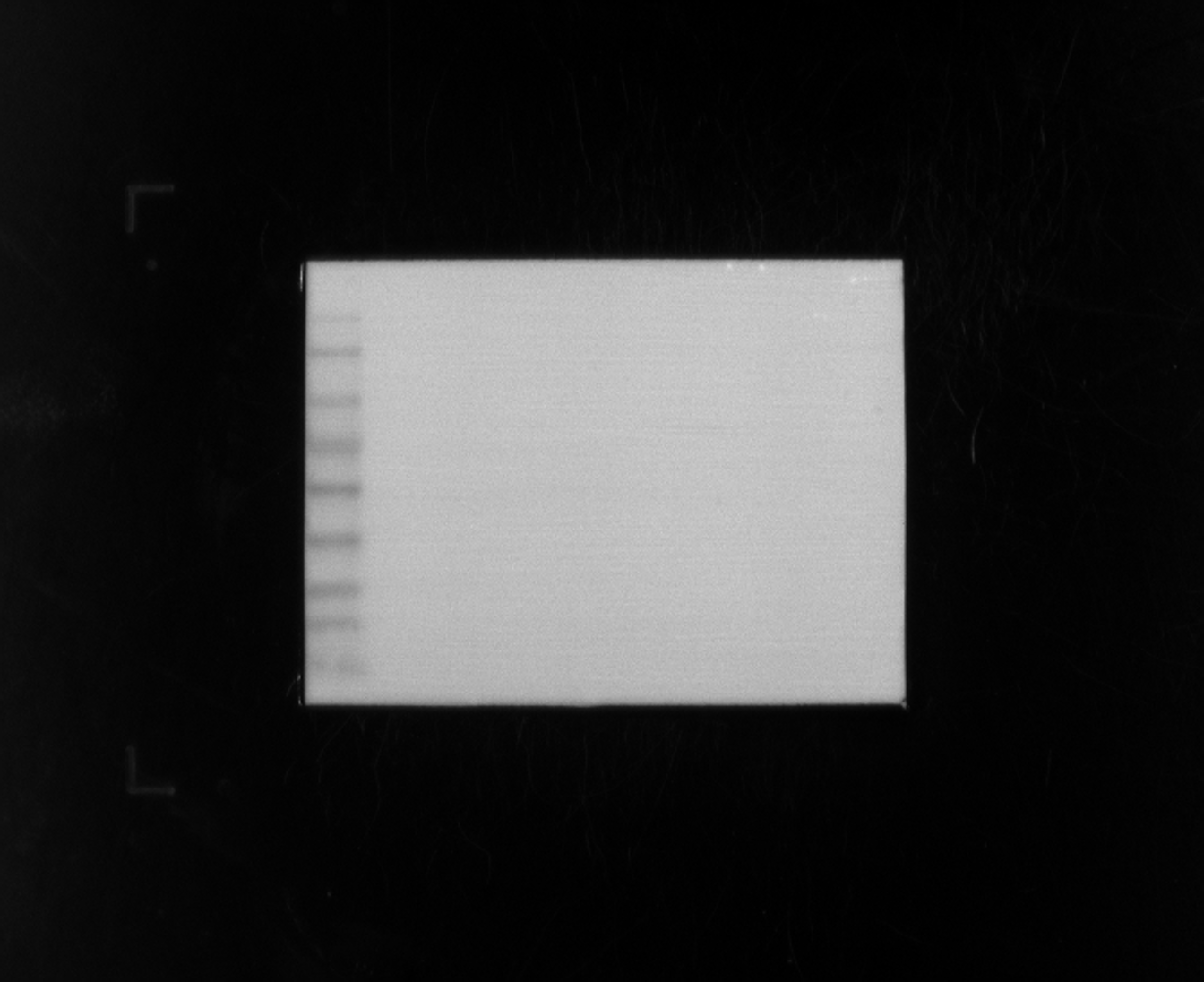

Supplement: Supplementary file 3 [file DataSheet2.zip › 4/p-ampk/marker.Tif]

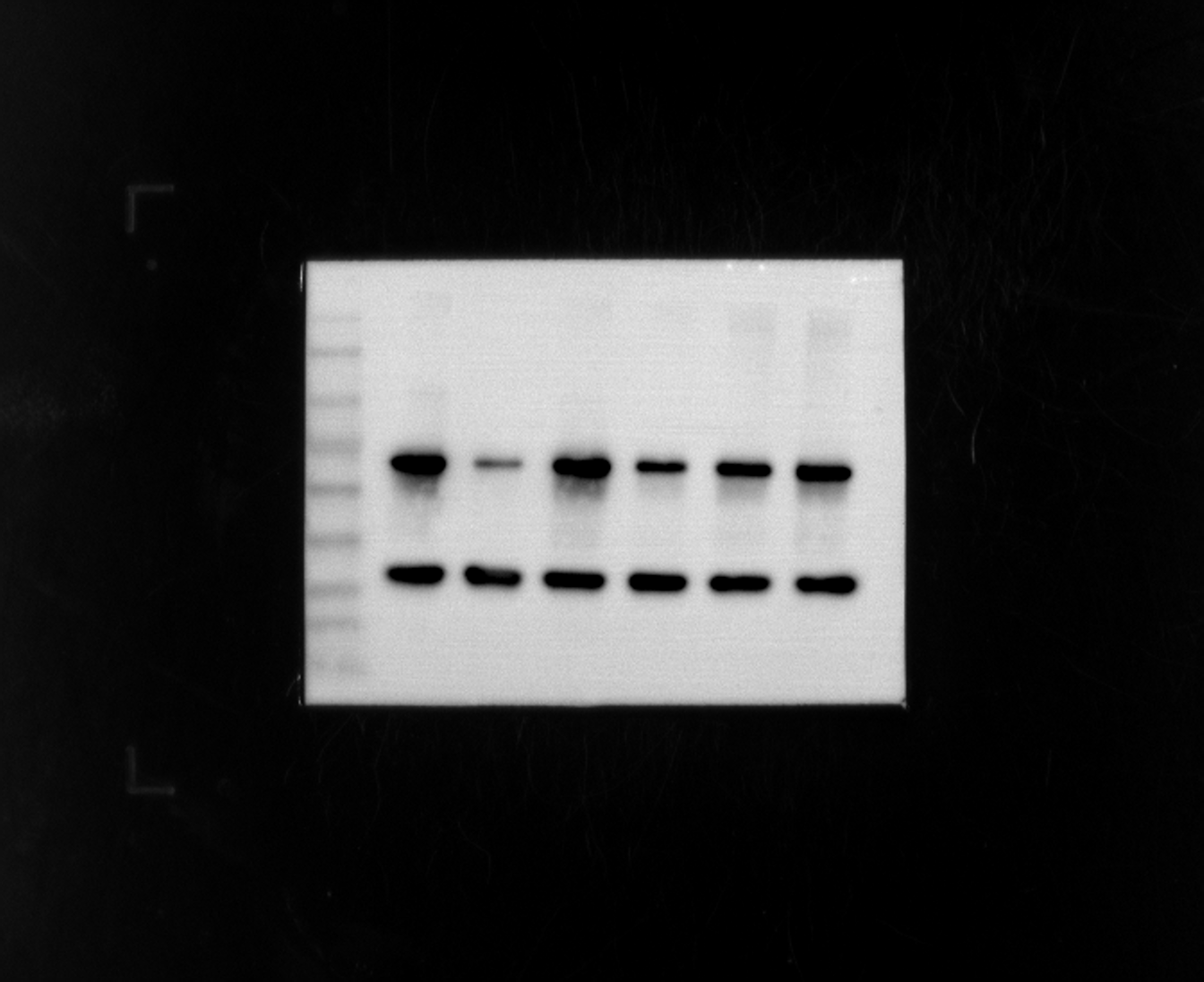

Supplement: Supplementary file 3 [file DataSheet2.zip › 4/p-ampk/merged 1s.Tif]

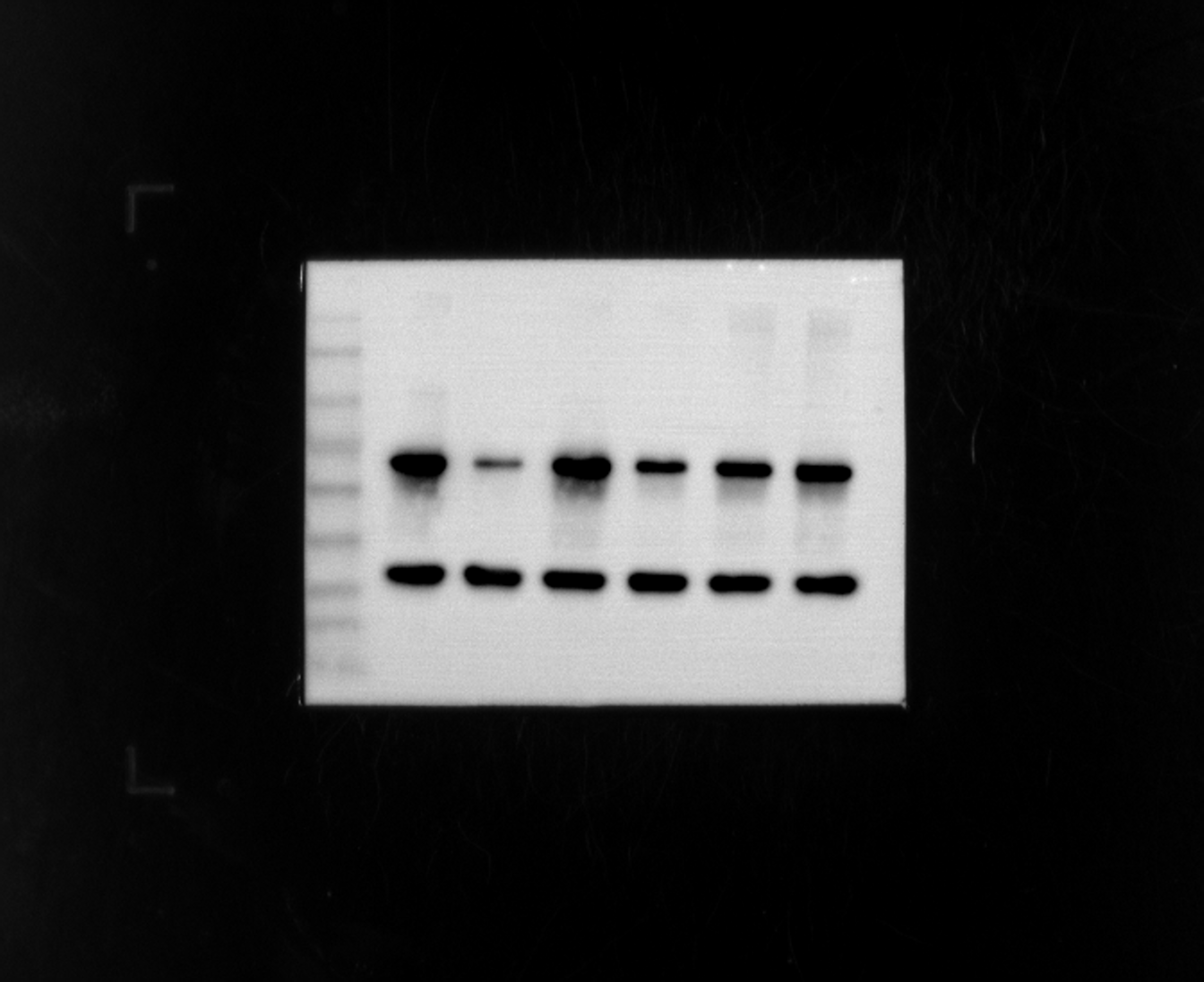

Supplement: Supplementary file 3 [file DataSheet2.zip › 4/p-ampk/merged 3s.Tif]

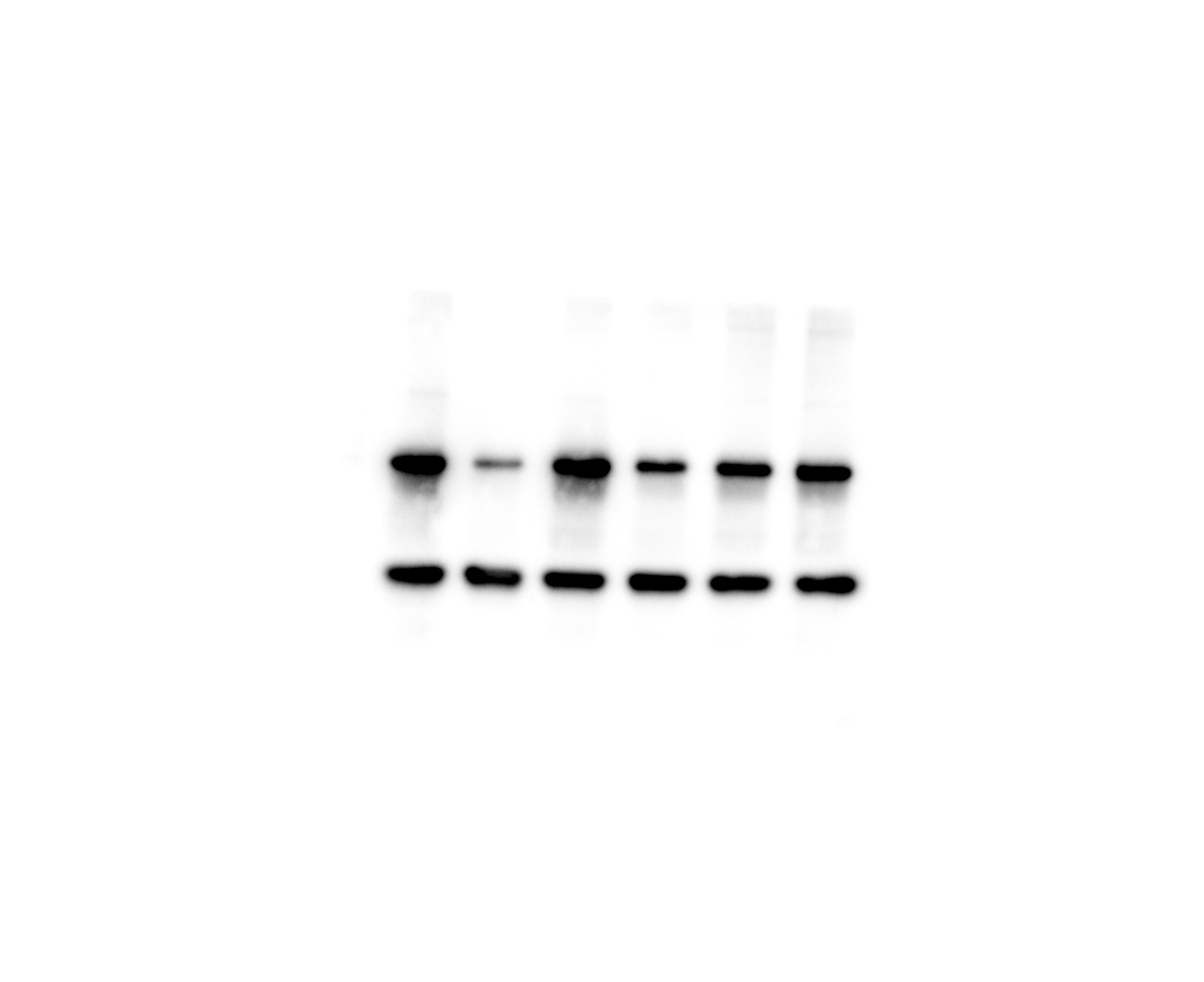

Supplement: Supplementary file 3 [file DataSheet2.zip › 4/p-ampk/p-ampk 1s.Tif]

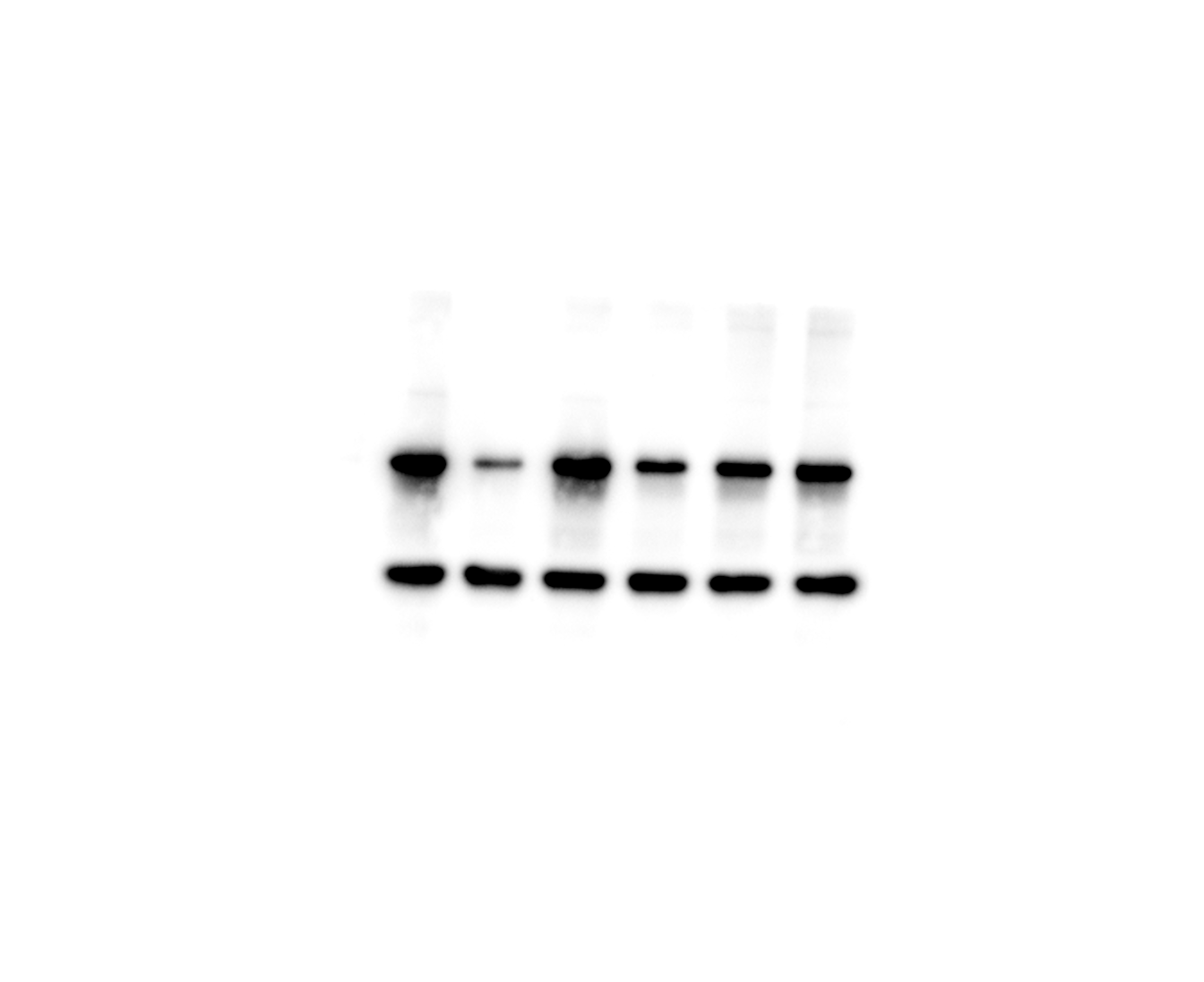

Supplement: Supplementary file 3 [file DataSheet2.zip › 4/p-ampk/p-ampk 3s.Tif]

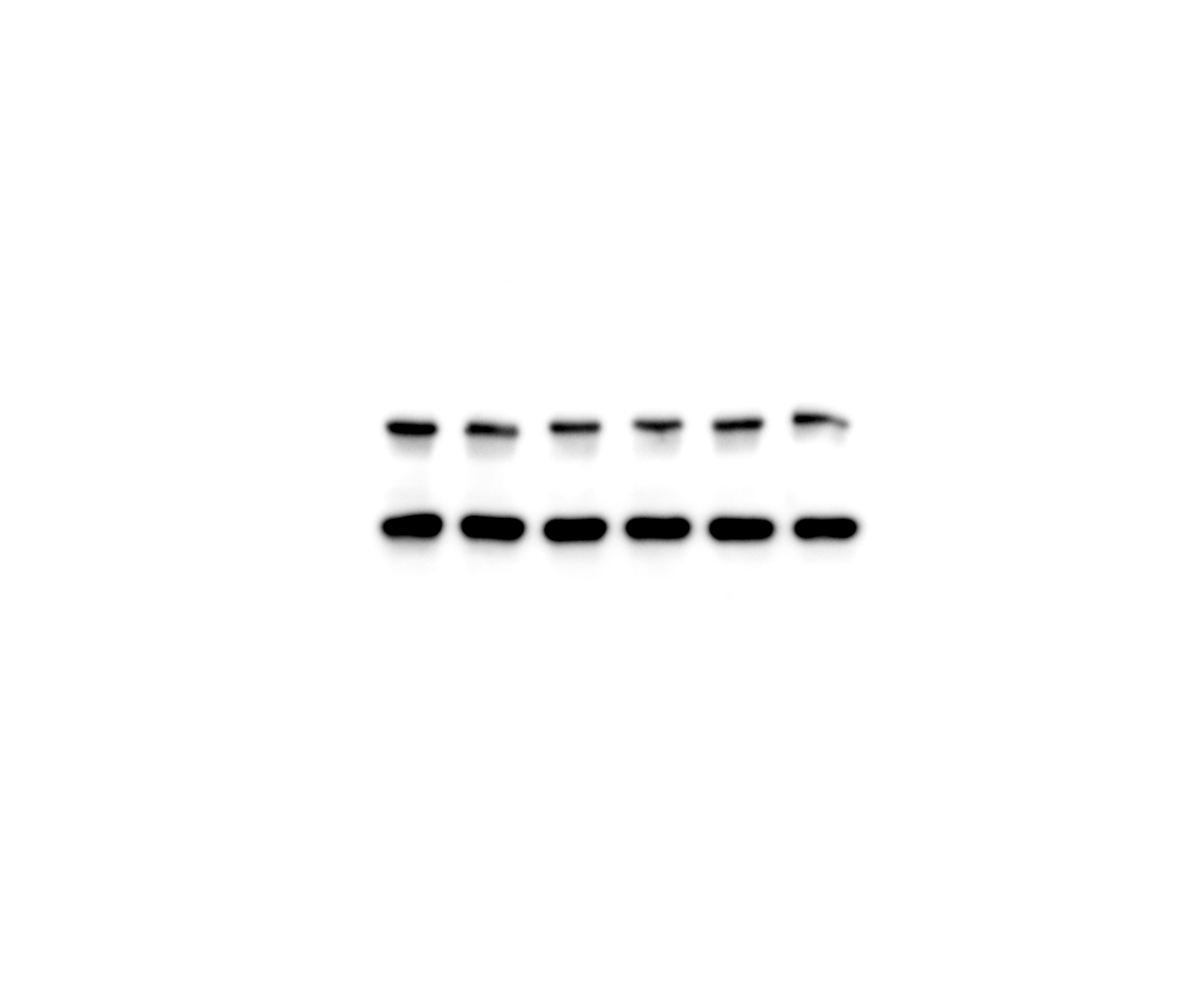

Supplement: Supplementary file 3 [file DataSheet2.zip › 5/ampk/ampk 1s.Tif]

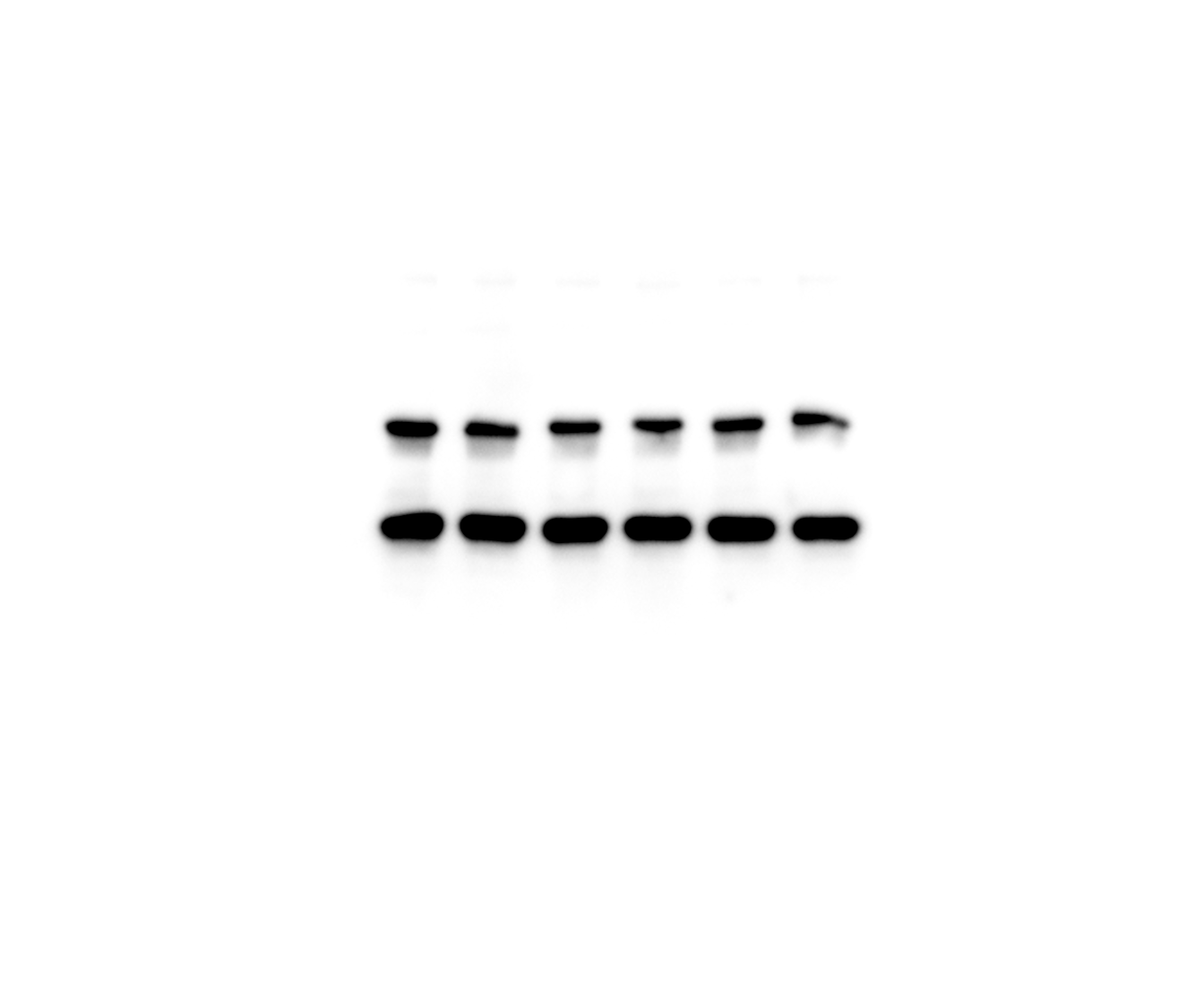

Supplement: Supplementary file 3 [file DataSheet2.zip › 5/ampk/ampk 3s.Tif]

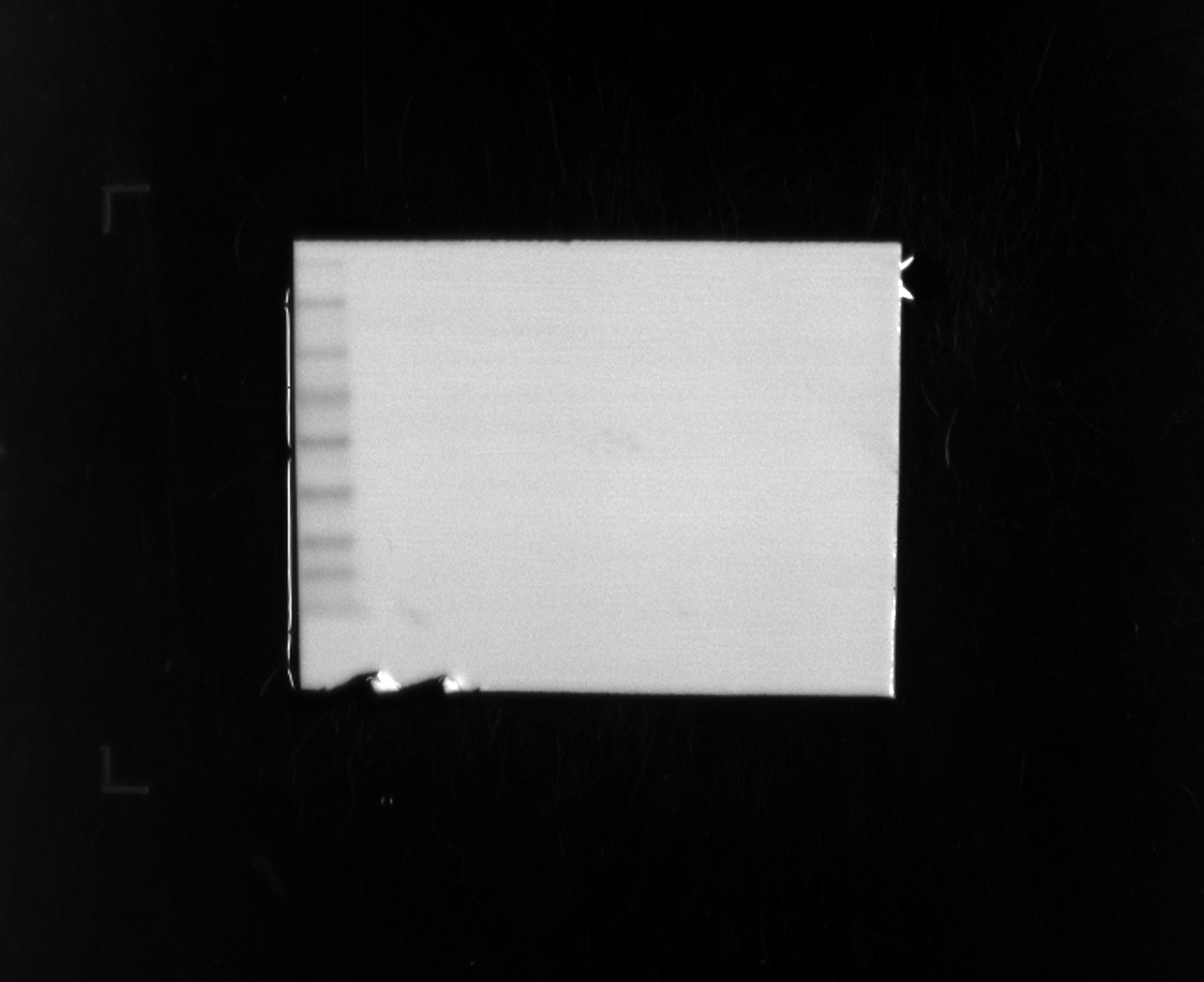

Supplement: Supplementary file 3 [file DataSheet2.zip › 5/ampk/marker.Tif]

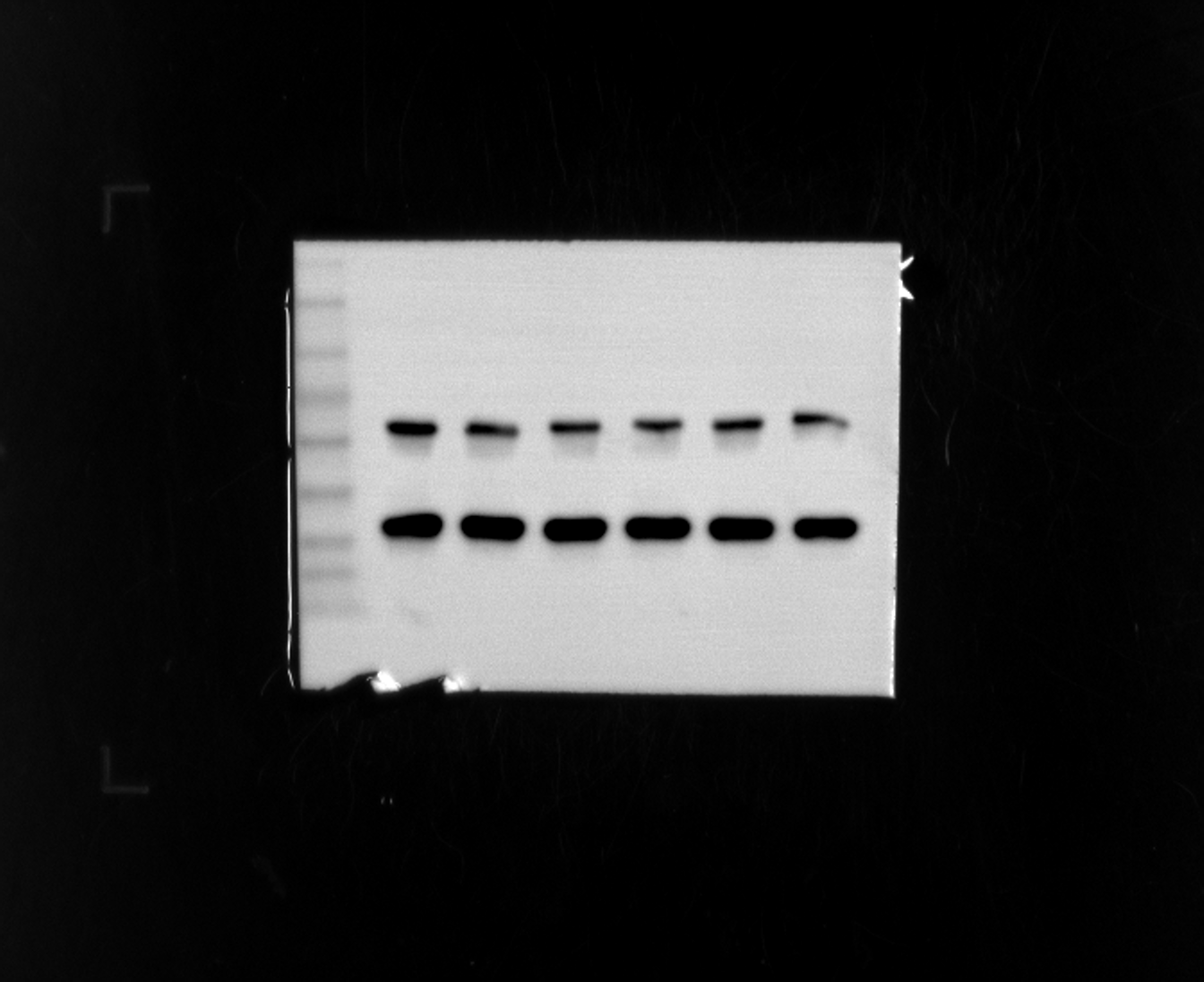

Supplement: Supplementary file 3 [file DataSheet2.zip › 5/ampk/merged 1s.Tif]

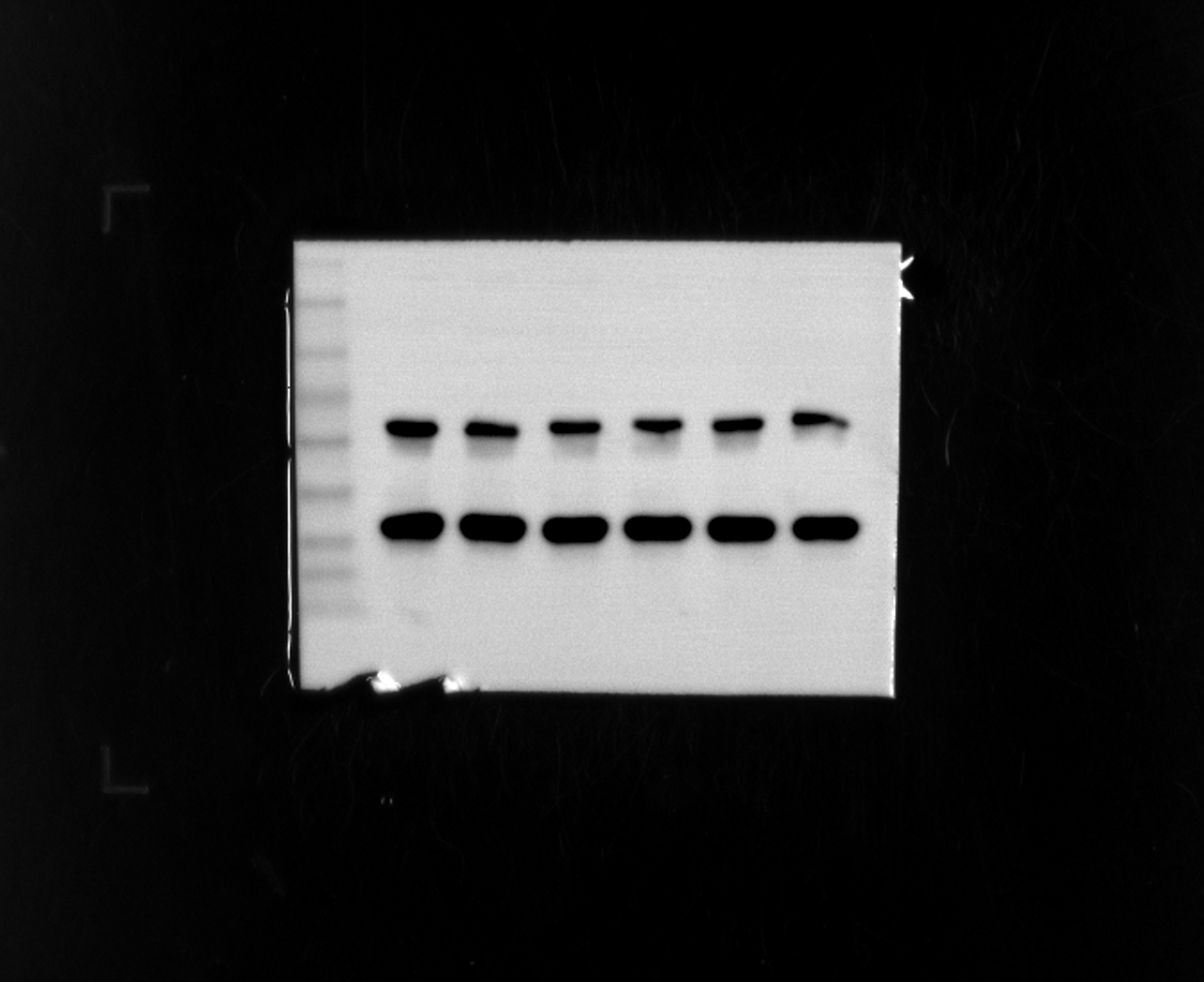

Supplement: Supplementary file 3 [file DataSheet2.zip › 5/ampk/merged 3s.Tif]

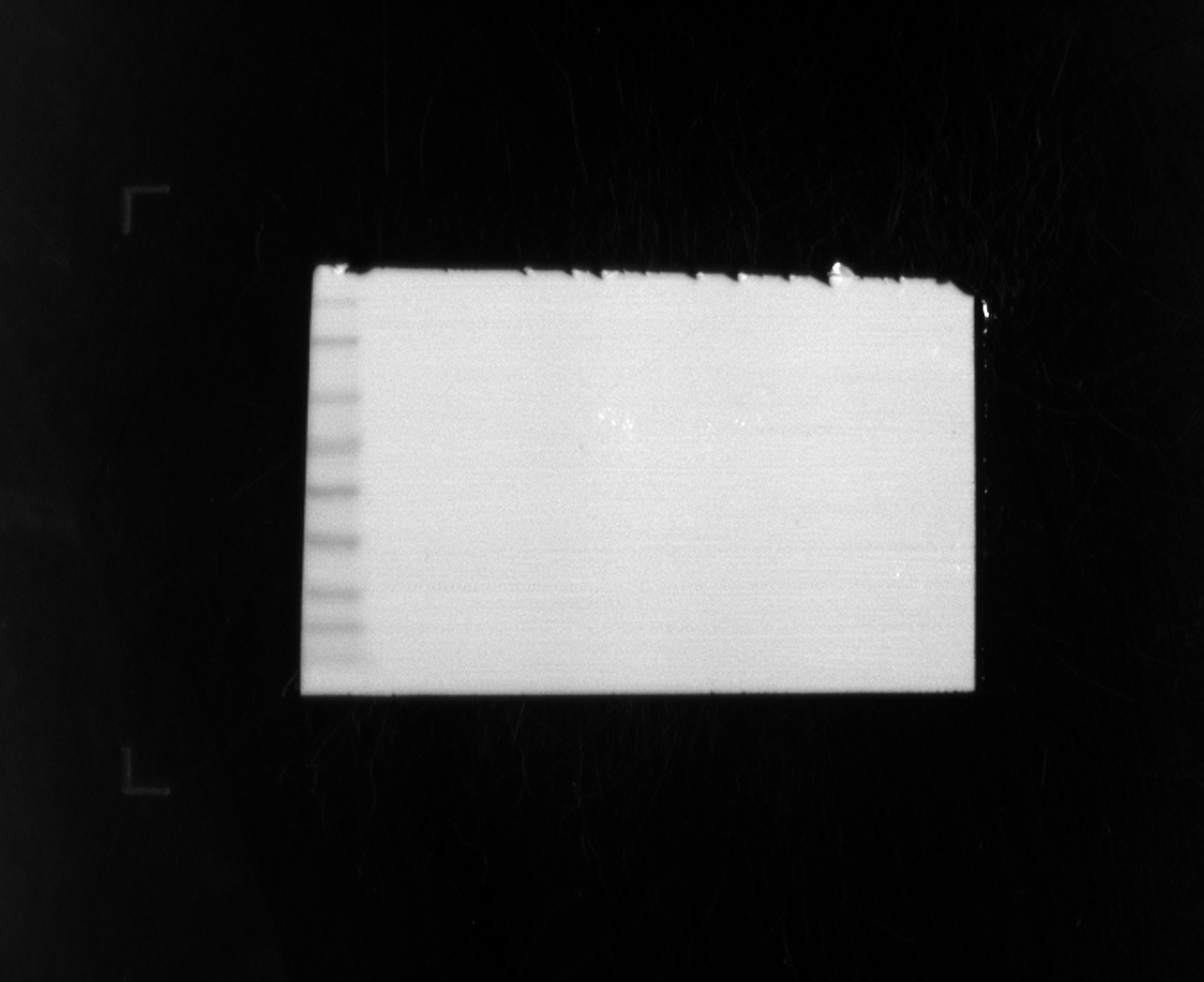

Supplement: Supplementary file 3 [file DataSheet2.zip › 5/PGC1A/marker.Tif]

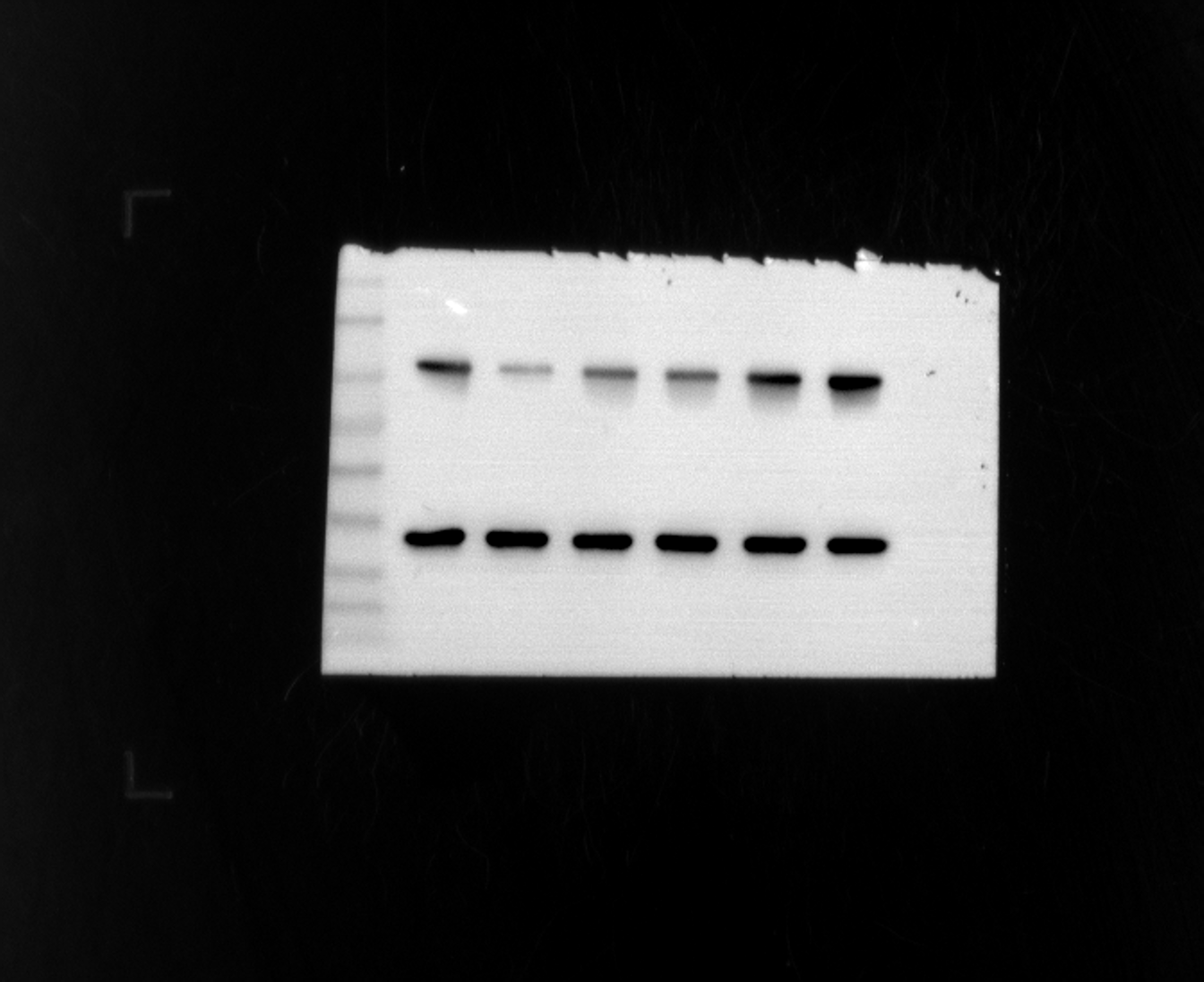

Supplement: Supplementary file 3 [file DataSheet2.zip › 5/PGC1A/merged 1s.Tif]

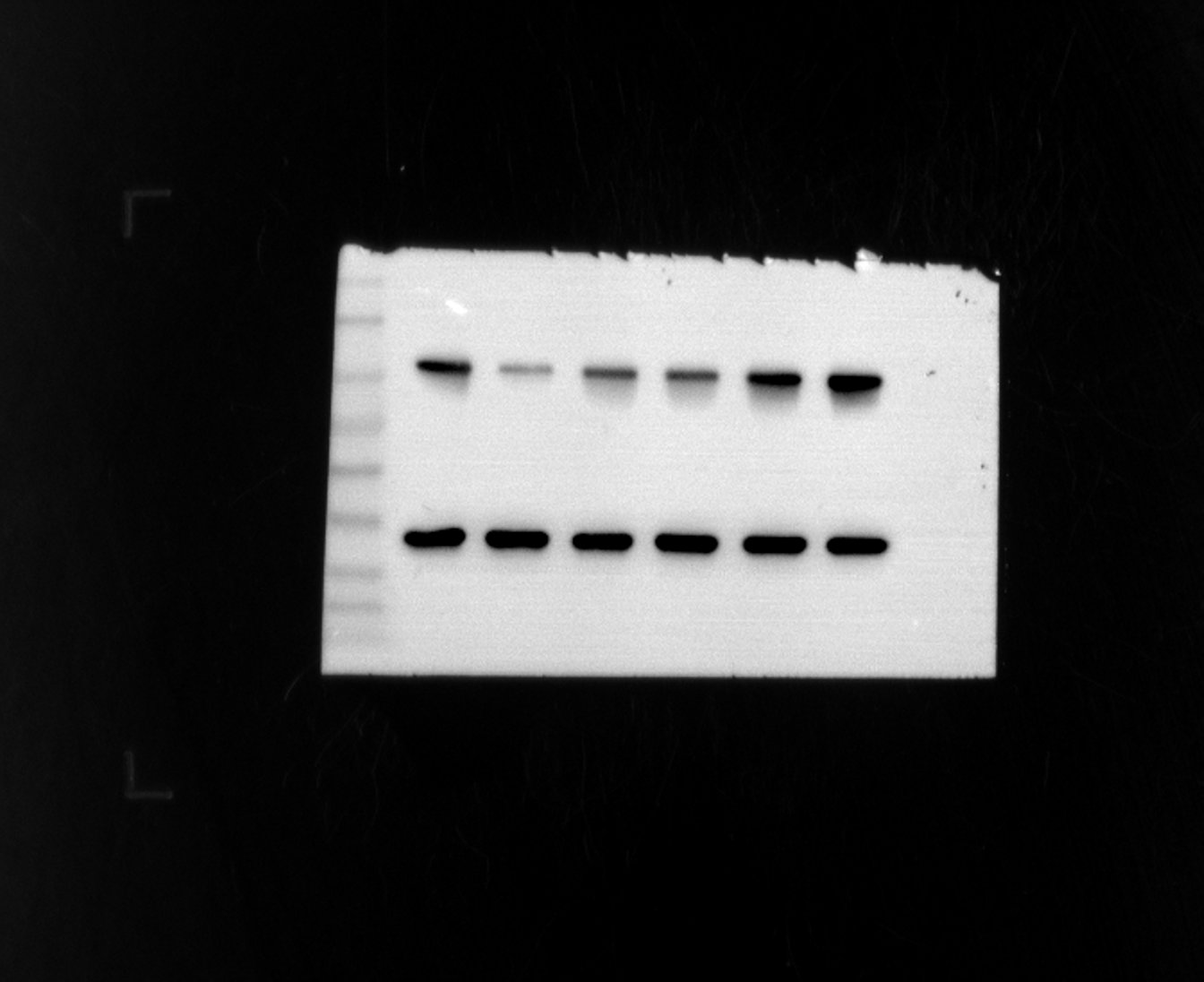

Supplement: Supplementary file 3 [file DataSheet2.zip › 5/PGC1A/merged 3s.Tif]

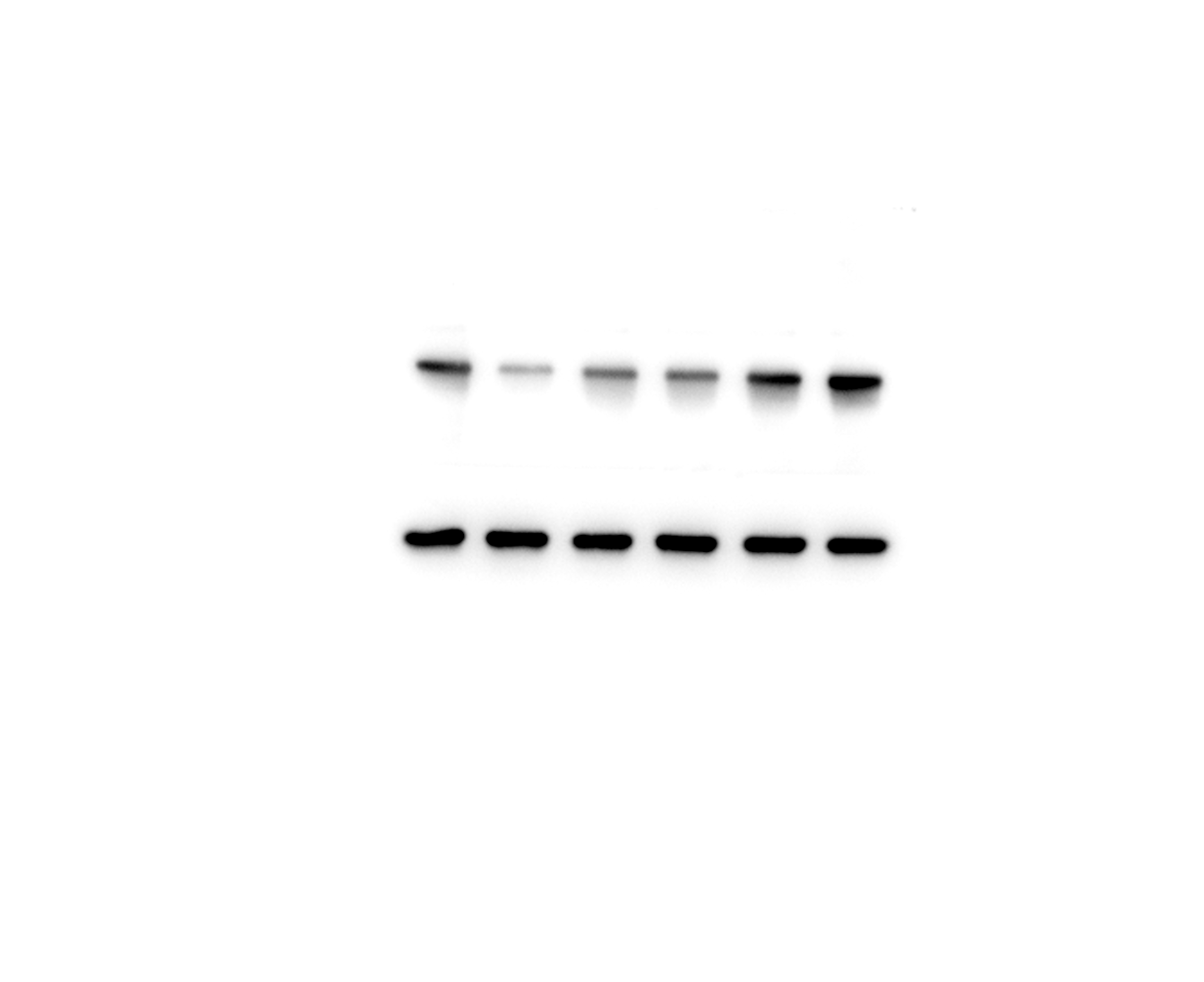

Supplement: Supplementary file 3 [file DataSheet2.zip › 5/PGC1A/PGC1A 1s.Tif]

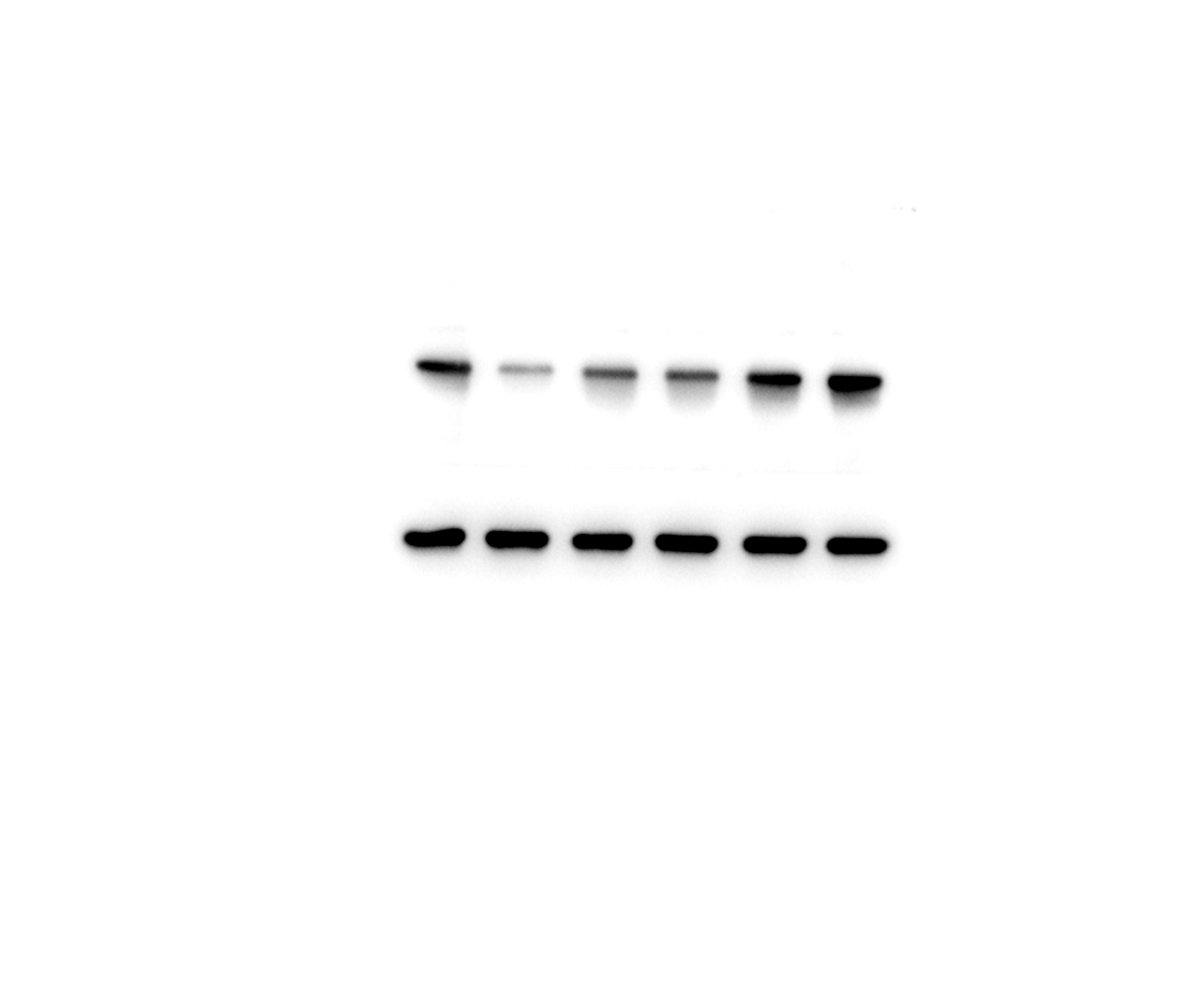

Supplement: Supplementary file 3 [file DataSheet2.zip › 5/PGC1A/PGC1A 3s.Tif]

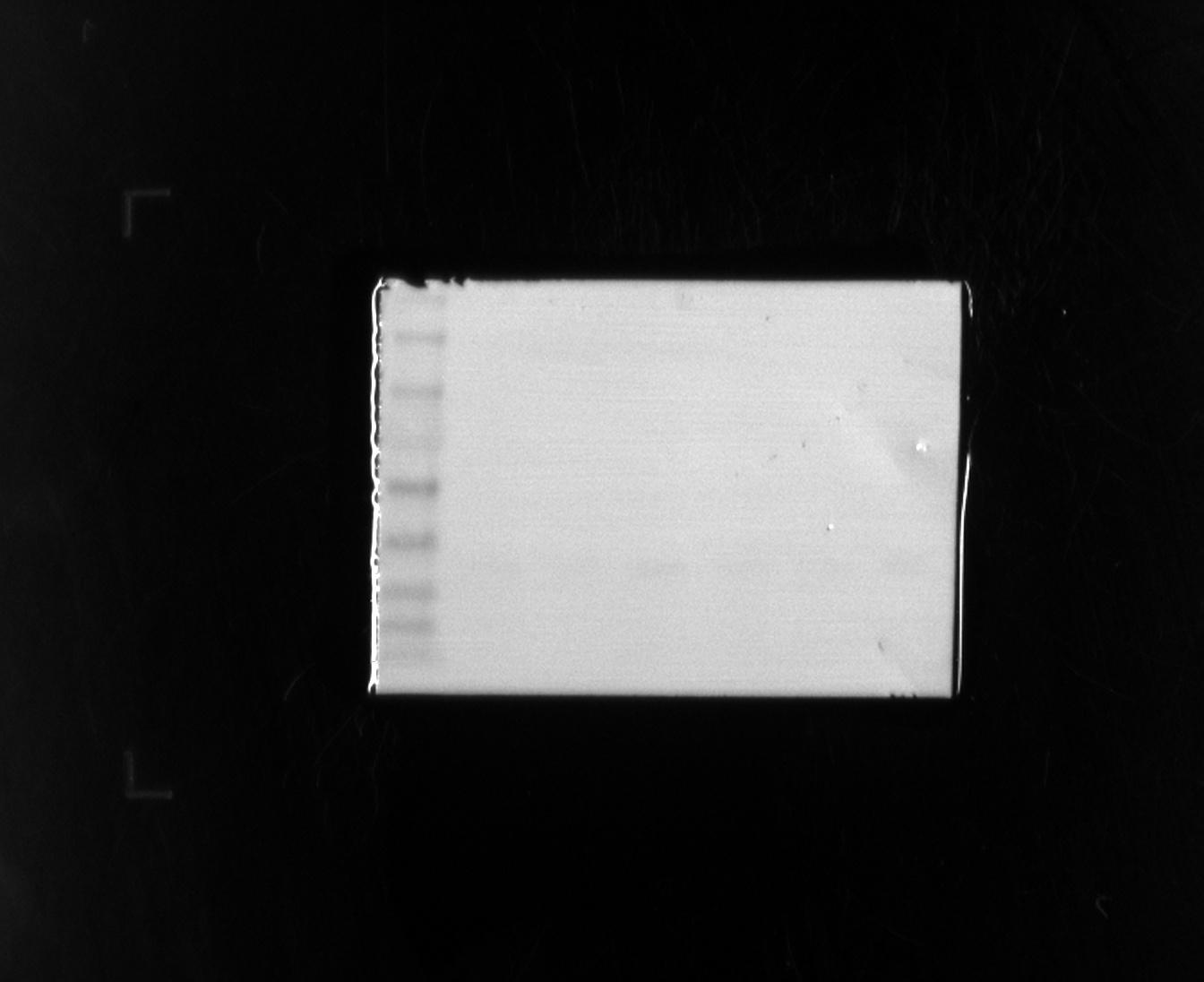

Supplement: Supplementary file 3 [file DataSheet2.zip › 5/SIRT1/marker.Tif]

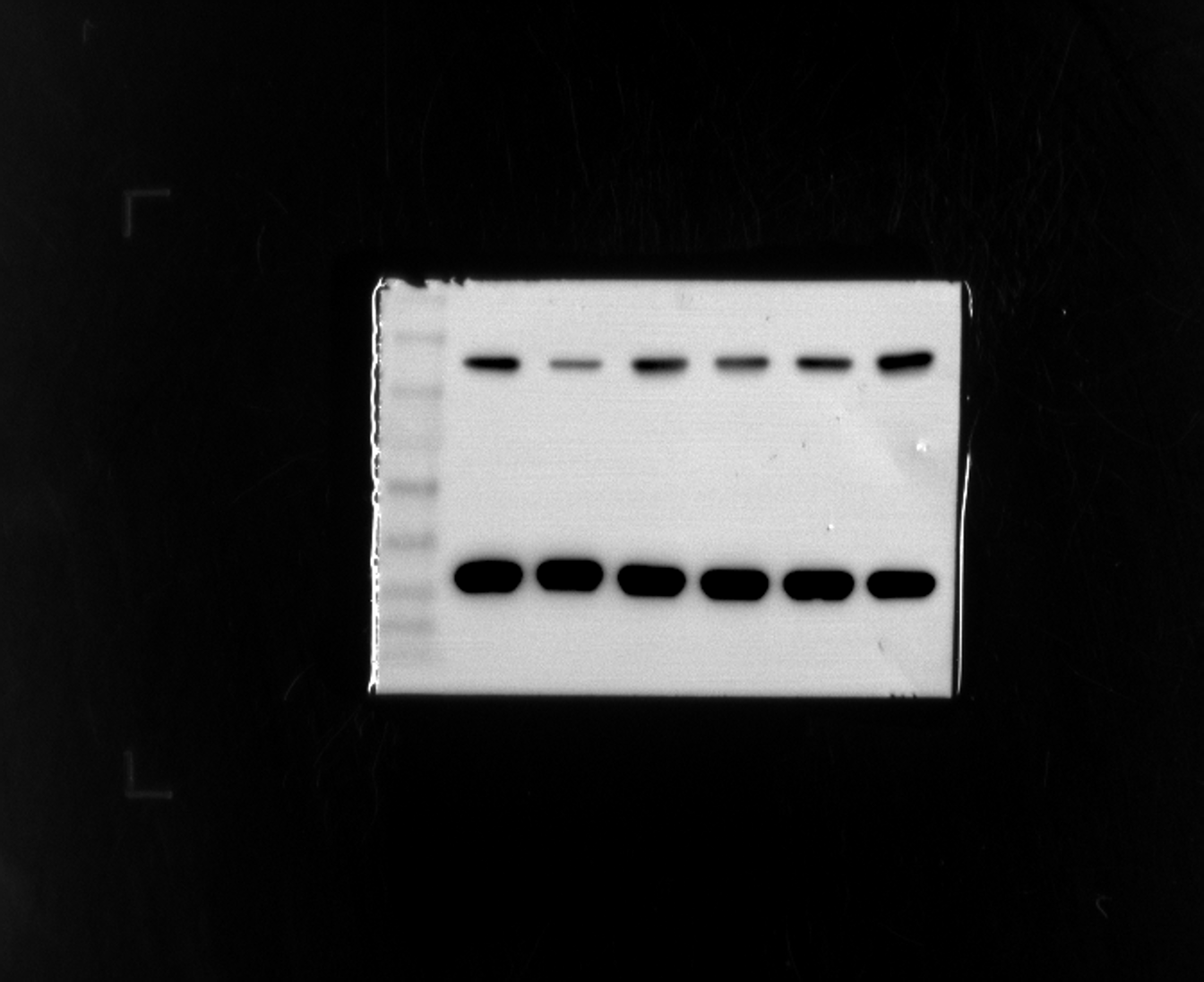

Supplement: Supplementary file 3 [file DataSheet2.zip › 5/SIRT1/mergerd 1s.Tif]
