# Supplementary figures and images for: Identification of mitochondria-related key genes in type 2 diabetes mellitus and elucidation of the Zhimu-Huangbai herb Pair’s mechanism: an integrated approach of bioinformatics, machine learning, and experimental validation (part 2 of 2)
Source: Front Cell Dev Biol. 2026 Mar 13;14:1763178. doi: 10.3389/fcell.2026.1763178 (PMC13021634; doi:10.3389/fcell.2026.1763178)

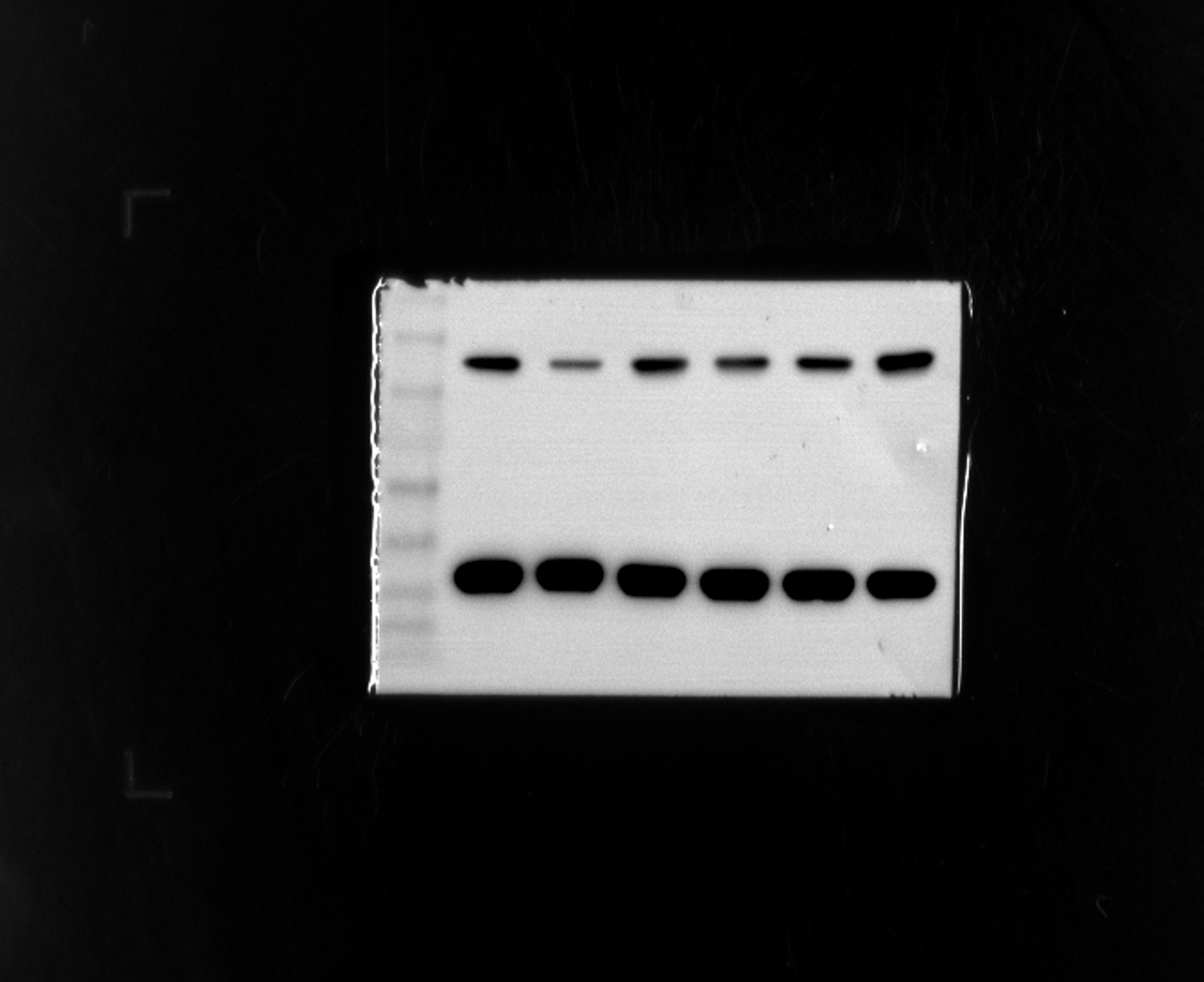

Supplement: Supplementary file 3 [file DataSheet2.zip › 5/SIRT1/mergerd 3s.Tif]

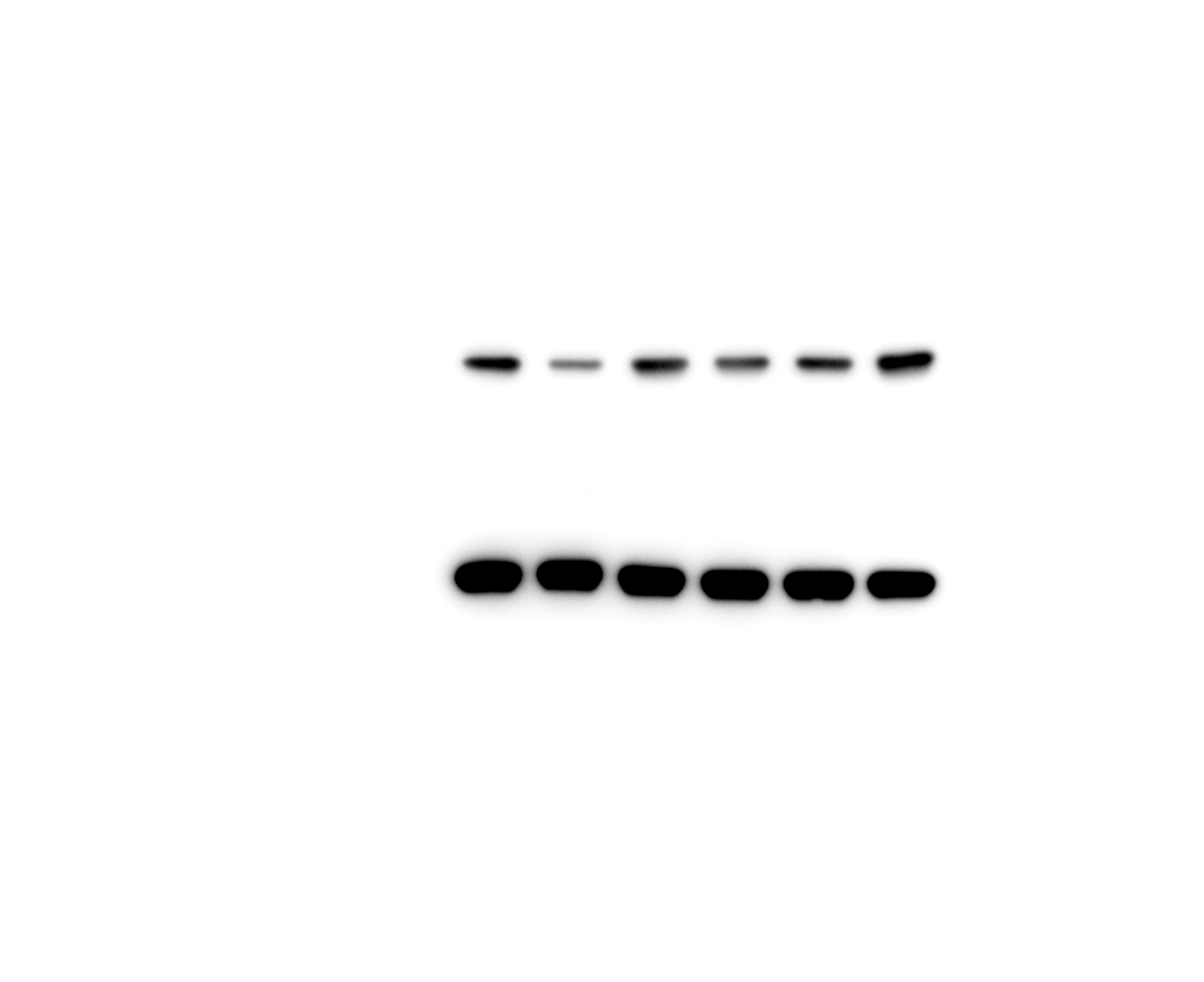

Supplement: Supplementary file 3 [file DataSheet2.zip › 5/SIRT1/SIRT1 1s.Tif]

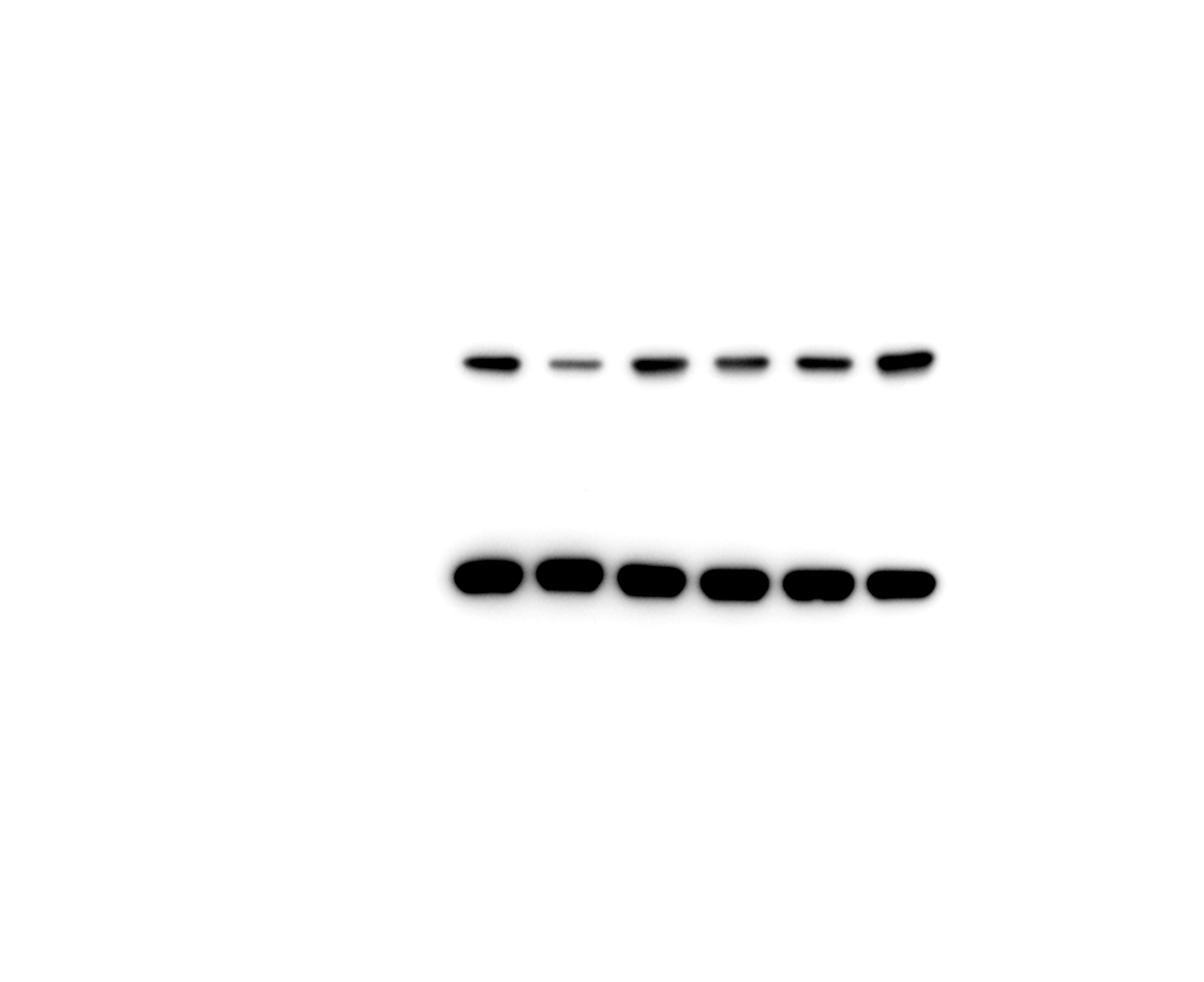

Supplement: Supplementary file 3 [file DataSheet2.zip › 5/SIRT1/SIRT1 3s.Tif]

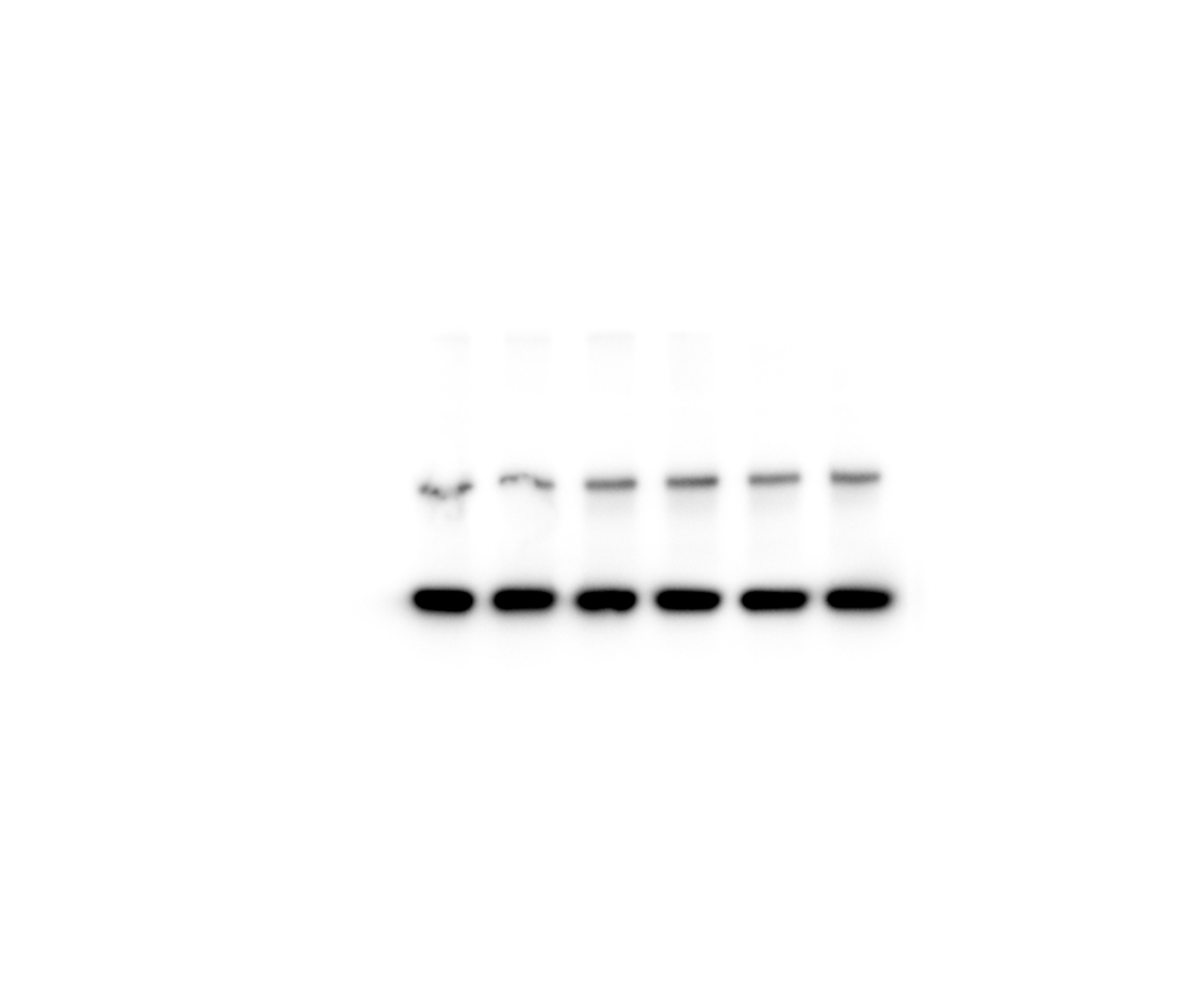

Supplement: Supplementary file 3 [file DataSheet2.zip › 6/ampk/ampk 1s.Tif]

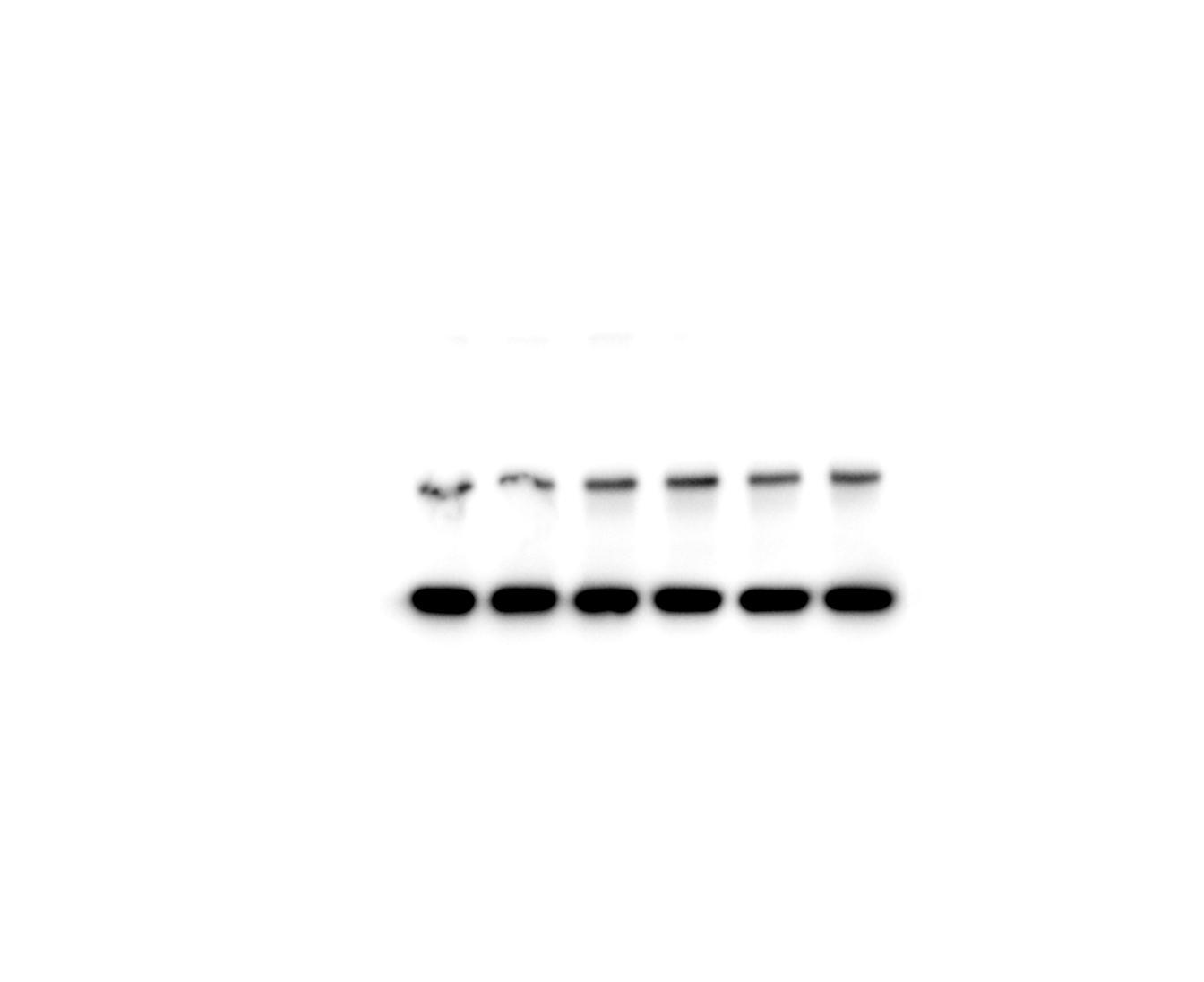

Supplement: Supplementary file 3 [file DataSheet2.zip › 6/ampk/ampk 3s.Tif]

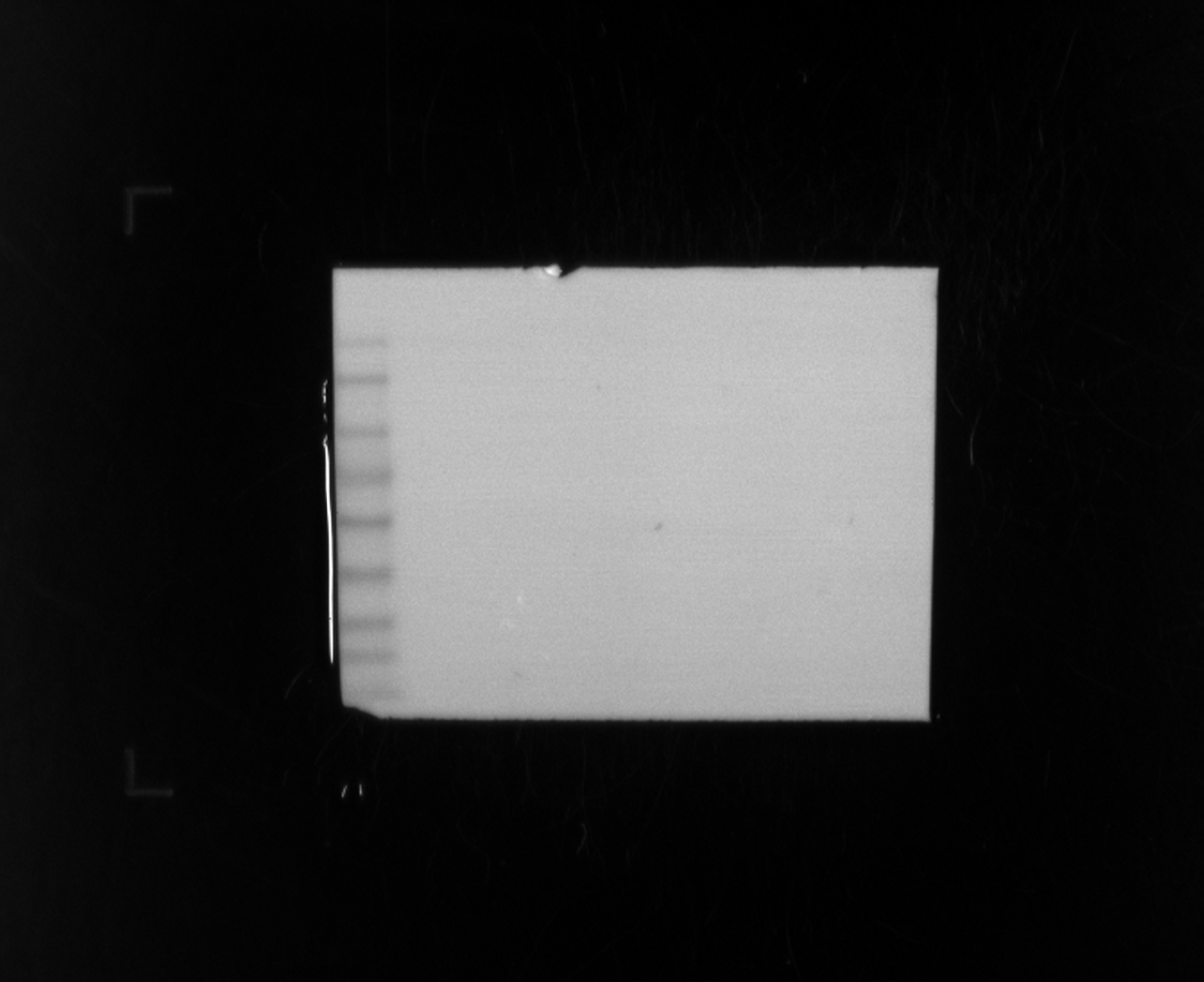

Supplement: Supplementary file 3 [file DataSheet2.zip › 6/ampk/marker.Tif]

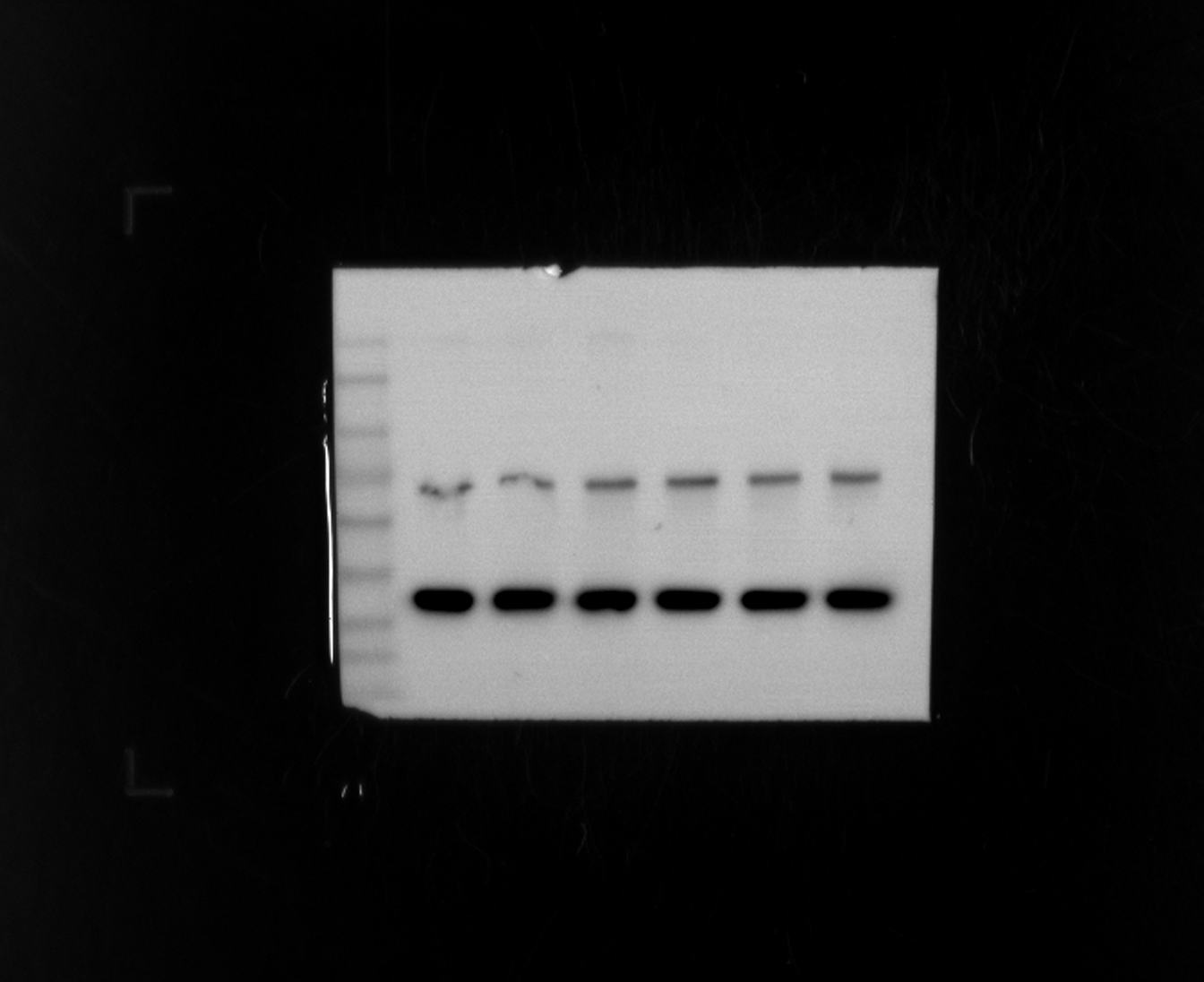

Supplement: Supplementary file 3 [file DataSheet2.zip › 6/ampk/merged 2s.Tif]

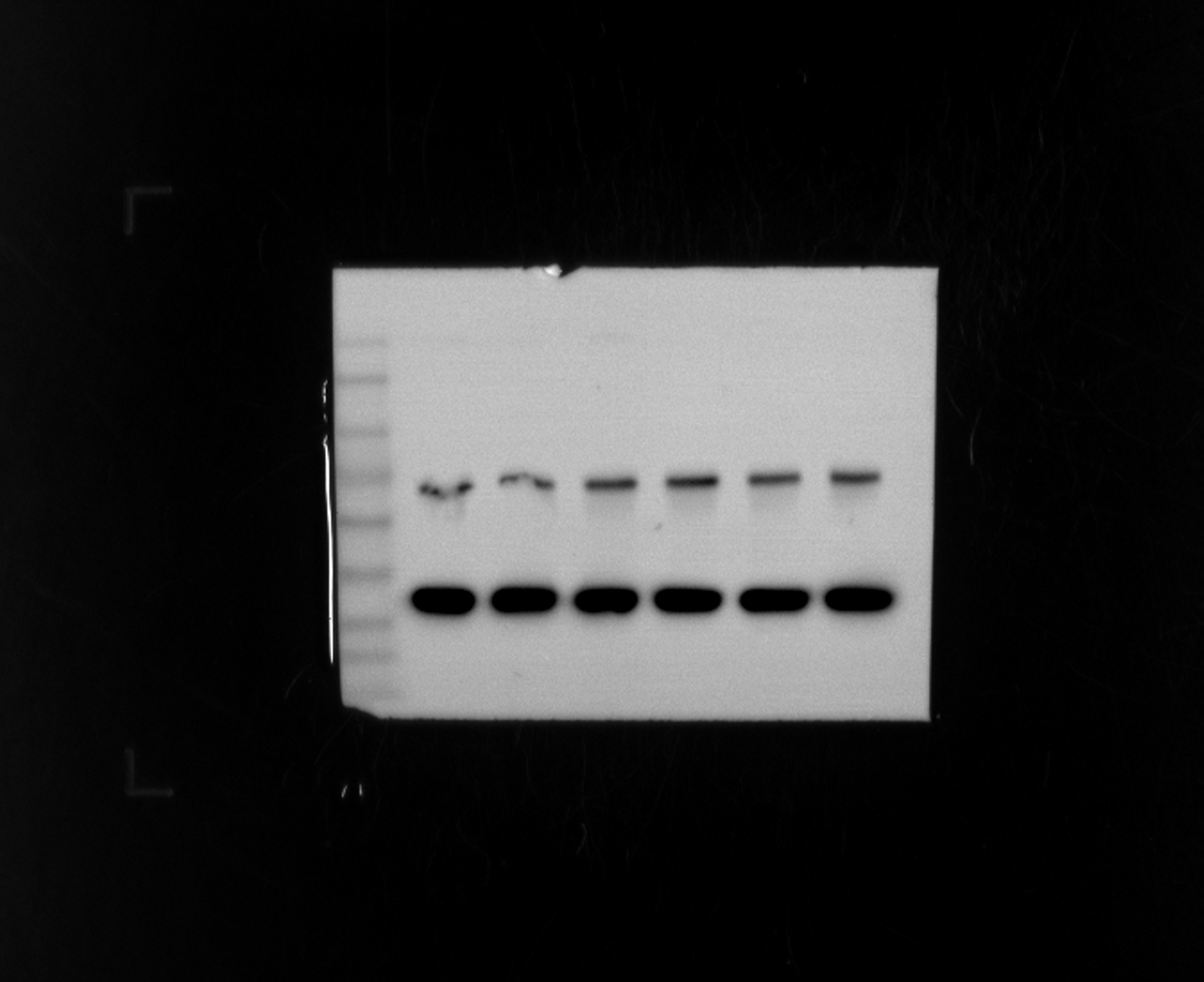

Supplement: Supplementary file 3 [file DataSheet2.zip › 6/ampk/merged 3s.Tif]

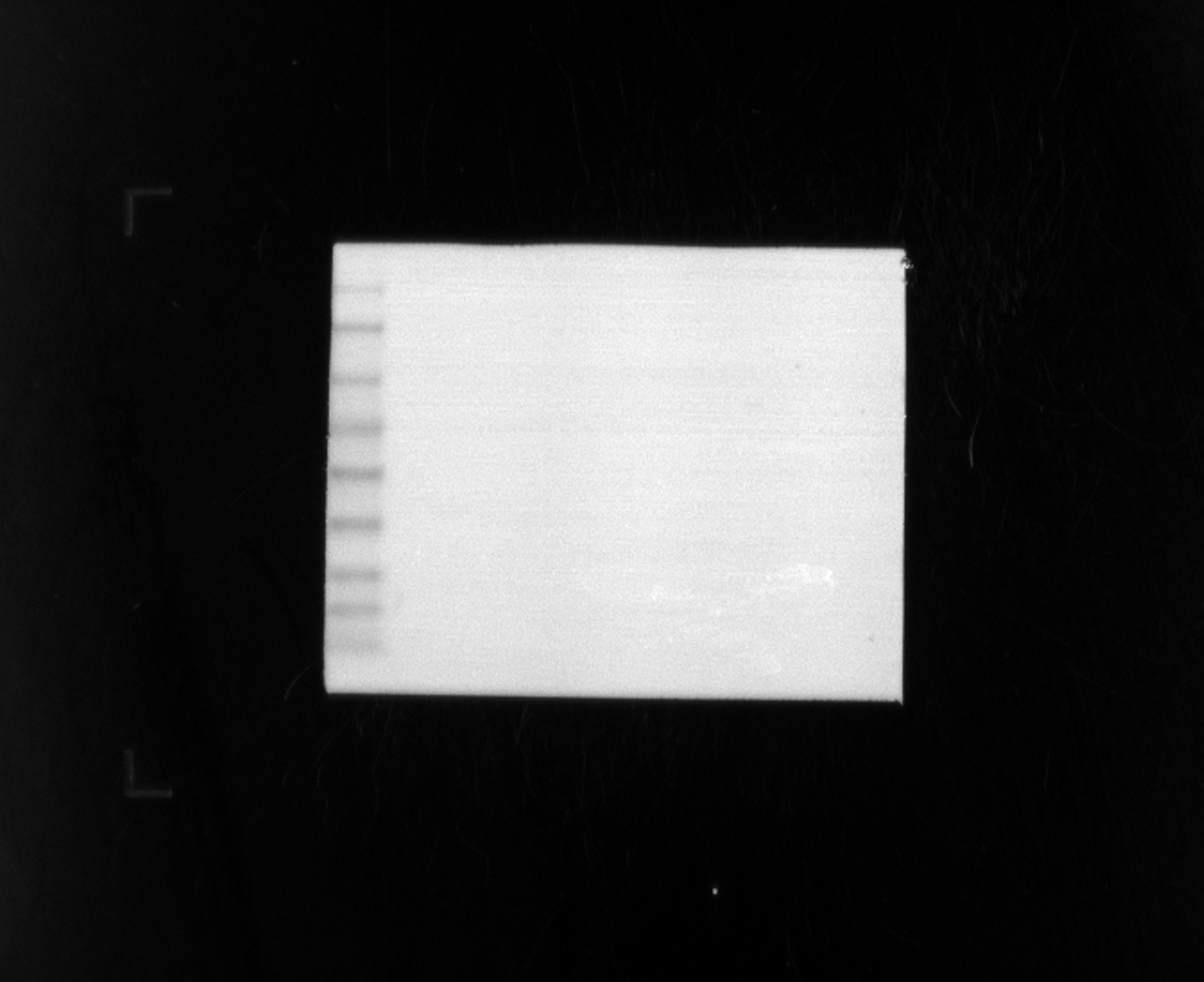

Supplement: Supplementary file 3 [file DataSheet2.zip › 6/PGC1A/marker.Tif]

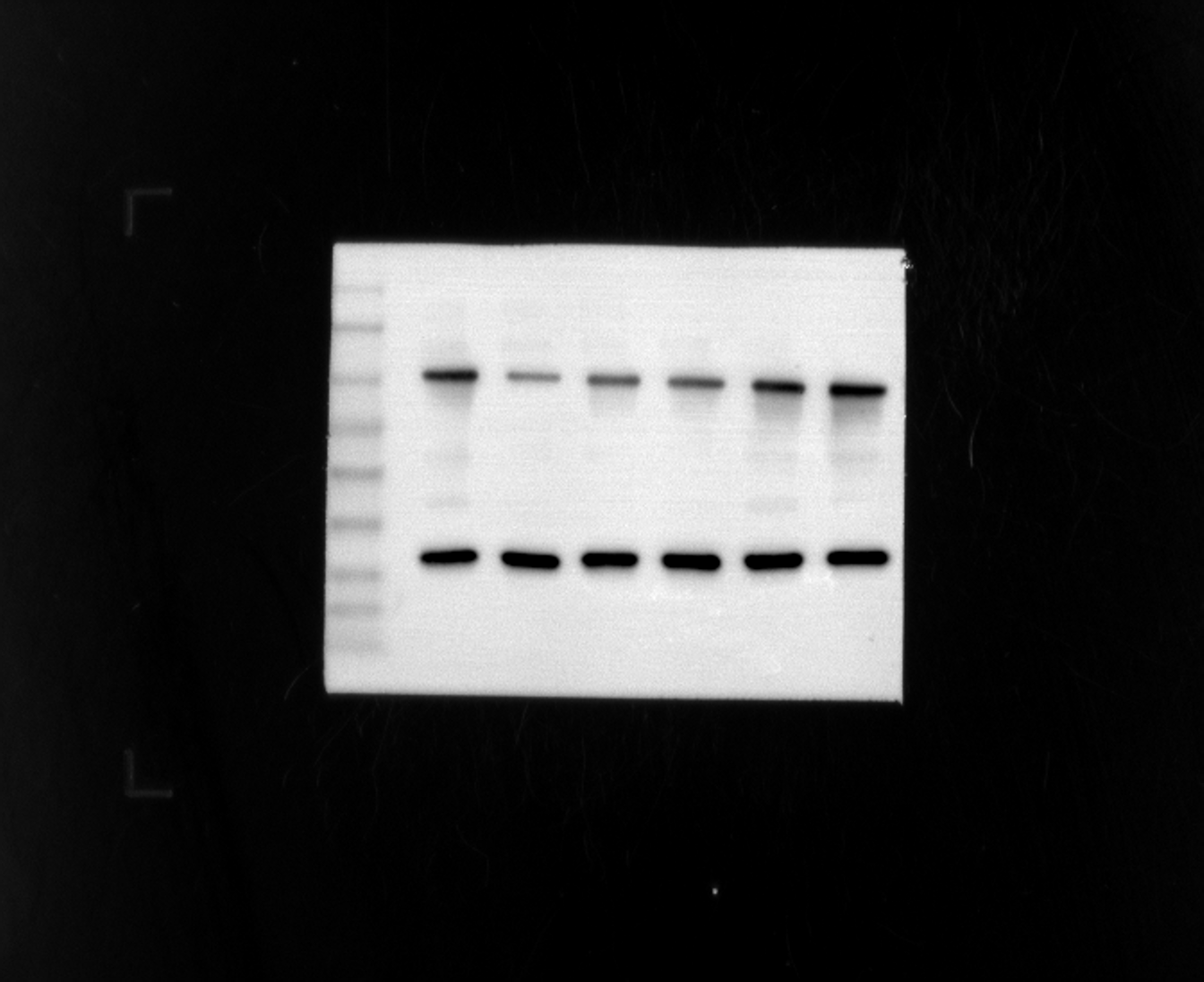

Supplement: Supplementary file 3 [file DataSheet2.zip › 6/PGC1A/merged 1s.Tif]

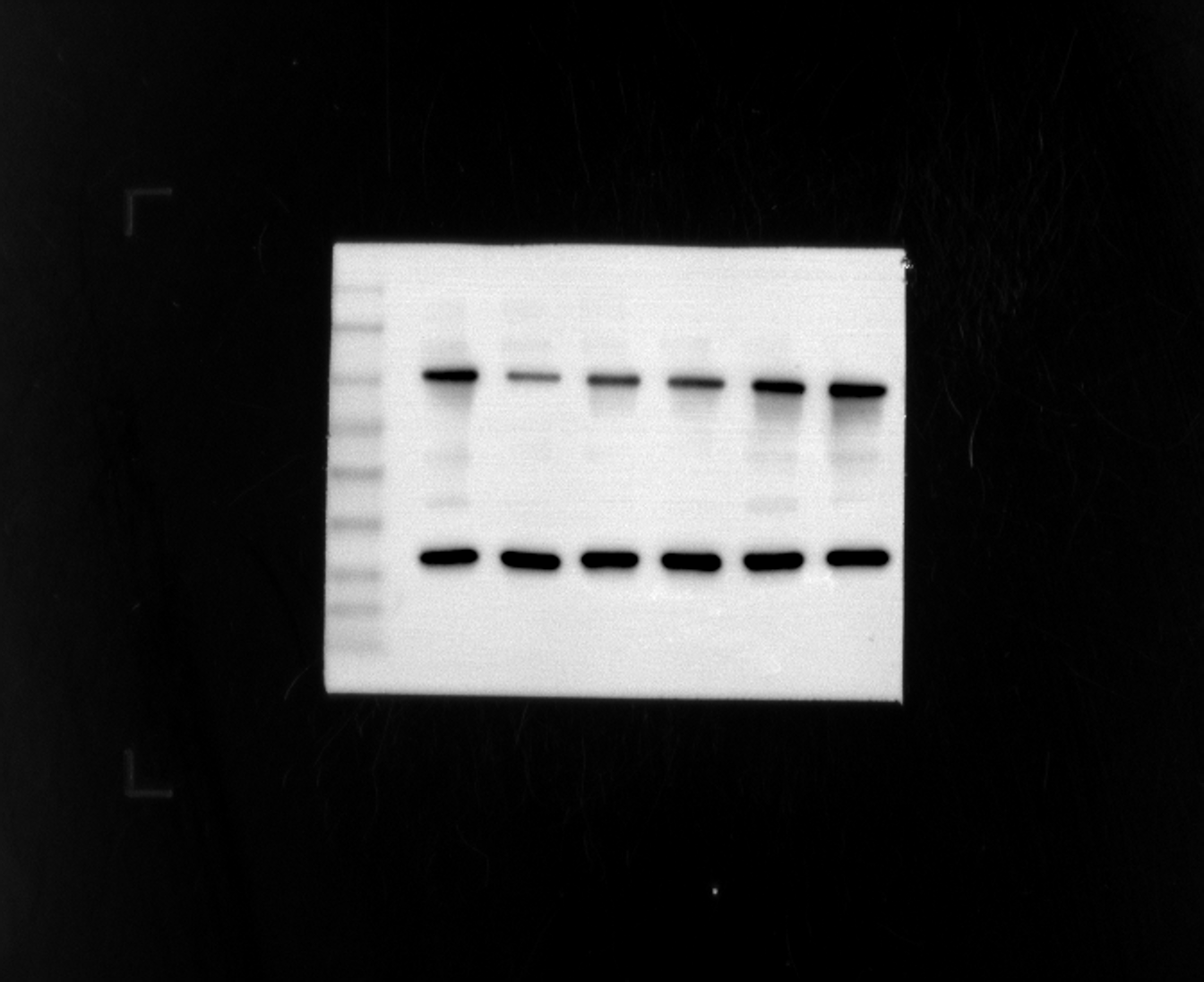

Supplement: Supplementary file 3 [file DataSheet2.zip › 6/PGC1A/merged 3s.Tif]

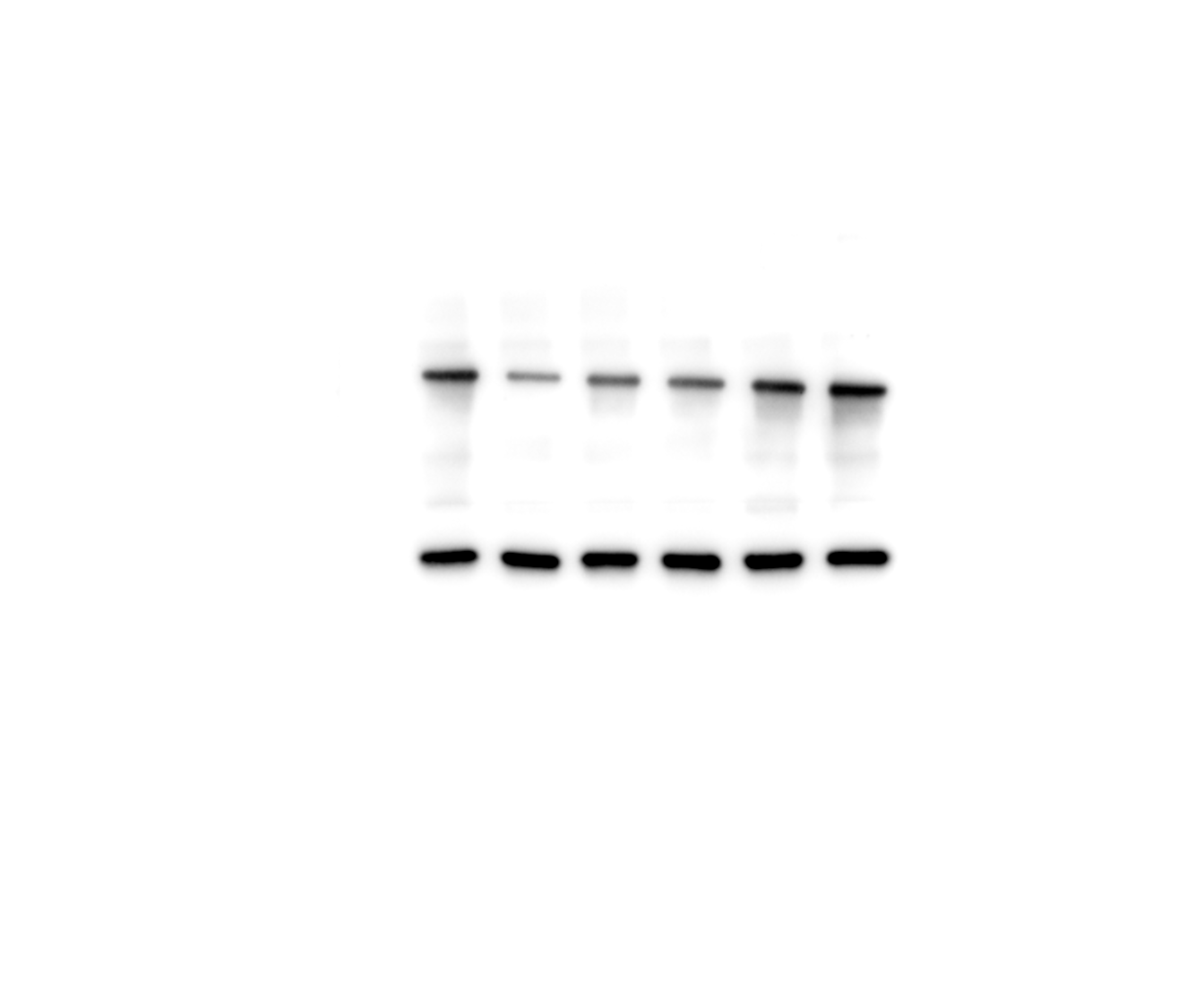

Supplement: Supplementary file 3 [file DataSheet2.zip › 6/PGC1A/PGC1A 1s.Tif]

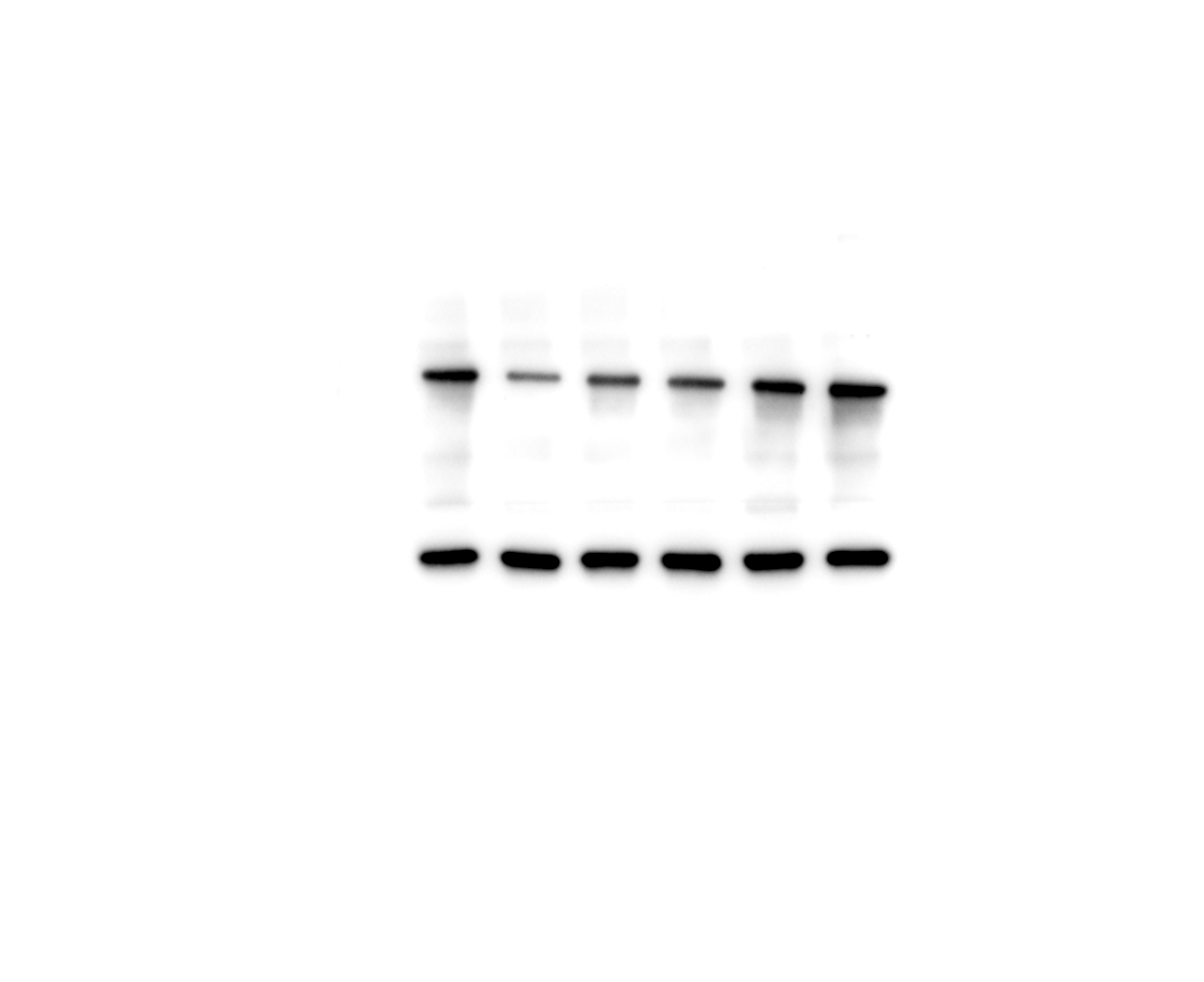

Supplement: Supplementary file 3 [file DataSheet2.zip › 6/PGC1A/PGC1A 3s.Tif]

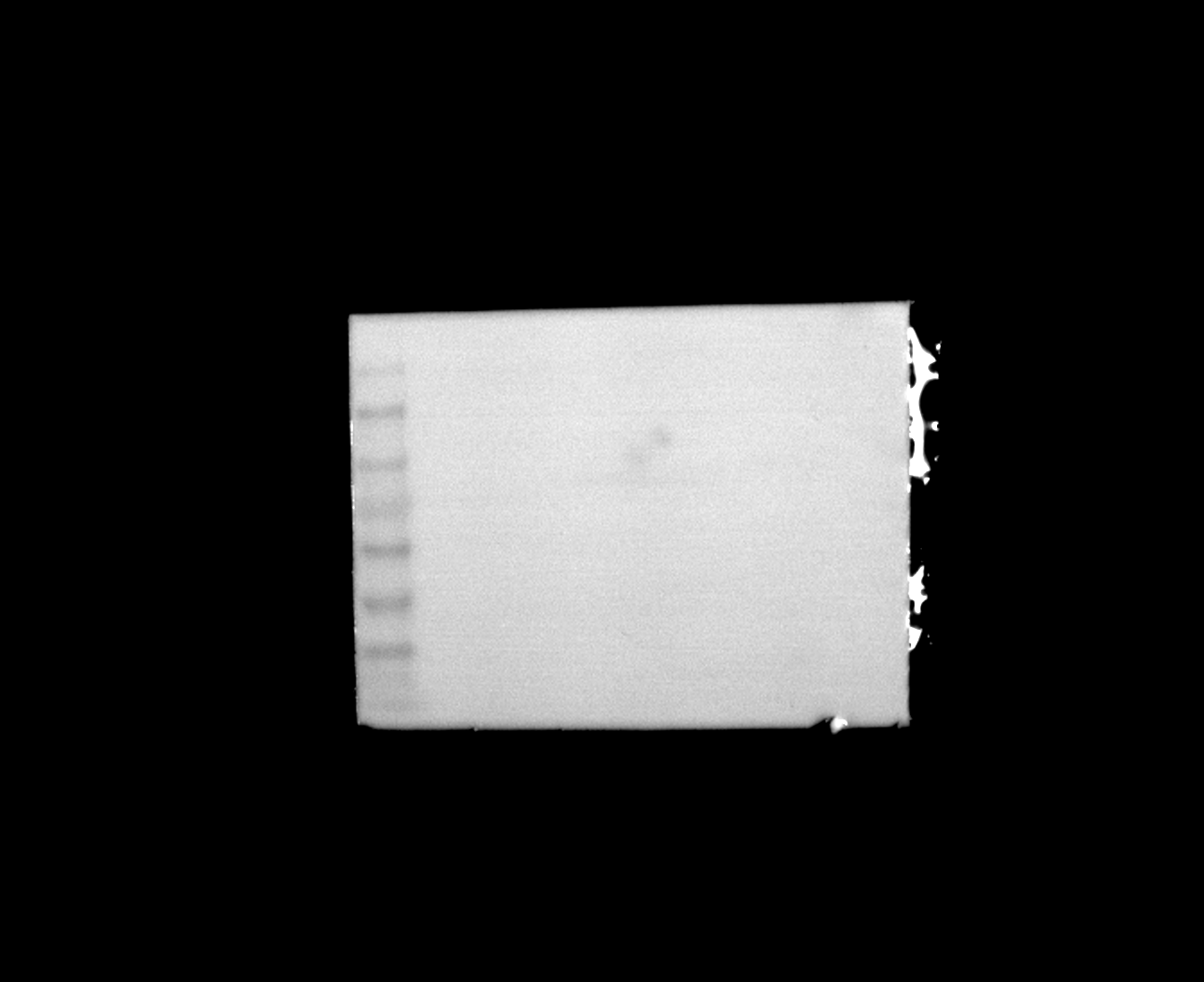

Supplement: Supplementary file 3 [file DataSheet2.zip › 6/SIRT1/marker.Tif]

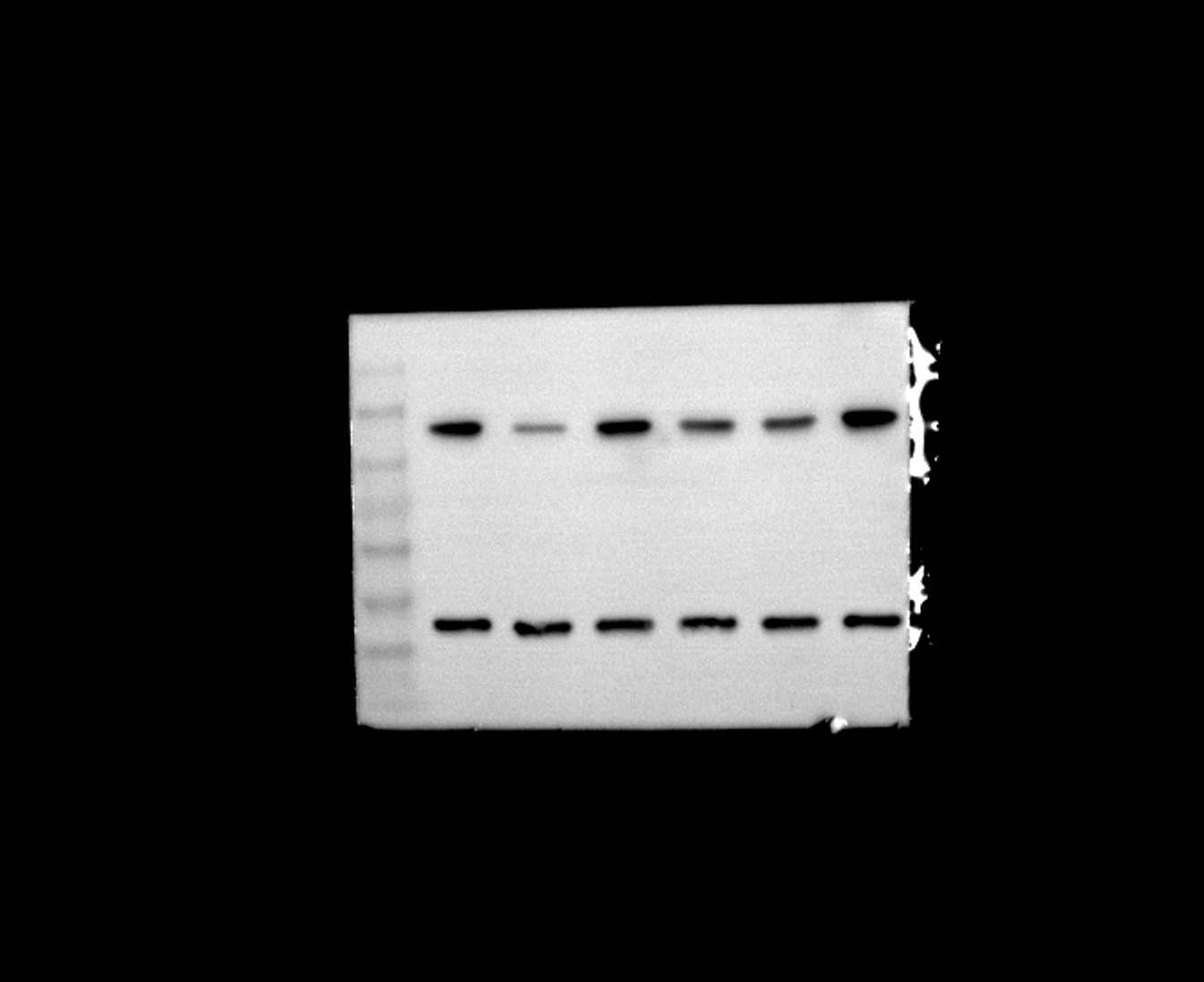

Supplement: Supplementary file 3 [file DataSheet2.zip › 6/SIRT1/merged 1s.Tif]

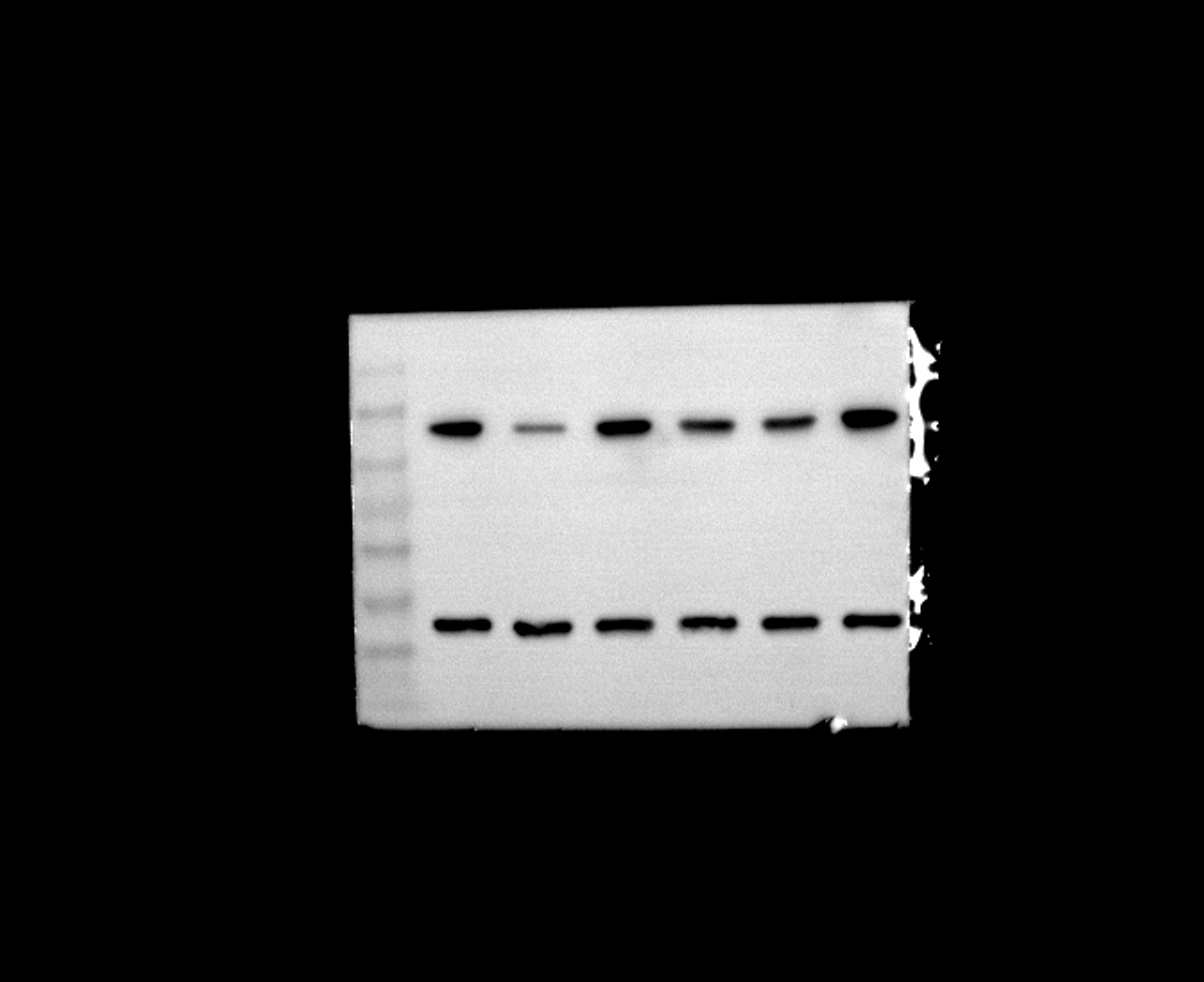

Supplement: Supplementary file 3 [file DataSheet2.zip › 6/SIRT1/merged 3s.Tif]

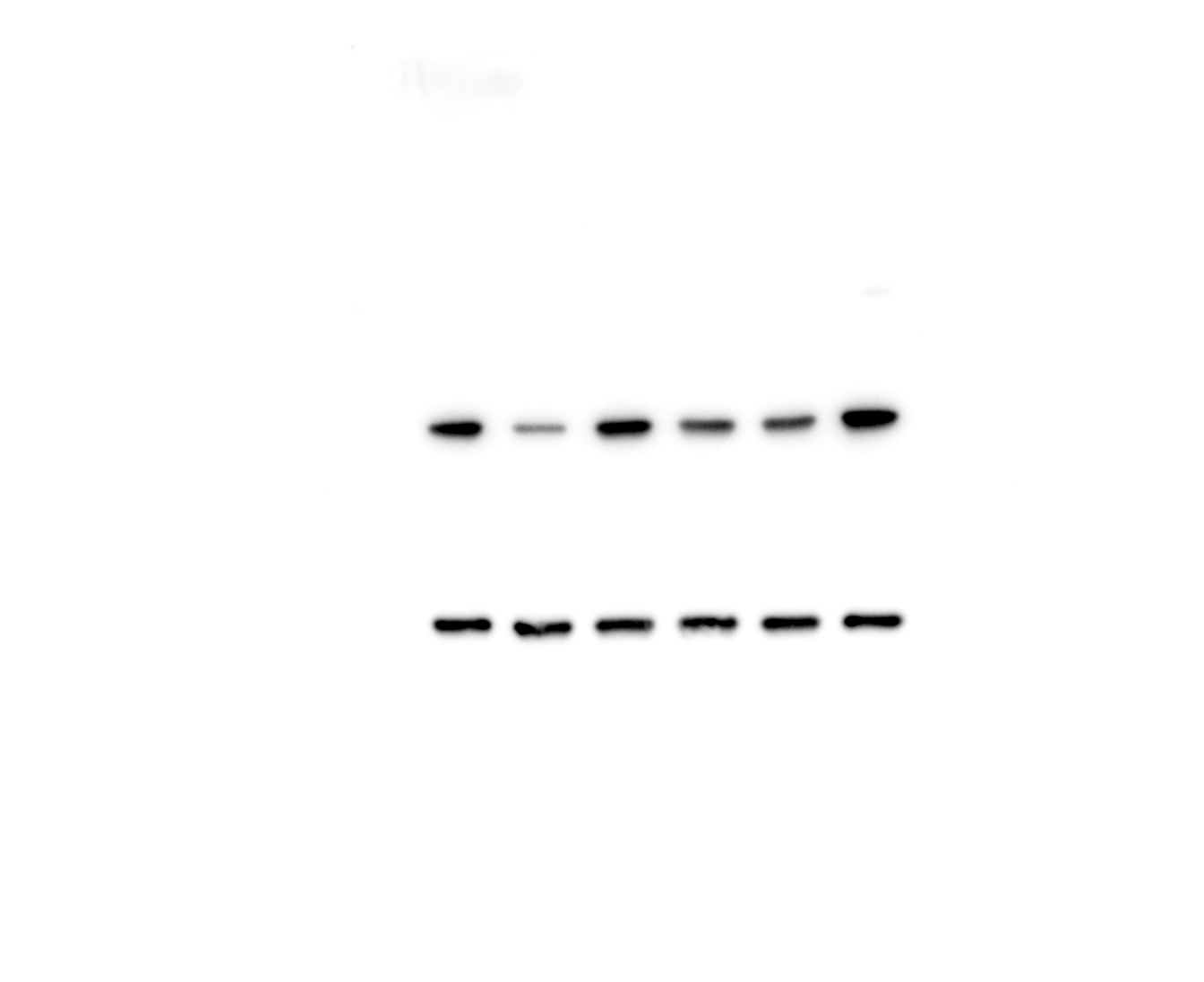

Supplement: Supplementary file 3 [file DataSheet2.zip › 6/SIRT1/SIRT1 1s.Tif]

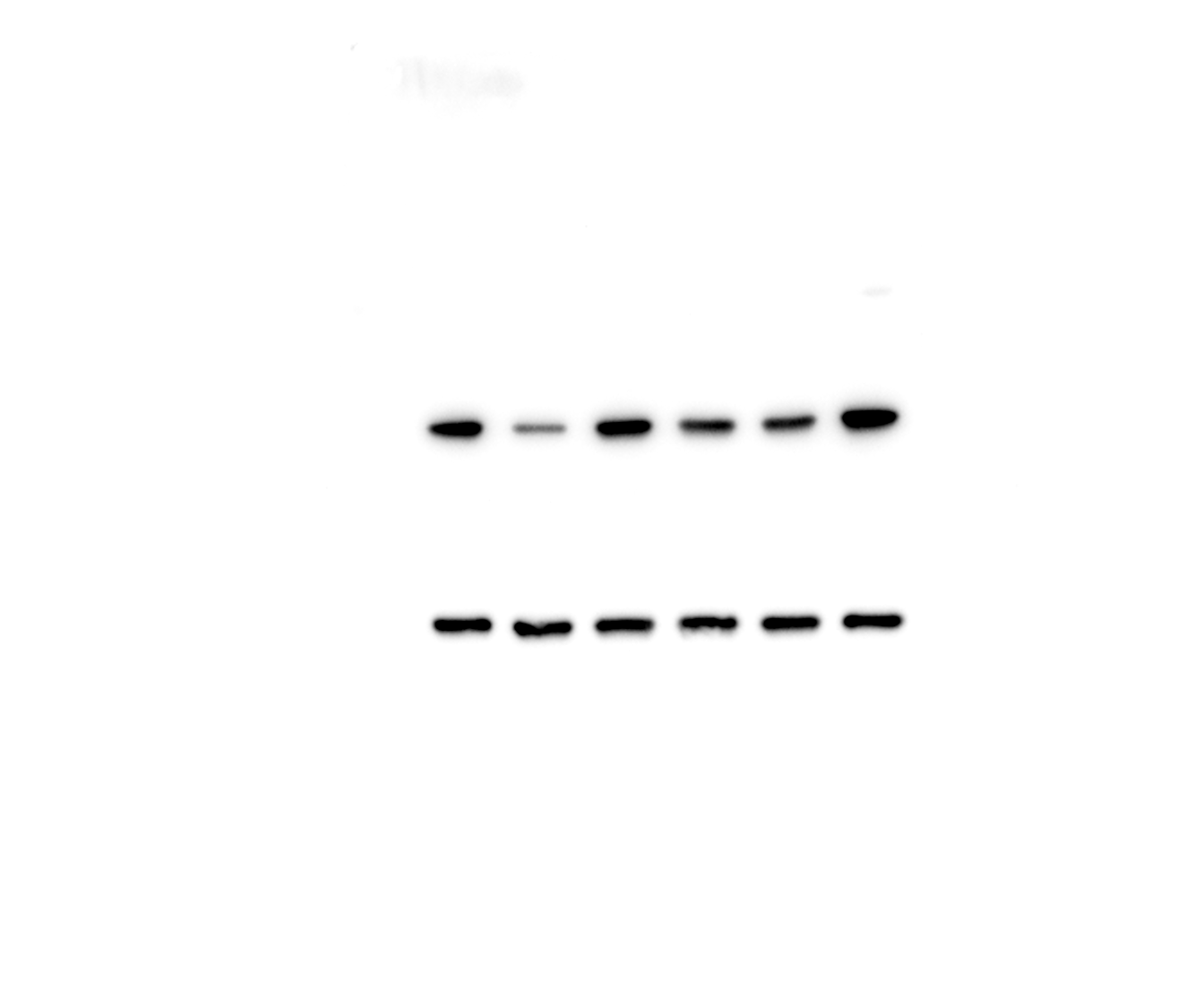

Supplement: Supplementary file 3 [file DataSheet2.zip › 6/SIRT1/SIRT1 3s.Tif]
